# Discovery and Optimization of Novel Nonhydroxamate LpxC Inhibitors for the Treatment of Multidrug-Resistant Gram-Negative Infections

**DOI:** 10.1021/acs.jmedchem.6c01036

**Published:** 2026-08-04

**Authors:** David P. Martin, Min Teng, Baskar Nammalwar, Christian Perez, Xiaoming Li, Jason Munguia, Konstantin Taganov, Junhua Fan, Sanjay Agarwalla, David Lonergan, Andrew P. Tomaras, Zachary Zimmerman, David T. Puerta

**Affiliations:** Blacksmith Medicines, Inc., San Diego, California 92121, United States

## Abstract

This report summarizes the discovery and optimization of a novel series of nonhydroxamate inhibitors targeting LpxC, a Zn^2+^-dependent hydrolase that is essential for the survival of Gram-negative bacteria. Beginning with a 5-hydroxypyrimidin-4-one metal-binding pharmacophore, structure-based approaches were utilized to generate a series of potent inhibitors that exhibited activity against a wide variety of Enterobacterales, including both susceptible and multidrug-resistant pathogens, and efficacy in murine thigh infection models. These novel compounds were evaluated in a rat model of cardiovascular toxicity to demonstrate the safety of the scaffold relative to another LpxC inhibitor that proved unsuccessful in Phase I clinical trials. A variety of inhibitors with potent in vivo efficacy and no hemodynamic effects were identified, which constituted an initial suite of potential development candidates.

## Graphical abstract

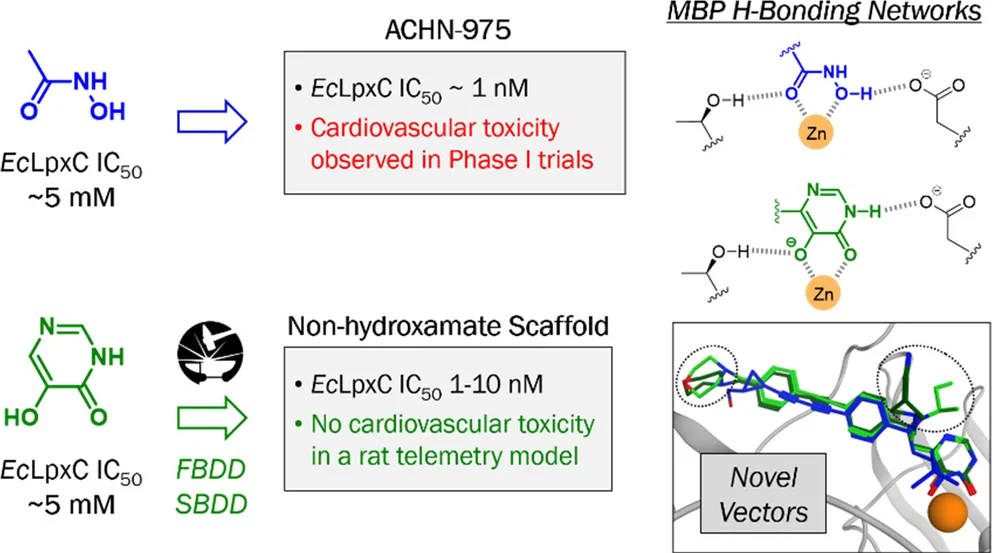

## Introduction

The emergence of microbial resistance to the current treatment options is one of the most significant public health threats of this century.^1^ Since 2017, the World Health Organization has been publishing lists of priority pathogens for therapeutic discovery based on a variety of parameters (e.g., mortality, impact on the global healthcare system, and prevalence of resistance) that are generally dominated by the so-called ESKAPE pathogens (*Enterococcus faecium*, *Staphylococcus aureus*, *Klebsiella* spp., *Acinetobacter baumannii*, *Pseudomonas aeruginosa*, and *Enterobacter* spp.).^2^ Infections caused by multidrug-resistant (MDR) Gram-negative bacteria, which include the latter four priority pathogens, are estimated to result in millions of deaths per year with that number projected to double by 2050 in the absence of new therapeutic interventions.^3^ Despite a significant and growing need for novel agents that overcome the developing resistance to existing drug classes,^4^ major hurdles exist that hamper the development of new antibacterial drugs that could help avert the worst-case scenarios.^5^

One novel antibacterial target is LpxC (UDP-3-*O*-(*R*-3-hydroxymyristoyl)-*N*-acetylglucosamine deacetylase), a Zn^2+^-dependent enzyme that catalyzes the first committed step in the biosynthesis of lipid A, a critical component of the outer membrane of Gram-negative bacteria (Figure 1A). Given its conservation across Gram-negative species and the lack of a human homologue, LpxC has been considered an attractive target since it was first characterized in the 1990s.^6,7^ The LpxC active site contains two flexible regions that are important for enzyme turnover and inhibitor binding (Figure 1B): a loop from “Insert I” (Thr60-Thr64 in the LpxC ortholog from *Escherichia coli*, *Ec*LpxC) closes over the metal center and glucosamine group of the substrate, while “Insert II” (Phe192-Ala215 in *Ec*LpxC), a β-α-β motif, forms a majority of the tunnel that accommodates the lipophilic acyl group of the substrate. The precise shape and conformational flexibility of these regions varies between LpxC orthologs from different species, which has been shown to have a significant influence on both the binding affinity and antibiotic spectrum of LpxC inhibitors.^8^

**Figure 1:**
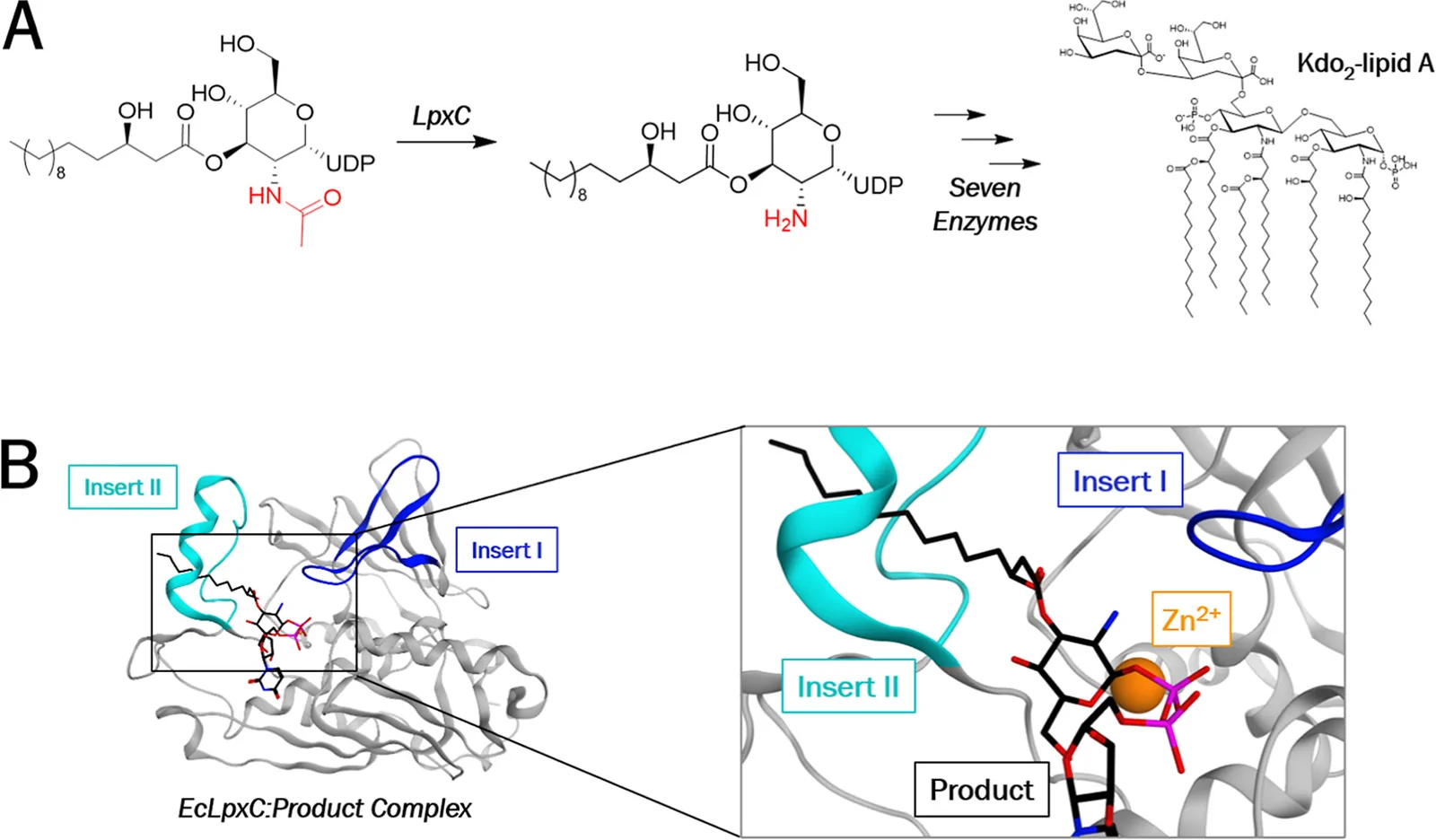
LpxC catalyzes the first committed step in the synthesis of Lipid A, a critical component of the outer membrane of Gram-negative bacteria (A). The structure of product-bound *Ec*LpxC (shown in black, PDB 4MDT) highlights two flexible regions of the active site that facilitate catalysis and are also critical to inhibitor binding (B): A loop from Insert I (dark blue) closes over the catalytic metal site (orange sphere), while Insert II (cyan) forms the lipophilic tunnel that binds the acyl group of the substrate.

Although LpxC has been pursued as an antibiotic target for over 35 years, a commercial drug targeting it has been elusive so far. Following pioneering work at Merck and British Biotech,^9,10^ Chiron Pharmaceuticals identified CHIR-090 (**1**, Figure 2A),^9^ which embodies the broad chemotype followed by most subsequent LpxC inhibitors: (1) A metal-binding pharmacophore (MBP) to engage the active site Zn^2+^ ion, (2) a hydrophobic backbone to occupy the lipid binding tunnel, (3) a linker between the MBP and backbone that can be elaborated to interact with the glucosamine binding pocket, and (4) a hydrophilic tail at the end of the backbone to interact with residues at the entrance to the lipid binding tunnel and enhance physicochemical properties. A wide variety of academic and industrial research programs followed that aimed to improve the potency, selectivity, and drug-like properties of LpxC inhibitor scaffolds.^11–13^ As is the case with most metalloenzymes, the LpxC inhibitor literature is dominated by scaffolds based on the hydroxamic acid (HA) MBP, which has often been reported to have significant safety liabilities associated with it.^14,15^

**Figure 2:**
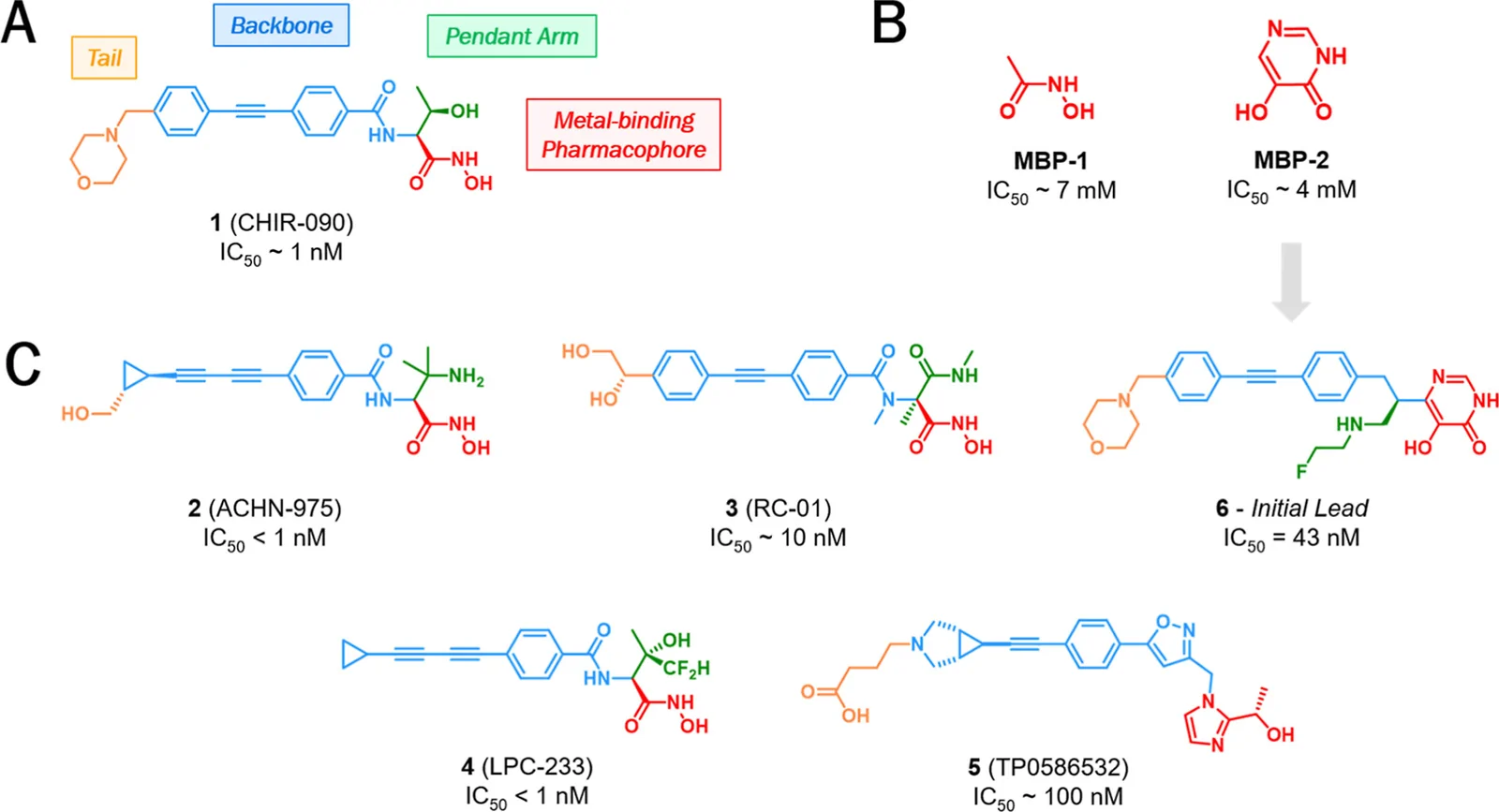
Structures of LpxC inhibitors. Compound **1** established the chemotype followed by most subsequent scaffolds (A). A vast majority of the efforts have been focused on scaffolds that utilize the HA MBP (**MBP-1**), but other starting points, including 5-hydroxypyrimidin-4-one (**MBP-2**), bind with similar affinity (B). The LpxC inhibitor scaffold discussed here, which originated with initial lead **6**, is highly differentiated from the two HA-based LpxC inhibitors (**2** and **3**) that were discontinued in Phase I clinical trials due to safety findings and two more recent inhibitors (**4** and **5**) that arose from efforts to design LpxC inhibitors with improved safety (C). IC_50_ values shown are those against LpxC from *E. coli* (*Ec*LpxC).

To date, two HA-based LpxC inhibitors, ACHN-975 (**2**, Figure 2C) and RC-01 (**3**), have progressed to Phase I trials but both efforts were terminated prematurely due to safety findings (NCT01870245 and NCT03832517, respectively). In the case of **2**, hypotension was observed that was associated with the maximal drug exposure in the plasma (*C*_max_); the basic primary amine moiety on the pendant arm of the molecule was later implicated as the driver of this cardiovascular (CV) toxicity.^16^ Notably, further optimization of HA-based scaffolds has also produced the inhibitor LPC-233 (**4**), which has demonstrated efficacy in various in vivo models and has potential for improved safety.^17,18^ Although the HA functional group has not been directly implicated in the safety findings for either **2** or **3**, there is a recognized need for the development of LpxC inhibitors based on alternative MBPs as well.^15,19^ Recent efforts at Taisho Pharmaceutical have led to the discovery of TP0586532 (**5**), which utilizes an imidazole-based MBP, which has exhibited efficacy against carbapenem-resistant *K. pneumoniae* in vivo with reasonable projected human doses.^20–22^

The novel, non-HA LpxC inhibitors discussed herein are a product of Blacksmith Medicines’ proprietary fragment-based drug discovery approach targeting metalloenzymes. The campaign to target LpxC began from the observation that 5-hydroxypyrimidin-4-one (**MBP-2**, Figure 2B) binds to the Zn^2+^ ion of LpxC with similar affinity to acetohydroxamic acid (**MBP-1**). An initial chemistry campaign elaborating from the fragment **MBP-2** led to the discovery of **6**, which was evaluated for its in vitro activity and in vivo efficacy in a variety of preclinical infection models (e.g., sepsis, thigh, and urinary tract). Although this molecule served as validation of the platform and scaffold, it had multiple limitations and liabilities (e.g., limited antibacterial spectrum, excessively high dose level requirements for efficacy, and the potential for metabolism to fluoroacetate, a known toxic compound),^23^ which prompted further exploration of the scaffold to alleviate those concerns. This paper describes the results of a follow-on lead optimization campaign focused on increasing intrinsic potency, expanding the antibiotic spectrum of the scaffold, decreasing the doses required for in vivo efficacy, and derisking the program with respect to the CV toxicity observed during treatment with **2**. While several hundred compounds were designed, synthesized, and evaluated before a qualified lead compound was identified, a more limited set of compounds are discussed in this paper to demonstrate some of the challenges are overcome during early lead optimization.

## Results and Discussion

The crystal structure of initial lead **6** bound to LpxC shows that it occupies the same binding sites as previously reported HA-based inhibitors, with its hydroxypyrimidone MBP acting as a bidentate donor to the active site Zn^2+^ (Figure 3A), its diphenylacetylene backbone occupying the substrate binding tunnel, and the pendant amine in a similar position to that of **2** (Figure 3B). Electron density corresponding to **6** was well-resolved in the active site (Figure S1) despite only binding with ∼10 μM affinity to the protein construct; this structure was obtained with the LpxC ortholog from *P. aeruginosa* (*Pa*LpxC), which was generally the most readily crystallized with the scaffold. A comparison to the crystal structure of **2** shows only a few relatively minor changes in the active site structure: 1) the side chain of Glu77 moves to accept a hydrogen bond from the pyrimidinone NH of **6** as opposed to the Zn^2+^-coordinating OH of the HA MBP (Figure 3A), (2) a displacement of Insert I to accommodate the larger MBP (Figure 3B), and (3) a displacement of Insert II to accommodate the larger diphenylacetylene backbone of **6**, including changes in the side chain conformations of Ile197 and Met194 (Figure 3C). Previous studies have demonstrated that Insert I of *Pa*LpxC is much more sensitive to disruption by inhibitors than LpxC from other species,^8,24^ which could explain the diminished activity of this scaffold against that ortholog.

**Figure 3:**
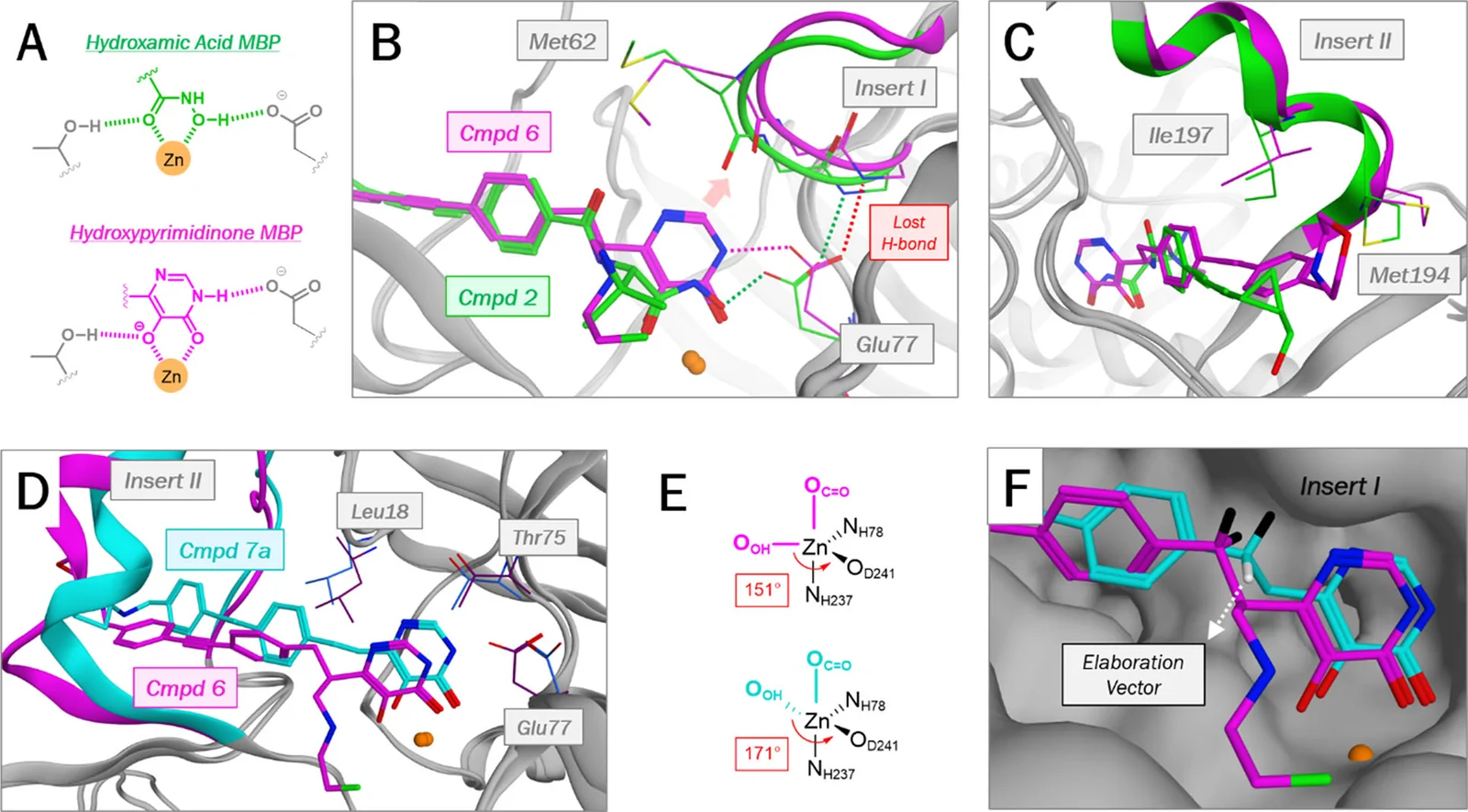
LpxC cocrystal structures of inhibitors based on the 5-hydroxypyrimidin-4-one MBP (magenta) compared to **2** (green, PDB 6MOO). The MBP of **6** adopts similar bidentate geometry to the Zn^2+^ ion of *Pa*LpxC but with a different arrangement of hydrogen bond donors and acceptors compared to HA (A). The larger MBP and change in hydrogen bonding to Glu77 causes a displacement in Insert I of the active site (B) and Insert II expands to accommodate the steric bulk of the diphenylacetylene backbone (C). The crystal structure of **7a** (cyan), which lacks a pendant arm, bound to *Ec*LpxC showed the inhibitor binding in the same pockets but in a modified pose (D) with a change from trigonal bipyramidal to square pyramidal coordination geometry (E). In this new pose, a new vector for elaboration from the linker is exposed (F, shown in white).

A subsequent crystal structure of **7a**, which lacks a pendant arm, bound to *Ec*LpxC showed that the scaffold maintains bidentate coordination to the active site Zn^2+^ ion, but in a slightly different geometry with significant impacts on both the second–sphere interactions formed by the MBP and the positioning of the rest of the inhibitor in the active site (Figure 3D). Electron density was well resolved for the backbone in all four *Ec*LpxC molecules in the asymmetric unit (Figure S2) showing a consistent pose (Figure S3). The coordination geometry adopted by **7a** is best described as square-pyramidal, compared to the distorted trigonal-bipyramidal geometry adopted by **6** (Figure 3E). Given that Zn^2+^ is a closed-shell ion (i.e., full d-electron count) and thus has little preference for specific geometries compared to other transition metals, this is unlikely to have a significant effect on the strength of metal coordination. The change in pose does not appear to break any of the polar contacts formed between the MBP and surrounding active site, but it does place the MBP ring closer to Thr76 (Thr75 in *Pa*LpxC), potentially forming more favorable nonpolar contacts. The first phenyl ring of the backbone is also positioned for more extensive nonpolar contacts with Leu18 (Leu19 in *Pa*LpxC) with insert II adopting a slightly more closed conformation. In addition to gaining insight into why compounds without a pendant arm might be more potent binders, this structure provided a path forward for refining the scaffold: only the first carbon of the linker proximal to the MBP (C1) was a viable vector for elaboration in the pose adopted by **6**, but in the new pose, one of the vectors extending from the second atom of the linker (C2) is oriented away from surrounding active site residues (Figure 3F). Although it was possible that this modified pose was specific only to *Ec*LpxC, scaffolds without a pendant arm or an artifact of crystallization, the result justified an initial chemistry campaign with compounds elaborated at that position (Figure 4).

**Figure 4:**
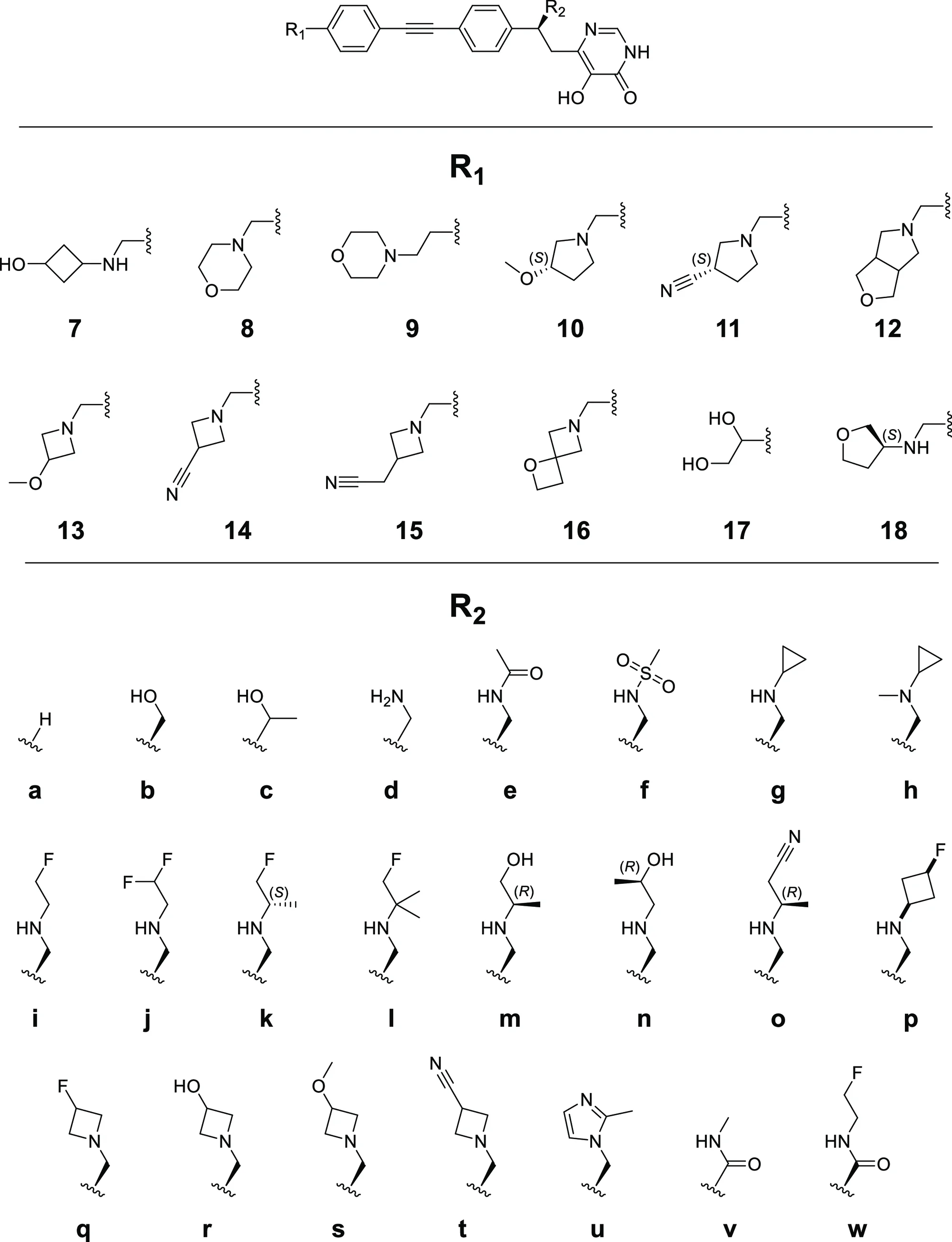
Structures and naming of inhibitors.

Compounds in this series were generally synthesized through the coupling of two key intermediates: the first contains the MBP, linker, and first backbone phenyl with a halogen to be appended via Sonogashira coupling to the second piece which contains the alkyne spacer, second backbone phenyl ring, and tail. The synthesis of the MBP began with 4,6-dichloro-5-methoxypyrimidine **19**, which was initially converted to its hydroxy analogue **20** prior to reprotection with a benzyl group to obtain **21**. This strategy enabled the use of a milder deprotecting agent in the final stages of compound preparation and improved the overall yield. The pyrimidinone core was completed by nucleophilic displacement of the chloro group with benzyl alcohol to yield bis­(benzyloxy) compound **22** (Scheme 1). For the synthesis of C1-pendant scaffolds, including **6**, nucleophilic aromatic substitution of the remaining chlorine was carried out with diethyl malonate in the presence of potassium *tert*-butoxide. Under these conditions, the product underwent a selective decarboxylation of one ester group to afford intermediate **23**, which could be alkylated with 4-iodobenzyl bromide to yield **24**. Following reduction using LiBH_4_ to generate hydroxy compound **25**, azide **26** was generated through a mesylated intermediate. This azide was then converted to a primary amine using the Staudinger reaction and subsequently alkylated with 1-bromo-2-fluoroethane, yielding key intermediate **27**. The second key intermediate, **30**, was prepared from 4-ethynylbenzaldehyde and morpholine via reductive amination (Scheme 2). Coupling to **28** using standard Sonogashira conditions afforded **31**, which was deprotected using TFA to afford desired product **6**.

**Scheme 1:**
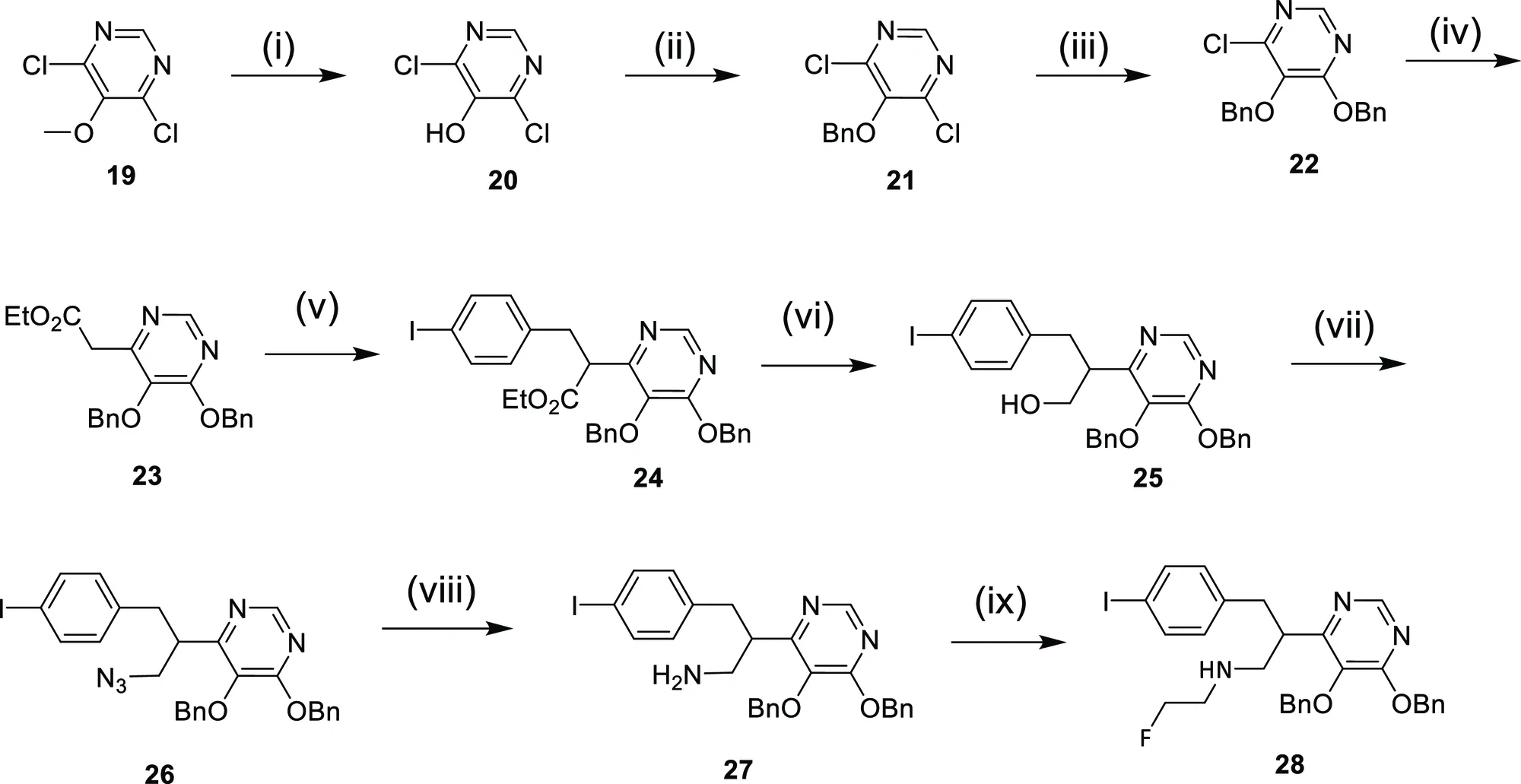
Synthesis of the Portion of the Scaffold Containing MBP, Linker/Pendant, and First Phenyl Ring of Compound **6**. Reagents and Conditions: (i) AlCl_3_, EDC, DCM (ii) BnBr, K_2_CO_3_, DMF (iii) BnOH, NaH, THF (iv) Diethyl Malonate, K^t^BuO, Toluene (v) NaH, THF, 4-Iodobenzyl Bromide (vi) LiBH_4_, EtOH/Diethyl Ether (vii) MsCl, NaN_3_, DMF (viii) PPh_3_, H_2_O/THF (ix) 1-Bromo-2-fluoroethane, K_2_CO_3_, DMF

**Scheme 2:**
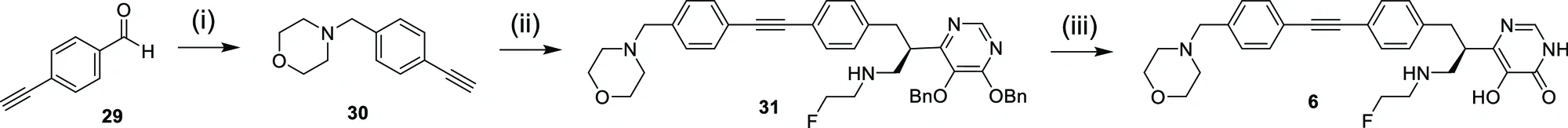
Synthesis of **6**. Reagents and Conditions: (i) Morpholine, AcOH, NaBH_3_CN (ii) **27**, PdCl_2_(PPh_3_)_2_/CuI, Et_3_N, Dioxane, Supercritical Fluid Chromatography (SFC) Separation (iii) TFA

For the C2-pendant series, intermediate **20** was converted to alkene **32** using Stille coupling followed by ozonolysis and reduction to obtain alcohol **33**. Conversion to a mesylate followed by nucleophilic displacement with NaI afforded iodomethyl analogue **34**. Substitution with ethyl 2-(4-iodophenyl)­acetate then yielded **35** in a reaction analogous to that used to generate **24** (Scheme 3). The ester **35** was reduced in the presence of DIBAL-H to afford the alcohol **37**, which was then either substituted to yield the corresponding primary amine **38** or oxidized to the corresponding aldehyde **39**, which allowed a variety of amines (intermediates **40d**–**u**) to be generated. Analogues lacking a pendant arm could be synthesized by hydrolysis of the ester to yield carboxylic acid **36** followed by decarboxylation to yield intermediate **40a**. Amide analogues were synthesized via amide coupling of **36** with amines to afford the intermediates **40v** and **40w**. These amide intermediates were used for the synthesis of a variety of inhibitors, but for the synthesis of **8v** and **8w**, this reaction was performed after the diphenylacetylene backbone had been installed (see the Supporting Information for details). All of the key intermediates underwent final product formation using the same method as reported in Scheme I for the final coupling and deprotection. To avoid resource-intensive chiral separation, compounds were initially synthesized as racemates at the pendant and screened for in vitro activity before enantiomerically pure materials were prepared by chiral SFC for follow-up testing. Absolute stereochemistry was not determined at this point, but the pose of **7a** bound to *Ec*LpxC suggested that the more active fraction would be the (*S*) enantiomer. Later in the program, a chiral auxiliary was used to selectively generate intermediates with (*S*) stereochemistry, and in every case where the chiral chromatograms of intermediates or final products were compared to the initial batches isolated with unknown stereochemistry, the more active fraction corresponded to the (*S*) enantiomer. Chemistry, manufacturing, and control considerations during preclinical development, including this enantioselective synthesis, will be the subject of a subsequent publication. Although the (*R*) enantiomers occasionally maintained activity against *E. coli* and *K. pneumoniae*, the loss in activity against *P. mirabilis* tended to be more universal (Table S1).

**Scheme 3:**
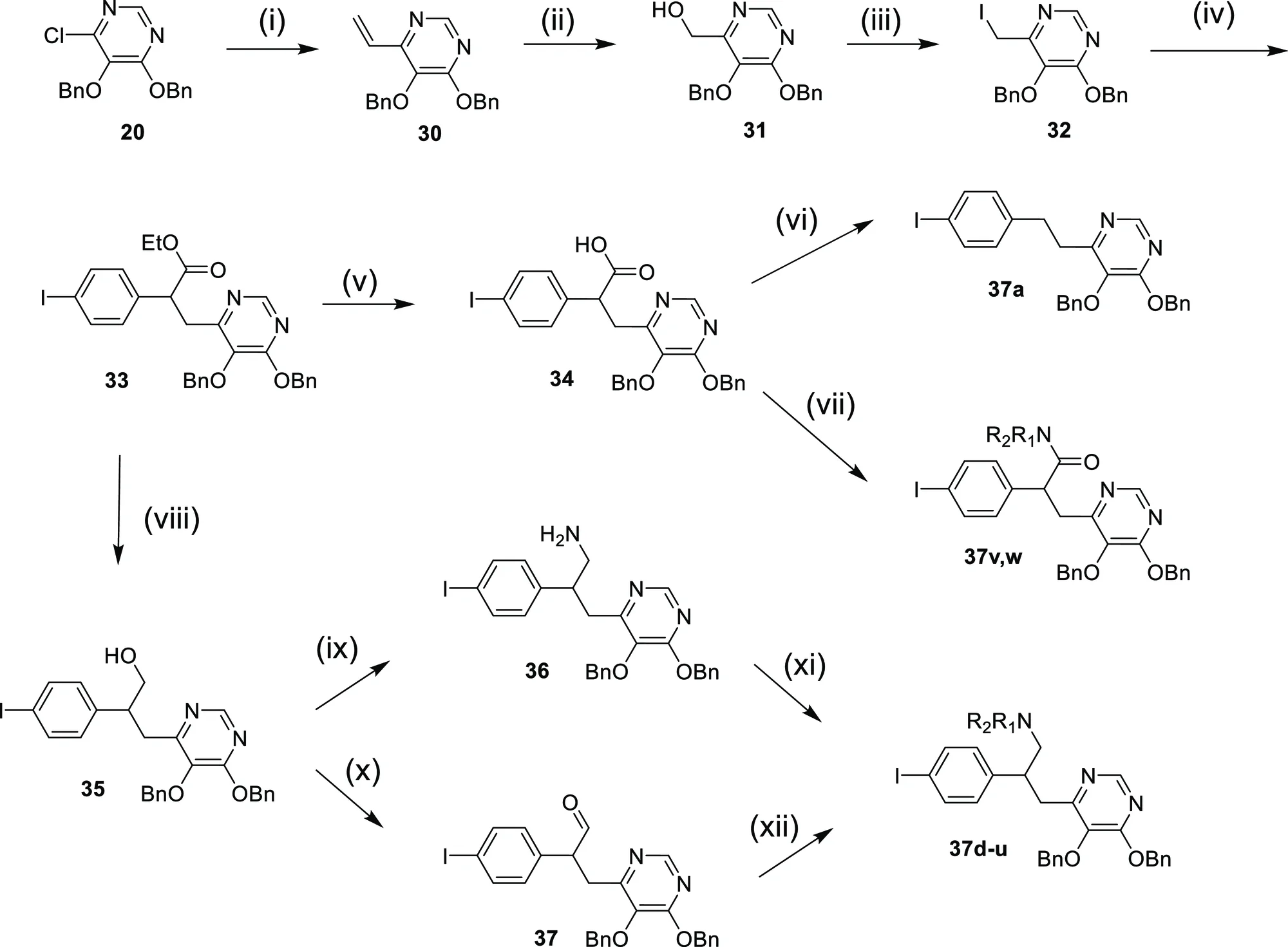
Synthesis of the MBP-Containing Portion of C2-Pendant Inhibitors; Reagents and Conditions: (i) Tributyl­(vinyl)­tin, Pd­(PPh_3_)_4,_ DMF (ii) (1) Ozone, Dimethyl Sulfide (2) NaBH_4_, DCM/MeOH (iii) (1) MsCl, DCM (2) NaI (iv) NaH, THF, Ethyl 2-(4-iodophenyl)­acetate (v) NaOH, THF/MeOH/H_2_O (vi) Heat (vii) EDC, R_1_R_2_NH/HOBT, DCM (viii) LiBH_4_, THF/EtOH (ix) (1) Phthalimide, DEAD, PPh_3_, THF (2) N_2_H_4_, EtOH (x) Dess-Martin Periodinane, (xi) R_1_Br, K_2_CO_3_, CuI, Dioxane (xii) 2-Picoline Borane, R_2_R_1_NH, AcOH, MeOH

Moving the pendant arm from C1 to C2 provided the vital breakthrough in generating a scaffold with improved potency and more favorable drug-like properties (Figure 5). The resulting analogs bind LpxC more tightly (as judged by a differential scanning fluorimetry-based assay and confirmed by Rapid-Fire MS-based enzymatic assay), which led almost universally to improved antimicrobial activity, although the difference was more muted against *P. aeruginosa*. Incorporating the pendant groups closer to the lipophilic backbone also enhanced the physicochemical properties of analogs pendant arms, which is reflected in lower plasma protein binding (mPPB) and higher solubility. Based on the relatively small decrease in logD predicted by simple connectivity-based computational methods (∼0.2 units), it is possible that intramolecular hydrogen bonding between the pendant arm and the hydroxyl group of the MBP is masking the polarity of C1 analogs.

**Figure 5:**
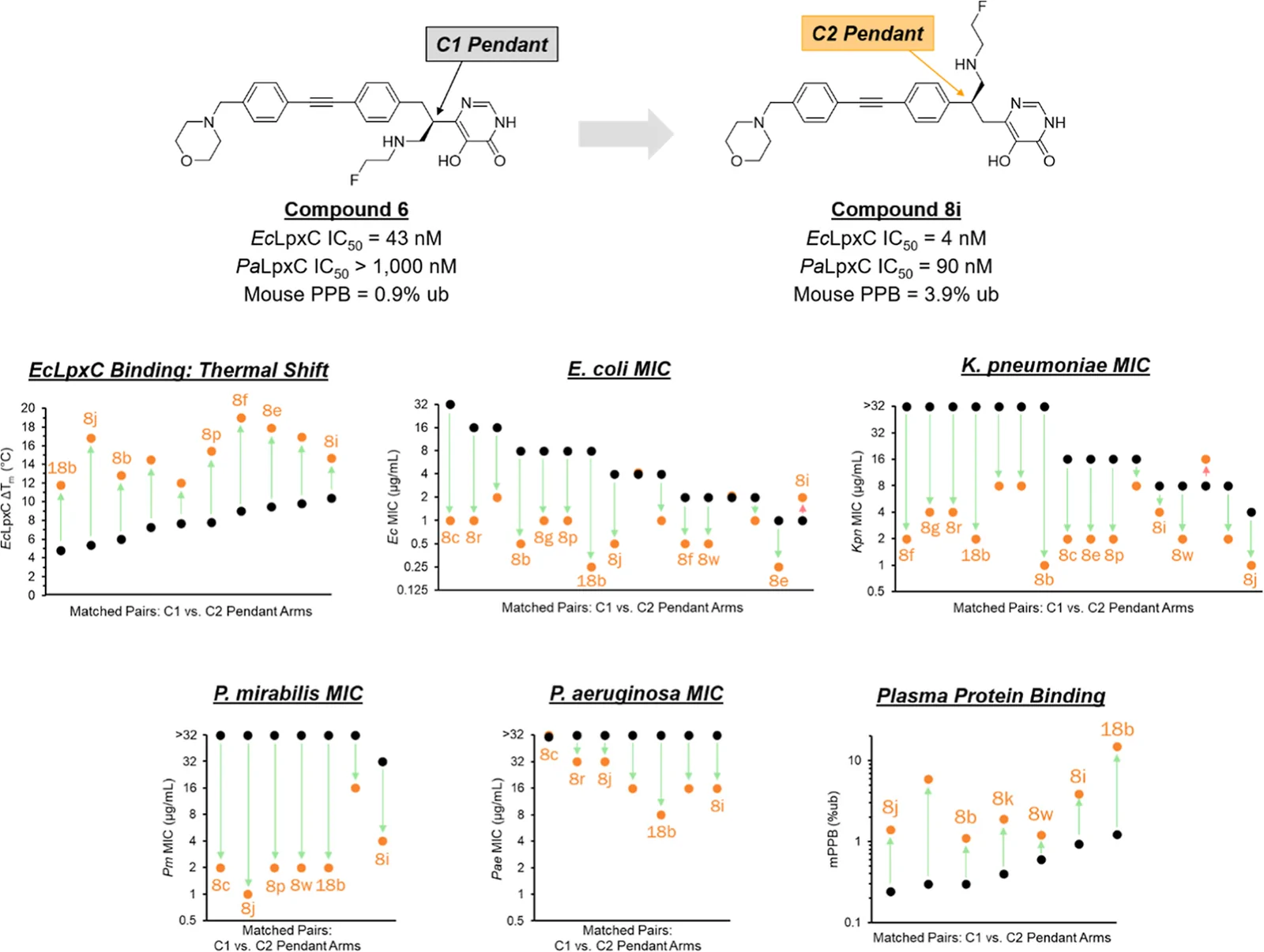
Impact of moving the pendant arm of LpxC inhibitors on their properties and antimicrobial activity. Moving the pendant arm from C1 (black circles) to C2 (orange circles) universally improves LpxC binding, antibacterial activity, and physicochemical properties.

The crystal structures of two compounds with C2 pendant arms bound to *Pa*LpxC show that the pose of **7a** discussed above is maintained by compounds in this family with no significant changes in the overall active site structure (Figure 6A). In the *Pa*LpxC:**8k** complex, only the MBP and backbone are well-resolved, likely due to disordered binding of the pendant arm and tail (Figure S4). There is also significant disorder in Insert I, with the peptide backbone refining with *B*-values >2× the rest of the protein. Insert II is more well-resolved but still shows side chain *B*-values ∼2× the average for the entire protein. In the *Pa*LpxC:**8t** complex, both the inhibitor and Inserts are more well-ordered. The cyanoazetidine pendant arm of **8t** can be resolved in the electron density (Figure S5) and is positioned to form several nonpolar contacts with Insert II. This is accompanied by a decrease in B-values for the side chains in this region, which are close to the average for the rest of the protein. Given that **8t** is one of the most potent inhibitors in this family against *Pa*LpxC (Table 1), it is possible that these interactions help bridge Inserts I and II, counterbalancing the energetic penalties associated with the displacement of Insert I by the pyrimidinone-based MBP. Another unique feature of the *Pa*LpxC:**8t** complex is the presence of a water molecule forming bridging hydrogen bonds between His19 and the pyrimidinone ring of the MBP (Figure 6B). Although these subtle changes can help explain the observed SAR, it is important to note that crystal packing interactions differ between all the structures discussed here (Figure S6). While it is certainly possible that these variations in crystal packing reflect changes in the lowest-energy active site conformation and are thus reasonable representations of the behavior of the complex in solution, it is also possible that they are artifacts of crystallization. Solution-based methods could potentially resolve this question but were outside the scope of this program.

**Figure 6:**
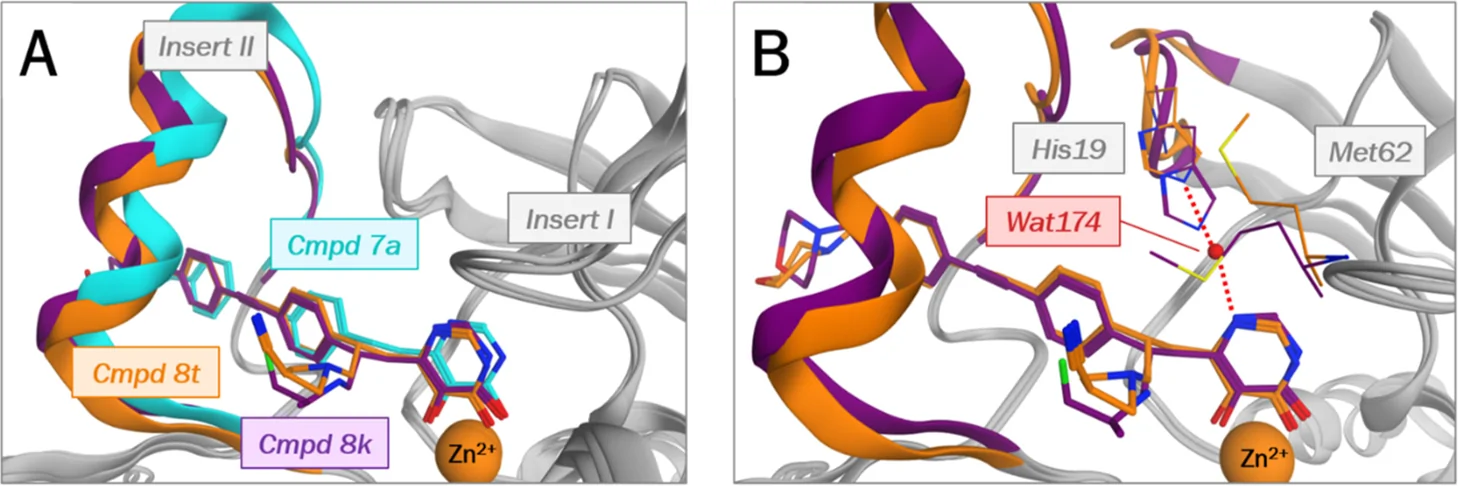
Crystal structures of **8k** (purple) and **8t** (orange) bound to *Pa*LpxC. (A): the scaffold adopts the same binding mode as in the **7a**:*Ec*LpxC complex (cyan). Insert I of *Ec*LpxC is structurally distinct and omitted for clarity. (B): the active site structure is relatively unchanged between the two complexes aside slight changes in Insert I and a bridging water molecule (red sphere) between the MBP of **8t** and His19.

**Table 1:** Antimicrobial Activity and LpxC Inhibition for Compounds with Variable Pendant Groups

	MIC (μg/mL)				LpxC IC_50_ (nM)			MIC (μg/mL)				LpxC IC_50_ (nM)	
cmpd	Ec	Kp	Pm	Pa	Ec	Pa	cmpd	Ec	Kp	Pm	Pa	Ec	Pa
**6**	1	8	32	>32	43	>1000	**8l**	0.25	0.5	4	8		
**8a**	0.25	0.5	0.25	>32	1	>1000	**8m**	0.5	4	8	4		
**8b**	0.5	1	4	8			**8n**	1	4	16	8		
**8c** ^a^	1	2	>32	>32			**8o**	0.25	1	2	8		
**8d** ^a^	32	>32	>32	>32			**8p**	1	2	8	16	8	70
**8e**	0.25	2	8	8			**8q**	0.13	0.5	0.5	8	2	49
**8f**	0.5	2	4	8			**8r**	1	4	8	2	1	26
**8g**	1	4	8	16	3	58	**8s**	0.5	1	8	8	6	110
**8h**	0.25	1	4	8			**8t**	0.25	0.5	2	8	1	21
**8i**	2	4	8	16	4	90	**8u**	0.25	0.5	4	4		
**8j**	0.5	1	4	32	2	25	**8v** ^a^	1	8	>32	>32		
**8k**	0.13	0.5	4	8			**8w**	0.5	2	4	16	3	140

a Compound was only tested as a racemate; Ec = *E. coli* ATCC25922; Kp = *K. pneumoniae* ATCC13883; Pm = *P. mirabilis* ATCC21100; Pa = *P. aeruginosa* PAO1.

Antimicrobial activity of compounds with C2 pendant arms are summarized in Tables 1 and 2. Compounds without a pendant arm (e.g., **8a**) tend to be very active against *Enterobacteriaceae* but failed to demonstrate any significant activity against *P. aeruginosa* in vitro due to a lack of target affinity (IC_50_ > 1 μM against *Pa*LpxC). Compounds with unsubstituted linkers also tend to be highly lipophilic and susceptible to rapid clearance in vitro (Table 3), making them unsuitable for efficacy screening. This is consistent with observations during the early lead identification efforts that led to **6**: due to the lipophilicity of the diphenylacetylene backbone, a significant amount of polarity must be incorporated on both the pendant arm and the tail for the resulting molecule to have favorable drug-like properties.

**Table 2:** Antimicrobial Activity and LpxC Inhibition for Compounds with Variable Tail Groups

	MIC (μg/mL)				LpxC IC_50_ (nM)			MIC (μg/mL)				LpxC IC_50_ (nM)	
cmpd	Ec	Kp	Pm	Pa	Ec	Pa	cmpd	Ec	Kp	Pm	Pa	Ec	Pa
**9k** ^a^	0.25	1	8	16			**13s**	0.5	4	32	8		
**10k**	1	4	8	8	32	280	**13t**	2	8	8	8		
**11k**	1	1	4	16			**14k**	0.25	1	4	16		
**11t**	0.5	2	16	32			**15k** ^a^	1	4	32	32		
**12k**	0.25	1	4	8			**16k**	1	4	8	16		
**13b**	0.25	1	2	8			**17b**	2	4	4	4		
**13g**	0.25	2	8	16			**17k**	1	4	2	8		
**13h**	0.13	0.25	2	4			**18b**	0.25	2	16	8	14	570
**13k**	0.25	2	8	8	17	150	**18k**	0.5	4	16	16		
**13p**	2	1	4	4									

a The compound was only tested as a racemate at the pendant; Ec = *E. coli* ATCC25922; Kp = *K. pneumoniae* ATCC13883; Pm = *P. mirabilis* ATCC21100; Pa = *P. aeruginosa* PAO1.

**Table 3:** In Vitro ADME and In Vivo PK Parameters for Selected Compounds

	in vitro ADME						
			Caco-2 (cm/s*10^–6^)		in vivo pk		
cmpd	mPPB (% ub)	mHep CL_int_ (mL/min/10^6^ cells)	A-B	B-A	CL (mL/min/kg)	*V*_dss_ (L/kg)	%F
**8a**	0.2	>500	ND		ND		
**8b**	1.1	45	5.2	5.0	60	5.5	40
**8g**	2.2	150	3.6	7.0	82	9.3	55
**8j**	1.4	25	5.6	1.0	140	4.9	88
**8k**	1.6	58	1.6	4.8	60	2.0	72
**8o**	2.0	68	0.4	5.2	ND		
**8p**	4.4	120	2.7	8.4	94	4.5	60
**8q**	2.0	41	1.7	4.6	ND		
**8s**	4.8	65	ND		ND		
**8t**	1.8	19	0.5	17	51	5.1	59
**8w**	0.6	7.4	ND		13	1.0	14
**9k**	6.2	56	1.7	4.7	99	13	44
**13k**	6.1	94	0.8	2.7	70	13	50
**18b**	8.1	24	0.8	6.8	130	19	15
**18k**	8.3	53	0.4	5.1	72	6.5	13

Between the tail and pendant arm positions, the latter proved to be more accommodative to functional diversity to modulate the properties of these inhibitors (Table 1). Appending a simple hydroxymethyl group (e.g., **8b**) resulted in compounds with good in vitro potency and reasonable drug-like properties. Introducing steric bulk onto the pendant arm at the first atom proximal to the linker (e.g., **8c**) generally led to a loss of antimicrobial activity against *P. mirabilis*, and *P. aeruginosa*. Compounds with amide-based pendants (e.g., **8w**) maintained the potency but tended to be highly lipophilic. Attempts to incorporate polarity in the phenyl regions of the molecule, e.g., substitution for a pyridyl ring, systematically led to significant loss of potency (Table S2). Given the need to decrease the lipophilicity of the scaffold, amine-based pendants were logical choices. The aminomethyl pendant of **8d** led to a drastic loss in activity due to a loss of target binding affinity, but elaboration into secondary or tertiary amines, amides, or other functional groups yielded a variety of potent inhibitors (**8e**–**u**) with a range of physicochemical characteristics and in vitro ADME profiles (Table 3). Pendants **k**, **m**, **n**, and **o** were constructed from starting materials with known absolute stereochemistry; these stereocenters tended to have little impact on inhibitor properties (Table S3), so only one enantiomer is shown for brevity.

The results of tail optimization largely mirrored those of the effort that led to **4**: tertiary amines tethered to the backbone by a methylene spacer were uniquely able to introduce polarity while maintaining broad-spectrum antimicrobial activity (Table 2). Some secondary amines (e.g., **18k**) and inhibitors with longer tethers to the amine (e.g., **9k**) also demonstrated promising activity, particularly against *E. coli* and *K. pneumoniae*, but they tended to underperform against *P. mirabilis* in vitro (Table 1) and were rapidly cleared in vivo (Table 3); these compounds did not show efficacy in early screening models using susceptible strains (Figure S7). More extensive substitution at the first carbon proximal to the backbone was occasionally tolerated (e.g., **17b** and **17k**; Table 1), but these were outliers, and this subset generally had weaker target binding, so efforts were focused elsewhere.

Consistent with their potency in vitro and reasonable ADME properties, most of the compounds in the series were efficacious against *E. coli* and *K. pneumoniae* in preliminary in vivo models; to narrow down to the most promising lead candidates, a more stringent evaluation was needed. An assessment of in vivo efficacy in models of *P. mirabilis* infection, against which the scaffold tends to have weaker in vitro activity, was pursued to ensure efficacy against more-resistant strains and coverage of a wider range of *Enterobacteriaceae*. To this end, a 24 h murine thigh infection model was chosen to screen for compounds likely to have favorable tissue distribution. Even with *P. mirabilis* representing a higher hurdle to efficacy, most compounds tested showed at least some degree of reduction in infectious bioburden (Table 4). To account for study-to-study variation, reductions in bioburden are presented relative to treatment with tigecycline, which served as the positive control in each study. Scatter plots for the individual studies can be found in Figure S8. Compound **13k**, which kept the infections near stasis with the dosing regimen evaluated (30 mg/kg every 4 h), was also included as an internal control in each of the studies that were used to generate the data in Table 3. Frequent dosing was used since many compounds displayed relatively high clearance in PK studies, and the dose level was chosen based on our experience of what distinguishes the most and least potent compounds. Among amine-based pendants, only **8p** (which showed a high propensity to hepatic clearance in vitro) was unable to significantly reduce bioburdens relative to vehicle. Several inhibitors showed an efficacy output equivalent to that demonstrated by **2** (ACHN-975).

**Table 4:** Efficacy against *P. mirabilis* in a Murine Thigh Infection Model^a^

**cmpd**	**MIC** (μg/mL)	efficacy^b^ (ΔCFU/g, log_10_)	**cmpd**	**MIC** (μg/mL)	efficacy (ΔCFU/g, log_10_)	**cmpd**	**MIC** (μg/mL)	efficacy (ΔCFU/g, log_10_)
**2**	NT	–1.16	**8r**	8	0.95	**13k**	8	1.71 ± 0.55^c^
**8b**	4	2.16	**8s**	8	–0.81	**13p**	4	0.49
**8g**	8	–1.08	**8t**	2	0.61	**13t**	8	1.05
**8j**	4	1.29	**10k**	8	1.62	**14k**	4	–0.99
**8k**	4	–0.84	**11t**	16	2.8	**17k**	8	0.24
**8m**	8	–0.02	**12k**	8	0.22	**18b**	16	2.44
**8n**	16	–0.10	**13b**	2	2.56	**18k**	16	0.28
**8p**	8	5.87	**13g**	8	–0.73	**Tig**	NT	0 ± 1.31
**8q**	0.5	1.52	**13h**	2	1.14			

a Bioburdens are given relative to tigecycline positive control, with negative values indicating more efficacious compounds.

b Change in bioburden relative to tigecycline (Tig) treatment.

c Compound **11k** was included as in all four studies as an internal control.

Given the discontinuation of ACHN-975 due to safety findings in phase I, evaluating the safety of this scaffold with respect to the potential for similar CV toxicity was critical to the program. Through an agreement facilitated by CARB-X, access to data and procedures from Achaogen (most importantly, validated in vivo models of CV toxicity) was provided to Blacksmith, allowing for head-to-head comparison.^25^ In a telemeterized rat CV toxicity model, compounds were dosed via 30 min IV infusion to anesthetized animals while monitoring a variety of hemodynamic parameters including mean arterial pressure and d*P*/d*T*_max_ as a measure of heart contractility. The dose (37.5 mg/kg) was chosen on the basis of where significant effects were reported for ACHN-975. As was found previously with the ACHN-975 scaffold, the results of this study suggest that basic amines (i.e., those with a substantial fraction protonated at physiological pH) drive the hemodynamic effects of the inhibitor scaffold (Table 5).^16^ Interestingly, the location of the amine within the inhibitor also played a significant role; the basicity of tail amines was found to be far more important than that of the pendant arm. Compounds **8r** and **8s**, which contain pendant amines predicted to have p*K*_a_ values similar to those of ACHN-975, showed no hemodynamic effects. It should be noted that the purpose of these experiments was to derisk the scaffold with respect to these effects rather than quantify a therapeutic index; more thorough toxicology studies will be discussed in subsequent publications. As an additional derisking step, selected compounds were screened against contemporary MDR isolates and were shown to have reasonable activity (Table S4).

**Table 5:** Effect of LpxC Inhibitors on Heart Contractility in a Rat CV Toxicity Model Compared to ACHN-975 (**2**)­^a^

cmpd	p*K*_a_ (tail)	p*K*_a_ (pendant)	% reduction in d*P*/d*t*_max_
**2**	-	8.3	**33**
**8j**	6.4	6.8	5
**8t**	6.4	7.4	0
**8q**	6.4	7.5	10
**8s**	6.4	8.2	0
**8r**	6.4	8.4	0
**8k**	6.4	8.7	15
**8g**	6.4	9.2	16
**8m**	6.4	9.5	**35**
**14k**	6.5	8.7	19
**13b**	7.4	-	**31**
**13k**	7.4	8.7	**45**
**16k**	7.5	8.7	**55**
**18b**	8.5	-	**35**

a Compounds were dosed at 37.5 mg/kg by 30 minute IV infusion. Compounds with a significant (>20%) reduction in d*P*/d*t*_max_ are shown in bold.

## Conclusions

Structure-based optimization of a novel nonhydroxamate MBP scaffold has led to the discovery of a variety of LpxC inhibitors with in vitro activity against MDR strains, favorable ADME properties, and in vivo efficacy against a range of *Enterobacteriaceae*. To assess the risk with respect to the safety issues that prevented a previous LpxC program from advancing past Phase I trials, an in vivo model of CV toxicity was utilized. As was the case with previous scaffolds, the key driver of hemodynamic effects was determined to be the presence of a basic amine. Several inhibitors were identified that were highly efficacious in vivo and had no observed effects on the CV functionality. Selection of an initial clinical application for the scaffold, advanced in vivo and microbiological characterization of the lead compounds, and selection of a clinical candidate will be the subject of a forthcoming publication.

## Experimental Section

### Synthesis

Compounds were synthesized as shown in Schemes 1, 2, and 3 and were >95% pure as determined by LC–MS. General information, as well as procedures and characterization for the synthesis of all intermediates and final compounds, can be found in the Supporting Information.

### Protein Expression and Purification

The synthetic gene of the recombinant *Ec*LpxC M1-P300 (Uniprot D5CV28) without tag was cloned in pET21b vector (Genscript). *Ec*LpxC was expressed by 1 mM IPTG in 2YT media (Teknova) in BL21 (DE3) pLysS over 2 h at 25 °C. To purify *Ec*LpxC, the cell pellet was resuspended in lysis buffer containing 20 mM phosphate pH 7.0, 20% glycerol, 20 mM DTT, 10 U/mL benzonase, and complete protease inhibitor tablets (Roche). The resuspended cells were lysed with a microfluidizer at 25,000 psi and clarified by centrifugation. Ammonium sulfate was added slowly to the lysate to 0.3 g/mL and stirred at 4 °C for 60 min. After centrifugation, the pellet was solubilized in 25 mM imidazole pH 7.0, 20% v/v glycerol, 2 mM DTT. The protein was further purified by ion exchange chromatography using a HiTrap Q FF column (Cytiva) and eluted with 25 mM imidazole pH 7.0, 20% glycerol, 2 mM DTT, 350 mM KCl. Finally, size exclusion chromatography was applied to the *Ec*LpxC sample using a Superdex 75 (Cytiva) column equilibrated in 25 mM HEPES pH 7.0, 100 mM KCl, 2 mM DTT, and 0.1 mM ZnSO_4_ before concentrating to 10 mg/mL.

Recombinant *Pa*LpxC M1-P299 C40S (Uniprot P47205) with His-SUMO-tag fused to its *N*-terminus was cloned in pBAD vector (Genscript). *Pa*LpxC was expressed by 0.02% arabinose in LB media (Teknova) in BL21 (DE3) over 18 h at 25 °C. To purify *Pa*LpxC, the cell pellet was resuspended in lysis buffer containing 25 mM HEPES pH 7.5, 250 mM NaCl, 10% glycerol, 2 mM TCEP, 10 U/mL benzonase, and complete protease inhibitor tablets (Roche). The resuspended cells were lysed with a microfluidizer at 25,000 psi and clarified by centrifugation. For IMAC, the protein was bound to a HisTrapFF (Cytiva) loaded with 0.1 M ZnSO_4_ and eluted with 25 mM HEPES pH 7.5, 250 mM NaCl, 10% glycerol, 2 mM TCEP and 500 mM imidazole. The tagged protein was treated with SUMO protease (with a ratio of 1 mg SUMO protease per 15 mg *Pa*LpxC) overnight at 4 °C. The untagged protein was further purified by a by ion exchange chromatography using a HiTrap Q FF column (Cytiva) and eluted with 25 mM HEPES pH 7.5, 500 mM NaCl, 5% glycerol, 2 mM TCEP, 0.1 mM ZnSO_4_ followed by size-exclusion chromatography using a Superdex 75 (Cytiva) with 20 mM Tris–HCl pH 7.0, 150 mM NaCl, 2 mM TCEP before concentrating to 5 mg/mL.

### Cocrystallization

The *Pa*LpxC complexes with compounds **6** and **8k** were formed by preincubating inhibitor (2 mM and 1 mM final concentration, respectively) and enzyme (5 and 17 mg/mL, respectively) at 4 °C. Crystals were grown by vapor diffusion at room temperature from drops containing equal volumes of protein solution with precipitant (**6**, 100 mM bis-tris propane pH 6.5, 200 mM sodium citrate tribasic dihydrate, 20% w/v PEG 3350; **8k**, 100 mM bis-tris propane pH 8.5, 0.2 M ammonium nitrate, 18% v/v PEG Smear High [Molecular Dimensions] respectively). The *Ec*LpxC complex with **7a** was formed by preincubating inhibitor (2 mM final concentration) and enzyme (10 mg/mL) at 4 °C. Crystals were grown by vapor diffusion at 4 °C from drops containing equal volumes of protein solution and precipitant (100 mM phosphate/citrate pH 4.2, 100 mM LiSO_4_, 200 mM sodium citrate tribasic dihydrate, 20% w/v PEG 1000). The *Pa*LpxC complex with **8t** was formed by incubating inhibitor (1 mM final concentration) with enzyme (∼20 mg/mL in 25 mM HEPES pH 7.5, 150 mM NaCl, 2 mM TCEP, 25 μM ZnCl_2_, 1% ethylene glycol) at room temperature. Crystals were grown by vapor diffusion at room temperature drops containing a 2:1 mixture of protein solution and precipitant (100 mM Tris pH 7.4, 32% PEG Smear Medium [Molecular Dimensions], 2% glycerol). Crystals were harvested and flash-cooled in liquid nitrogen for diffraction data collection.

### Diffraction Data Collection, Processing, and Model Building

Data for the *Pa*Lpx:**6**, *Ec*LpxC:**7a**, and *Pa*LpxC:**8k** complexes were collected at Diamond Light Source (Oxford, U.K.). Each complete data set was collected from an individual crystal under a cryogenic stream at 100 K and processed using the automated pipeline autoPROC^26^ that executes XDS,^27^ POINTLESS,^28^ and AIMLESS^29^ of the CCP4 suite,^30^ as well as the STARANISO module.^31^ The structures were solved by the molecular replacement method, using Phaser^32^ with PDB entries 3P3G (*Ec*LpxC) and 4J3D (*Pa*LpxC) as the starting models. These models were then refined using *BUSTER* ^33^ and manually corrected using *COOT*.^34^ Compound dictionaries were generated using *GRADE2*.^35^

Data for the *Pa*LpxC:**8t** complex were collected at the crystallography facility at the University of California, San Diego using Rigaku MicroMax-007 HF rotating anode X-ray source (λ = 1.5406 Å). Data were integrated and scaled in the Bruker APEX3 software suite followed by phasing and model building in the CCP4 software suite.^30^ Initial phasing was performed by molecular replacement using Phaser with the PDB entry 4J3D (*Pa*LpxC) structure as a search model. The model was further refined by alternating manual inspection in Coot^34^ with bulk refinement using Refmac5.^36^ Data collection and refinement statistics can be found in Table S5.

### Differential Scanning Fluorimetry

*Ec*LpxC (0.2 mg/mL final concentration) and inhibitor (20 μM final concentration) were combined in buffer (50 mM HEPES pH 7.5, 100 mM NaCl) along with SYPRO Orange (final concentration of 10x). Experiments were carried out in 20 μL triplicates using a QuantStudio 3 RT-PCR system ramping from 25 to 95 °C at a rate of 0.1 °C/s. Melting points (*T*_m_) were defined as the temperature at which d*F*/d*t* reached a maximum and compared to inhibitor-free wells to determine Δ*T*_m_.

### RF/MS-Based LpxC Activity Assays

Reactions were carried out in a final volume of 30 μL with 25 mM HEPES (pH 7.3), 150 mM NaCl, 2 mM DTT, 0.01% BSA, 2 μM UDP-3-O­[*R*-3-hydroxymyristoyl]-*N*-acetylglucosamine, 0.5 nM *Ec*LpxC or 10 nM *Pa*LpxC and 1% DMSO. After 15 min of incubation at RT, reactions were stopped by addition of 30 μL of 10% TCA in water. Samples were centrifuged (4350 rpm, 4 °C, 5 min) and subjected to RF/MS analysis on an Agilent RapidFire 300 coupled to an Agilent 6460 QQQ mass spectrometer. Analytes were retained on a C4 (type A) cartridge using the following solvents and flow rates: pump 1 −H_2_O with 5 mM NH_4_OAc at 2.0 mL/min, pump 2 −MeOH/H_2_O (90/10) at 1.5 mL/min, pump 3 −MeOH/H_2_O (90/10) at 0.5 mL/min and pump 4 −ACN (organic)/H_2_O (aqueous). Substrate and product of the reaction were detected in negative mode ESI-MS/MS by multiple reaction monitoring of transitions 832.2/384.9 (UDP-3-O­[*R*-3-Hydroxymyristoyl]-*N*-acetylglucosamine substrate) and 790.4/385.0 (UDP-3-*O*-(*R*-3-hydroxymyristoyl)-glucosamine product).

### Antimicrobial Susceptibility Testing

Bacterial MICs were determined according to CLSI standards.^37^ Briefly, inhibitors were suspended to 6.4 mg/mL in DMSO. Inhibitors are serially diluted 1:2 across 11 wells to make 50× concentration of serial dilution range. Bacteria are suspended in 2 mL of PBS to OD_600 nm_ ≈ 0.1. Bacteria are then diluted 1:200 in CA-MHB to make a stock that is between 105 and 106 CFU/mL. To each well of a 96 well plate, 98 μL was added of the diluted bacteria. Serially diluted inhibitor (2 μL) was then added to appropriate rows. Plates were gently shaken for ∼4 min to mix on a plate shaker and then incubated for 16–20 h at 37 °C before visually recording bacterial growth.

### Murine Thigh Infection Model

All efficacy experiments were performed at Evotec under UK Home Office License PPL PA67E0BAA, and with local ethical clearance by Evotec’s onsite independent Animal Welfare Review Body. All experiments were performed in a dedicated Containment Level 2 facility by trained scientists that had completed parts A, B and C of the UK Home Office Personal License course and held a current personal license. Male CD1 mice (*n* = 5) were rendered neutropenic by intraperitoneal injections of cyclophosphamide on Day −4 (150 mg/kg) and on Day −1 (100 mg/kg). One hour prior to treatment, 50 μL of *P. mirabilis* B861 inoculum (∼10^7^ CFU/mL) was administered intramuscularly into each hind limb thigh. One hour later, compounds were administered by IV bolus injection at 30 mg/kg with repeated dosing every 4 h. One group was culled prior to the first dose as a pretreatment control while all remaining animals were euthanized 4 h after the final dose (24 h after the first dose), at which point the thighs were homogenized and remaining bacterial bioburdens determined by plating and viable counting. Tigecycline (20 mg/kg IV q4h) was included as a comparator. MICs against *P. mirabilis* B861 were only determined for a small subset of compounds but were generally within 2-fold of the values for ATCC21100, which are given in Tables 1, 2 and 4.

### Rat cardiovascular Toxicity Model

Experimental procedures were carried out at the University of Illinois at Chicago (an AAALAC-accredited institution) in compliance with all applicable laws and regulations, including the Animal Welfare Act and its regulations, the PHS Policy on Humane Care and Use of Laboratory Animals, and the National Research Council’s most recent edition of the Guide for the Care and Use of Laboratory Animals. All experiments were approved by the Institutional Animal Care and Use Committee of the University of Illinois at Chicago (Assurance ID A8668-07). Male Sprague–Dawley rats (0.239–0.291 kg, *n* = 3) were anesthetized with isoflurane, and an endotracheal tube was inserted and connected to a ventilator that was set for positive-pressure ventilation based on the animal’s body weight. The ventilator was also connected to a vaporizer that delivered approximately 1–2% isoflurane driven by 100% oxygen. Rats were placed in the dorsal decubitus position on a warming platform to maintain body temperature at approximately 37 °C. A femoral vein was cannulated for infusion of test material while a Millar pressure catheter was introduced into a femoral artery to measure systolic arterial pressure and diastolic pressure. Another Millar pressure catheter was introduced into the right carotid artery to measure left ventricular pressure. External electrocardiographic leads were placed in a lead II configuration. After equilibration, compounds were administered at 37.5 mg/kg by 30 min IV infusion. All animals were monitored from equilibration until 60 min after the end of the infusion. Animals were euthanized under anesthesia upon completion of data collection.

## Supplementary Material





## Notes

Molecular formula strings and selected data (CSV)
Full synthetic procedures, crystal structure data collection and refinement statistics, and inhibitor electron density for *Pa*LpxC:**4** crystal structure, for the four monomers in the *Ec*LpxC:**5a** crystal structure, for the *Pa*LpxC:**6k** crystal structure, for the *Pa*LpxC:**6t** crystal structure, differences in crystal packing interactions, overlay of four active sites in the *Ec*LpxC:**4** crystal structure, compounds **9k** and **18k** not showing efficacy in a murine thigh infection model, and scatter plots for individual in vivo efficacy studies (PDF)

## Abbreviations

CV: cardiovascular
HA: hydroxamic acid
MBP: metal-binding pharmacophore
LpxC: UDP-3-*O*-(*R*-3-hydroxymyristoyl)-*N*-acetylglucosamine deacetylase.
