# Supplementary material for: Discovery and Optimization of Novel Nonhydroxamate LpxC Inhibitors for the Treatment of Multidrug-Resistant Gram-Negative Infections
Source: J Med Chem. 2026 Aug 4;69(15):18692–704. doi: 10.1021/acs.jmedchem.6c01036 (PMC13492277; doi:10.1021/acs.jmedchem.6c01036)
Supplement: Supplementary file 1 [file jm6c01036_si_002.pdf]

SUPPORTING INFORMATION

Discovery and optimization of novel non-hydroxamate LpxC inhibitors for the treatment of multidrug-resistant Gram-negative infections

David P. Martin*, Min Teng, Baskar Nammalwar, Christian Perez, Xiaoming Li, Jason Munguia, Konstantin Taganov, Junhua Fan, Sanjay Agarwalla, David Lonergan, Andrew P. Tomaras, Zachary Zimmerman, and David T. Puerta

Blacksmith Medicines, Inc., San Diego, California 92121, United States

*E-mail: martin@blacksmithmedicines.com

TABLE OF CONTENTS

General Information S3

General Synthetic Procedures S3

Compound Synthesis and Characterization

Procedures for the preparation of Compound **2** S8

Procedures for the preparation of Compound **6** S8

Procedures for the preparation of Compound **7a** S14

Procedures for the preparation of Compounds **8a-w** S16

Procedures for the preparation of Compound **9k** S69

Procedures for the preparation of Compound **10k** S72

Procedures for the preparation of Compounds **11k** and **11t** S74

Procedures for the preparation of Compound **12k**.. S77

Procedures for the preparation of Compounds **13b-t** S79

Procedures for the preparation of Compound **14k**.. S87

Procedures for the preparation of Compound **15k**.. S88

Procedures for the preparation of Compound **16k**.. S90

Procedures for the preparation of Compounds **17b** and **17k** S92

Procedures for the preparation of Compounds **18b** and **18k** S96

LC/MS traces for Compounds **2**, **8b**, **8g**, **8j**, **8k**, **8m**, **8n**, **8p-t**, **10k**, **11t**, **12k**, **13b**, **13g**, **13h**, **13k**, **13p**, **13t**, **14k**, **16k**, **18b**, and **18k**... S100

Table S1 .... S125

Table S2 .... S125

Table S3 .... S126

Table S4 .... S127

Figure S1 .... S128

Figure S2 .... S128

Figure S3 .... S129

Figure S4 .... S129

Figure S5 .... S130

Figure S6 .... S130

Figure S7 .... S131

Figure S8 .... S131

General Information. Starting materials, reagents, and solvents were purchased from commercial sources and used without further purification unless otherwise noted. Silica gel chromatography (230-400 mesh) was performed using manually packed columns. Preparative HPLC was performed using either YMC-ACTUS-TRIART C18 or RediSep Gold C18 SiO_2 columns with 1% formic acid in acetonitrile or 10 mM $NaHCO_3$ in H_20/acetonitrile as the eluent. When acid salts of final products were obtained, they were passed through PL_HCO_3 MP SPE resin to generate the free base. For LC-MS traces, separation was achieved on Atlantis dC18 (50 x 4.6) columns using a flow rate of 1.5 mL/min and a gradient of 5-95% acetonitrile in Mobile Phase A (0.1% formic acid in H_2O). SFC separations were carried out using (R,R)-WHELK or CHIRALPAK-IH columns with a mixture of CO_2 and 0.5% isopropyl amine in MeOH as the eluent. NMR spectra were run on Bruker Avance spectrometers (400 MHz) at 296 K with chemical shifts (δ, in parts per million) referenced to the solvent peak. Coupling constants (J) are given where possible.

General Procedure A:

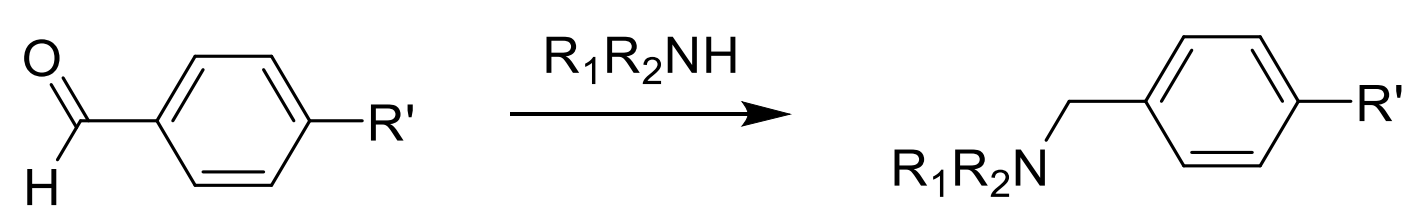

To a solution of aldehyde (~100 mM) in MeOH or a mixture of MeOH and DCM was added amine (2 eq) and the mixture stirred at room temperature for 2 h. $NaBH_3CN$ (3 eq) was then added and the mixture stirred for an additional 2-16 h until completion. The solvent was then removed under reduced pressure and the resulting residue purified by silica gel chromatography.

General Procedure B:

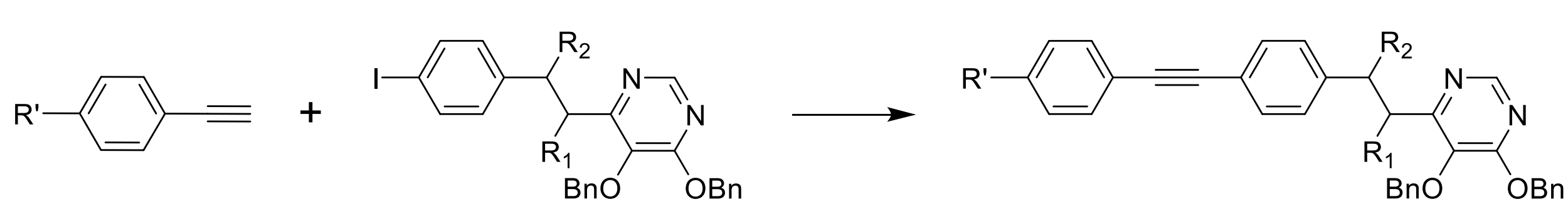

To a solution of iodophenyl analog (1 eq) in toluene (~100 mM) was added the appropriate alkyne (1.5 eq) along with triethyl amine (4 eq). The reaction mixture was purged with nitrogen for 10 min, followed by the addition of $Pd(PPh_3)_2$ (0.02 eq) and CuI (0.06 eq) and the reaction mixture was stirred for 3 h at 25 °C. After completion of the reaction, solvent was removed under reduced pressure and the reaction mixture was dissolved in water and extracted with EtOAc. The combined organic layers were washed with brine, dried over Na_2SO_4, filtered and concentrated and the crude product was purified by silica gel chromatography.

General Procedure C:

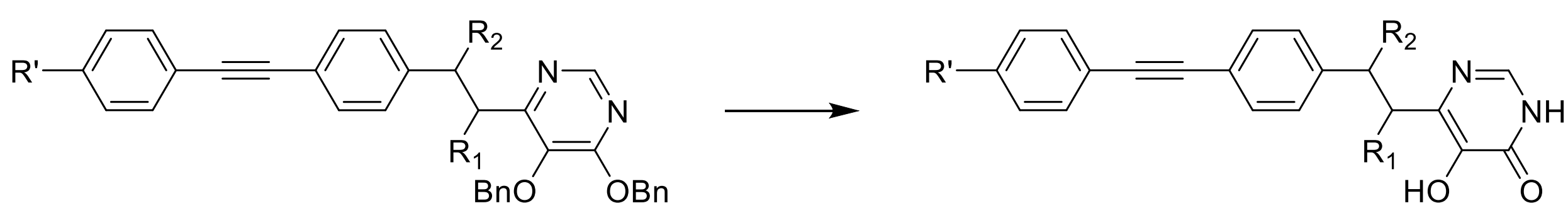

To a cooled solution of benzyl-protected compound in DCM (~ 15 mM) was added BCl_3 (1 M in DCM, 20 eq) and the mixture allowed to warm to 25 °C and stir for 2 h. The reaction mixture was quenched with methanol, concentrated under reduced pressure, and purified by reverse phase HPLC.

General Procedure D:

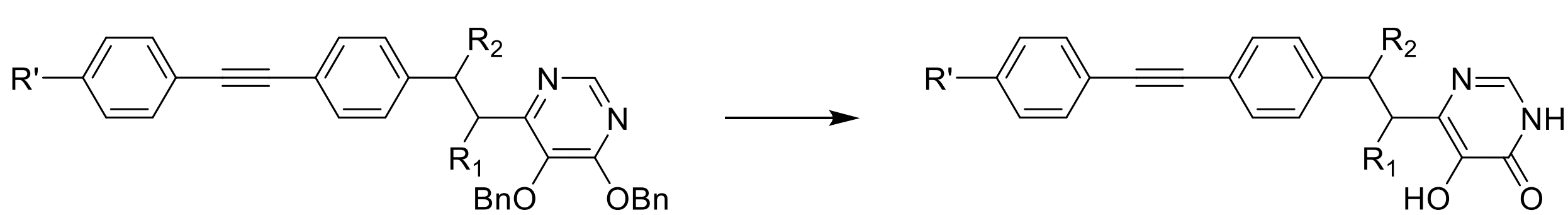

In a sealed tube, a solution of benzyl-protected compound (~40 mM) in 1:1 DCE:TFA was heated to 100 °C for 2 h. The solvent was then removed under reduced pressure and the residue

taken up in 10% MeOH in DCM. The resulting solution was washed with saturated aqueous $NaHCO_3$, dried over Na_2SO_4, and concentrated prior to purification by reverse phase HPLC.

General Procedure E:

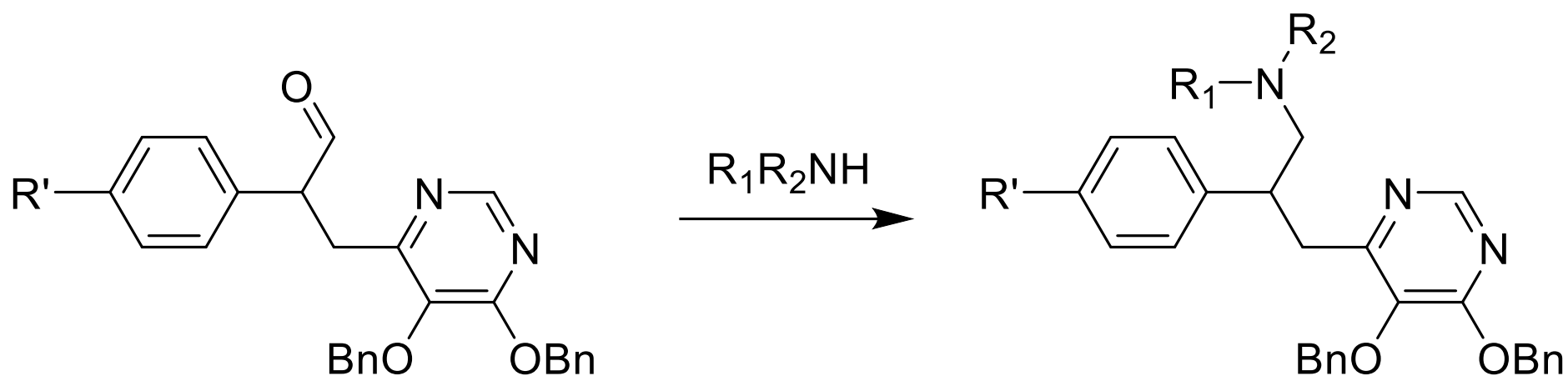

To a solution of aldehyde (~100 mM) in 2:1 MeOH:DCM was added amine (1.2 eq) and acetic acid (1.2 eq) and the mixture stirred at room temperature for 2-16 h. Cyanoborohydride resin (3 eq) was then added and the mixture stirred until completion, 1-2 h. The solvent was then removed under reduced pressure and the resulting residue purified by silica gel chromatography.

General Procedure F:

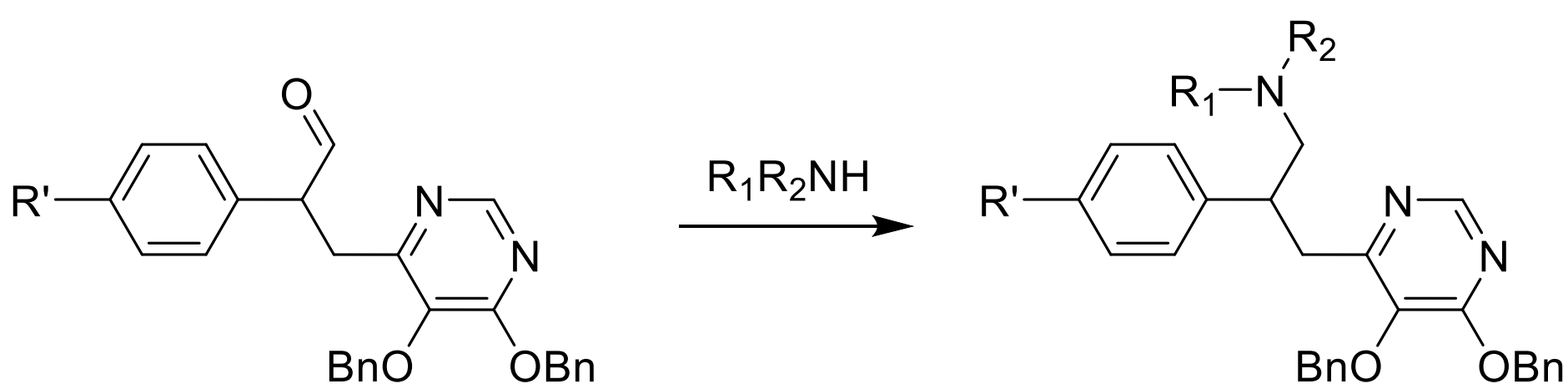

To a solution of aldehyde (~100 mM) in a ~5:1 mixture of MeOH and THF was added amine (1.2 eq), acetic acid (1.2 eq; sodium acetate used for amines supplied as the HCl salt), and 2-picoline borane complex (1 eq). The mixture stirred for 12 h at room temperature before the solvent was then removed under reduced pressure and the resulting residue purified by silica gel chromatography.

General Procedure G:

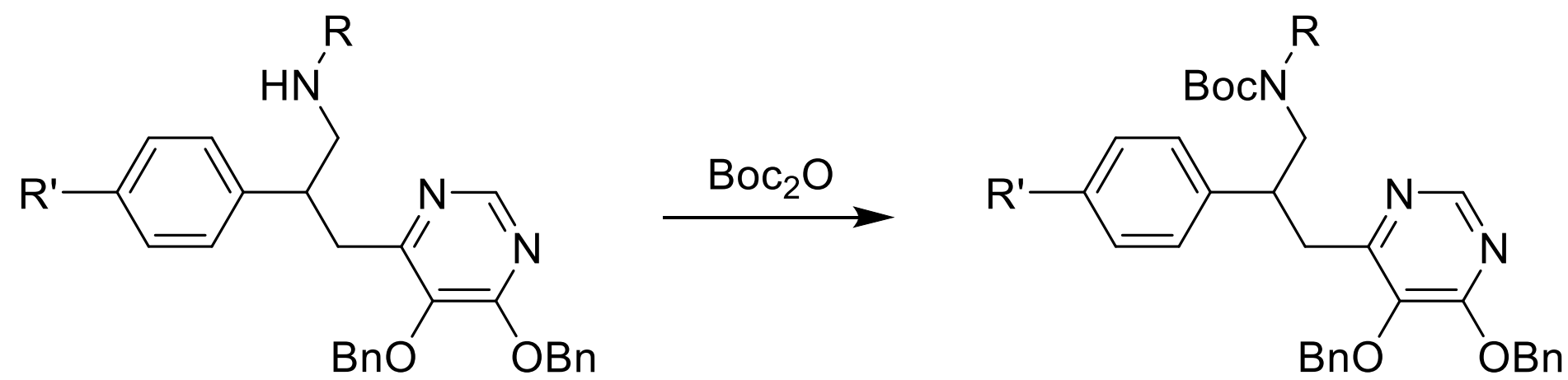

To a solution of amine (~50 mM) in THF or DCM, TEA (2 eq) and $(Boc)_2O$ (1.2 eq) were added and stirred for 2-6 h. After completion of the reaction, water was added and the mixture extracted with EtOAc, combined organic layers were washed with brine solution and organic layer was dried over Na_2SO_4 and concentrated and the crude product was purified by silica gel chromatography. The resulting racemate was separated by SFC before proceeding to the next step.

General Procedure H:

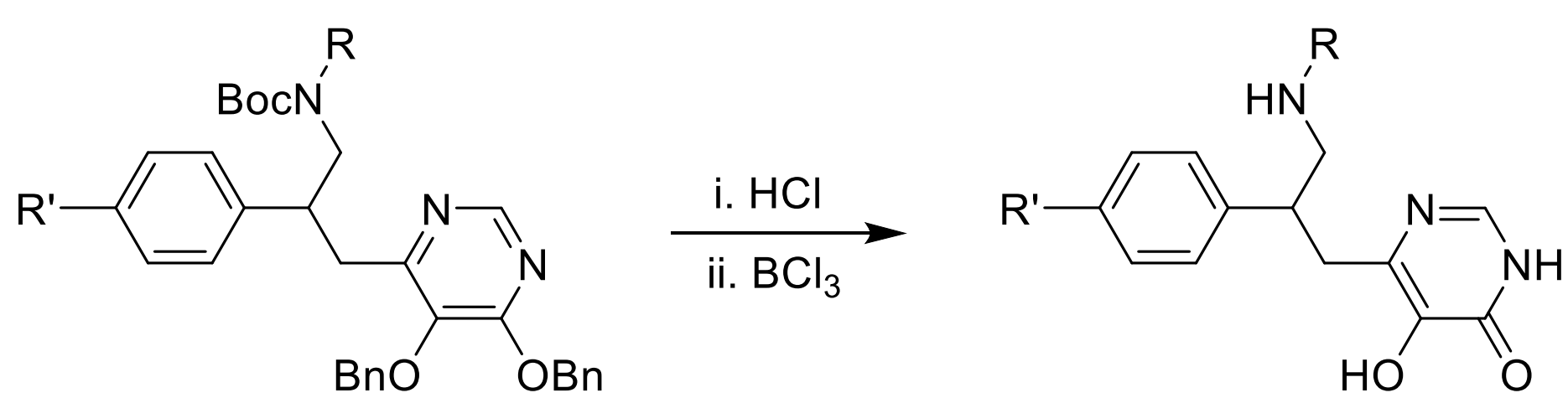

Compounds were dissolved in DCM (~25 mM) followed by the addition of an equal volume of HCl (4 M in 1,4-dioxane, ~100 eq) was added and the reaction stirred until completion (3-14 h). Solvent was distilled off under reduced pressure and the crude was taken up in saturated $NaHCO_3$ and extracted with DCM. The organic layer was dried over Na_2SO_4, concentrated, and taken up in dry DCM (2 mL). BCl_3 (1M in DCM, 5 mL) was added and the mixture stirred at 25 °C for 2 h. Then the reaction mixture was quenched with methanol concentrated under reduced pressure. The crude product was purified by HPLC.

General Procedure I:

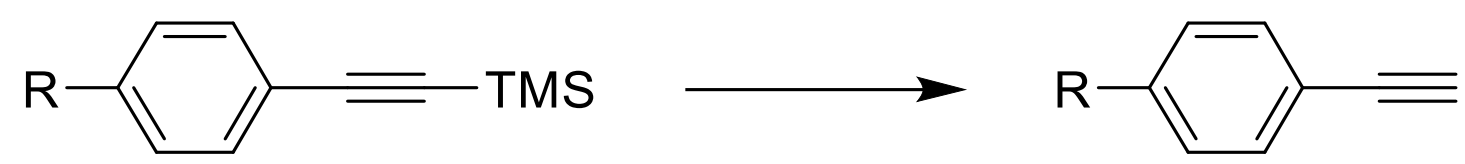

To a solution of TMS-alkyne in dry DCM (~150 mM) was added TBAF (3 eq) and the reaction mixture was stirred at 25 °C for 1 h. After completion of the reaction, water (5x volume) was added and organic layer was separated, dried over Na_2SO_4, filtered and concentrated under reduced pressure to obtain the desired product.

General Procedure J

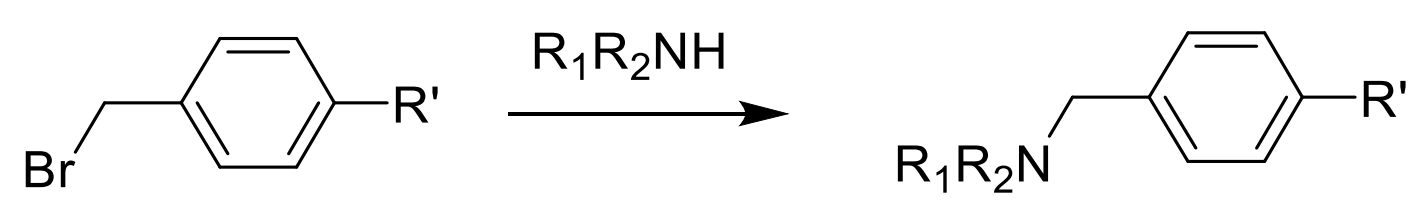

To a solution of 4-substituted bromomethylbenzene in DMF (~300 mM) was added K_2CO_3 (2 eq), amine (1.2 eq) and the reaction mixture stirred at 25 °C for 3 h. After the completion of SM by TLC, crushed ice was added to the reaction mass and the obtained solid was filtered and dried under reduced pressure to yield product.

Procedures for the preparation of Compound 2 (ACHN-975)

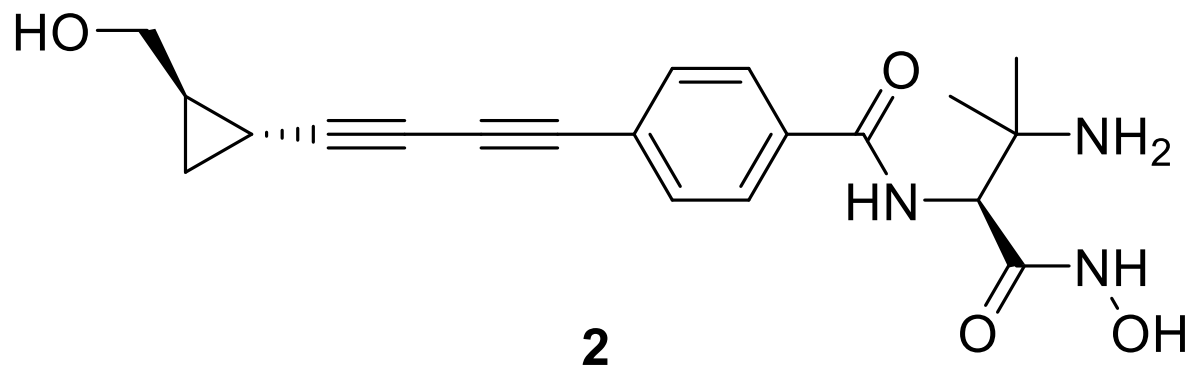

2

Compound **2** was prepared as described in patent application WO2012154204. LC-MS (m/z): 370.2 $[M+H]^+$. ^{1}H-NMR (D_2O): δ 8.313 (s, 1 H), 7.875 (d, 2H, J = 5.0 Hz), 7.621 (d, 2H, J = 5.0 Hz), 4.498 (s, 1H), 3.435-3.394 (m, 1H), 3.254-3.210 (m, 1H), 1.466-1.417 (m, 2H), 1.227 (s, 3H), 1.165 (s, 3H), 0.959-0.925 (m, 2H).

Procedures for the preparation of Compound 6

4,6-dichloropyrimidin-5-ol (20):

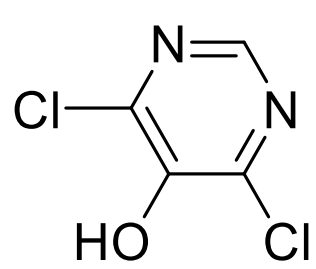

20

To a solution of 4,6-dichloro-5-methoxypyrimidine (50.0 g, 0.270 mol) in dichloroethane (800 mL) at 0 °C was added anhydrous aluminum chloride (50.5 g, 0.411 mol) in a single portion. The reaction mixture was warmed to 50 °C and stirred vigorously for 6 h. The mixture was cooled to 0 °C and aqueous HCl solution (1 M, 400 mL) was slowly added followed by MeOH (100 mL). The mixture was allowed to warm to room temperature and stirred vigorously for 10 min, then diluted with water and extracted with EtOAc. The combined organic layers were dried over Na_2SO_4, filtered and concentrated to afford **20** (41.0 g, 91%). LC-MS (m/z): 165.9 $[M+H]^+$.

5-(benzyloxy)-4,6-dichloropyrimidine (21):

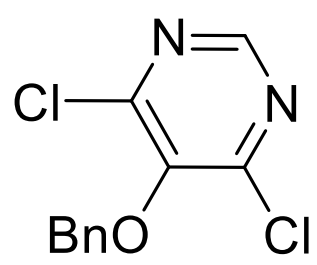

21

To a solution of **20** (6.50 g, 35.5 mmol) in DMF (120 mL), benzyl bromide (8.42 mL, 71.0 mmol) was added followed by potassium carbonate (14.70 g, 106 mmol). The reaction mixture was stirred at 60 °C for 1 h. The mixture was concentrated and the residue was partitioned between EtOAc and water. The organic layer was washed with water and brine, dried over Na_2SO_4, filtered and concentrated. The residue was purified by flash chromatography on silica gel (10% EtOAc in pet. ether) to afford **21** (9.5 g, 94%). LC-MS (m/z): 256.1 $[M+H]^+$.

4,5-bis(benzyloxy)-6-chloropyrimidine (22):

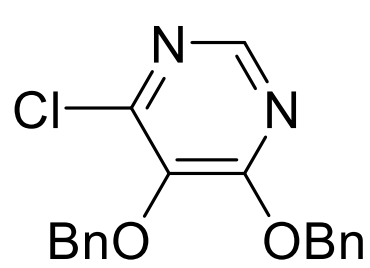

22

To a solution of benzyl alcohol (3.80 g, 0.0352 mol) in THF (100 mL), NaH (60% in mineral oil, 1.40 g, 0.0352 mol) was added at 0 °C and stirred for 30 min. To this a solution of **21** (9.00 g, 0.035 mol) in THF (20 mL) was added at 0 °C and stirred for 30 min. After completion of the reaction, the reaction mixture was diluted with water and extracted with EtOAc. The organic layer was washed with brine solution and dried over Na_2SO_4, filtered and concentrated under reduced pressure to get **22** (11 g, 96%). LC-MS (m/z): 327.6 $[M+H]^+$.

Ethyl-2-[5,6-bis(benzyloxy)pyrimidin-4-yl]acetate (23):

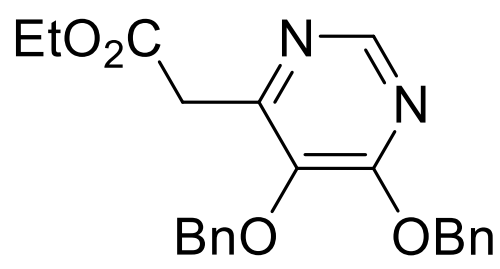

23

To a solution of **22** (10.0 g, 0.0306 mol) in dry toluene (20 mL), diethyl malonate (29.4 g, 0.183 mol) and potassium tert-butoxide (10.3 g, 0.0918 mol) were added and the mixture refluxed for 3 h. After completion of the reaction, the reaction mixture was diluted with water and extracted with EtOAc (2 x 100 mL). The combined organic layers were washed with brine solution and organic layer was dried over anhydrous Na_2SO_4, filtered and concentrated under reduced pressure. The residue was purified by flash chromatography on silica gel (15% EtOAc in pet. ether) to afford **23** (3.0 g, 26%). LC-MS (m/z): 379.4 $[M+H]^+$.

Ethyl-2-[5,6-bis(benzyloxy)pyrimidin-4-yl]-3-(4-iodophenyl)propanoate (24):

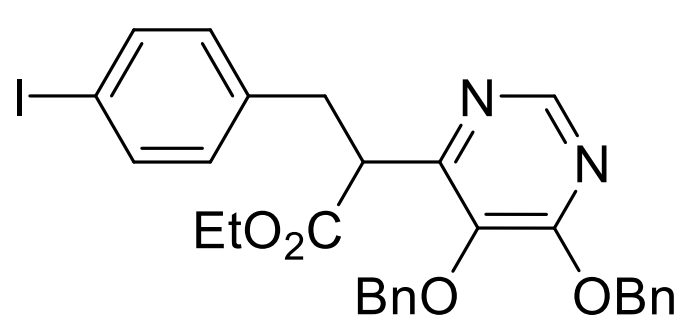

24

To a solution of **23** (3.0 g, 0.0079 mol) in DMF (30 mL), NaH (60%, 0.317 g, 0.00790 mol) was added at 0 °C and stirred for 10 min. Then 4-iodobenzyl bromide (2.35 g, and 0.00790 mol) was added and stirred at 0 °C for 1 h. After completion of the reaction, the reaction mixture was quenched with saturated aqueous NH_4Cl, diluted with water, and extracted with EtOAc (2 x 10 mL). The combined organic layers were washed with brine solution and organic layer was dried over Na_2SO_4, filtered and concentrated under reduced pressure. The crude product was purified by column chromatography on silica gel (10% EtOAc in pet. ether) to afford **24** (4.4 g, 94%). LC-MS (m/z): 595.3 $[M+H]^+$.

2-[5,6-bis(benzyloxy)pyrimidin-4-yl]-3-(4-iodophenyl)propan-1-ol (25):

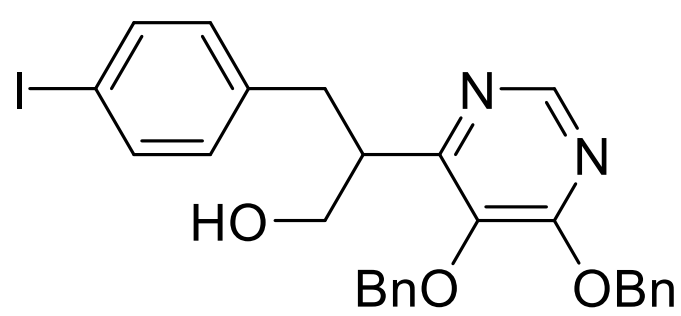

25

To solution of **24** (4.20 g, 0.00706 mol) in diethyl ether (45 mL) and ethanol (15 mL), $LiBH_4$ (0.449 g, 0.0212 mol) was added at 0 °C and stirred the reaction mass at 25 °C for 6 h. After completion of the reaction, the reaction mixture was quenched with saturated aqueous NH_4Cl and extracted with EtOAc (2 x 200 mL). The combined organic layers were washed with brine solution, dried over Na_2SO_4, filtered and concentrated and the crude product was purified by column chromatography on silica gel (20% EtOAc in pet. ether) to afford **25** (3.0 g, 77%). LC-MS (m/z): 553.3 $[M+H]^+$.

4-[1-azido-3-(4-iodophenyl)propan-2-yl]-5,6-bis(benzyloxy)pyrimidine (26):

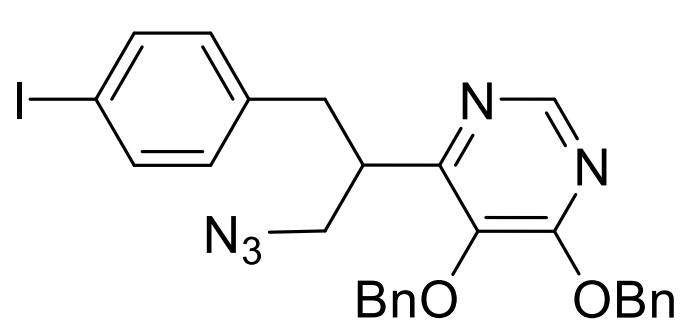

26

To a solution of **25** (3 g, 0.0054 mol) in DCM (30 mL) at 0 °C, Et_3N (1.5 mL, 0.0108 mol) and mesyl chloride (0.933 g, 0.00814 mol) was added and stirred for 1 h. After completion of the reaction, water was added and extracted with DCM and the organic layer was washed with water and brine solution and dried over Na_2SO_4, filtered and concentrated under reduced pressure. The crude mesylate (3.40 g, 0.00539 mol) was then taken up in DMF (10 mL) and NaN_3 (1.75 g, 0.0269 mol) was added and the mixture heated at 100 °C for 6 h. After completion of the reaction, water

was added and the mixture extracted with EtOAc. The organic layer was washed with water, dried over Na_2SO_4, and concentrated to get **26** (3.0 g, 97%). LC-MS (m/z): 578.3 $[M+H]^+$.

2-[5,6-bis(benzyloxy)pyrimidin-4-yl]-3-(4-iodophenyl)propan-1-amine (27):

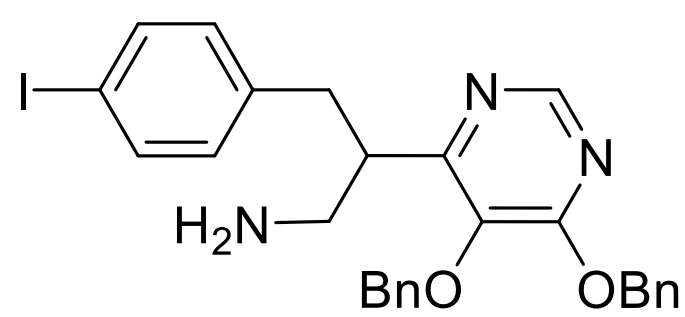

27

To a solution of **26** (3.00 g, 0.00519 mol) in a mixture of THF (45 mL) and H_2O (15 mL), PPh_3 (4.08g, 0.0155 mol) was added and the reaction was heated at 85 °C for 2 h. After completion of the reaction, the reaction mixture was concentrated and the crude product was purified by column chromatography on silica gel (20-30% MeOH in DCM) to afford **27** (2.2g, 79%). LC-MS (m/z): 552.2 $[M+H]^+$.

2-[5,6-bis(benzyloxy)pyrimidin-4-yl]-N-(2-fluoroethyl)-3-(4-iodophenyl)propan-1-amine (28):

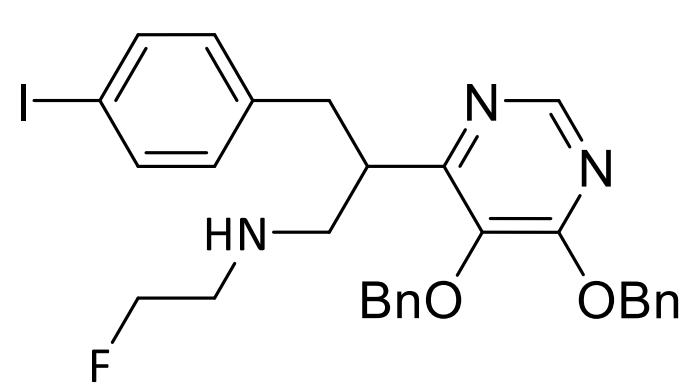

28

To a solution of **27** (1.5 g, 0.00270 mol) in 1,4-dioxane (40 mL) was added potassium carbonate (0.938 g, 0.00680 mol), 1-bromo-2-fluoroethane (1.03 g, 0.0816 mol) and CuI (0.2 g) respectively and the reaction mixture was heated at 110 °C for 16 h. The mixture was concentrated and the residue was partitioned between EtOAc and water. The organic layer was washed with water and brine, dried over Na_2SO_4, filtered and concentrated. The residue was purified by flash

chromatography on silica gel silica using 10% MeOH in DCM to afford **28** (0.75 g, 47%). LC-MS (m/z): 597.47 $[M+H]^+$.

4-(4-ethynylbenzyl)morpholine (30):

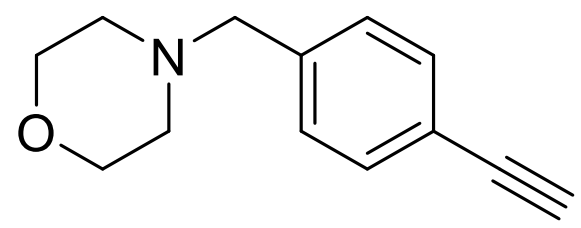

30

Compound **30** was synthesized from 4-ethynylbenzaldehyde (**29**) and morpholine utilizing General Procedure A (1.9 g, 30%). LC-MS (m/z): 202 $[M+H]^+$.

(*R*)-2-[5,6-bis(benzyloxy)pyrimidin-4-yl]-N-(2-fluoroethyl)-3-(4-{[4-(morpholinomethyl)-phenyl]ethynyl}phenyl)propan-1-amine (29):

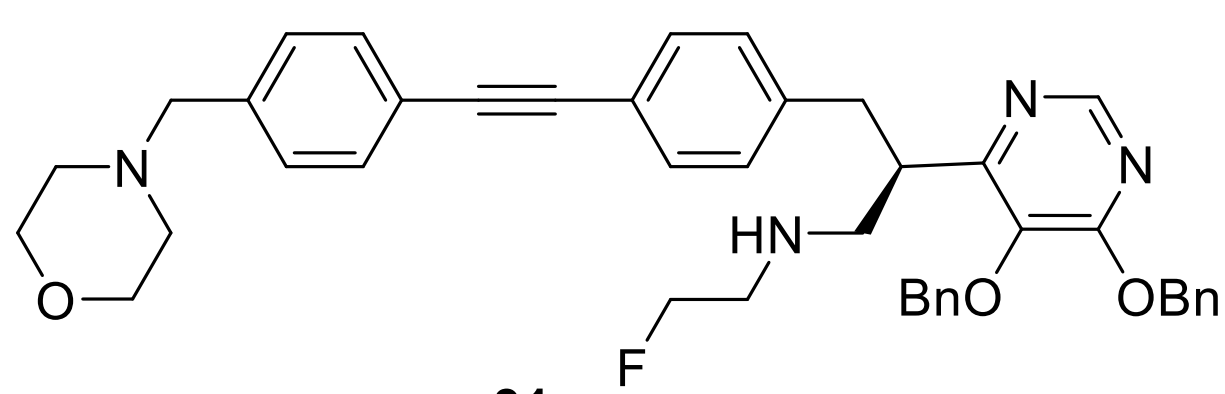

31

Compound **31** was prepared from **28** and **30** using General Procedure B (0.45 g, 54%). LC-MS (m/z): 671.7 $[M+H]^+$. The racemic mixture was separated by chiral SFC separation to obtain **31**-Enantiomer 1 (0.17 g, 3.22 min retention time) and **31**-Enantiomer 2 (0.18 g, 4.95 min retention time) with unknown absolute stereochemistry.

6-[(*R*)-1-(2-fluoroethyl)amino-3-(4-{[4-(morpholinomethyl)phenyl]ethynyl}phenyl)-propan-2-yl]-5-hydroxypyrimidin-4(3H)-one (6):

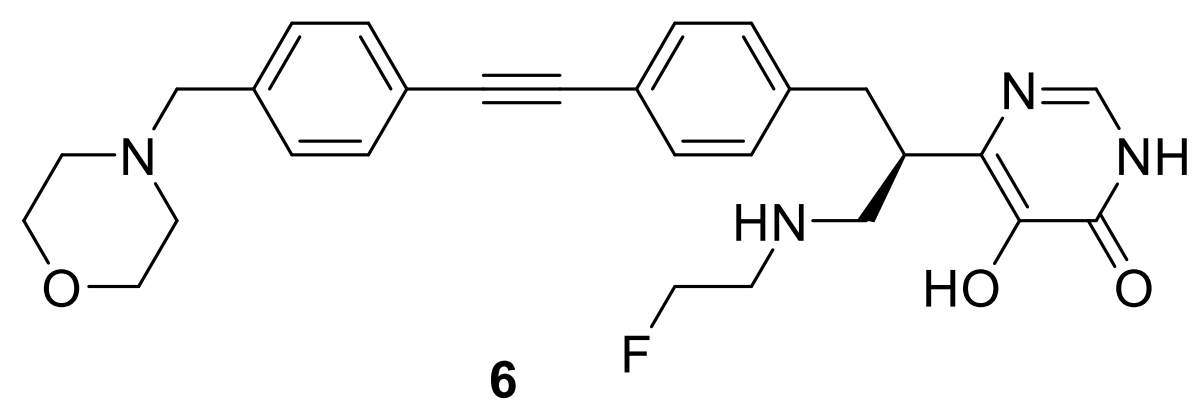

6

31-Enantiomer 2 was converted to **6** using General Procedure D (0.055 g, 44%). LC-MS (m/z): 491.2 $[M+H]^+$. ^{1}H NMR (DMSO-d_6): δ 7.8 (s, 1H), 7.48 (d, 2H, J = 8.4), 7.38 (dd, 4H, J = 8.4, 12.0), 7.13 (d, 2H, J = 8.4), 4.57 (br s, 1H), 4.45 (br s, 1H), 3.58 (t, 4H, J = 4.4), 3.50 (s, 2H), 2.96 (m, 6H), 2.50 (t, 4H, J = 4.4)

Procedures for the preparation of Compound 7a

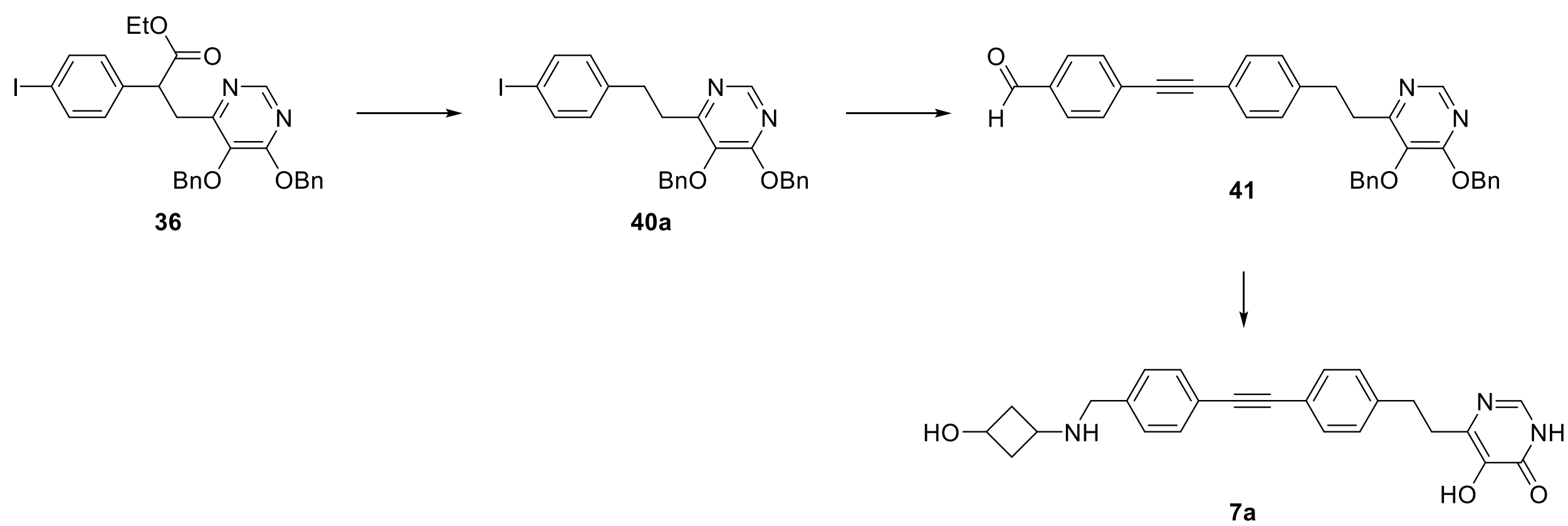

3-[5,6-bis(benzyloxy)pyrimidin-4-yl]-2-(4-iodophenyl)propanoic acid (40a):

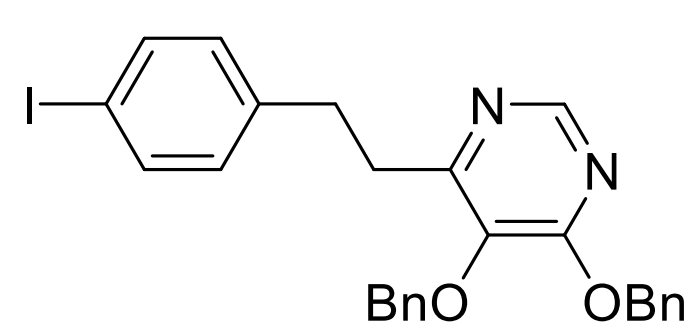

40a

To a stirred solution of **36** (2.00 g, 3.26 mmol) in 1:1 EtOH:THF (15 mL) was added 1.5 M NaOH in water (15 mL) and the resulting mixture was stirred at RT for 2 h. The reaction mixture was diluted with 100 mL water and extracted with 1:1 diethyl ether:EtOAc. The aqueous layer was acidified with 6 M aqueous HCl to pH 5-6 and again extracted with EtOAc. The combined organics

were dried over $MgSO_4$ and concentrated under reduced pressure. The resulting carboxylic acid intermediate was heated at 70 °C in a water bath under reduced pressure until full decarboxylation was observed. The crude product was then purified by silica gel chromatography eluting with a gradient of EtOAc in Heptanes from 0 to 100% to afford **40a** (600 mg, 33%). LC-MS (m/z): 523 $[M+H]^+$.

4-[(4-{2-[5,6-bis(benzyloxy)pyrimidin-4-yl]ethyl}phenyl)ethynyl]benzaldehyde (41):

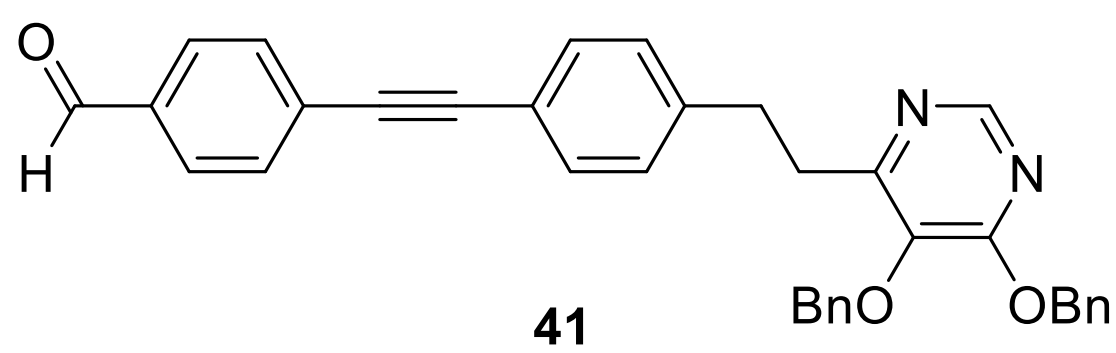

41

The compound was prepared from **40a** and 4-ethynylbenzaldehyde using General Procedure B (300 mg, 37%). LC-MS (m/z): LC-MS (m/z): 525 $[M+H]^+$.

3-[5,6-bis(benzyloxy)pyrimidin-4-yl]-2-(4-iodophenyl)propanoic acid (7a):

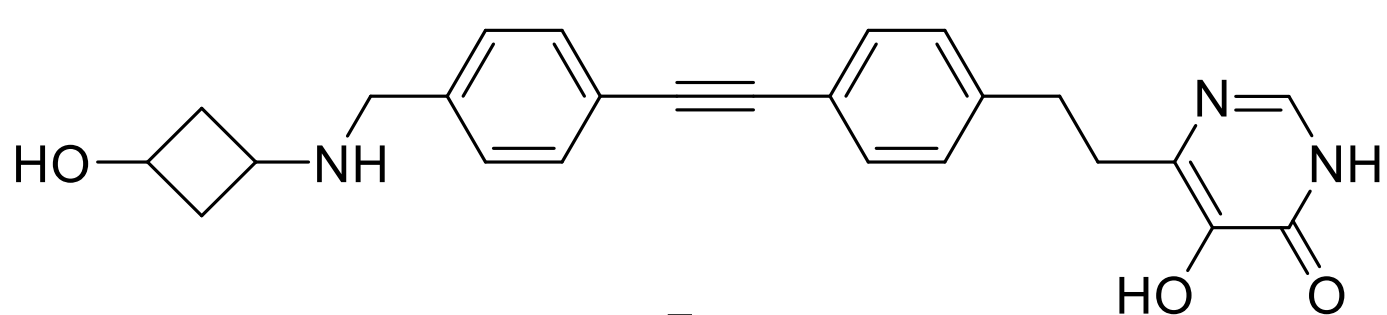

7a

Compound **7a** was generated from **41** by reductive amination with 3-aminocyclobutan-1-ol (General Procedure A) and then, without purification of the intermediate, proceeding to debenzylation utilizing General Procedure C (80 mg, 34%). LC-MS (m/z): 416.5 $[M+H]^+$. ^1H NMR (DMSO-d_6): δ 7.73 (s, 1H), 7.48-7.42 (m, 4H), 7.35 (d, 2H, J = 8.0), 7.23 (d, 2H, J = 8.0), 2.92 (t, 2H, J = 6.8), 2.80 (t, 2H, J = 6.8), 2.67-2.63 (m, 1H), 2.42-2.36 (m, 3H), 1.95-1.92 (m, 1H), 1.60-1.55 (m, 2H).

Procedures for the preparation of Compound 8a

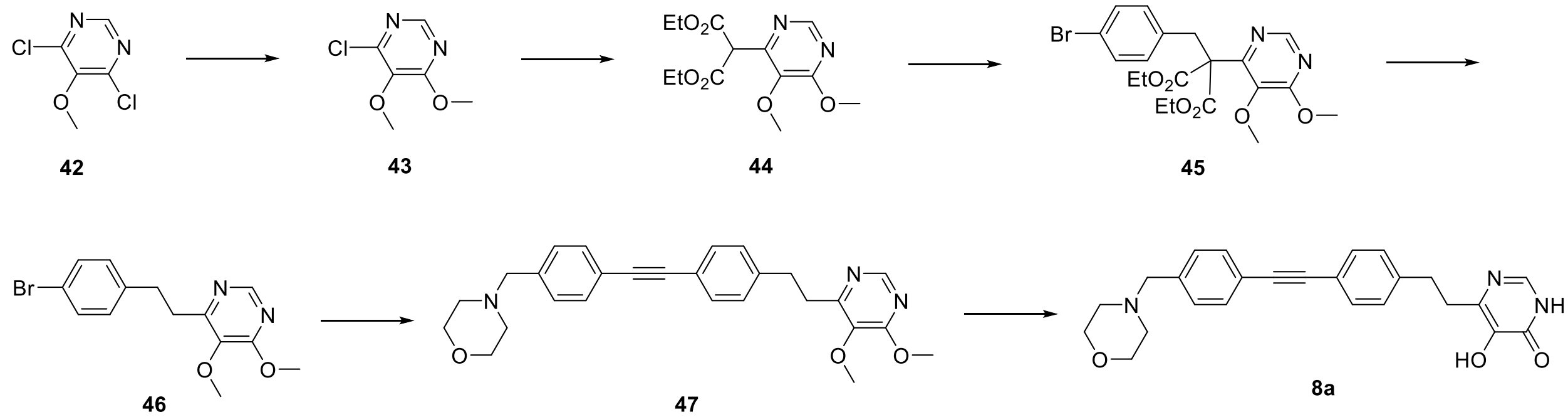

4,5-bis(benzyloxy)-6-vinylpyrimidine (43):

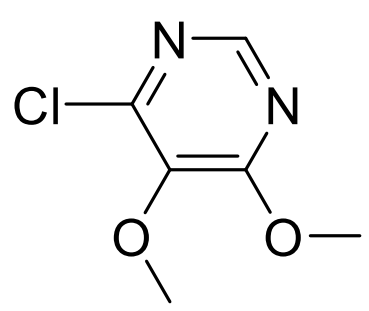

43

To a solution of **42** (25.0 g, 0.139 mol, 1 eq) in methanol (300 mL), NaOMe (33 mL 25% wt in methanol) was added slowly at 0 °C over 30 min. The reaction mixture was then allowed to warm to 25 °C and stirred for 12 h at which point the mixture was quenched with saturated aqueous NH_4Cl. The solvent was removed under reduced pressure, the crude product dissolved in dichloromethane and washed with water and brine solution. The organic layer was dried over Na_2SO_4, filtered and concentrated to yield **43** (18 g, 73%). LC-MS (m/z): 175 $[M+H]^+$

Diethyl-2-(5,6-dimethoxypyrimidin-4-yl)malonate (44):

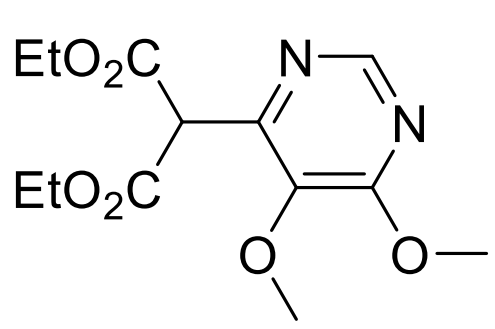

44

Diethyl malonate (3.3 g, 0.020 mol) was added slowly to a slurry of 60% NaH (0.50 g, 0.12 mol) in DMF (20 mL) at 0 °C and stirred for 30 min. To this stirred solution, **43** (6.0 g, 0.020 mol) and

CuI (0.12 g, 0.0013 mol) were added and heated to 100 °C for 4 h. After completion of the reaction, reaction mixture was cooled and diluted with water and extracted with EtOAc (2 x 200 mL). The combined organic layers were washed with brine solution, dried over Na_2SO4, filtered and concentrated. The crude product was purified by column chromatography to yield **44** (0.80 g, 21%).

Diethyl-2-(4-bromobenzyl)-2-(5,6-dimethoxypyrimidin-4-yl)malonate (45):

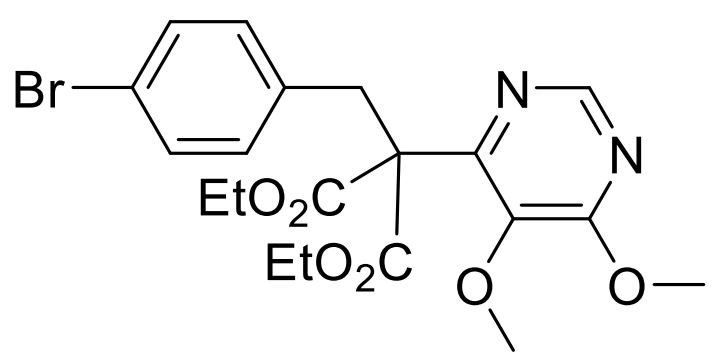

45

To a solution of **44** (0.20 g, 0.67 mmol) in DMF (5 mL), Cs_2CO_3 (1.5 eq) and 4-bromo benzyl bromide (1 eq) were added and stirred at 25 °C for 1 h. After completion of the reaction, the reaction mixture was diluted with water and extracted with EtOAc (2 x 25 mL). The combined organic layers were washed with brine solution, dried over Na_2SO_4, filtered and concentrated. The crude product was purified by column chromatography to yield **45** (0.21 g, 67%). LC-MS (m/z): 469.32 $[M+H]^+$.

4-(4-bromophenethyl)-5,6-dimethoxypyrimidine (46):

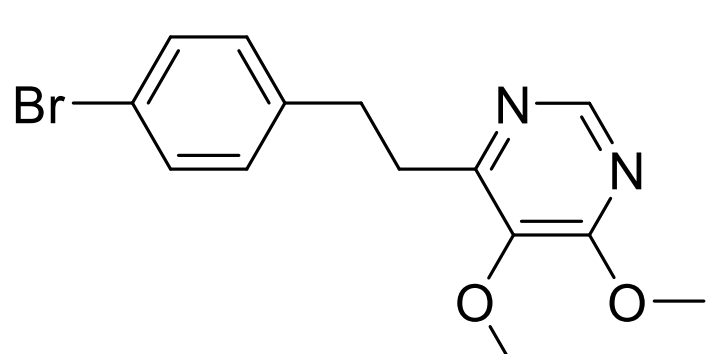

46

To a solution of **45** (0.20 g, 0.74 mmol) in EtOH (3 mL) and H_2O (3 mL) was added NaOH (0.15 g, 3.7 mmol) and the mixture stirred at 25 °C for 3 h. After completion of the reaction, the reaction mixture was diluted with water, acidified with 1.5 N HCl and extracted with EtOAc (2 x 20 mL).

The combined organic layers were concentrated and the obtained crude product was stirred in 2N HCl for 3h. After completion of the reaction, the reaction mixture was diluted with water and extracted with EtOAc (2 x 10 mL). The combined organic layers were washed with brine solution, dried over Na_2SO_4, filtered and concentrated. The crude product was purified by column chromatography to yield **46** (0.058 g, 45%). LC-MS (m/z): 325.2 $[M+H]^+$.

4-[4-({4-[2-(5,6-dimethoxypyrimidin-4-yl)ethyl]phenyl}ethynyl)benzyl]morpholine (47):

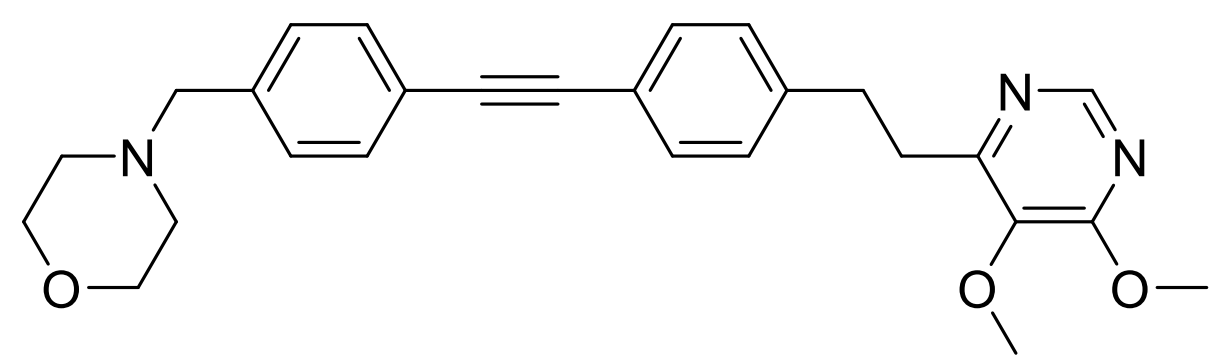

47

To solution of **46** (0.050 g, 0.14 mmol) in DMF (1 mL) were added 4-(4-ethynylbenzyl)-morpholine (0.055 g, 0.27 mmol), dipropylamine (0.057 g, 0.57 mmol) and triphenylphosphine (0.05 g, 0.0002 mol). The reaction mixture was purged with nitrogen for 10 min, $PdCl_2(PPh_3)_2$ (4.0 mg, 0.014 mmol) was added followed by CuI (2 mg, 0.08 mmol) and the reaction mixture heated in a sealed tube for 3 h at 100 °C. The reaction mixture was then diluted with water and extracted with EtOAc (2 x 10 mL). The combined organic layers were washed with brine solution, dried over Na_2SO_4, filtered and concentrated and the crude product was purified by column chromatography to yield **47** (0.030 g, 44%). LC-MS (m/z): 444.2 $[M+H]^+$.

5-hydroxy-6-(4-{[4-(morpholinomethyl)phenyl]ethynyl}phenethyl)pyrimidin-4(3H)-one (8a):

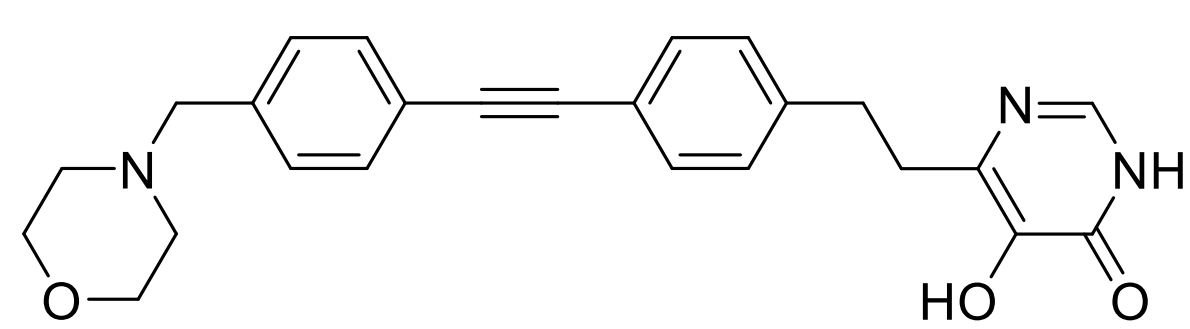

8a

To a solution of **47** (0.030 g, 0.070 mmol) in pyridine, lithium iodide (0.056 g, 0.42 mmol) was added and microwaved for 2 h at 170 °C. After completion of the reaction, the solvent was removed and the residue dissolved in EtOAc followed by washing with water and brine solution. The organic layer was dried over Na_2SO_4, filtered and concentrated. The crude product was purified by prep TLC to yield **8a** (5.0 mg, 18%). LC-MS (m/z): 416 $[M+H]^+$. ^{1}H-NMR (DMSO-d_6): δ 7.72 (br s, 1H), 7.48-7.41 (m, 4H), 7.34 (d, J = 7.60 Hz, 2H), 7.23 (d, J = 6.80 Hz, 2H), 3.56 (m, 4H), 3.46 (m, 2H), 2.90 (m, 2H), 2.79 (m, 2H), 2.34 (m, 4H)

Procedures for the preparation of Compound 8b

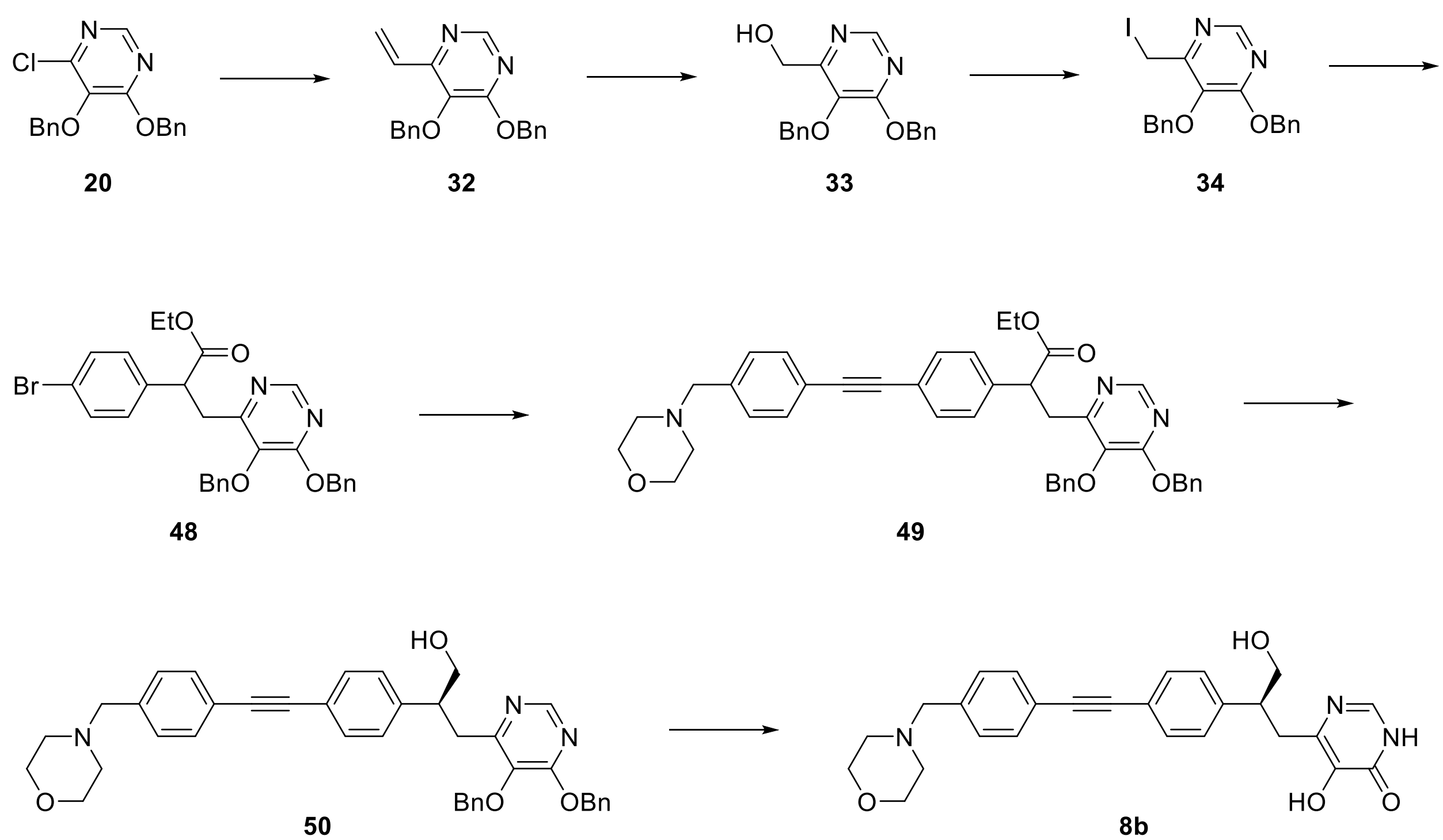

4,5-bis(benzyloxy)-6-vinylpyrimidine (32):

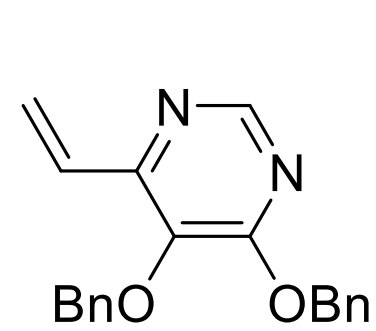

32

To a solution of **20** (25 g, 0.077 mol) in DMF (250 mL, purged with nitrogen for 15 min), tributyl(vinyl)tin (26.79 g, 0.8435 mol) was added and purged with nitrogen for an additional 10 min. To this purged reaction mixture $Pd(PPh_3)_4$ (5.3 g, 0.0046 mol) was added and heated to 100 °C for 12 h. After completion of the reaction, the reaction mixture was cooled and diluted with water and extracted with EtOAc (2 x 750 mL). The combined organic layers were washed with brine solution, dried over Na_2SO_4, filtered and concentrated at 40-45 °C. The crude product was purified by silica gel chromatography (Petether/EtOAc gradient, 5-10%) to get **32** (~20 g, 80%). To avoid polymerization of the product, it was concentrated at 35-37 °C, was not dried completely, and was taken to the next step immediately. LC-MS (m/z): 319.2 $[M+H]^+$.

[5,6-bis(benzyloxy)pyrimidin-4-yl]methanol (33):

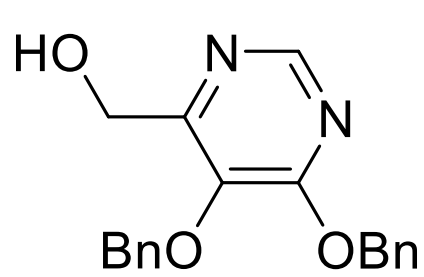

33

A solution of **32** (20.0 g, 0.0628 mol) in 1:1 DCM:MeOH (200 mL) was cooled to -78 °C and treated with ozone for 2 hr. After completion of the reaction, the reaction mixture was bubbled with oxygen for 10 min. Dimethyl sulfide (10 mL, 0.5 vol.) was added at -78 °C and the reaction mixture stirred for 1 h. The reaction mixture was then warmed to -30 °C and sodium borohydride (4.75 g, 0.126 mol) was carefully added in portions and then stirred for 20 min. The reaction mixture was diluted with DCM (1 L) and washed with water and brine, dried over Na_2SO_4, and concentrated. The crude product was purified by silica gel chromatography (Petether/EtOAc gradient, 35-45%) to get of **33** (10 g, 50%). LC-MS (m/z): 323.2 $[M+H]^+$.

4,5-bis(benzyloxy)-6-(iodomethyl)pyrimidine (34):

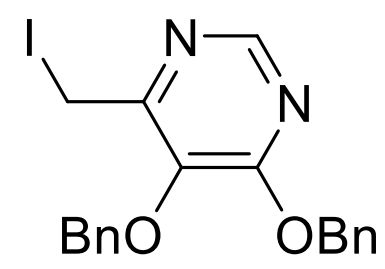

34

To a solution of **33** (10.0 g, 0.0310 mol) in DCM (100 mL) at 0 °C, triethylamine (6.3 g, 0.062 mol) was added followed by methanesulfonyl chloride (5.33 g, 0.0465 mol). The mixture was stirred at 0 °C for 30 min and then quenched with cold water. After completion of the reaction, the mixture was diluted with DCM and washed with water and brine solution. The organic layer was dried over anhydrous Na_2SO_4, filtered and concentrated under reduced pressure at 35-37 °C**.** The crude mesylate (12 g, 0.030 mol) was dissolved in acetone (120 mL) and cooled to 0 °C. To this NaI (9.0 g, 0.060 mol) was added and the mixture stirred at 0 °C for 30 min. After completion of the reaction, the mixture was diluted with water and extracted with DCM (500 mL). The organic layer was washed with brine solution and dried over anhydrous Na_2SO_4, filtered and concentrated under reduced pressure. Crude product was washed with cooled hexane to yield pure **34**. LC-MS (m/z): 433.1 $[M+H]^+$.

Ethyl-3-[5,6-bis(benzyloxy)pyrimidin-4-yl]-2-(4-bromophenyl)propanoate (48):

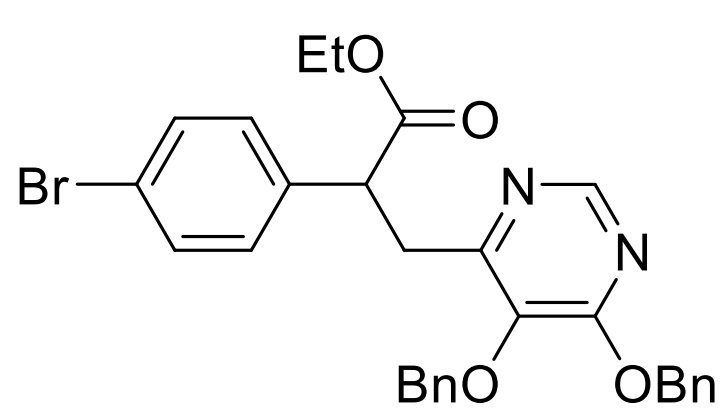

48

To a solution of ethyl-2-(4-bromophenyl)acetate (2.24 g, 0.00920 mol) in DMF (40 mL), NaH (60%, 0.37 g, 0.0092 mol) was added at 0 °C and stirred for 10 min. Then **34** (4.0 g, 0.0092 mol) was added to the reaction mixture and stirred at 0 °C for 1 h. After completion of the reaction, the reaction mixture was quenched with saturated aqueous NH_4Cl and diluted with water and extracted

with EtOAc (2 x 100 mL). The combined organic layers were washed with brine solution and organic layer was dried over Na_2SO_4, filtered and concentrated under reduced pressure. The crude product was purified by silica gel chromatography (Pet ether/EtOAc gradient, 0-10%) yield **48** (3.7 g, 74%). LC-MS (m/z): 547.2 $[M+H]^+$.

Ethyl 3-[5,6-bis(benzyloxy)pyrimidin-4-yl]-2-(4-{[4-(morpholinomethyl)phenyl]ethynyl}-phenyl)propanoate (49):

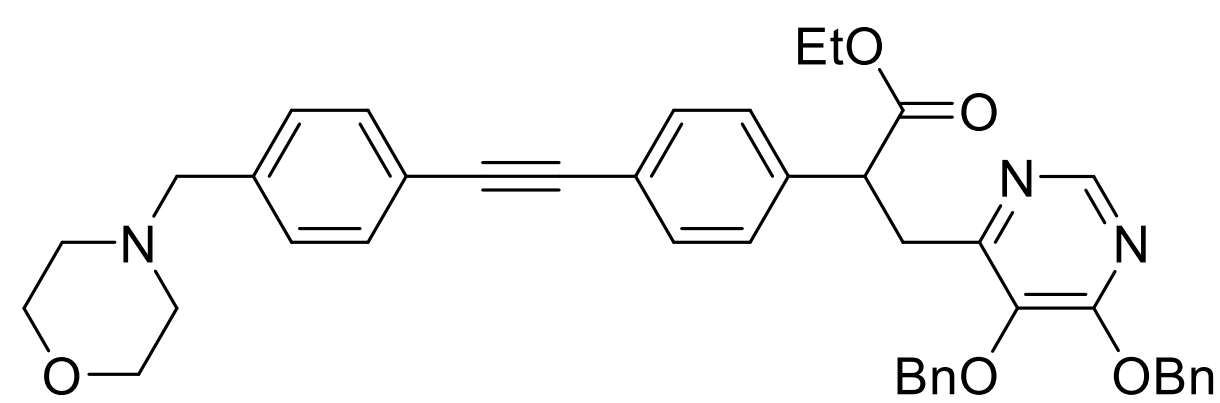

49

To a solution of **48** (3.7 g, 0.0067 mol) in DMF (40 mL) were added **30** (2.8 g, 0.014 mol), triethylamine (6.8 g, 0.068 mol) and PPh_3 (0.17 g, 0.0000067 mol) and the reaction was degassed with nitrogen for 15 min. $PdCl_2(PPh_3)_2$ (0.094 g, 0.00013 mol) was added followed by CuI (0.076 g, 0.00040 mol) and the reaction mixture was heated at 100 °C for 2 h. The reaction mixture was then diluted with water and extracted with EtOAc (2 x 100 mL). The combined organic layers were washed with brine solution, dried over Na_2SO_4, filtered and concentrated and the crude product was purified by silica gel chromatography (DCM/MeOH gradient, 15-30 %) to yield **49**. LC-MS (m/z): 668.4 $[M+H]^+$.

(*S*)-3-[5,6-bis(benzyloxy)pyrimidin-4-yl]-2-(4-{[4-(morpholinomethyl)phenyl]ethynyl}-phenyl)propan-1-ol (50):

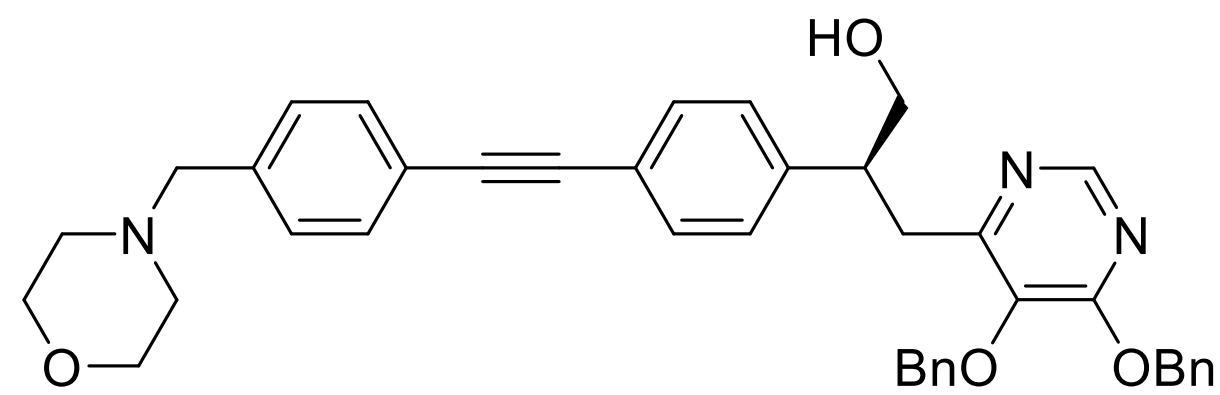

50

To a solution of **49** (0.30 g, 0.00045 mol) in a mixture of diethyl ether (7 mL) and EtOH (3 mL), $LiBH_4$ (0.029 g, 0.0013 mol) was added and stirred at 25 °C for 3 h. The reaction mixture was then quenched with saturated aqueous NH_4Cl and diluted with water. The reaction mixture was extracted with EtOAc (2 x 10 mL). The combined organic layers were dried over Na_2SO_4, filtered, concentrated under reduced pressure to yield a racemic mixture (0.18 g, 64%) which was then purified by SFC to yield **50**. LC-MS (m/z): 626.4 $[M+H]^+$.

(*S*)-5-hydroxy-6-[3-hydroxy-2-(4-{[4-(morpholinomethyl)phenyl]ethynyl}phenyl)propyl]-pyrimidin-4(3H)-one (8b):

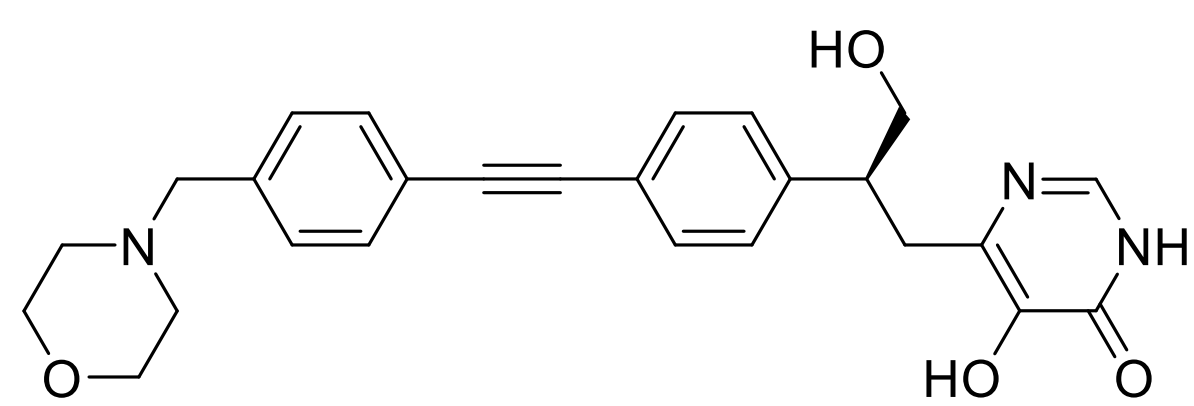

8b

Compound **8b** was generated from **50** utilizing General Procedure C (0.010 g, 15%). LC-MS (m/z): 446.3 $[M+H]^+$. ^1H NMR (DMSO-d_6): δ 12.43 (br s, 1H), 9.06 (br s, 1H), 7.70 (s, 1H), 7.50-7.41 (m, 4H), 7.35 (d, J = 8.08 Hz, 2H), 7.24 (d, J = 8.04 Hz, 2H), 4.76 (d, J = 5.44 Hz, 1H), 4.10-4.09 (m, 2H), 3.58-3.56 (m, 4H), 3.48 (s, 2H), 3.16 (d, J = 5.20 Hz, 1H), 2.72-2.66 (m, 2H), 2.50-2.49 (m, 4H)

Procedures for the preparation of Compound 8c

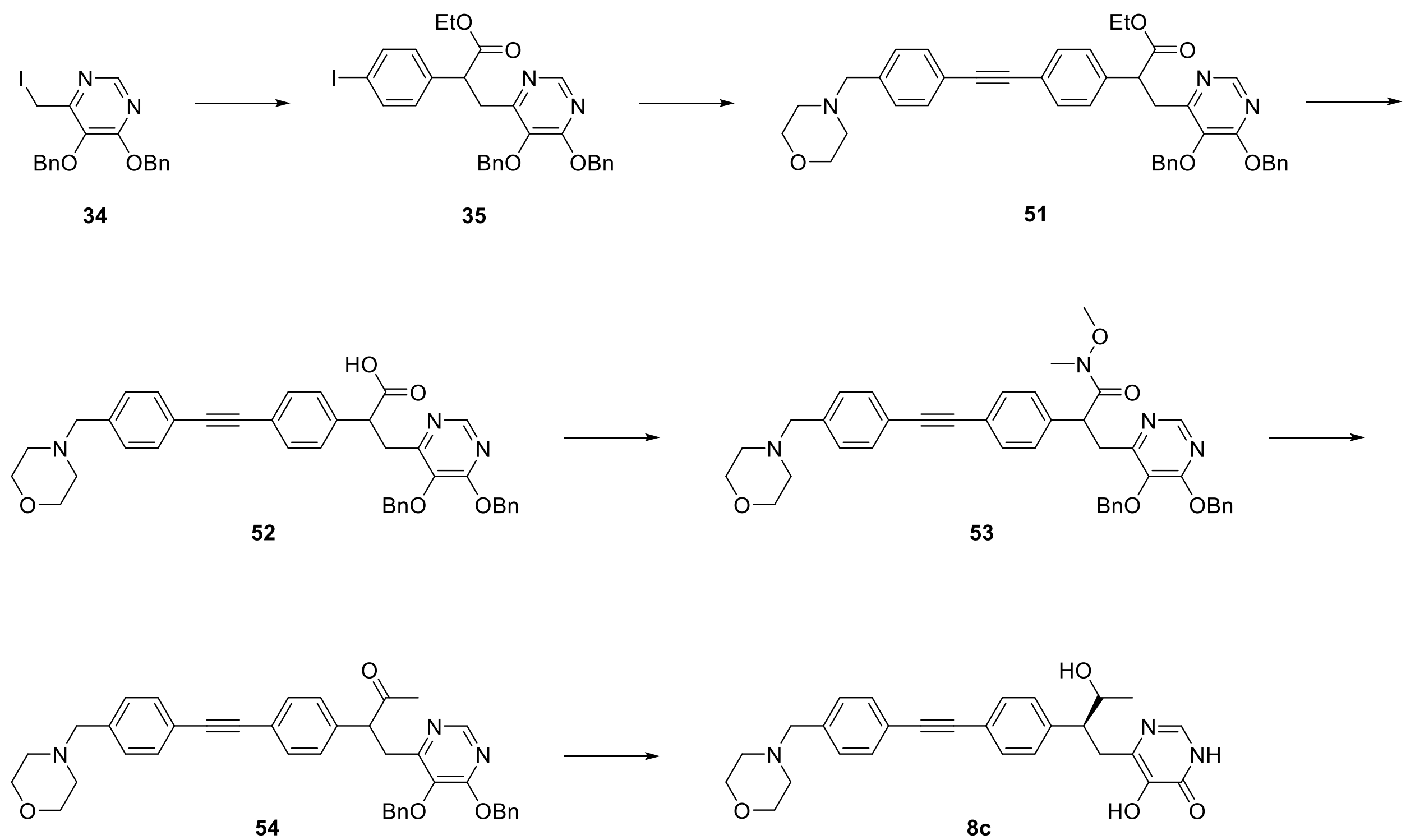

Ethy-3-[5,6-bis(benzyloxy)pyrimidin-4-yl]-2-(4-iodophenyl)propanoate (35):

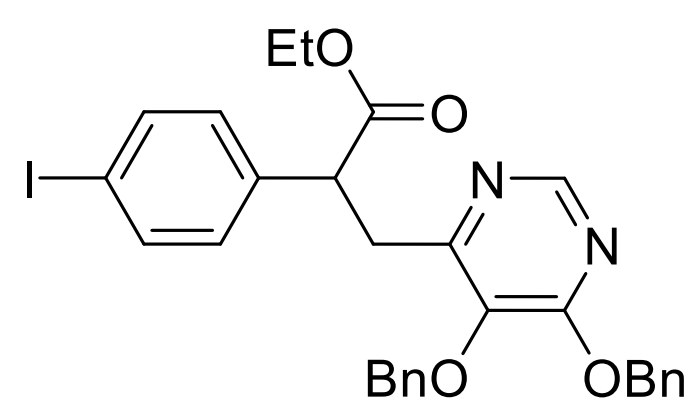

35

A solution of **34** (7.00 g, 0.0162 mol) and ethyl 2-(4-iodophenyl)acetate (5.38 g, 0.0186 mol) in DMF (30 mL) was cooled to 0 °C under nitrogen followed by the addition of NaH (60%, 0.714 g). The mixture was allowed to stir for 15 min at 0 °C and then allowed to warm to room temperature for an additional 3 h. The reaction was then quenched with ice and aqueous ammonium chloride then extracted with EtOAc. The combined organic layers were dried over Na_2SO_4, concentrated, and purified by silica gel chromatography (0-20% EtOAc in hexanes) to yield **35** (8.3 g, 87%). LC-MS (m/z): 595.2 $[M+H]^+$.

Ethyl-3-[5,6-bis(benzyloxy)pyrimidin-4-yl]-2-(4-{[4-(morpholinomethyl)phenyl]ethynyl}-phenyl)propanoate (51):

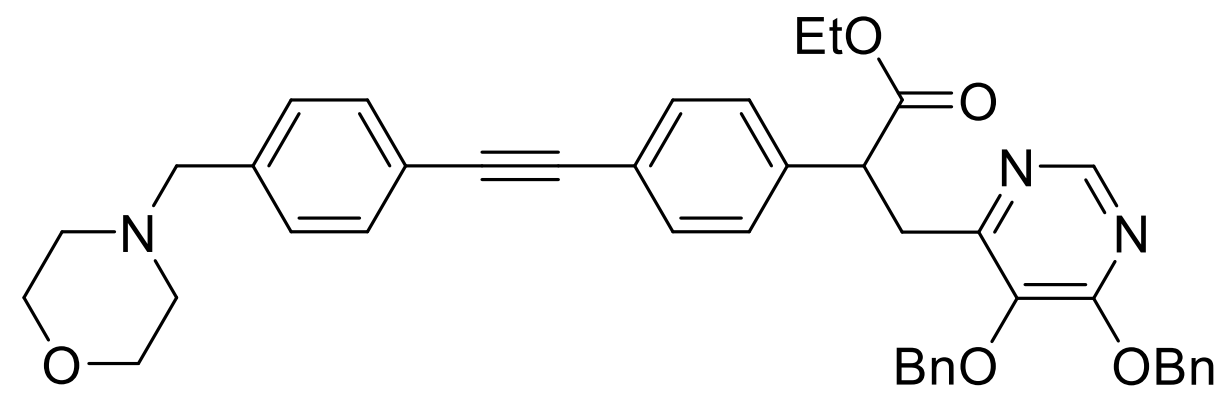

51

Compound **51** was generated from **30** and **35** utilizing General Procedure B (150 mg, 28%). LC-MS (m/z): 668.3 $[M+H]^+$.

3-[5,6-bis(benzyloxy)pyrimidin-4-yl]-2-(4-{[4-(morpholinomethyl)phenyl]ethynyl}phenyl)-propanoic acid (52):

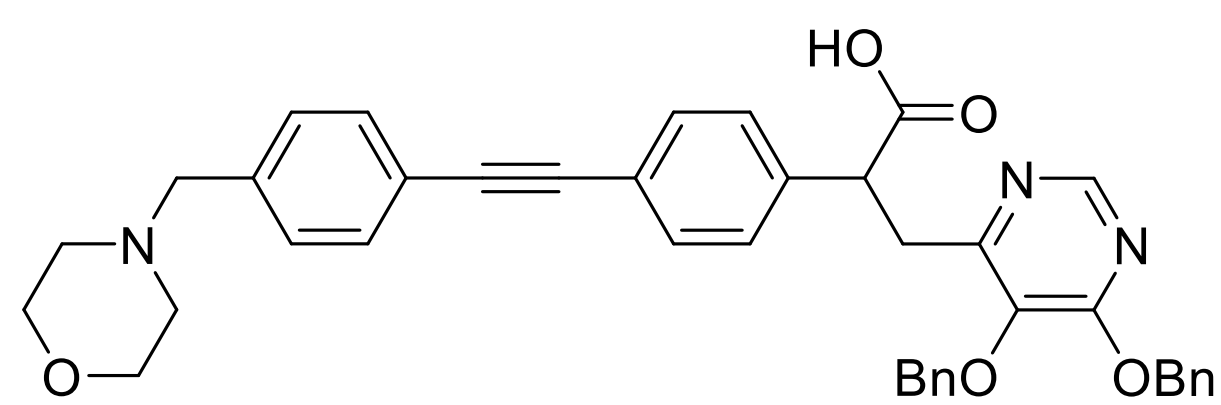

52

To a solution of **51** (200 mg, 0.336 mmol) in 1:1 EtOH:water (5 mL) NaOH (0.077 g, 1.94 mmol) was added and stirred at 25 °C for 2 h. The reaction mixture was then diluted with water and extracted with EtOAc. The aqueous layer was acidified with HCl and extracted with EtOAc, and the combined organic layers were washed with brine and dried over Na_2SO_4. Removal of the solvent under reduced pressure yielded **52** as a pure solid (110 mg, 59%). LC-MS (m/z): 639.2 $[M+H]^+$.

3-[5,6-bis(benzyloxy)pyrimidin-4-yl]-N-methoxy-N-methyl-2-(4-{[4-(morpholinomethyl)-phenyl]ethynyl}phenyl)propanamide (53):

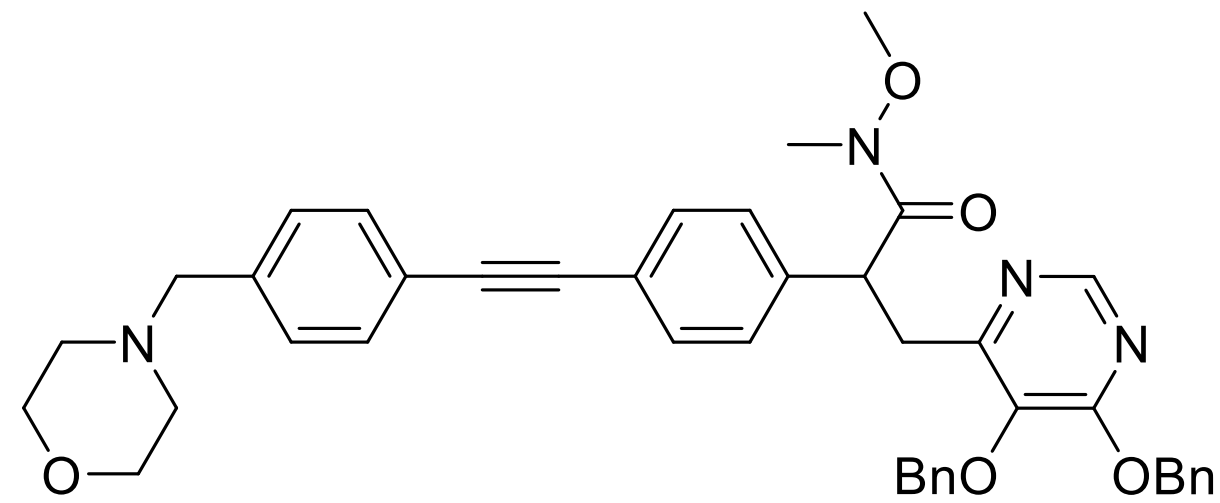

53

To a solution of **52** (500 mg, 0.78 mmol) in DCM (5 mL), *N*,*O*-dimethylhydroxylamine (150 mg, 1.56 mmol), HOBt (210 mg, 1.56 mmol) and DIPEA (0.60 mL, 3.3 mmol) were added and the mixture allowed to stir for 2 h. The reaction mixture was then washed with water, dried over Na_2SO_4, and the solvent removed under reduced pressure to yield **53** (430 mg, 80%). LC-MS (m/z): 683.3 $[M+H]^+$.

4-[5,6-bis(benzyloxy)pyrimidin-4-yl]-3-(4-{[4-(morpholinomethyl)phenyl]ethynyl}phenyl)-butan-2-one (54):

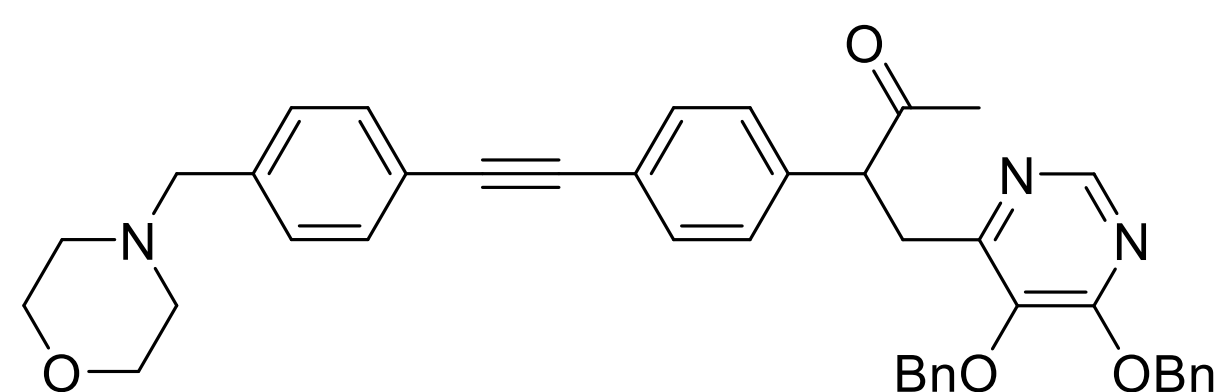

54

To a solution of **53** (426 mg, 0.624 mmol) in THF (mL) was cooled to -20 °C prior to the addition of methyl magnesium bromide (1.2 mL of a 1.5 M solution in diethyl ether, 1.9 mmol). The mixture was allowed to warm to 25 °C and stirred for 1 h before it was quenched with saturated aqueous NH_4Cl, diluted with water, and extracted with ethyl acetate. The combined organic layers were washed with brine, dried over Na_2SO_4, and the solvent removed under reduced pressure. The resulting residue was purified by silica gel chromatography to yield **54** (300 mg, 75%), LC-MS (m/z): 638.1 $[M+H]^+$.

5-hydroxy-6-[3-hydroxy-2-(4-{[4-(morpholinomethyl)phenyl]ethynyl}phenyl)butyl]-pyrimidin-4(3H)-one (8c):

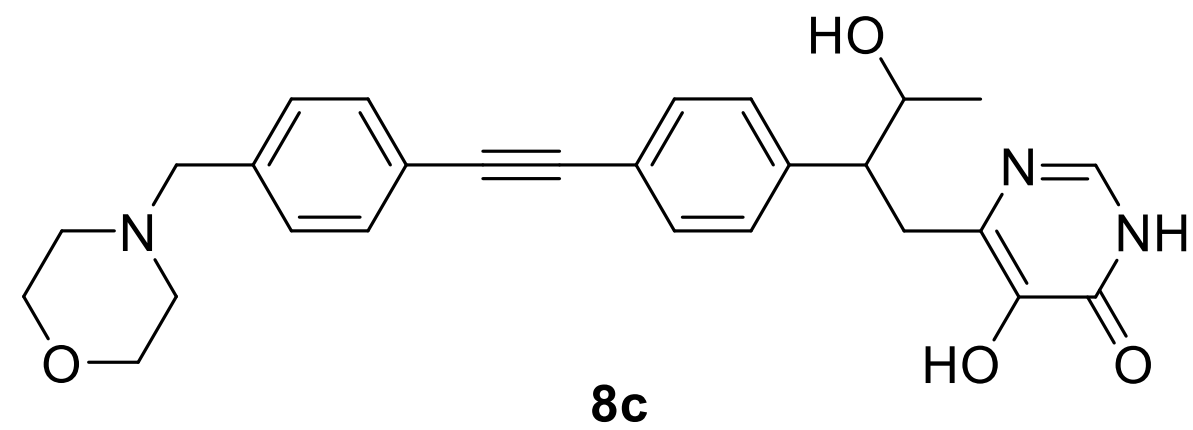

8c

To a solution of **54** (100 mg, 0.16 mmol) in MeOH (20 mL) was added sodium borohydride (35 mg, 0.94 mmol) and the mixture was stirred at 25 °C for 10 min. The reaction was then quenched with saturated aqueous NH_4Cl and the resulting mixture extracted with ethyl acetate. The combined organic layers were concentrated and the resulting residue used without further isolation to generate **8c** according to General Procedure C (13 mg, 18%). LC-MS (m/z): 460.0 $[M+H]^+$.

Procedures for the preparation of Compound 6d

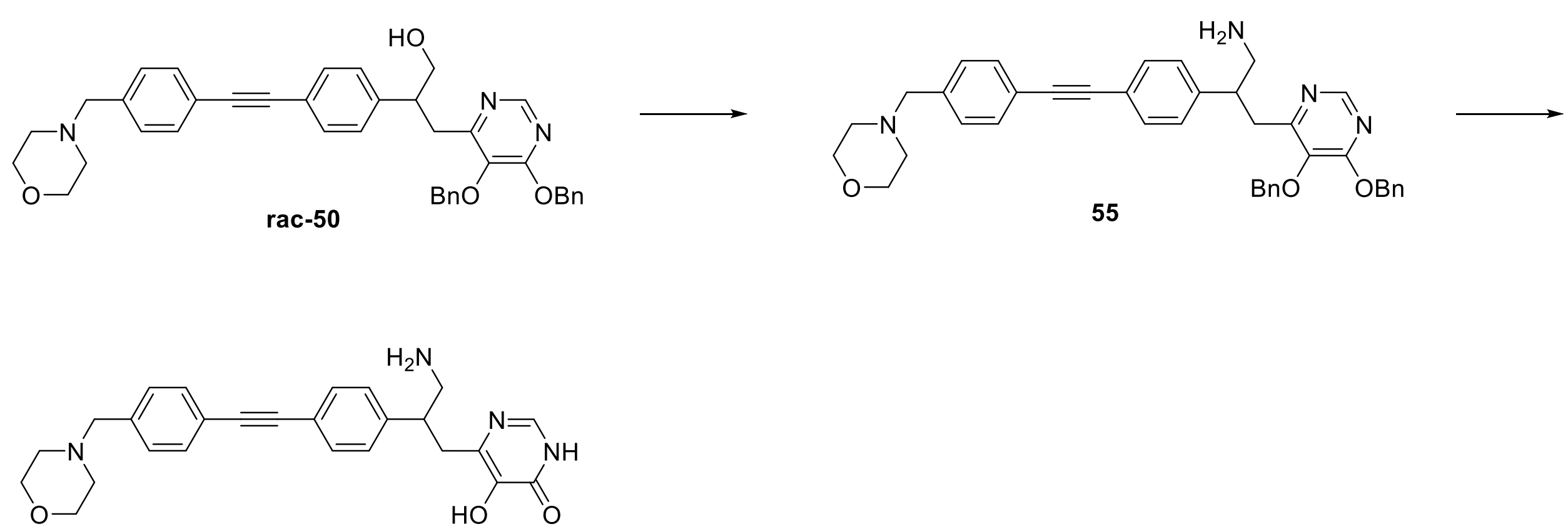

3-(5,6-bis{benzyloxy}pyrimidin-4-yl)-2-{4-[(4-(morpholinomethyl)phenyl]ethynyl}phenyl)propan-1-amine (55):

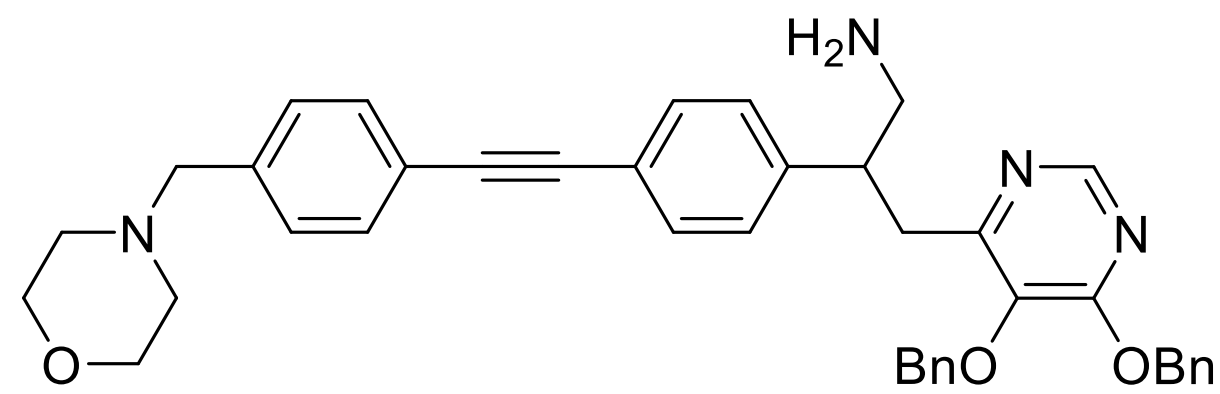

55

To a solution of **rac-50** (2.38 g, 0.0038 mol) in DCM (25 mL) at room temperature, Et_3N (1.1 mL, 0.0076 mol) and mesyl chloride (0.657 g, 0.00573 mol) was added and stirred for 1 h. After completion of the reaction, water was added and extracted with DCM and the organic layer was washed with water and brine solution and dried over Na_2SO_4, filtered and concentrated under reduced pressure. The crude mesylate (3.40 g, 0.00483 mol) was then taken up in DMF (10 mL) and NaN_3 (1.57 g, 0.0241 mol) was added and the mixture heated at 100 °C for 6 h. After completion of the reaction, water was added and the mixture extracted with EtOAc. The organic layer was washed with water, dried over Na_2SO_4. The resulting crude was taken up in a mixture of THF (45 mL) and H_2O (15 mL), PPh_3 (3.66 g, 0.0139 mol) was added and the reaction was heated at 85 °C for 2 h. After completion of the reaction, the reaction mixture was concentrated and the crude product was purified by column chromatography on silica gel (20-30% MeOH in DCM) to afford **55** (1.97 g, 83%). LC-MS (m/z): 625.3 $[M+H]^+$.

6-[3-amino-2-(4-{[4-(morpholinomethyl)phenyl]ethynyl}phenyl)propyl]-5-hydroxypyrimidin-4(3H)-one (8d):

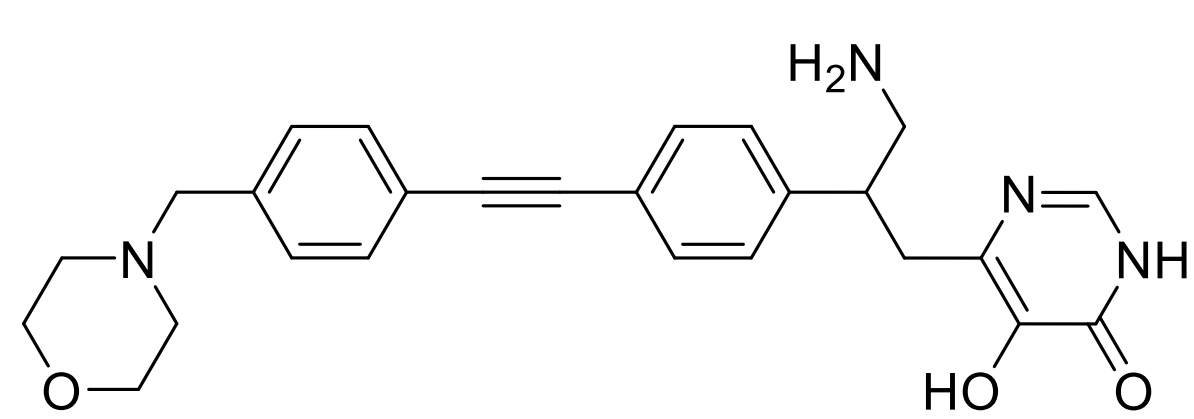

8d

Compound **8d** was prepared from **55** utilizing General Procedure C (78 mg, 54%). LC-MS (m/z): 445.2 $[M+H]^+$.

Procedures for the preparation of Compound 8e

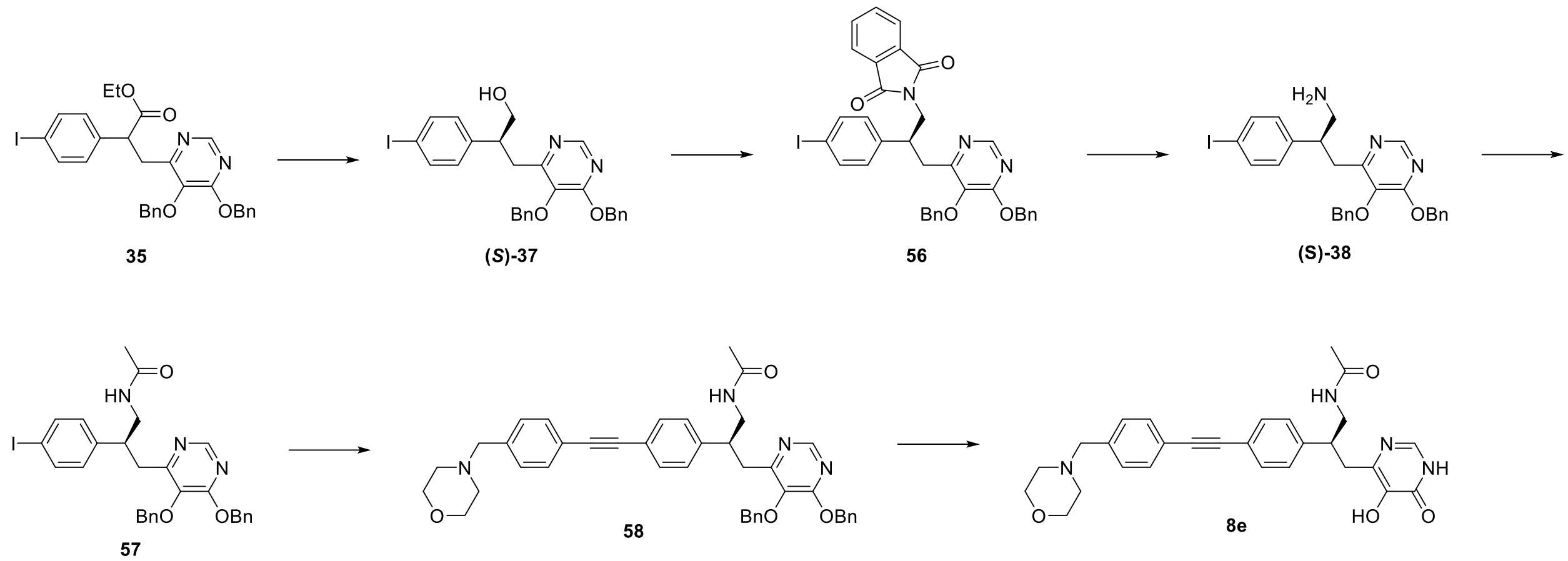

3-(5,6-bis(benzyloxy)pyrimidin-4-yl)-2-(4-iodophenyl)propan-1-ol (37):

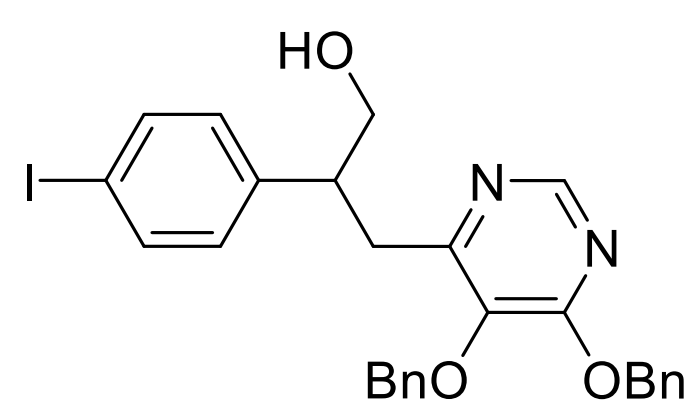

37

To a solution of **35** (2.33 g, 0.00393 mol) in THF (10 mL) was added $LiBH_4$ (138 mg, 0.00629 mol) followed by EtOH (20 mL) and the mixture stirred at room temperature for 16 h. The mixture was then diluted with water and extracted with EtOAc. The combined organic layers were concentrated and purified by silica gel chromatography to yield **37** (1.54 g, 71%). LC-MS (m/z): 553.3 $[M+H]^+$. **(*S*)-37** and **(*R*)-37** were isolated by SFC for use as starting materials to generate enantiomerically-pure final compounds.

2-{(*S*)-3-[5,6-bis(benzyloxy)pyrimidin-4-yl]-2-[4-iodophenyl]propyl}isoindoline-1,3-dione (56):

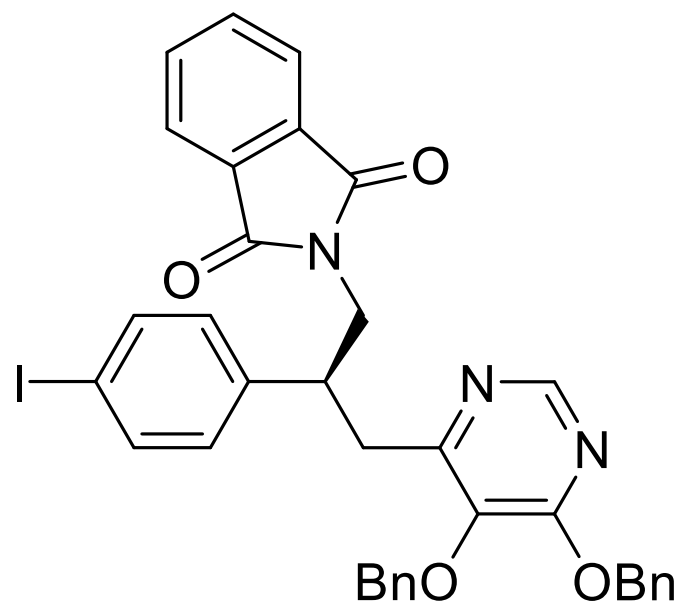

56

To a solution of ***(S*)-37** (3.2 g, 5.8 mmol) in dry THF (75 mL), phthalimide (1.27 g, 8.69 mmol) and (2.27 g, 8.69 mmol) were added and stirred for 10 min. Then, DEAD (1.29 g, 6.96 mmol) was added in one portion and the reaction stirred for 3 h. After completion of the reaction, the mass was dissolved in water and extracted with EtOAc (2 x 100 mL), the combined organic layers were washed with brine solution and organic layer was dried over Na_2SO_4 and concentrated. The crude product was purified by column chromatography on silica gel (20-35% EtOAc in pet. ether) to yield **56** (3.1 g, 79%). LC-MS (m/z): 682.1 $[M+H]^+$.

(*S*)-3-(5,6-bis(benzyloxy)pyrimidin-4-yl)-2-(4-iodophenyl)propan-1-amine ((*S*)-38):

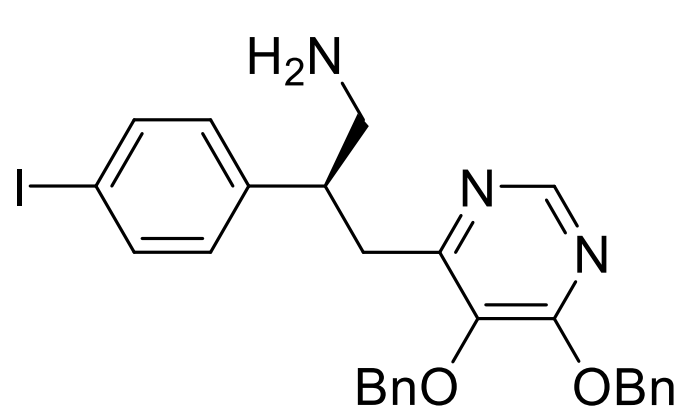

(S)-38

To a solution of **56** (3.0 g, 4.4 mol) in ethanol (30 mL), N_2H_4 hydrate (0.66 g, 13 mmol) was added and heated the reaction mass at 75 °C for 1 h. After completion of the reaction, reaction mass was filtered and solid obtained was washed with excess ethanol. The filtrate was concentrated under reduced pressure yield **(*S*)-38**. (2.2 g, 91%). LC-MS (m/z): 552.0 $[M+H]^+$. Early in the program, this intermediate was also isolated as a racement, **rac-38**.

***N*-{3-[5,6-bis(benzyloxy)pyrimidin-4-yl]-2-[4-iodophenyl]propyl}acetamide (57):**

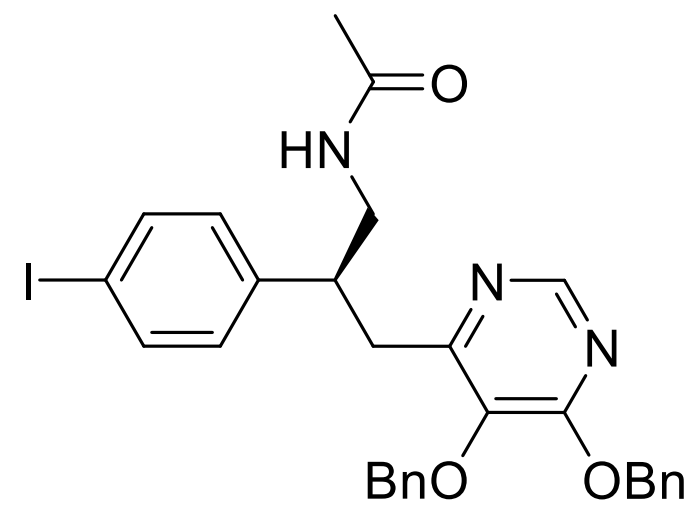

57

To a solution of **38** (0.50 g, 0.91 mmol) and Et_3N (0.20 mL, 1.4 mmol) in DCM (5 mL), was added a solution of acetyl chloride (0.12 mL, 1.1 mmol) in DCM (1 mL) dropwise at 0 °C and stirred at same temperature for 30 min. After completion of the reaction, the reaction mixture was diluted with DCM (5 mL) and washed with water (10 mL). The organic layer was dried over anhydrous Na_2SO_4 and concentrated under reduced pressure. The crude product was purified silica gel chromatography (80 - 100% EtOAc in pet. ether) to yield **57** (0.35 g, 65%). LC-MS (m/z): 594.2 $[M+H]^+$.

***N*-[3-(5,6-bis{benzyloxy}pyrimidin-4-yl)-2-(4-{[4-(morpholinomethyl)phenyl]ethynyl}-phenyl)propyl]acetamide (58):**

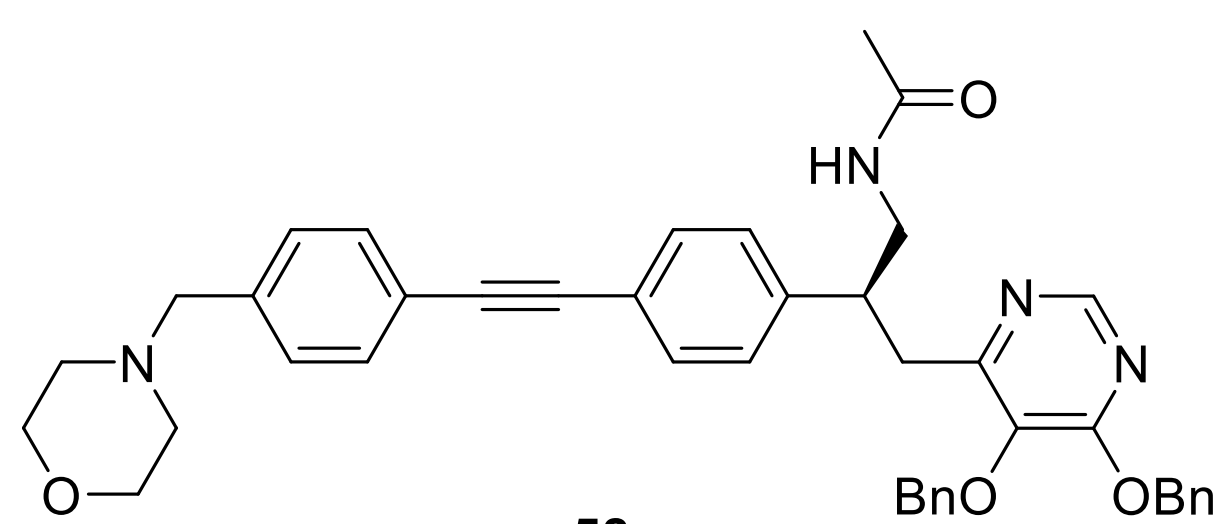

58

Compound **58** was prepared from **30** and **57** utilizing General Procedure B (250 mg, 64%). LC-MS (m/z): 667.3 $[M+H]^+$.

***N*-[3-(5-hydroxy-6-oxo-1,6-dihydropyrimidin-4-yl)-2-(4-{[4-(morpholinomethyl)phenyl]-ethynyl}phenyl)propyl]acetamide (8e):**

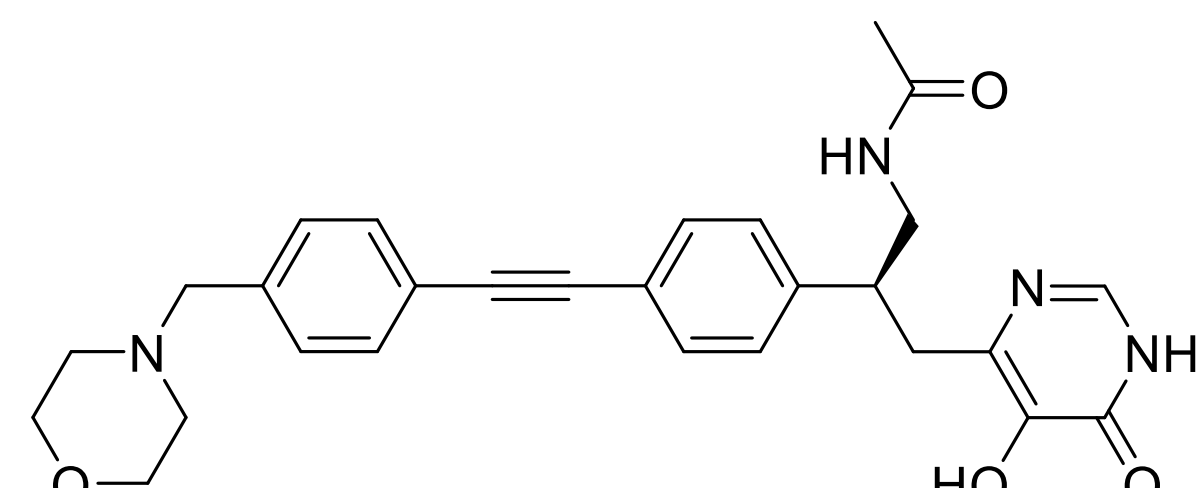

8e

Compound **8e** was prepared from **58** utilizing General Procedure D (120 mg, 66%). LC-MS (m/z): 487.2 $[M+H]^+$. ^{1}H NMR (DMSO-d_6): δ 9.07 (s, 1H), 7.86-7.83 (m, 1H), 7.65-7.50 (m, 5H), 7.41 (*d*, J = 8.0 Hz, 2H), 7.21 (*d*, J = 8.0 Hz, 2H), 3.59-3.44 (m, 5H), 3.31-3.27 (m, 4H), 2.78-2.68 (m, 2H), 2.36-2.34 (m, 5H), 1.73 (s, 3H).

Procedures for the preparation of Compound 8f

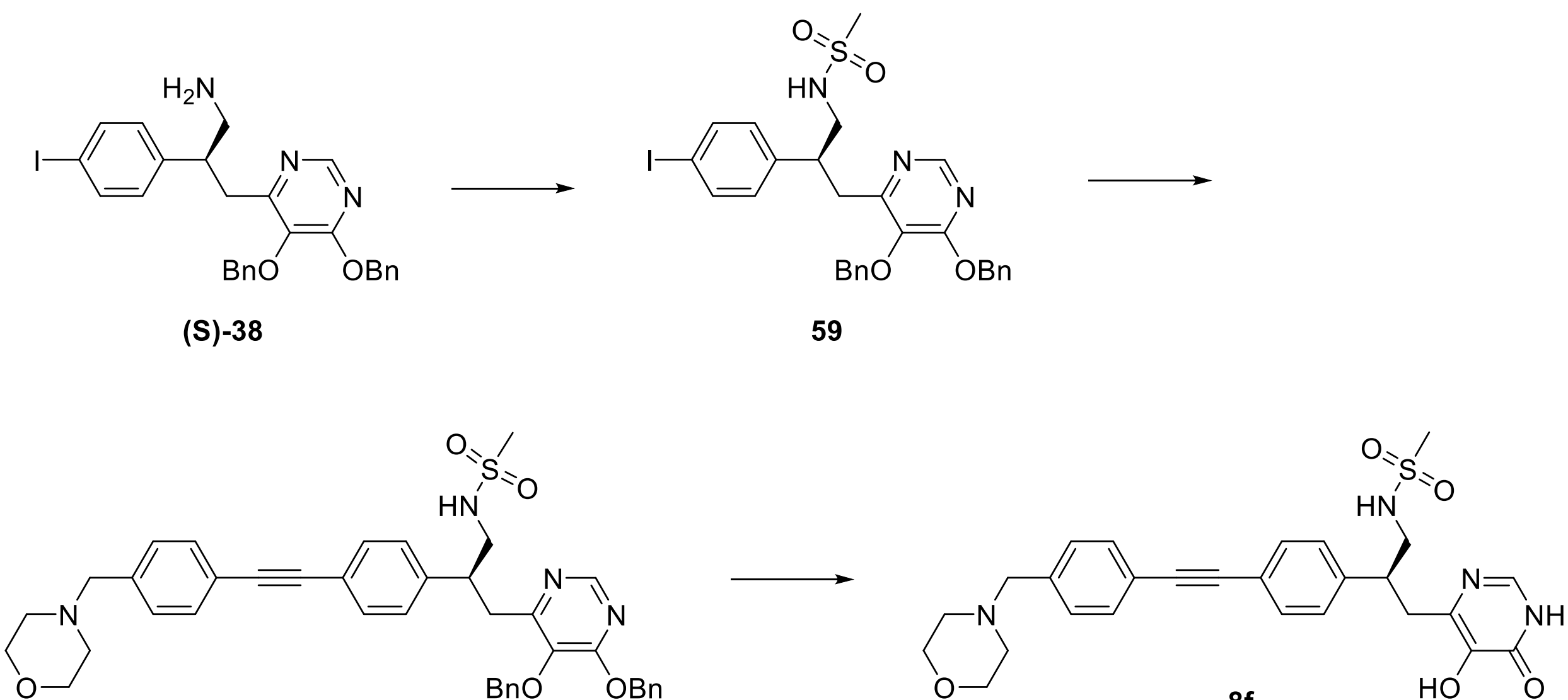

(S)-38 **59** **60** **8f**

(*S*)-*N*-{3-[5,6-bis(benzyloxy)pyrimidin-4-yl]-2-[4-iodophenyl]propyl}methanesulfonamide (59):

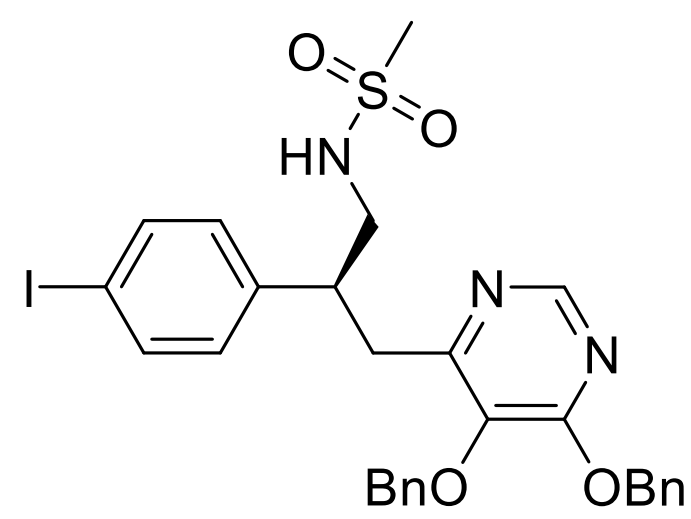

59

To a solution of **(S)-38** (1.8 g, 3.2 mmol) and Et_3N (1.3 mL, 3.5 mmol) in DCM (16 mL), was added a solution of methanesulfonyl chloride (0.27 mL, 9.6 mmol) in DCM (1 mL) dropwise at 0 °C and stirred at same temperature for 30 min. After completion of the reaction, the reaction mixture was diluted with DCM (50 mL) and washed with water (20 mL). The organic layer was dried over anhydrous Na_2SO_4 and concentrated under reduced pressure. The crude product was purified silica gel chromatography (80 - 100% EtOAc in pet. ether) to yield **59** (0.33 g, 17%). LC-MS (m/z): 630.2

(S)-N-[3-(5,6-bis{benzyloxy}pyrimidin-4-yl)-2-(4-{[4-(morpholinomethyl)phenyl]ethynyl}-phenyl)propyl]methanesulfonamide (60):

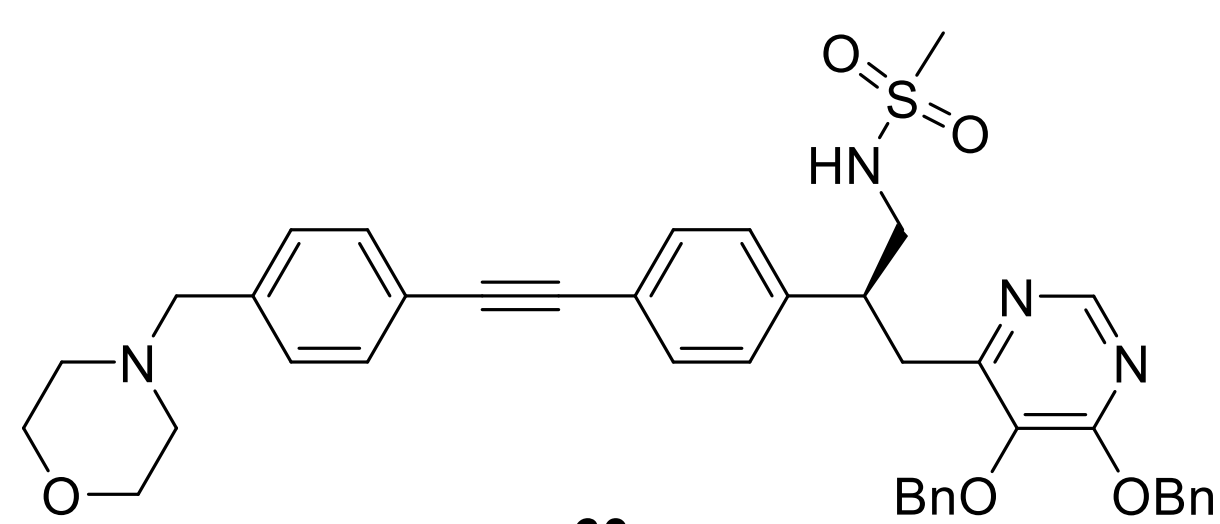

60

Compound **60** was prepared from **30** and **59** utilizing General Procedure B (300 mg, 81%). LC-MS (m/z): 703.2 $[M+H]^+$.

(S)-N-[3-(5-hydroxy-6-oxo-1,6-dihydropyrimidin-4-yl)-2-(4-{[4-(morpholinomethyl)-phenyl]ethynyl}phenyl)propyl]methanesulfonamide (8f):

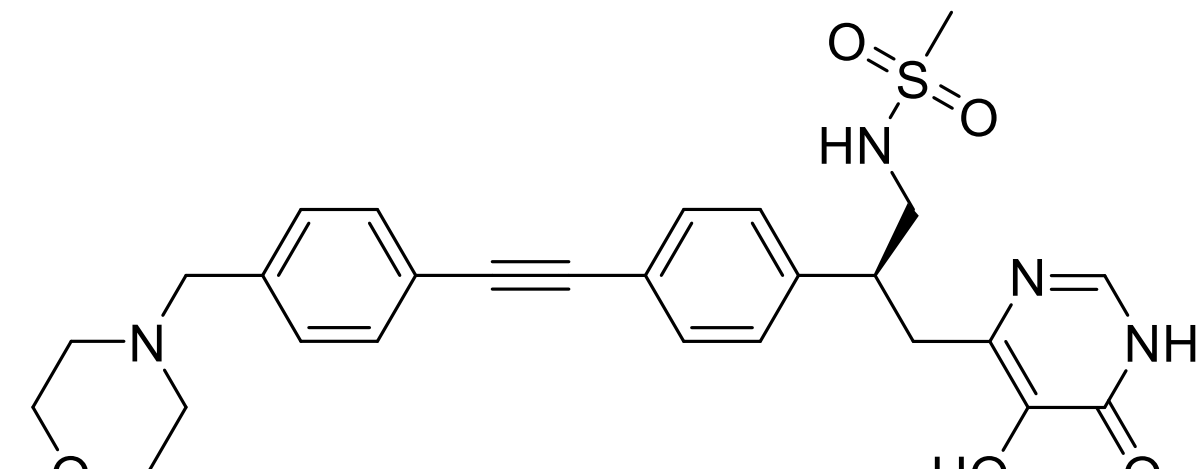

8f

Compound **8f** was prepared from **60** utilizing General Procedure D (150 mg, 68%). LC-MS (m/z): 523.2 $[M+H]^+$. ^{1}H-NMR (400 MHz, DMSO-d_6): δ 7.70 (s, 1H), 7.59 (d, J = 7.4 Hz, 2H), 7.50-7.49 (m, 2H), 7.45 (d, J = 8.16 Hz, 2H), 7.31 (d, J = 8.12 Hz, 2H), 4.13 (s, 2H), 3.85-3.75 (m, 5H), 3.40-3.39 (m, 6H), 3.06-3.01 (m, 5H), 2.81 (s, 3H).

Procedures for the preparation of Compound 8g

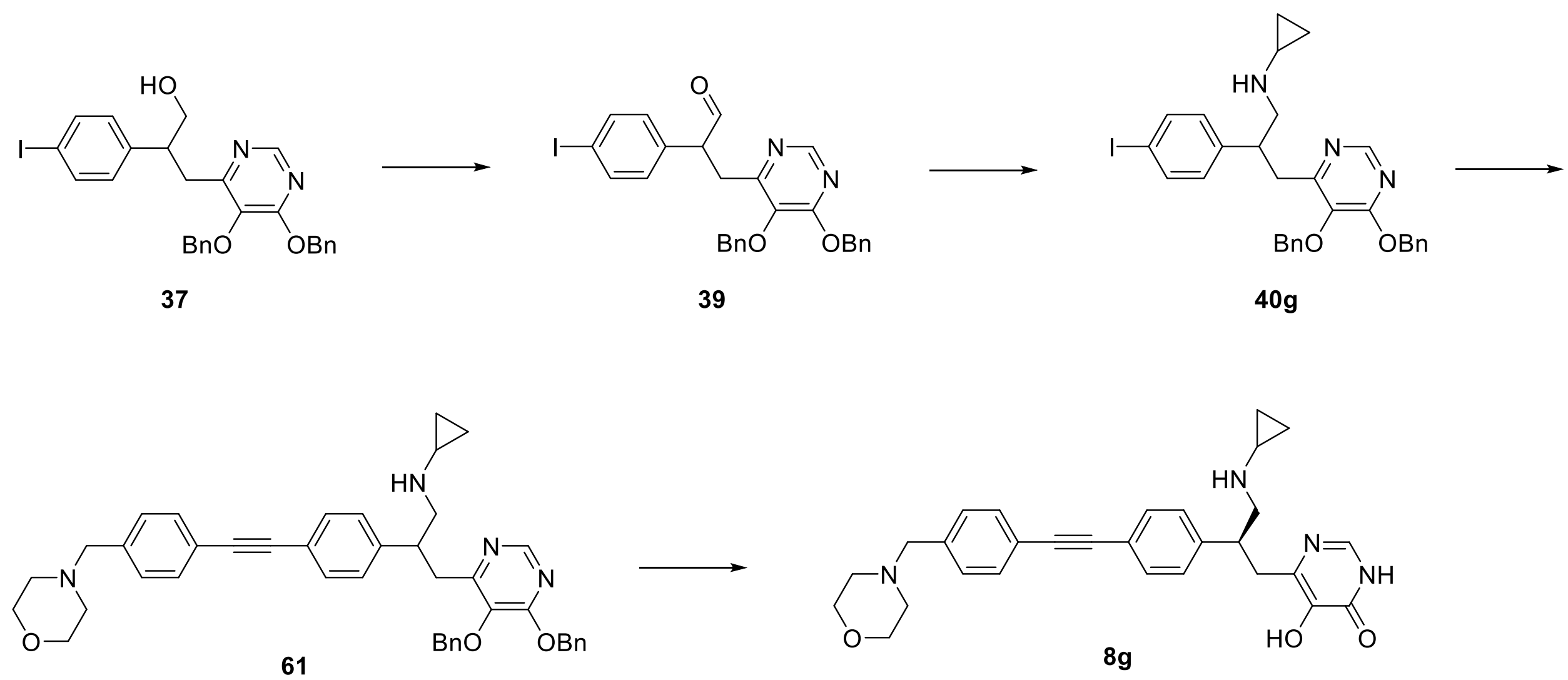

3-[5,6-bis(benzyloxy)pyrimidin-4-yl]-2-(4-iodophenyl)propanal (39):

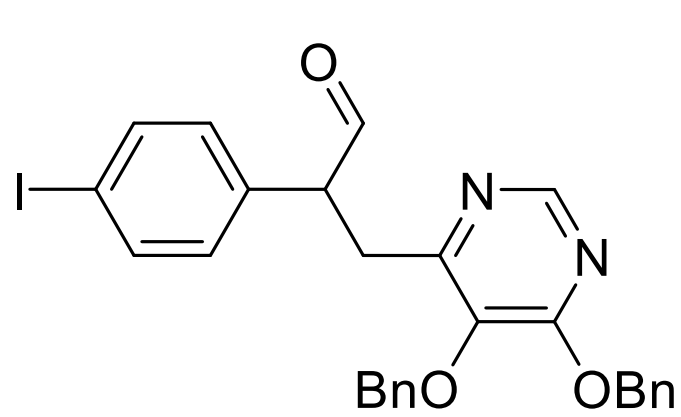

39

To a solution of **37** (1.00 g, 1.81 mmol) in DCM (10 mL) at 0 °C, Dess-Martin periodinane (1.25 g, 2.95 mmol) was slowly added over 20 min. The solution was then allowed to warm to room temperature and stir for 90 min. The mixture was then washed with water and saturated $NaHCO_3$, dried over Na_2SO_4, and concentrated. The resulting residue was purified by silica gel chromatography (0-100% EtOAc/hexanes) to yield **39** (0.70 g, 70%). LC-MS (m/z): 551 $[M+H]^+$.

***N*-{3-[5,6-bis(benzyloxy)pyrimidin-4-yl]-2-[4-iodophenyl]propyl}cyclopropanamine (40g):**

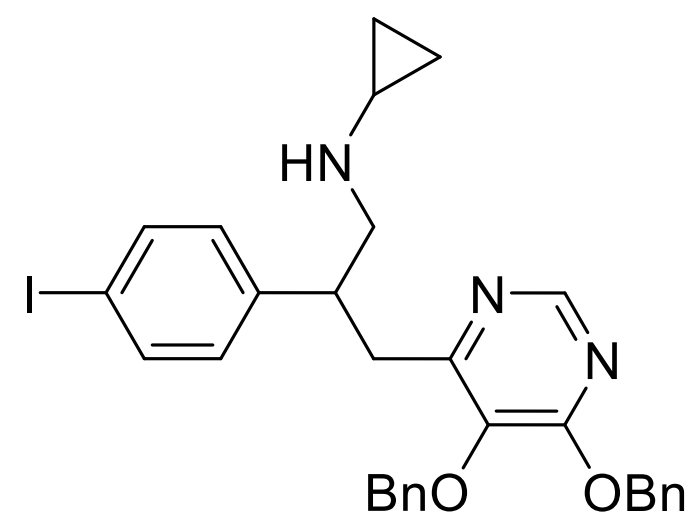

40g

Compound **40g** was prepared from **39** and cyclopropylamine utilizing General Procedure F (400 mg, 19%). LC-MS (m/z): 592.2 $[M+H]^+$.

***N*-[3-(5,6-bis{benzyloxy}pyrimidin-4-yl)-2-(4-{[4-(morpholinomethyl)phenyl]ethynyl}-phenyl)propyl]cyclopropanamine (61):**

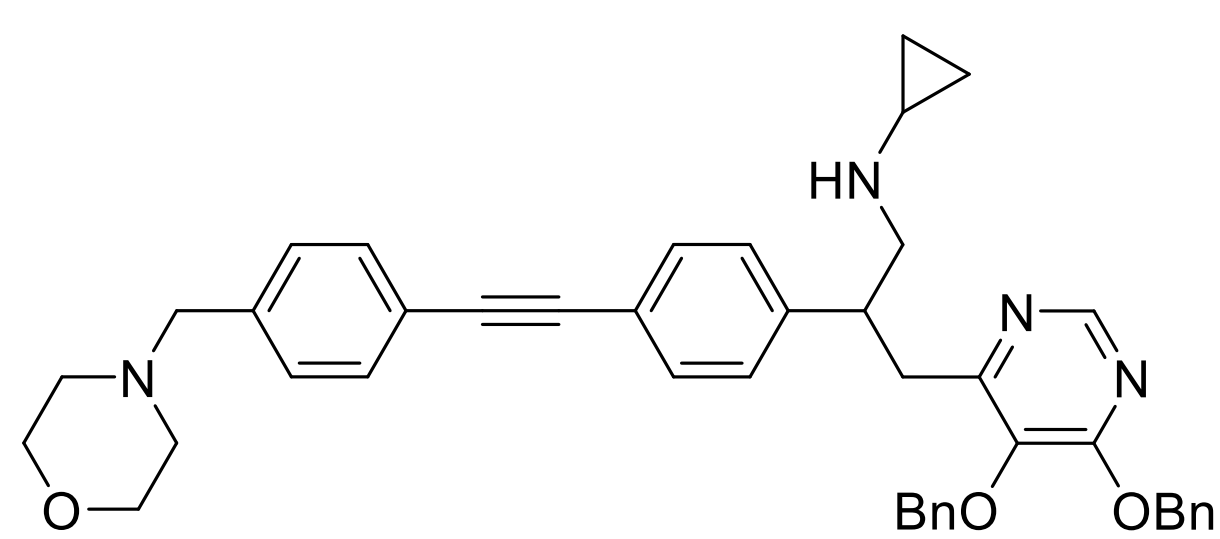

61

Compound **61** was prepared from **30** and **40g** utilizing General Procedure B (350 mg, crude). LC-MS (m/z): 665.3 $[M+H]^+$.

(*S*)-6-[3-(cyclopropylamino)-2-(4-{[4-(morpholinomethyl)phenyl]ethynyl}phenyl)propyl]-5-hydroxypyrimidin-4(3H)-one (8g):

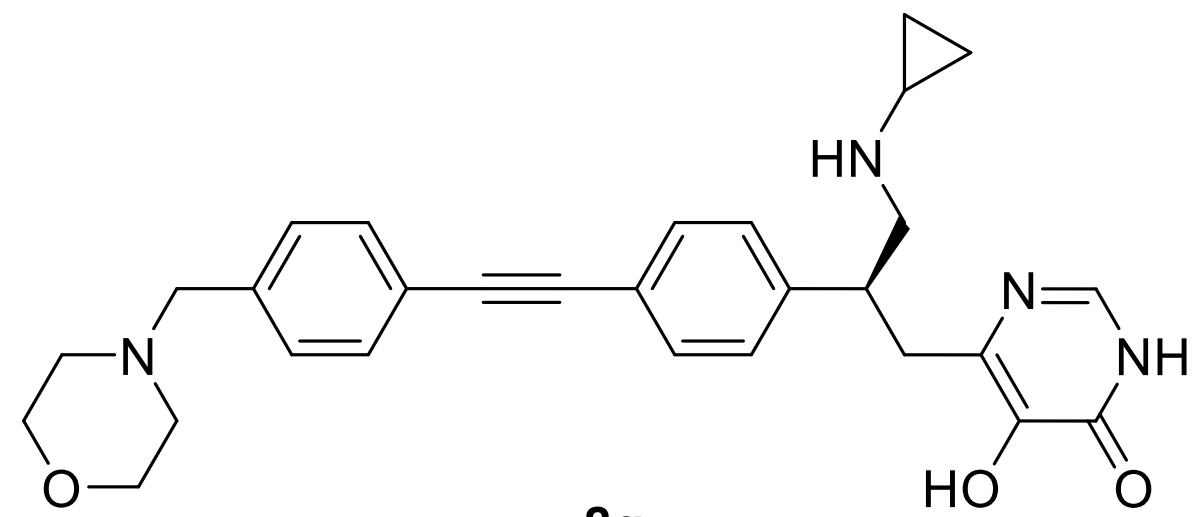

8g

Racemic **8g** was prepared from crude **61** utilizing General Procedure D (85 mg, 26% over two steps), which was then subjected to SFC to yield **8g**. LC-MS (m/z): 485.2 $[M+H]^+$. ^1H NMR (DMSO-d_6): δ 12.47 (br s, 1H), 9.21 (br s, 1H), 8.50 (s, 1H), 7.69 (s, 1H), 7.48-7.31 (m, 8H), 3.60-3.40 (m, 5H), 3.40-3.20 (m, 4H), 3.00-2.80 (m, 2H), 2.70-2.60 (m, 2H), 2.30-2.20 (m, 3H), 0.74-0.70 (m, 4H).

Procedures for the preparation of Compound 8h

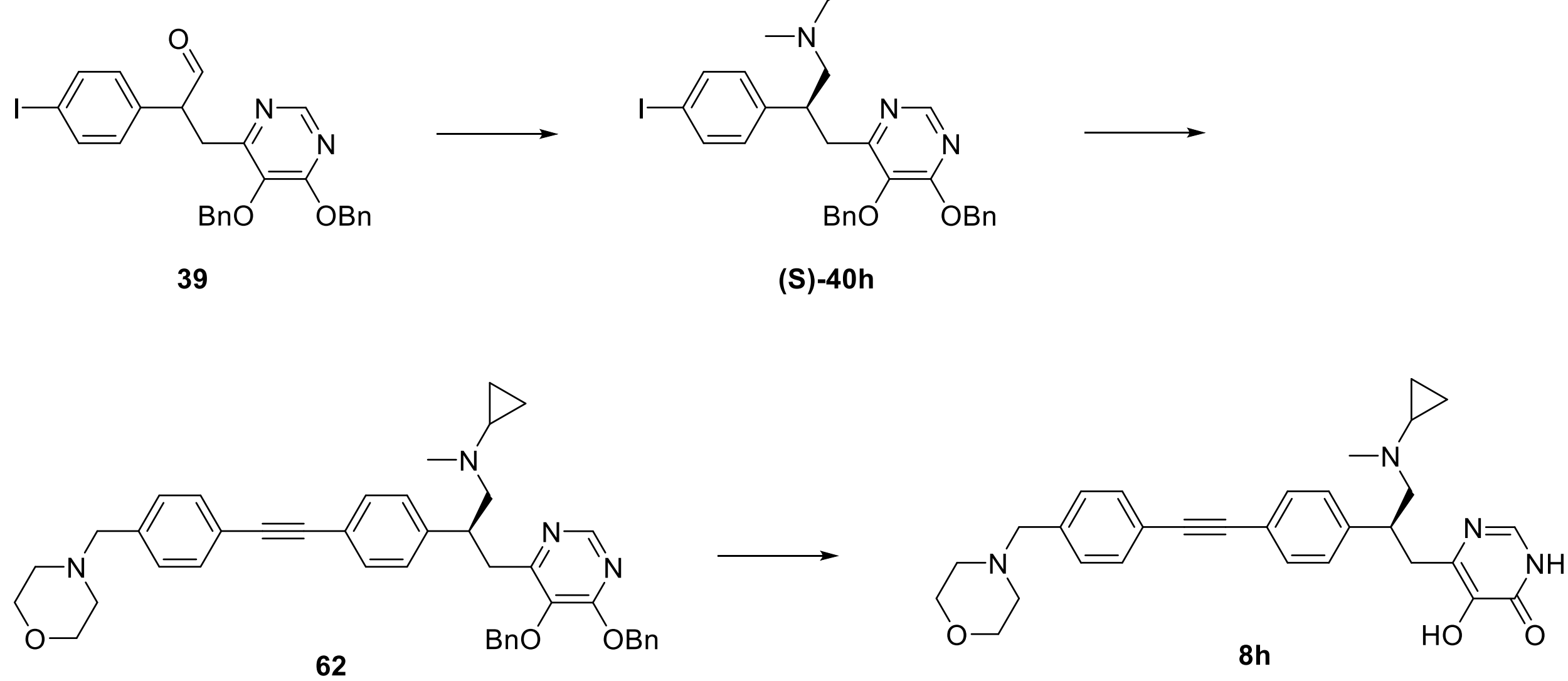

39 **(S)-40h** **62** **8h**

***N*-{3-[5,6-bis(benzyloxy)pyrimidin-4-yl]-2-[4-iodophenyl]propyl}-N-methylcyclopropanamine (40h):**

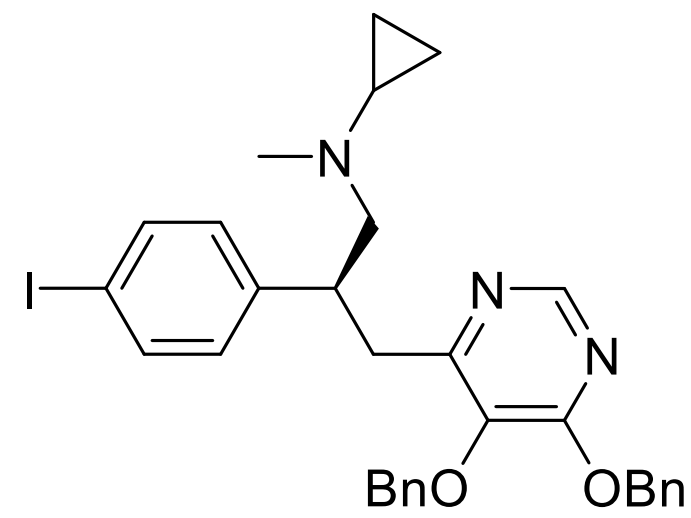

(S)-40h

Compound **(*S*)-40h** was prepared from **39** and *N*-methylcyclopropanamine hydrochloride utilizing General Procedure F (400 mg, 19%). LC-MS (m/z): 592.2 $[M+H]^+$. The resulting racemate was separated by SFC before proceeding to the next step.

(*S*)-N-[3-(5,6-bis{benzyloxy}pyrimidin-4-yl)-2-(4-{[4-(morpholinomethyl)phenyl]ethynyl}-phenyl)propyl]-N-methylcyclopropanamine (62):

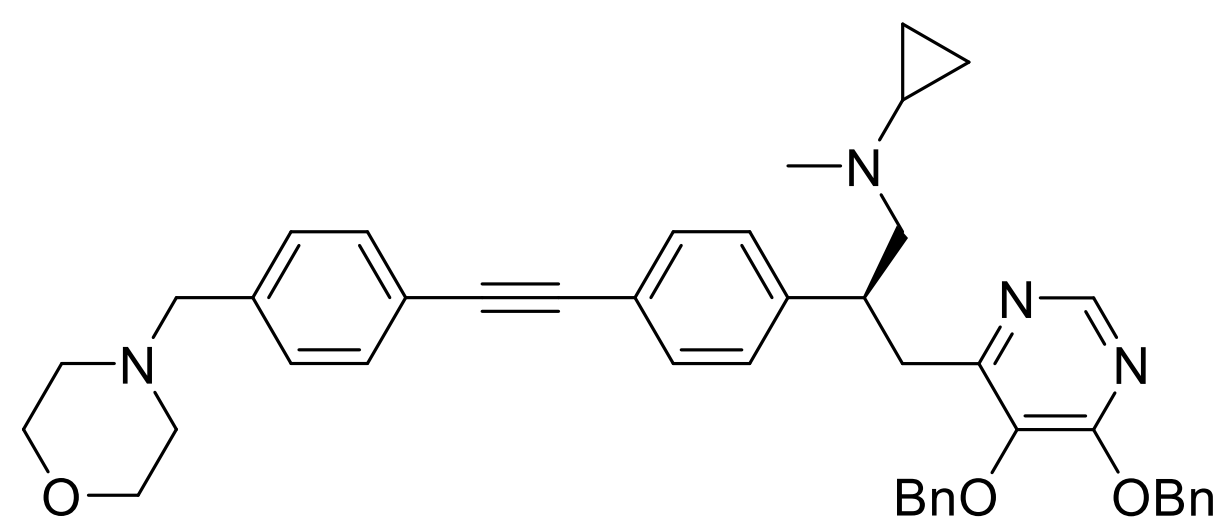

62

Compound **62** was prepared from **30** and **(*S*)-40h** utilizing General Procedure B (420 mg, 75%). LC-MS (m/z): 678.3 $[M+H]^+$.

(*S*)-6-[3-(cyclopropyl{methyl}amino)-2-(4-{[4-(morpholinomethyl)phenyl]ethynyl}phenyl)-propyl]-5-hydroxypyrimidin-4(3H)-one (8h):

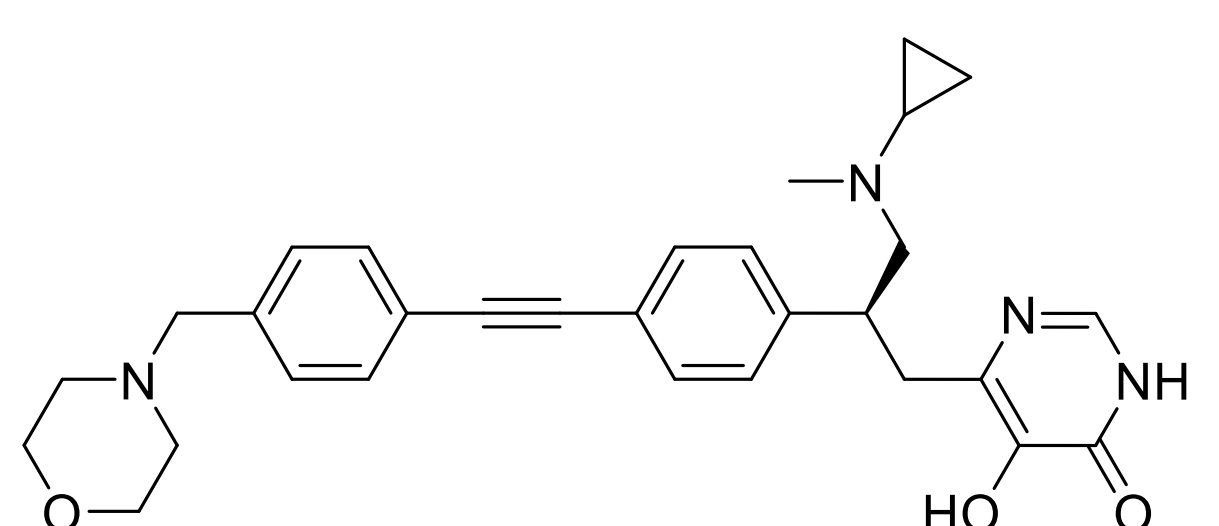

8h

Compound **8h** was prepared from **62** utilizing General Procedure D (200 mg, 65%). LC-MS (m/z): 499.2 $[M+H]^+$. ^1H NMR (DMSO-d_6): δ 8.24 (s, 1H), 7.64 (s, 1H), 7.47 (d, J = 7.6 Hz, 1H), 7.38-7.33 (m, 4H), 7.18 (d, J = 7.60 Hz, 1H), 3.57-3.54 (m, 5H), 3.48 (s, 2H), 3.16 (s, 1H), 2.80-2.68 (m, 5H), 2.40-2.35 (m, 5H), 2.22 (s, 3H), 1.61-1.56 (m, 1H), 0.36-0.35 (m, 2H), 0.17-0.15 (m, 2H).

Procedures for the preparation of Compound 8i

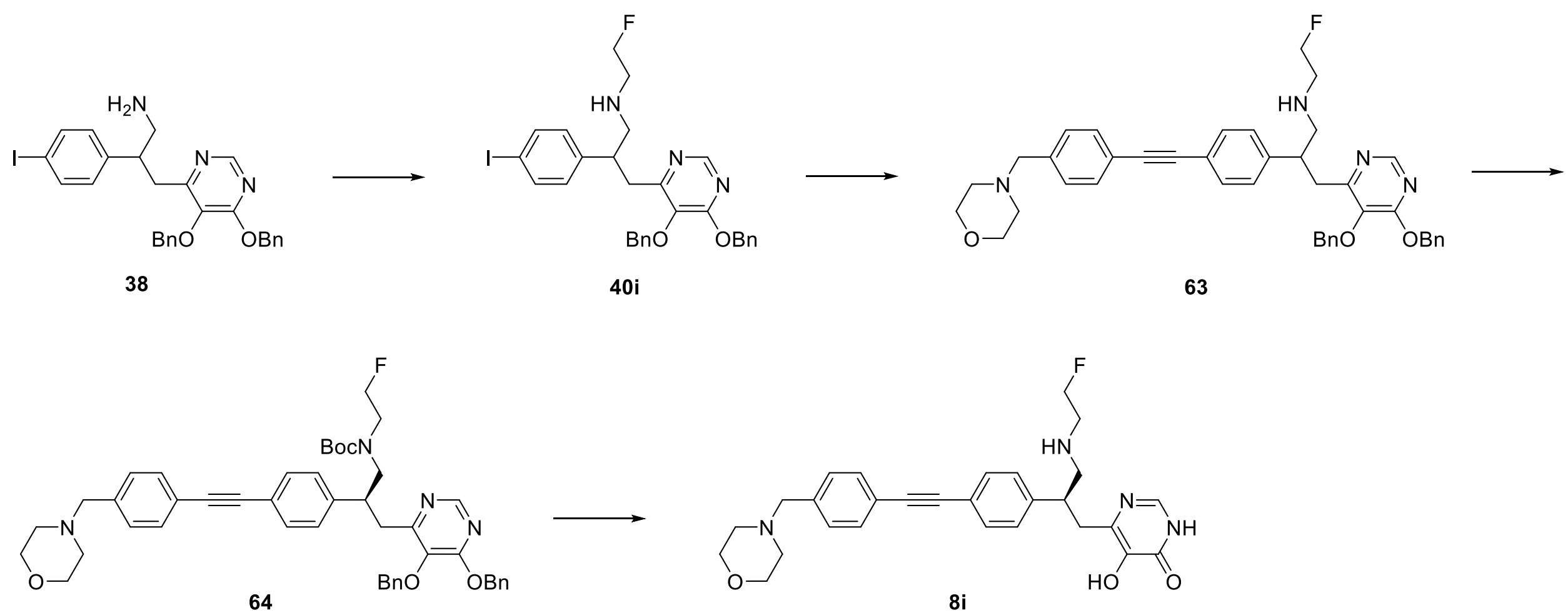

3-[5,6-bis(benzyloxy)pyrimidin-4-yl]-*N*-(2-fluoroethyl)-2-(4-iodophenyl)propan-1-amine (40i):

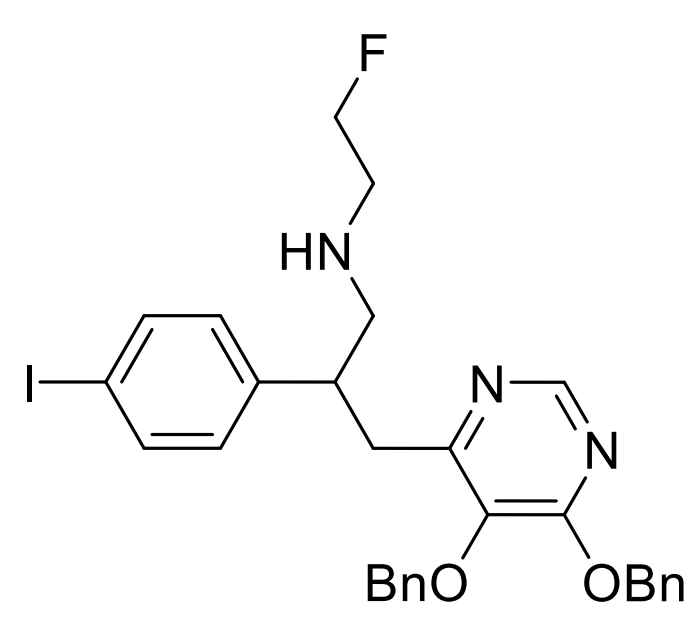

40i

To a solution of **38** (2.2 g, 0.0039 mol) in 1,4-dioxane (50 mL) was added potassium carbonate (1.37 g, 0.0100 mol), 1-bromo-2-fluoroethane (0.79 g, 0.0079 mol) and CuI (76 mg, 0.10 eq) and the reaction mixture was heated at 100 °C for 12 h. The mixture was then diluted with water and

extracted with EtOAc. The combined organic layers were washed with brine, dried over Na_2SO_4, filtered and concentrated. The residue was purified silica gel silica chromatography (10% MeOH in DCM) to afford **40i** (0.85 g, 36%). LC-MS (m/z): 598.1 $[M+H]^+$.

3-[5,6-bis(benzyloxy)pyrimidin-4-yl]-N-(2-fluoroethyl)-2-(4-{[4-(morpholinomethyl)-phenyl]ethynyl}phenyl)propan-1-amine (63):

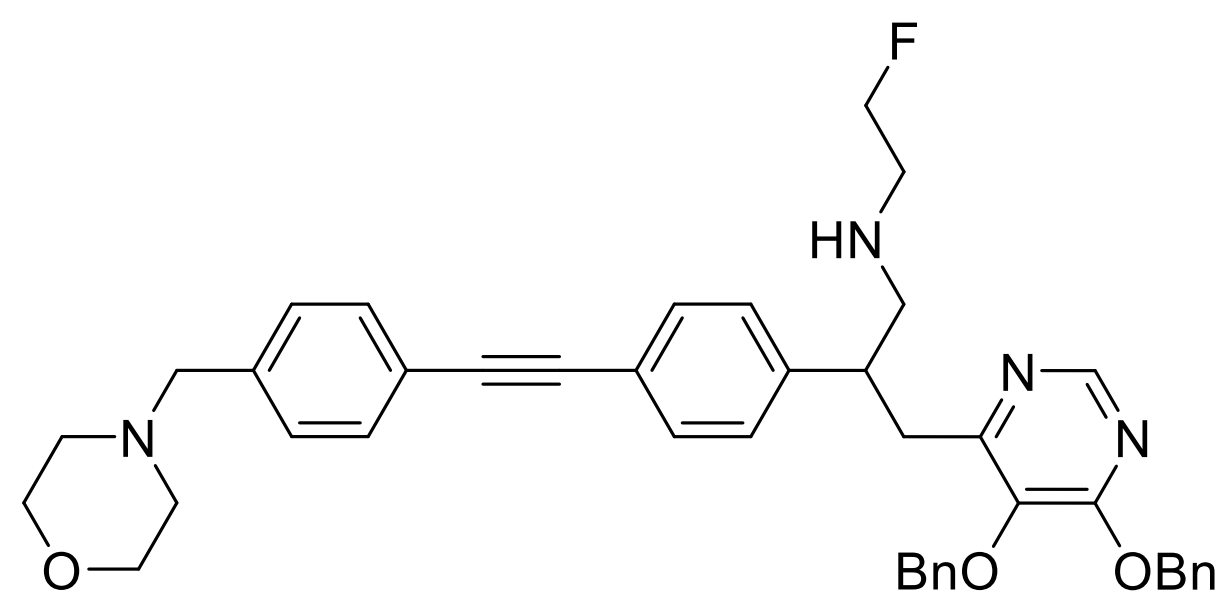

63

Compound **63** was prepared from **30** and **40i** utilizing General Procedure B (920 mg, crude). LC-MS (m/z): 671.3 $[M+H]^+$.

tert-butyl-[(*S*)-3-(5,6-bis{benzyloxy}pyrimidin-4-yl)-2-(4-{[4-(morpholinomethyl)phenyl]-ethynyl}phenyl)propyl]-(2-fluoroethyl)carbamate (64):

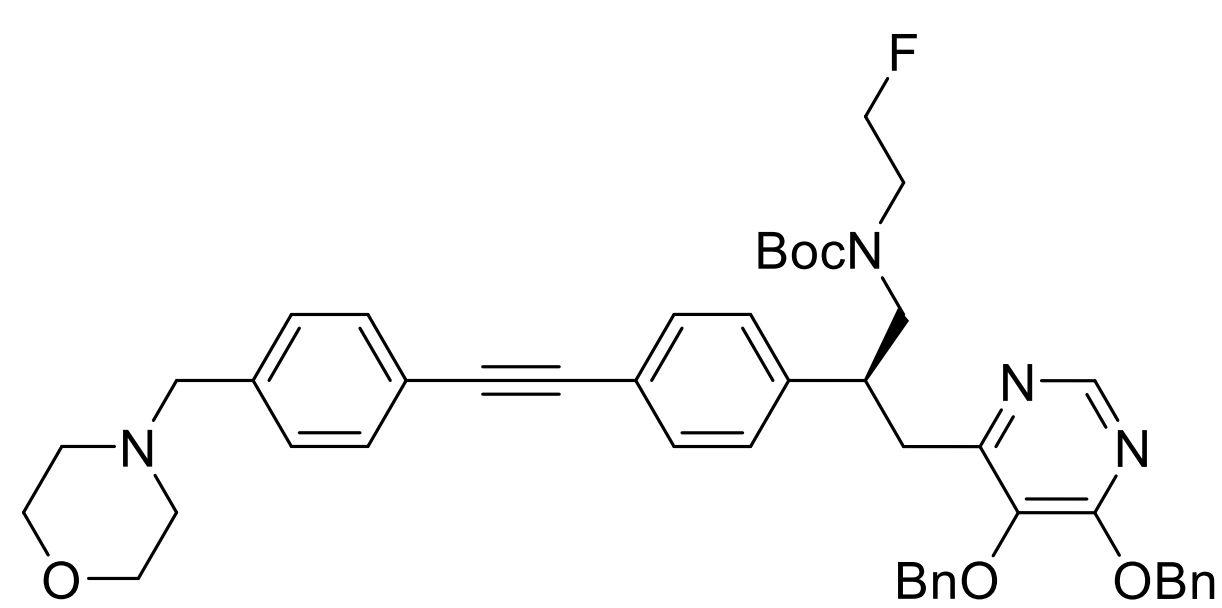

64

Crude **63** was converted to **64** utilizing General Procedure G (0.50 g racemate, 47%) followed by SFC separation. LC-MS (m/z): 671.3 $[M+H]^+$.

(*S*)-6-[3-({2-fluoroethyl}amino)-2-(4-{[4-(morpholinomethyl)phenyl]ethynyl}phenyl)propyl]-5-hydroxypyrimidin-4(3H)-one (8i):

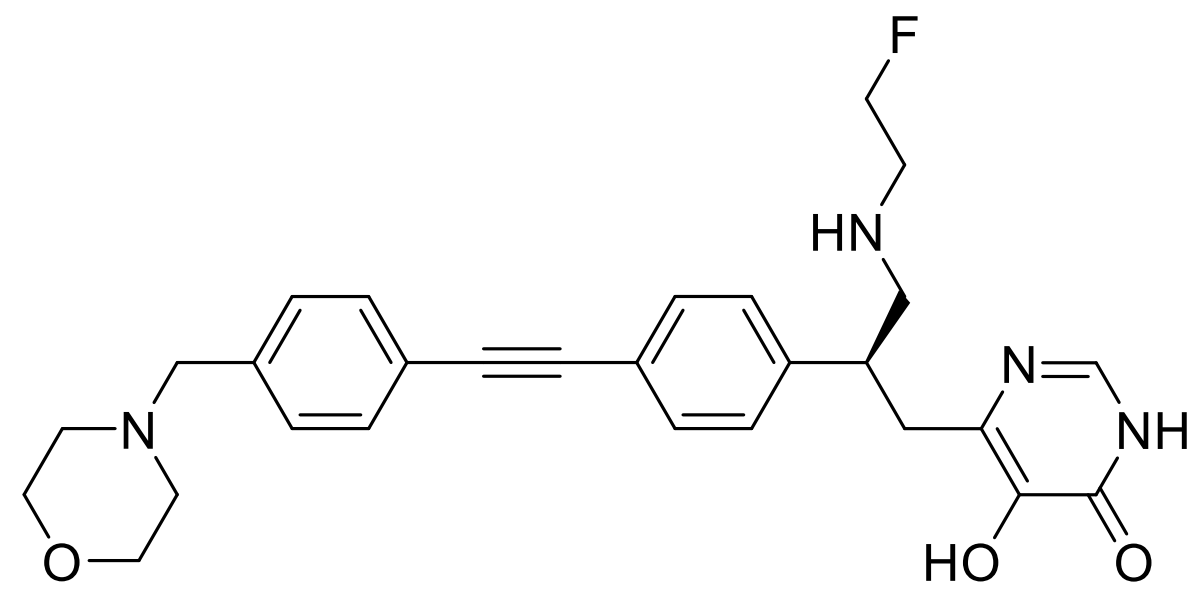

8i

Compound **64** was converted to **8i** utilizing General Procedure H (0.110 g, 81%). LC-MS (m/z): 491.3 $[M+H]^+$. ^{1}H-NMR (DMSO-d_6): δ 8.15 (br s, 2H), 7.67 (br s, 1H), 7.50-7.43 (m, 4H), 7.36 (d, J = 8.16 Hz, 2H), 7.28 (d, J = 8.12 Hz, 2H), 4.59-4.57 (m, 1H), 4.47-4.45 (m, 1H), 3.59-3.57 (m, 4H), 3.50-3.42 (m, 4H), 3.17 (m, 1H), 3.04-3.02 (m, 3H), 2.93-2.90 (m, 3H), 2.36-2.33 (m, 4H).

Procedures for the preparation of Compound 8j

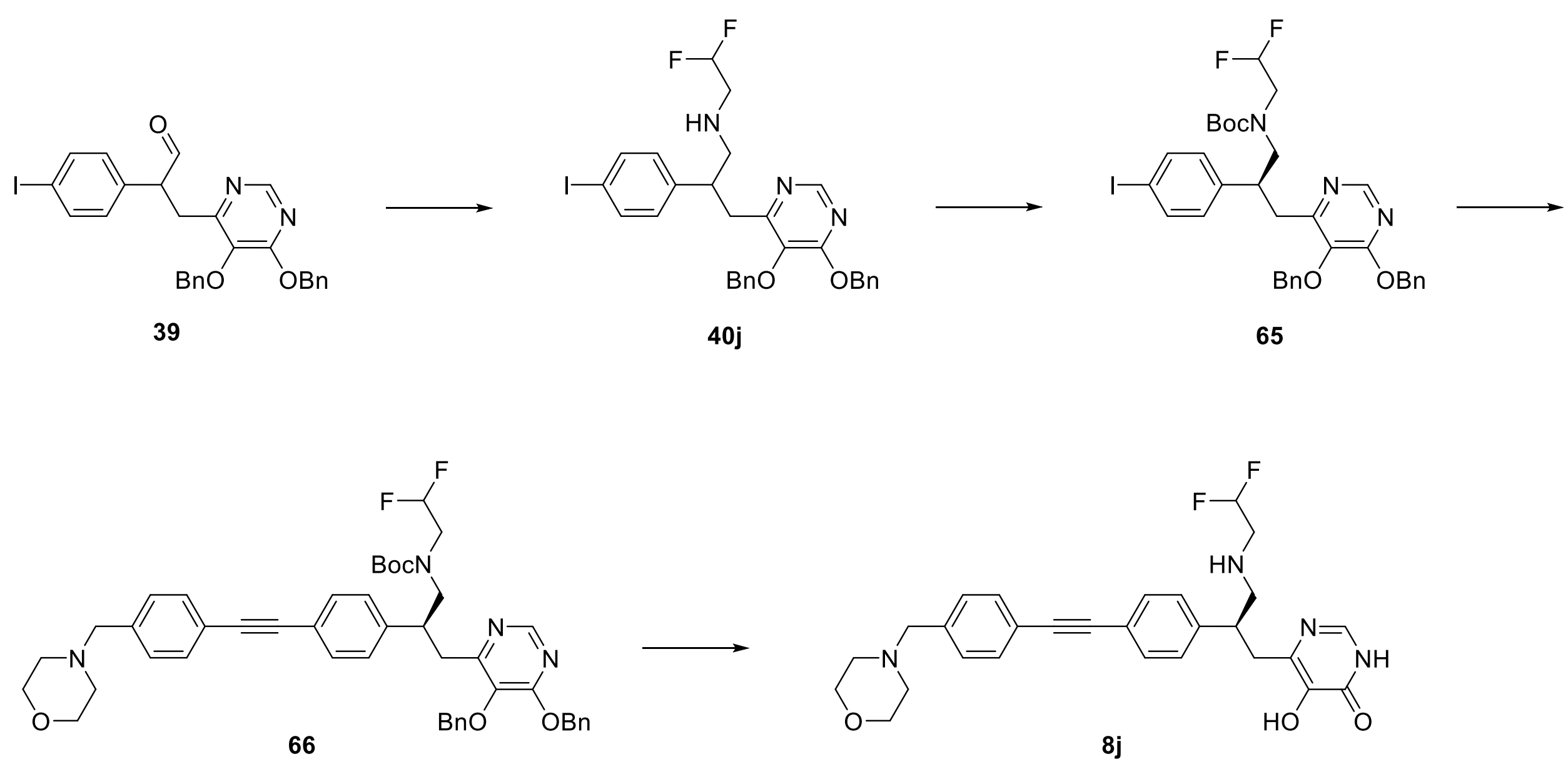

3-[5,6-bis(benzyloxy)pyrimidin-4-yl]-*N*-(2,2-difluoroethyl)-2-(4-iodophenyl)propan-1-amine (40j):

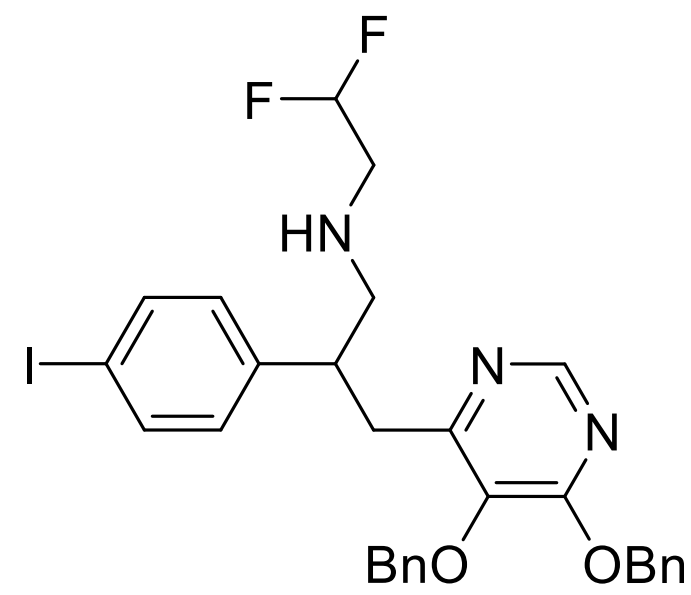

40j

Compound **40j** was prepared from **39** and 2,2-difluoroethan-1-amine utilizing General Procedure F (2.5 g, 55%). LC-MS (m/z): 616.2 $[M+H]^+$.

***tert*-butyl-(*S*)-{3-[5,6-bis(benzyloxy)pyrimidin-4-yl]-2-[4-iodophenyl]propyl}-(2,2-difluoroethyl)carbamate (65):**

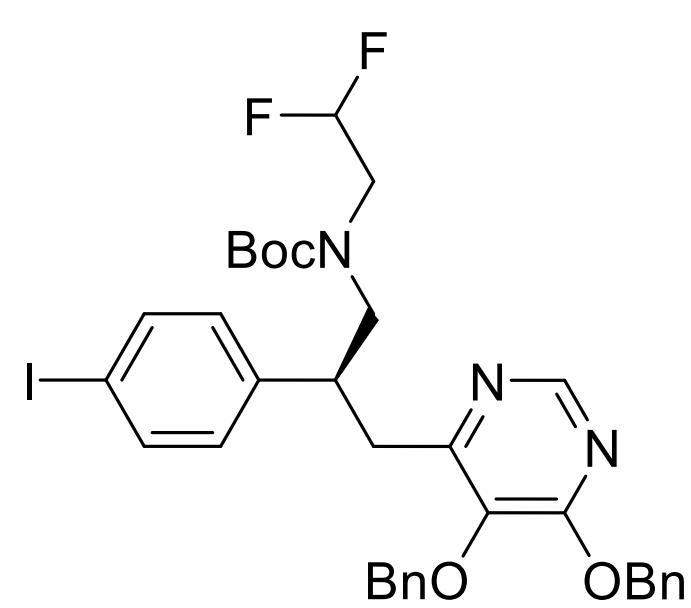

65

Compound **65** was prepared from **40j** utilizing General Procedure G (2.5 g racemate, 86%) followed by SFC separation. LC-MS (m/z): 616.2 $[M+H]^+$.

***tert*-butyl-(*S*)-[3-(5,6-bis{benzyloxy}pyrimidin-4-yl)-2-(4-{[4-(morpholinomethyl)phenyl]-ethynyl}phenyl)propyl](2,2-difluoroethyl)carbamate (66):**

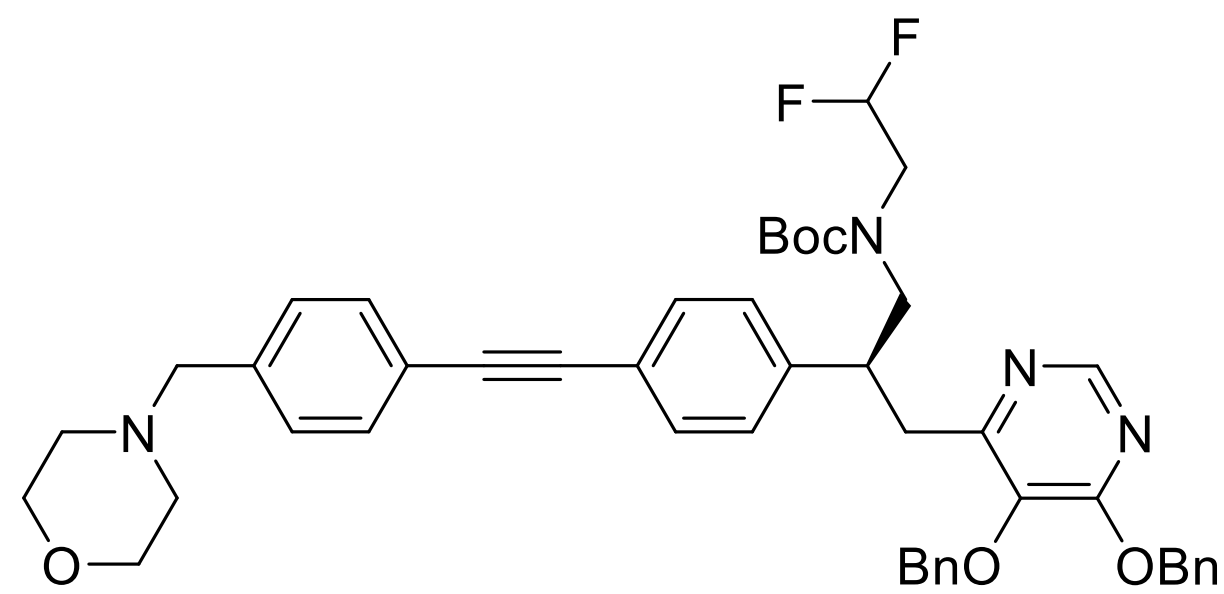

66

Compound **66** was prepared from **30** and **65** utilizing General Procedure B (200 mg, 90%). LC-MS (m/z): 789.3 $[M+H]^+$.

(*S*)-6-[3-({2,2-difluoroethyl}amino)-2-(4-{[4-(morpholinomethyl)phenyl]ethynyl}phenyl)-propyl]-5-hydroxypyrimidin-4(3H)-one (8j):

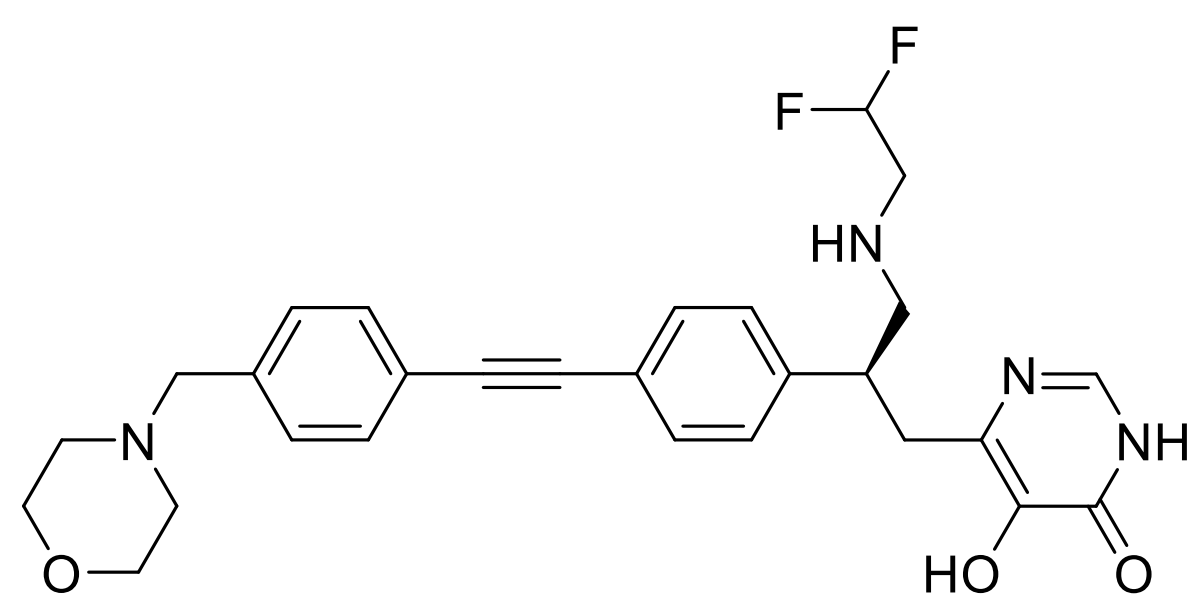

8j

Compound **68** was converted to **8j** utilizing General Procedure H (96 mg, 74%). LC-MS (m/z): 509.2 $[M+H]^+$. ^{1}H-NMR (DMSO-d_6): δ 11.57 (br s, 1H), 9.47 (br s, 1H), 7.89 (s, 1H), 7.69 (d, J = 8.4 Hz, 2H), 7.62 (d, J = 8.0 Hz, 2H), 7.51 (d, J = 8.0 Hz, 2H), 7.36 (d, J = 8.0 Hz, 2H), 6.57-6.42 (m, 1H), 4.50-4.30 (m, 2H), 4.00-3.80 (m, 2H), 3.85-3.75 (m, 2H), 3.75-3.60 (m, 1H), 3.50-3.30 (m, 5H), 3.25-3.00 (m, 4H), 2.00-1.85 (m, 2H).

Procedures for the preparation of Compound 6k

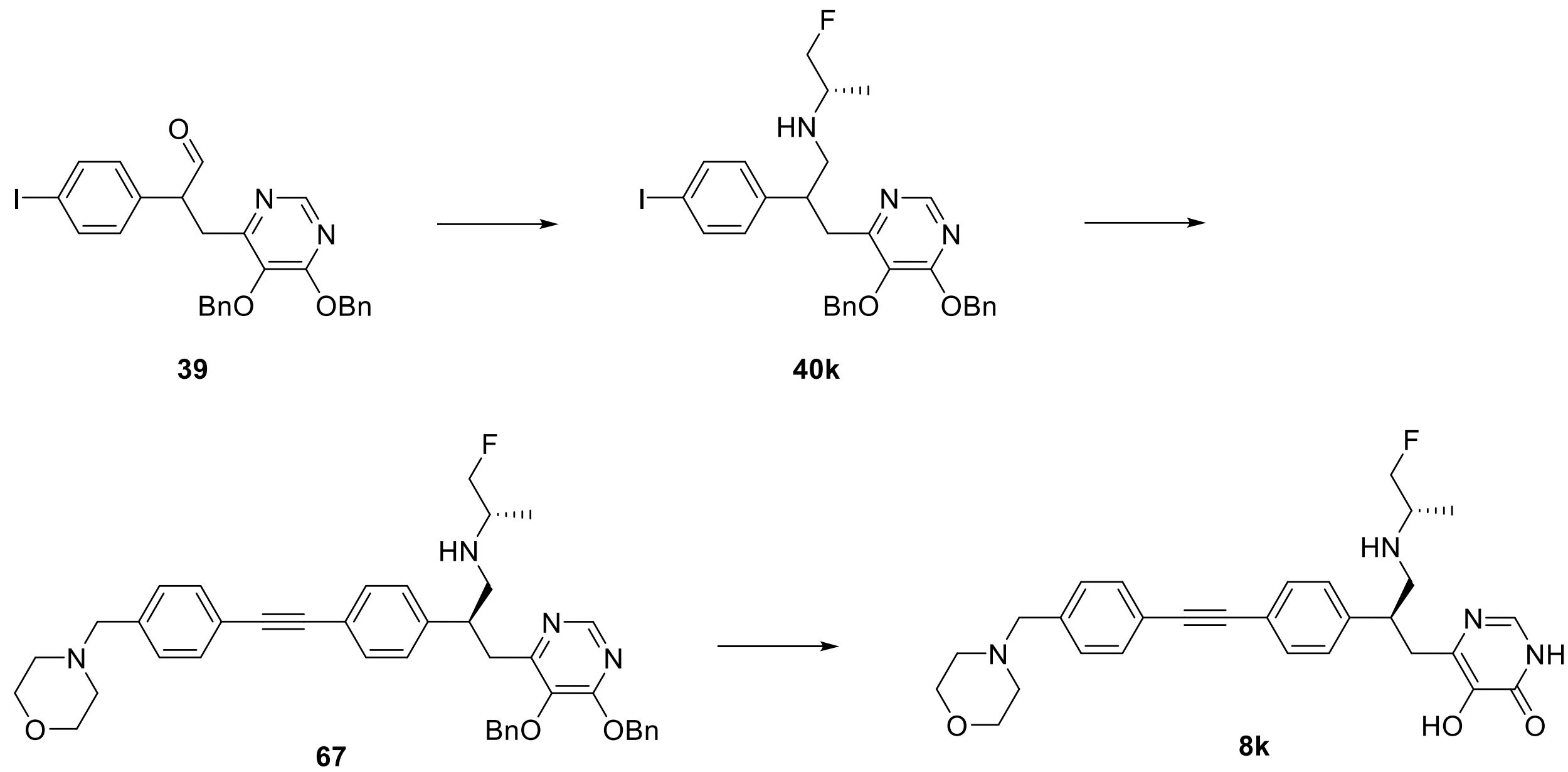

3-[5,6-bis(benzyloxy)pyrimidin-4-yl]-*N*-((*S*)-1-fluoropropan-2-yl)-2-(4-iodophenyl)propan-1-amine (40k):

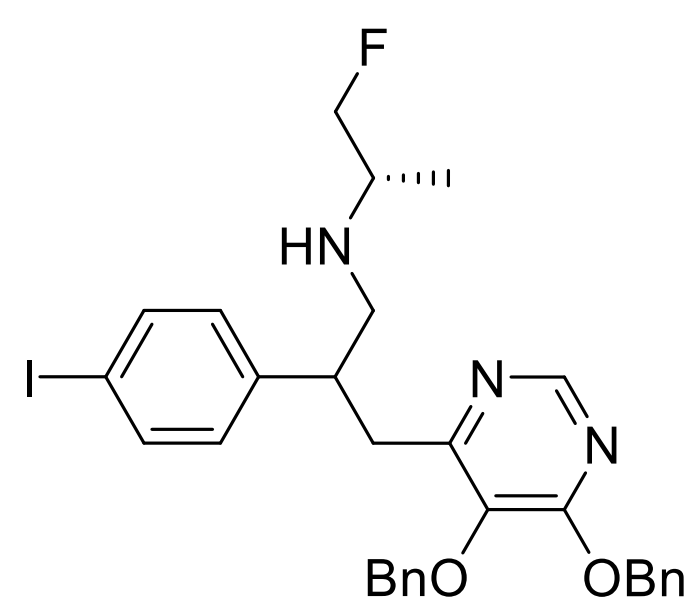

40k

Compound **39** was converted to **40k** utilizing General Procedure E (0.12 g, 60%). LC-MS (m/z): 612.1 $[M+H]^+$.

(*S*)-3-[5,6-bis(benzyloxy)pyrimidin-4-yl]-*N*-((*S*)-1-fluoropropan-2-yl)-2-{4-[(4-(morpholinomethyl)phenyl]ethynyl}phenyl)propan-1-amine (67):

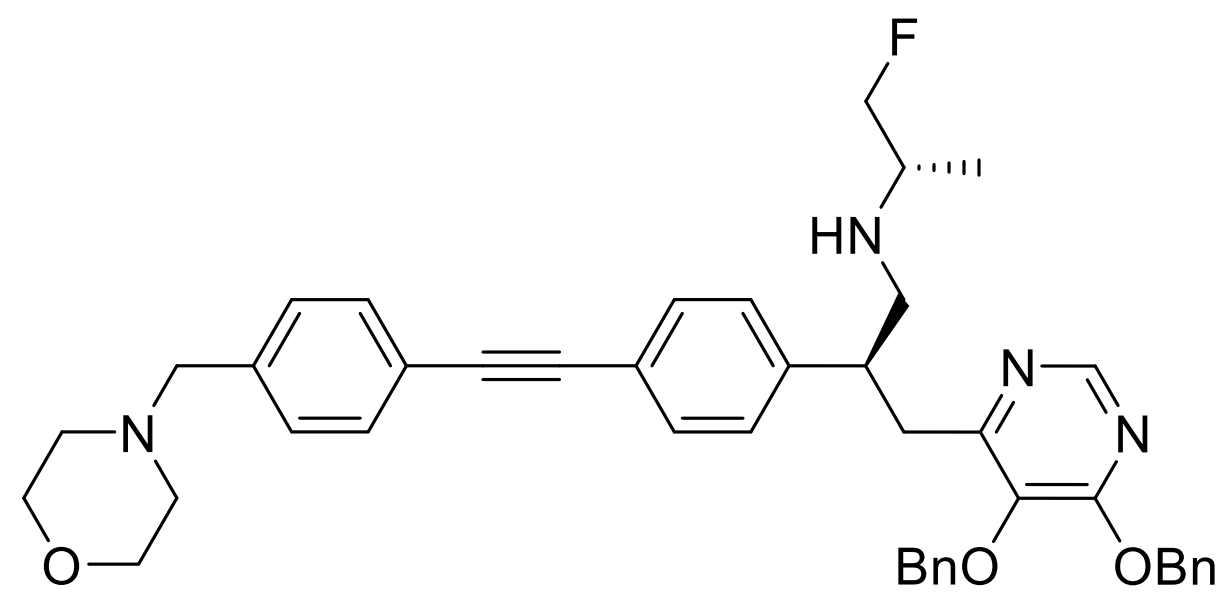

67

Compound **67** was prepared from **30** and **40k** utilizing General Procedure B (80 mg, 60%) followed by SFC separation. LC-MS (m/z): 685.4 $[M+H]^+$.

6[(*S*)-3-({(*S*)-1-fluoropropan-2-yl}amino)-2-(4-{[4-(morpholinomethyl)phenyl]ethynyl}-phenyl)propyl]-5-hydroxypyrimidin-4(3H)-one (8k):

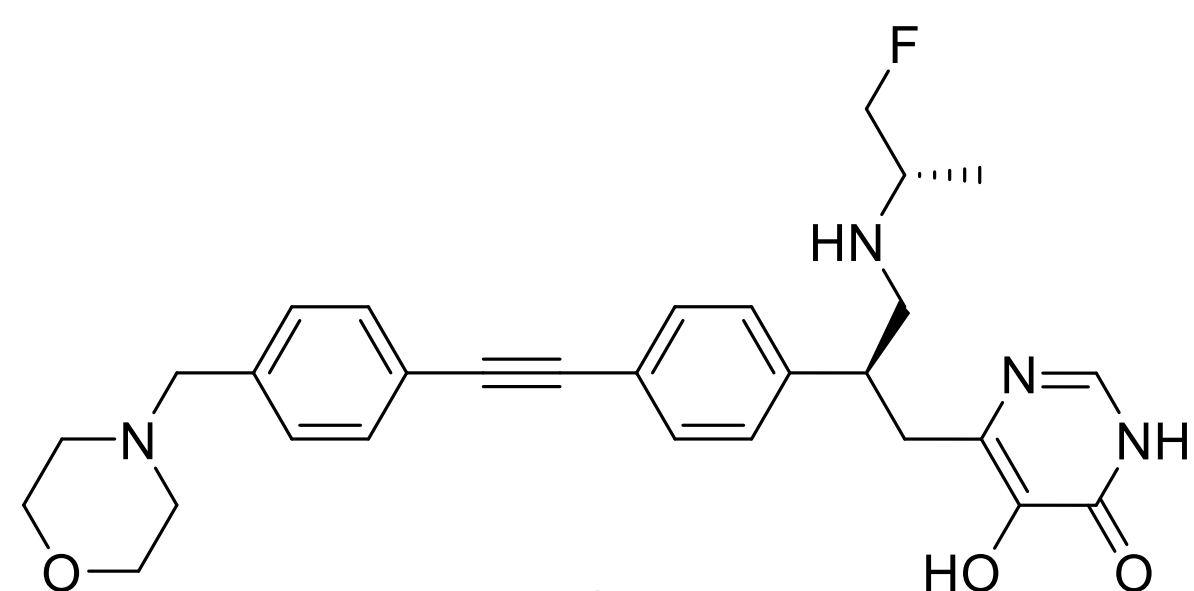

8k

Compound **8k** was prepared from **67** utilizing General Procedure D (200 mg, 65%). LC-MS (m/z): 505.3 $[M+H]^+$. ^{1}H NMR (DMSO-d_6): δ 8.16 (br s, 2H), 7.66 (br s, 1H), 7.48-7.42 (m, 4H), 7.35 (d, J = 8.08 Hz, 2H), 7.27 (d, J = 7.44 Hz, 2H), 4.51-4.29 (m, 3H), 3.74 (m, 4H), 3.56 (s, 2H), 3.48-3.15 (m, 4H), 2.88-2.87 (m, 3H), 2.35 (m, 4H), 1.06 (d, J = 6.08 Hz, 3H)

Procedures for the preparation of Compound 8l

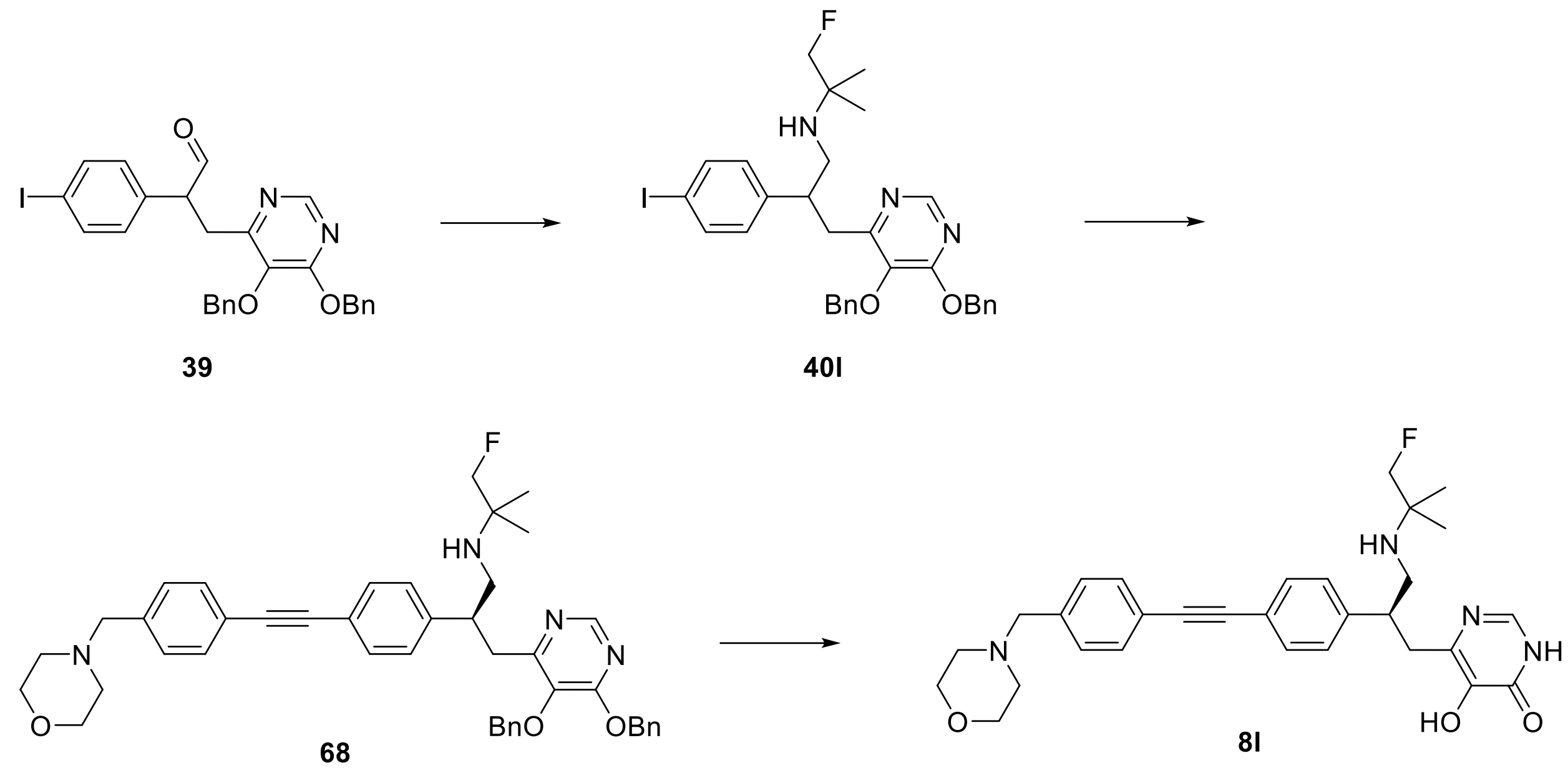

3-[5,6-bis(benzyloxy)pyrimidin-4-yl]-N-(1-fluoro-2-methylpropan-2-yl)-2-(4-iodophenyl)-propan-1-amine (40l):

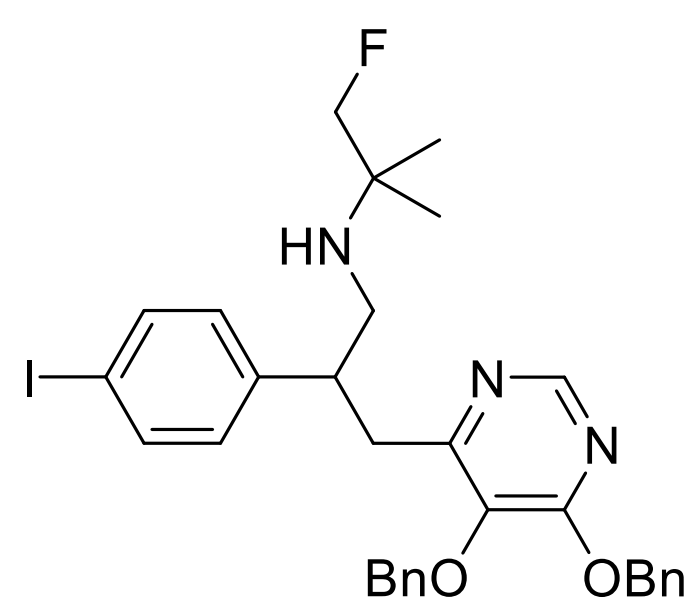

40l

Compound **40l** was prepared from **39** and 1-fluoro-2-methylpropan-2-amine hydrochloride utilizing General Procedure F (2.7 g, 60%). LC-MS (m/z): 626.2 $[M+H]^+$. The resulting racemate was separated by SFC before proceeding to the next step.

(*S*)-3-[5,6-bis(benzyloxy)pyrimidin-4-yl]-N-(1-fluoro-2-methylpropan-2-yl)-2-(4-{[4-(morpholinomethyl)phenyl]ethynyl}phenyl)propan-1-amine (68):

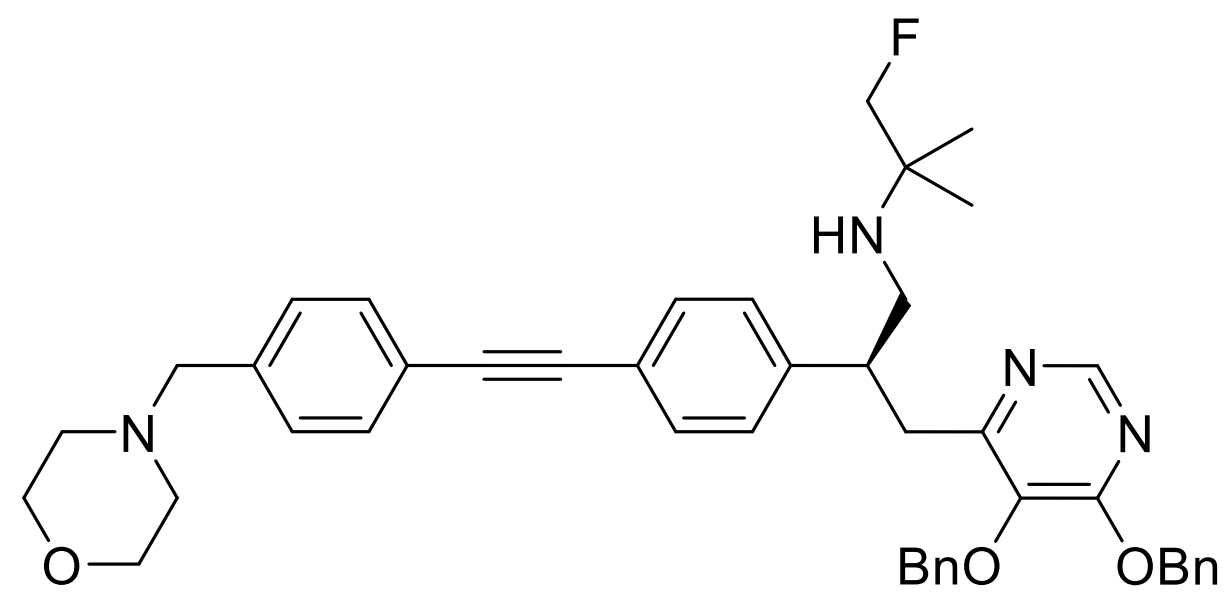

68

Compound **68** was prepared from **30** and **(*S*)-40l** utilizing General Procedure B (400 mg, 71%). LC-MS (m/z): 699.2 $[M+H]^+$.

(*S*)-6-[3-({1-fluoro-2-methylpropan-2-yl}amino)-2-(4-{[4-(morpholinomethyl)phenyl]-ethynyl}phenyl)propyl]-5-hydroxypyrimidin-4(3H)-one (8l):

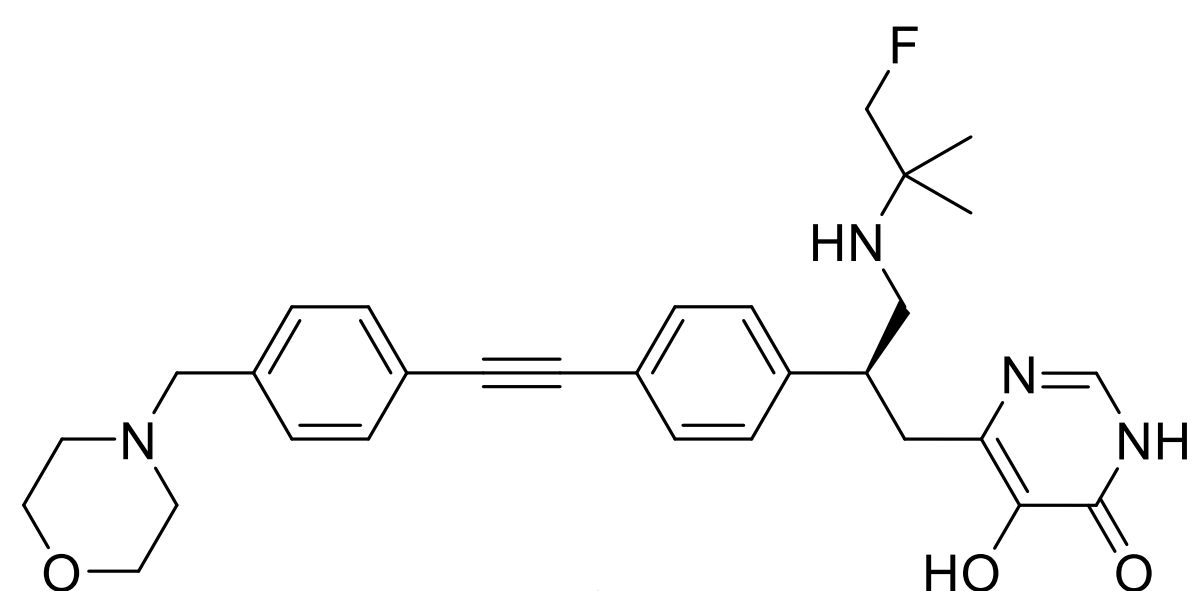

8l

Compound **8l** was prepared from **68** utilizing General Procedure D (200 mg, 65%). LC-MS (m/z): 519.3 $[M+H]^+$. ^1H NMR (DMSO-d_6): δ 8.14 (s, 1H), 7.68 (s, 1H), 7.49 (d, J = 8.0 Hz, 2H), 7.44 (d, J = 8.0 Hz, 2H), 7.36 (d, J = 8.0 Hz, 2H), 7.28 (d, J = 8.0 Hz, 2H), 4.40-4.28 (m, 2H), 3.65-3.55 (m, 4H), 3.50 (s, 3H), 3.10-3.00 (m, 2H), 2.95-2.85 (m, 2H), 2.40-2.30 (m, 4H), 1.30-1.15 (m, 6H).

Procedures for the preparation of Compound 8m

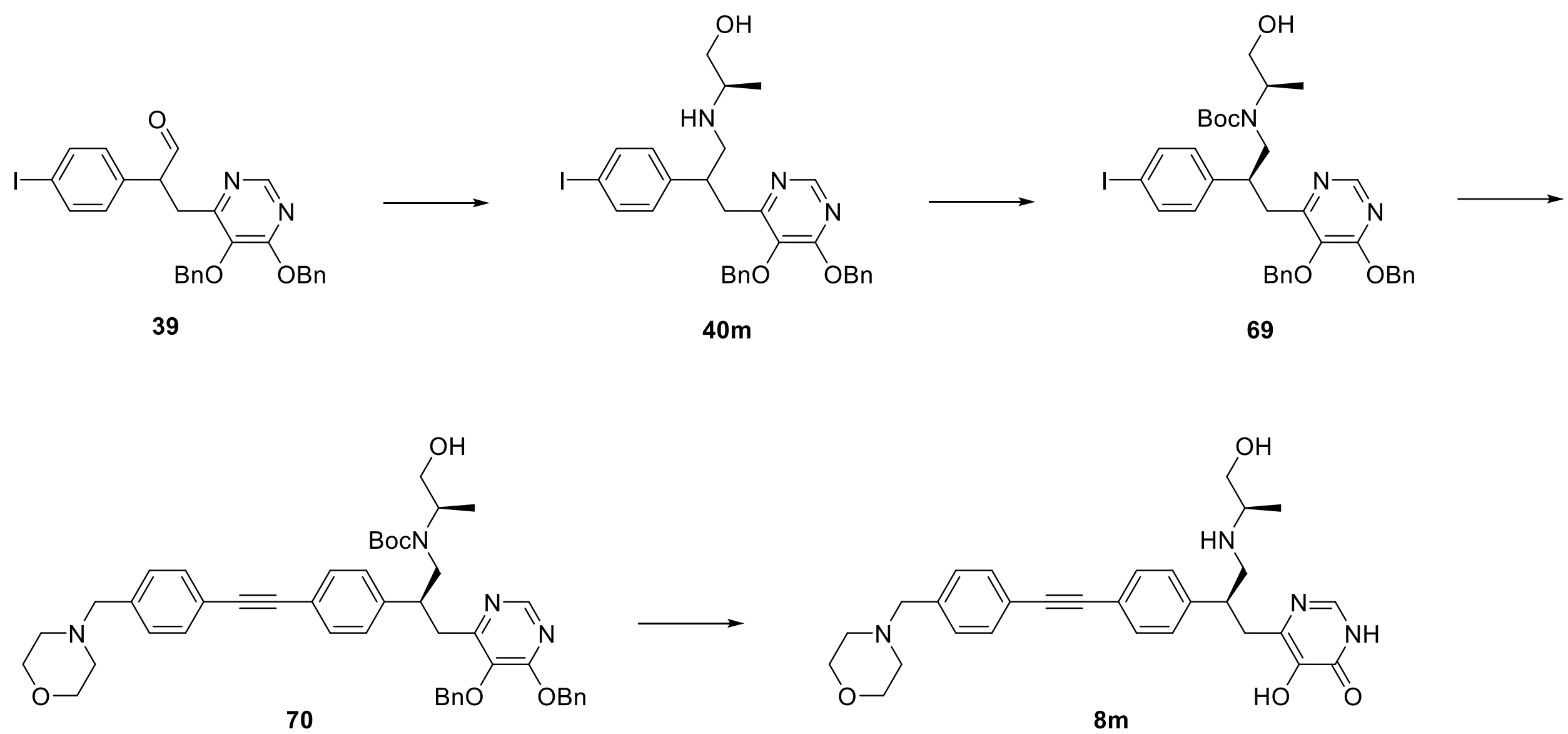

(2*R*)-2-({3-[5,6-bis(benzyloxy)pyrimidin-4-yl]-2-[4-iodophenyl]propyl}amino)propan-1-ol (40m):

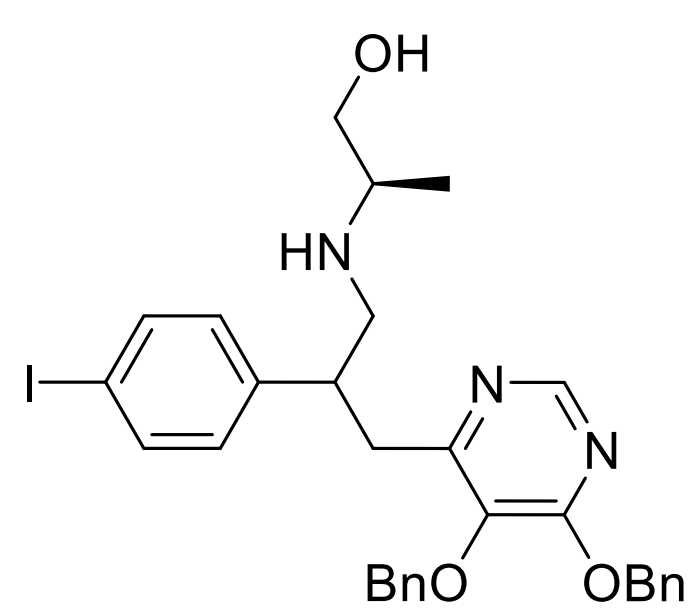

40m

Compound **40m** was prepared from **39** and (*R*)-2-aminopropan-1-ol hydrochloride utilizing General Procedure E (2.4 g, 73%). LC-MS (m/z): 610.1 $[M+H]^+$.

***tert*-butyl-{(*S*)-3-[5,6-bis(benzyloxy)pyrimidin-4-yl]-2-[4-iodophenyl]propyl}((*R*)-1-hydroxypropan-2-yl)carbamate (69):**

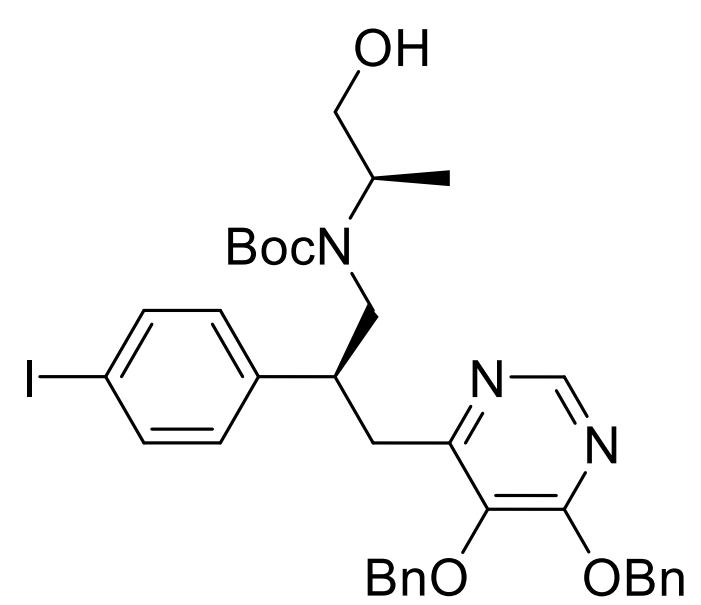

69

Compound **69** was prepared from **40m** utilizing General Procedure G (2.2 g racemate, 85%) followed by SFC separation. LC-MS (m/z): 710.2 $[M+H]^+$.

***tert*-butyl-[(*S*)-3-(5,6-bis{benzyloxy}pyrimidin-4-yl)-2-(4-{[4-(morpholinomethyl)phenyl]-ethynyl}phenyl)propyl]((*R*)-1-hydroxypropan-2-yl)carbamate (70):**

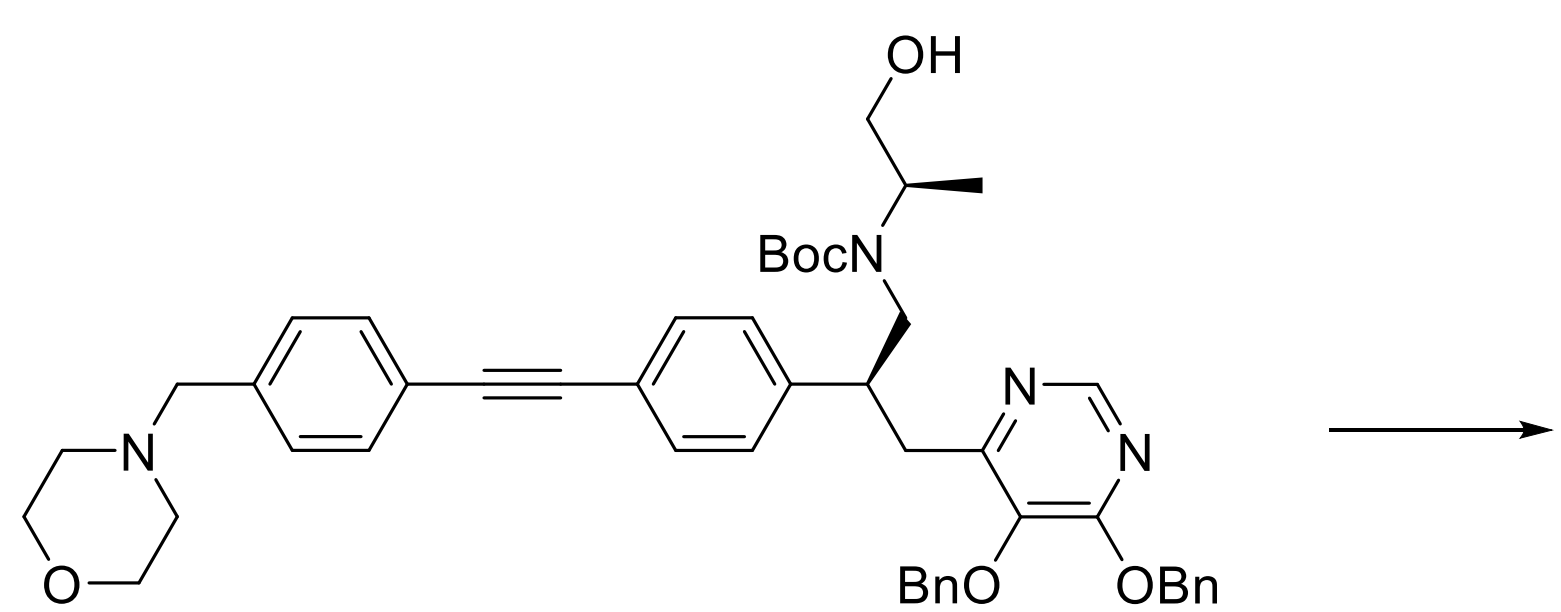

70

Compound **70** was prepared from **30** and **69** utilizing General Procedure B (140 mg, 42%). LC-MS (m/z): 783.3 $[M+H]^+$.

5-hydroxy-6-[(*S*)-3-({(*R*)-1-hydroxypropan-2-yl}amino)-2-(4-{[4-(morpholinomethyl)-phenyl]ethynyl}phenyl)propyl]pyrimidin-4(3H)-one (8m):

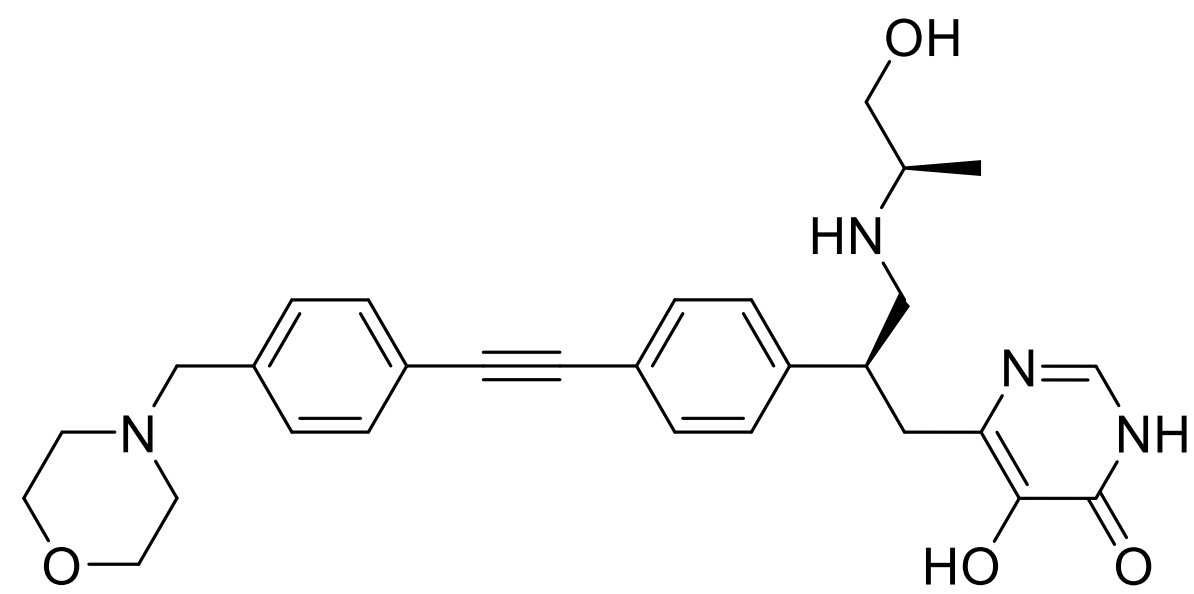

8m

Compound **70** was converted to **8m** utilizing General Procedure D (60 mg, 68%). LC-MS (m/z): 503.3 [M+H]$^+$. ^{1}H-NMR (DMSO-d_6): δ 8.23 (br s, 1H), 7.66 (br s, 1H), 7.49 (d, J = 6.24 Hz, 2H), 7.42 (d, J = 8.04 Hz, 2H), 7.35 (d, J = 8.08 Hz, 2H), 7.26 (d, J = 8.08 Hz, 2H), 3.58-3.57 (m, 4H), 3.49 (s, 2H), 3.36-3.35 (m, 1H), 3.30-3.27 (m, 1H), 3.24-3.22 (m, 1H), 2.94-2.82 (m, 4H), 2.70-2.50 (m, 1H), 2.35 (m, 1H), 0.91 (d, J = 6.04 Hz, 3H).

Procedures for the preparation of Compound 8n

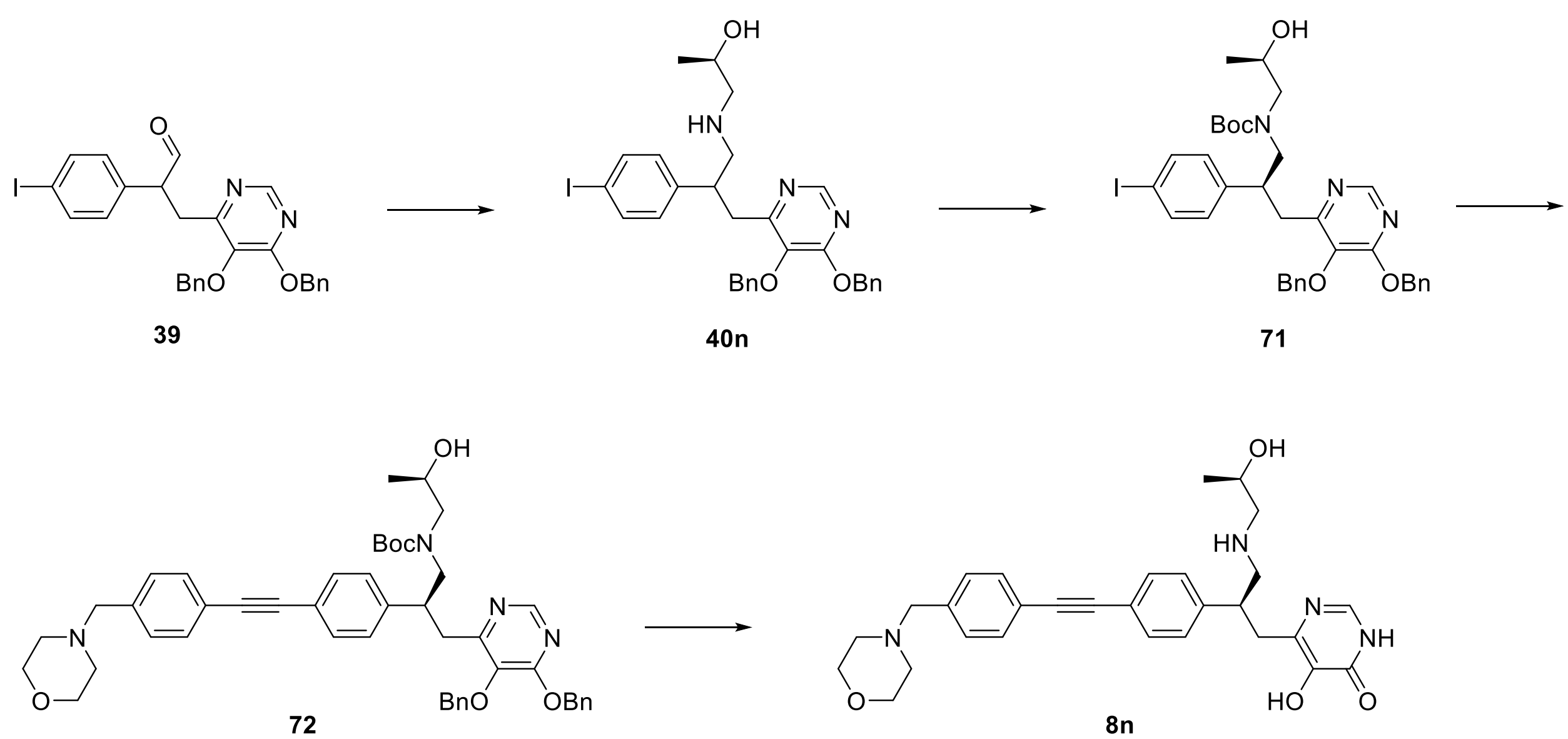

(2*R*)-1-({3-[5,6-bis(benzyloxy)pyrimidin-4-yl]-2-[4-iodophenyl]propyl}amino)propan-2-ol (40n):

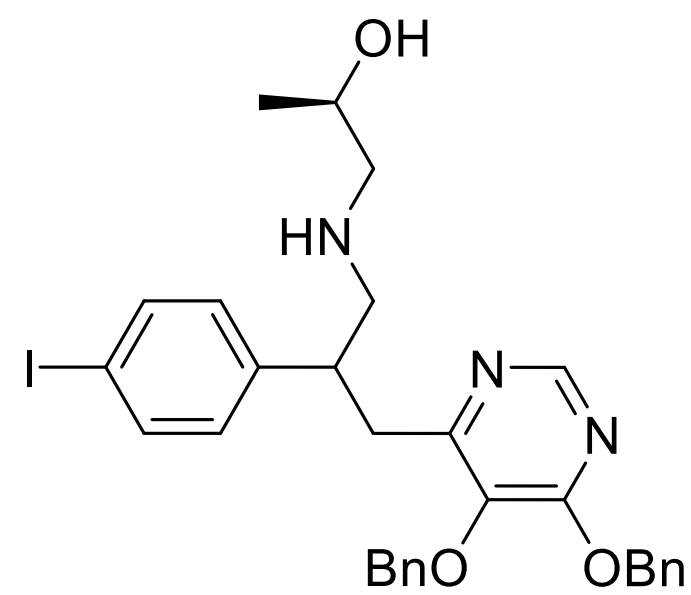

40n

Compound **40n** was prepared from **39** and (*R*)-1-aminopropan-2-ol hydrochloride utilizing General Procedure E (2.2 g, crude). LC-MS (m/z): 624.2 $[M+H+H_2O]^+$.

***tert*-butyl-{(*S*)-3-[5,6-bis(benzyloxy)pyrimidin-4-yl]-2-[4-iodophenyl]propyl}((*R*)-2-hydroxypropyl)carbamate (71):**

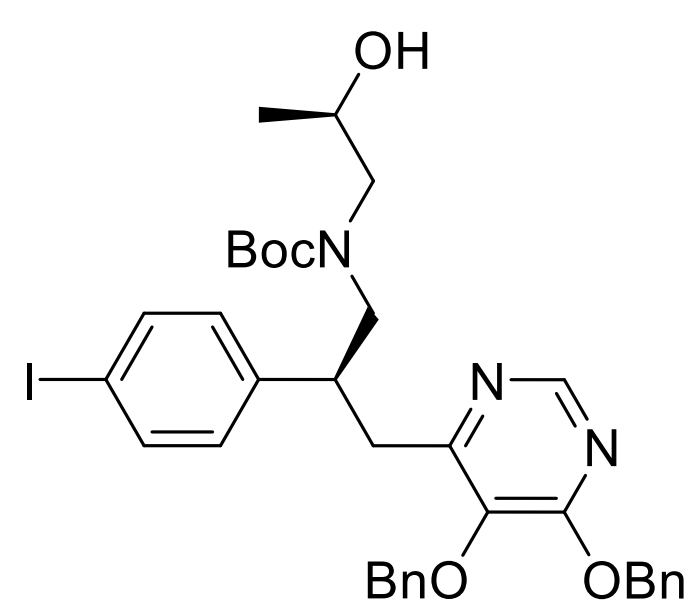

71

Compound **71** was prepared from **40n** utilizing General Procedure G (0.70 g racemate, 29% over two steps) followed by SFC separation. LC-MS (m/z): 710.2 $[M+H]^+$.

***tert*-butyl-[(*S*)-3-(5,6-bis{benzyloxy}pyrimidin-4-yl)-2-(4-{[4-(morpholinomethyl)phenyl]-ethynyl}phenyl)propyl]((*R*)-2-hydroxypropyl)carbamate (72):**

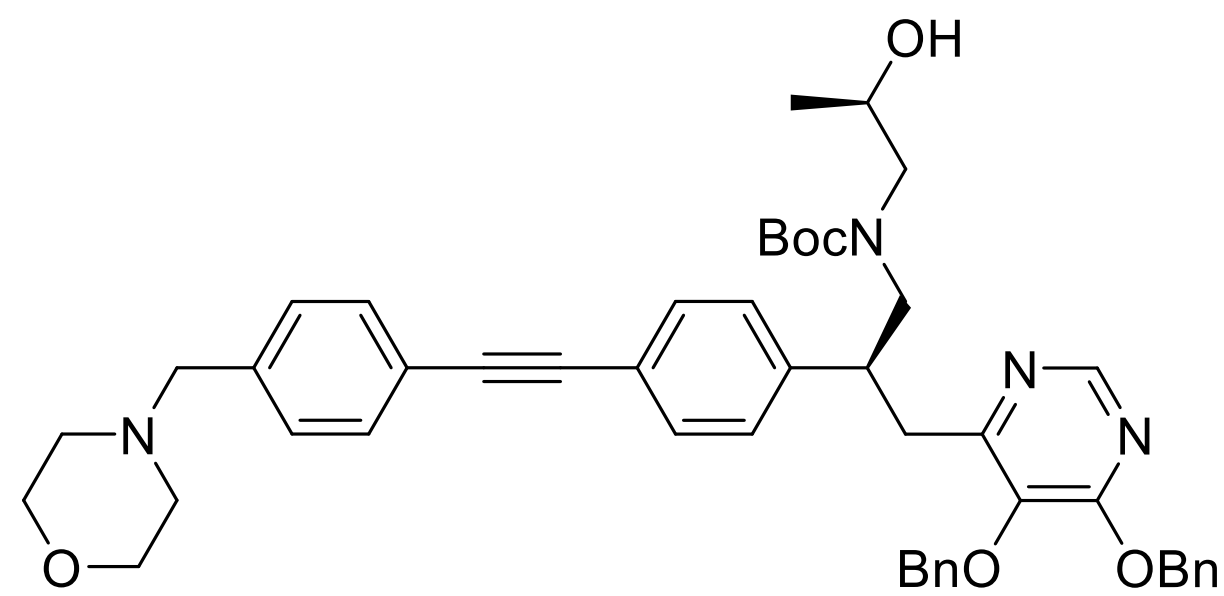

72

Compound **72** was prepared from **30** and **71** utilizing General Procedure B (160 mg, 38%). LC-MS (m/z): 783.3 $[M+H]^+$.

5-hydroxy-6-[(S)-3-({(R)-2-hydroxypropyl}amino)-2-(4-{[4-(morpholinomethyl)phenyl]-ethynyl}phenyl)propyl]pyrimidin-4(3H)-one (8n):

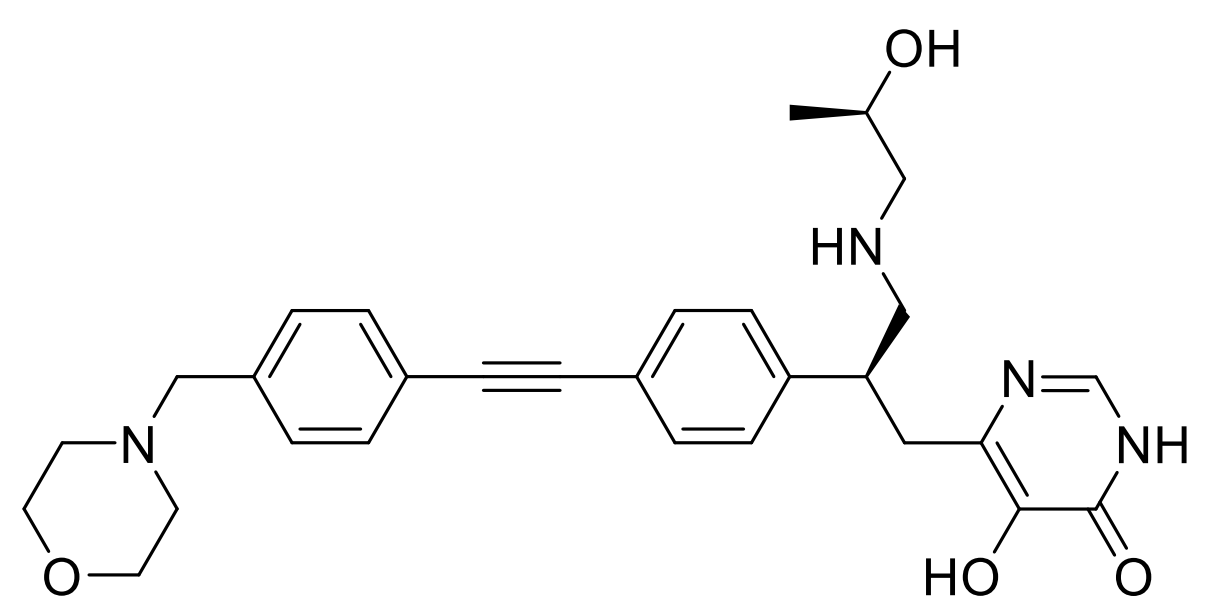

8n

Compound **72** was converted to **8n** utilizing General Procedure D (40 mg, 39%). LC-MS (m/z): 503.2 $[M+H]^+$. ^{1}H-NMR (DMSO-d_6): δ 7.62 (s, 1H), 7.49 (d, J = 7.6 Hz, 3H), 7.41 (d, J = 7.6 Hz, 2H), 7.35 (d, J = 7.6 Hz, 2H), 7.25 (d, J = 6.8 Hz, 2H), 3.75-3.60 (m, 4H), 3.55-3.40 (m, 3H), 3.35-3.20 (m, 1H), 2.90-2.60 (m, 6H), 2.45-2.25 (m, 6H), 0.97 (d, J = 5.6 Hz, 3H).

Procedures for the preparation of Compound 8o

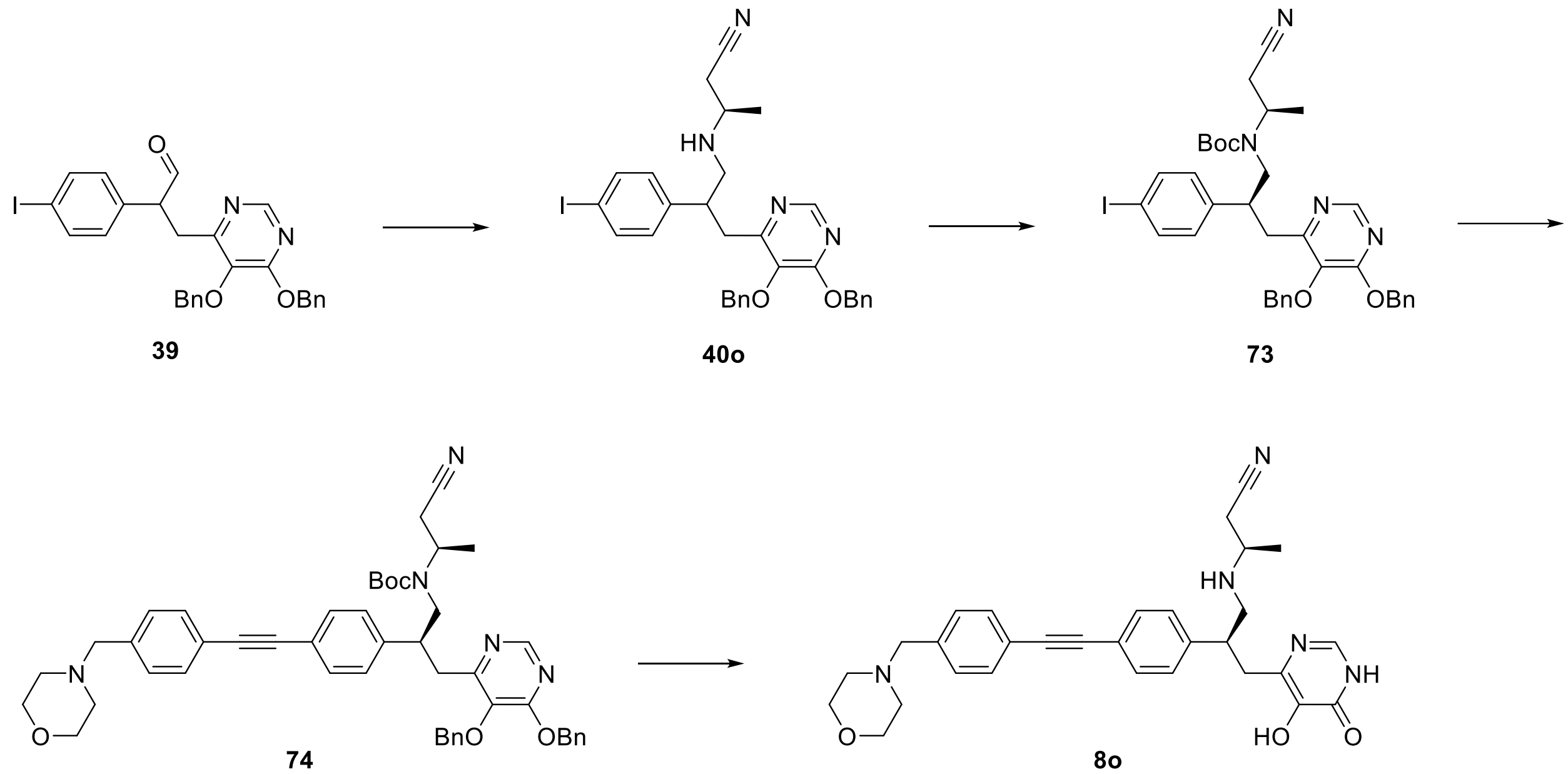

(3*R*)-3-({3-[5,6-bis(benzyloxy)pyrimidin-4-yl]-2-[4-iodophenyl]propyl}amino)butanenitrile (40o):

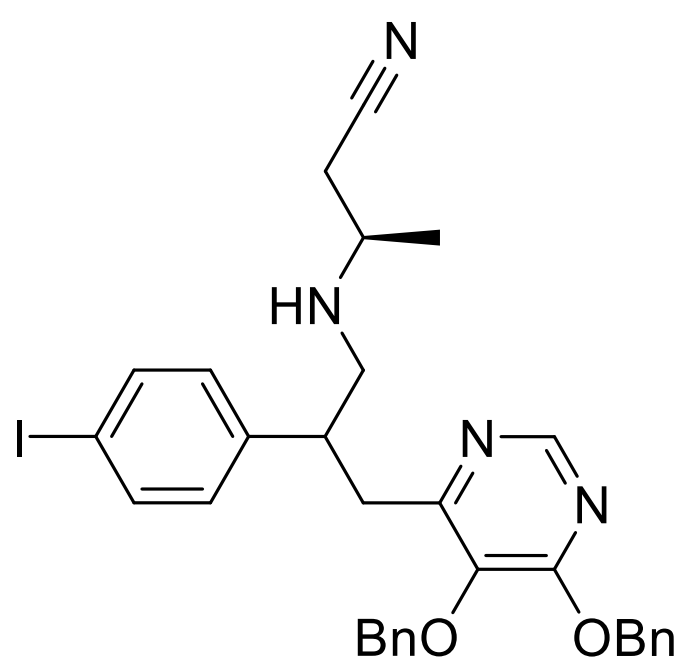

40o

Compound **40o** was prepared from **39** and (*R*)-3-aminobutanenitrile utilizing General Procedure F (1.3 g, 60%). LC-MS (m/z): 619.2 $[M+H]^+$.

***tert*-butyl-{(*S*)-3-[5,6-bis(benzyloxy)pyrimidin-4-yl]-2-[4-iodophenyl]propyl}((*R*)-1-cyanopropan-2-yl)carbamate (73):**

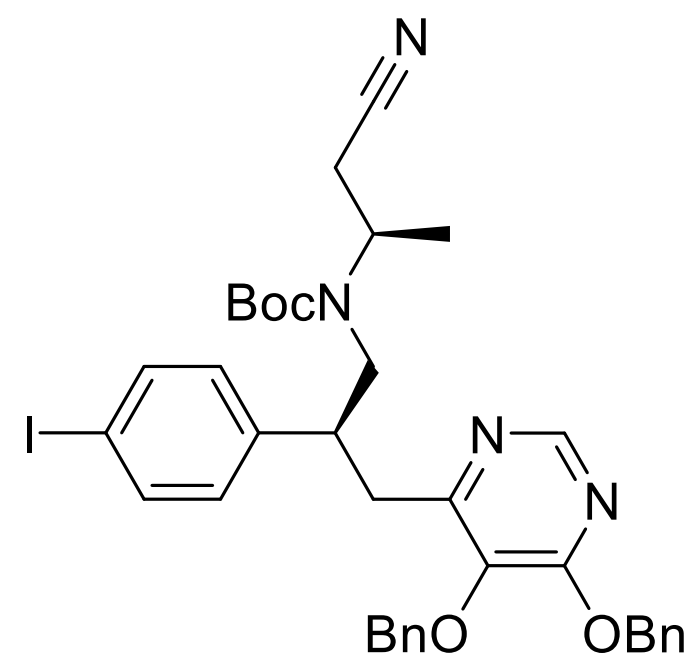

73

Compound **73** was prepared from **40o** utilizing General Procedure G (0.84 g racemate, 68%) followed by SFC separation. LC-MS (m/z): 719.1 [M+H]$^+$.

***tert*-butyl-[(*S*)-3-(5,6-bis{benzyloxy}pyrimidin-4-yl)-2-(4-{[4-(morpholinomethyl)phenyl]-ethynyl}phenyl)propyl]((*R*)-1-cyanopropan-2-yl)carbamate (74):**

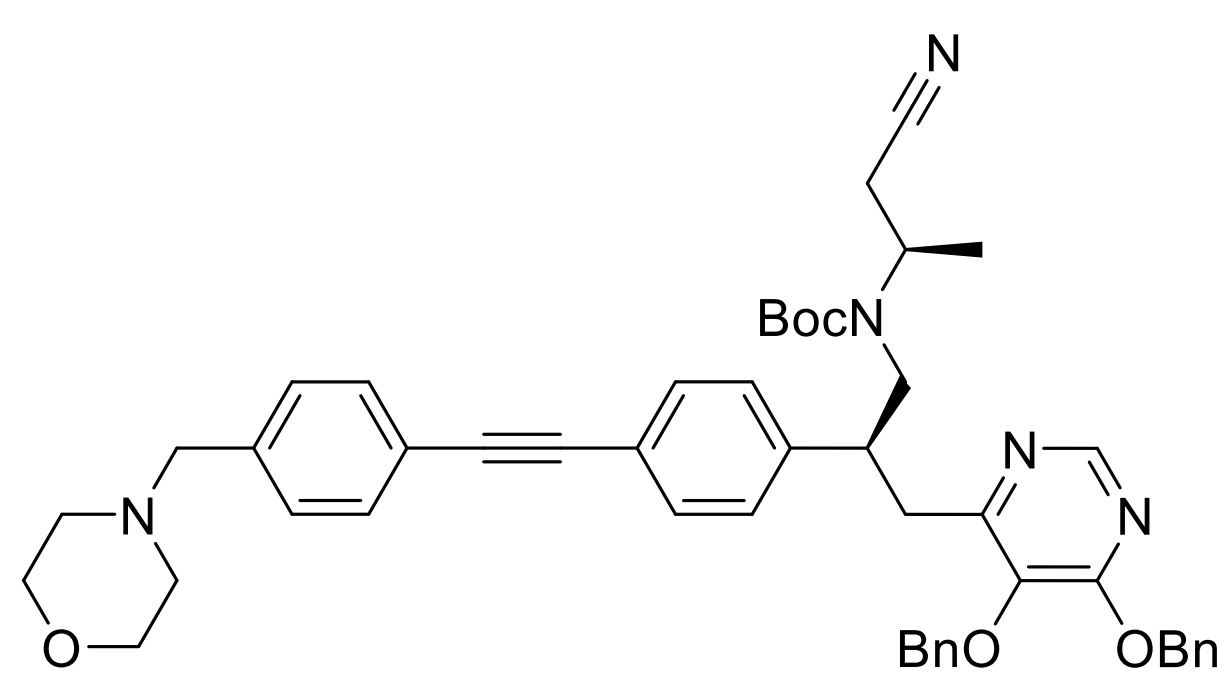

74

Compound **74** was prepared from **30** and **73** utilizing General Procedure B (170 mg, 47%). LC-MS (m/z): 792.3 [M+H]$^+$.

(*R*)-3-{[(*S*)-3-(5-hydroxy-6-oxo-1,6-dihydropyrimidin-4-yl)-2-(4-{[4-(morpholinomethyl)-phenyl]ethynyl}phenyl)propy]amino}butanenitrile (8o):

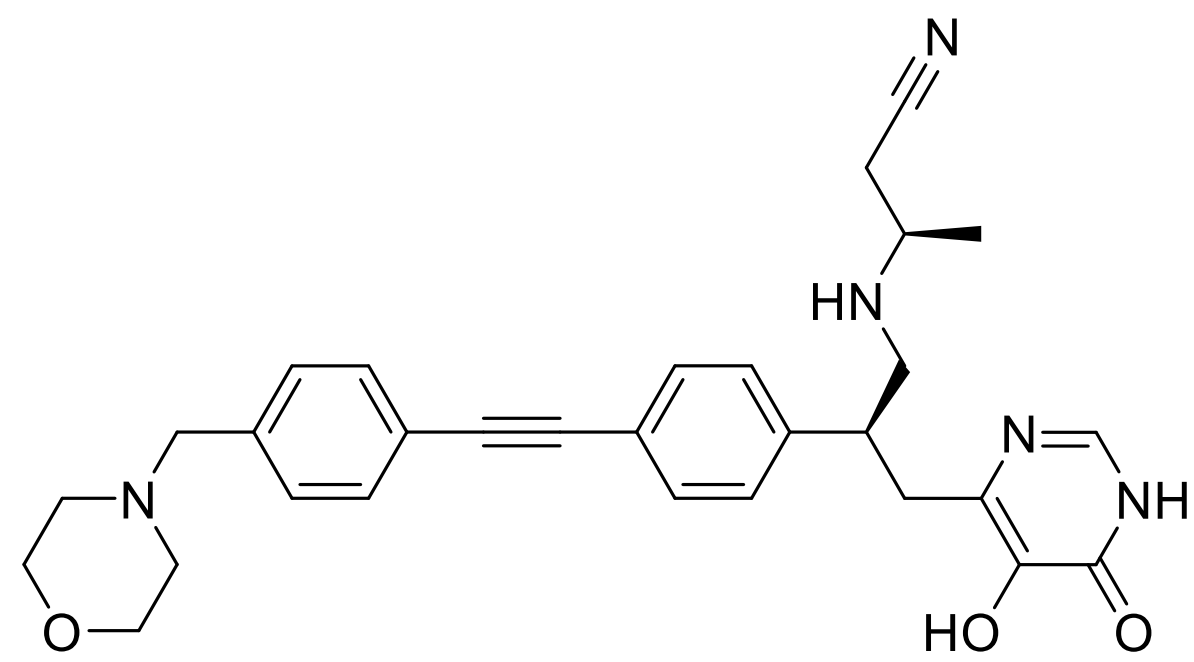

8o

Compound **74** was converted to **8o** utilizing General Procedure D (50 mg, 52%). LC-MS (m/z): 512.3 $[M+H]^+$. ^{1}H-NMR (DMSO-d_6): δ 12.40 (br s, 1H), 8.29 (br s, 1H), 7.66 (br s, 1H), 7.49 (d, J = 8.00 Hz, 2H), 7.40 (d, J = 8.40 Hz, 2H), 7.35 (d, J = 8.40 Hz, 2H), 7.25 (d, J = 8.40 Hz, 2H), 3.59-3.57 (m, 4H), 3.49-3.34 (m, 2H), 2.95-2.91 (m, 1H), 2.90-2.80 (m, 5H), 2.52-2.50 (m, 2H), 1.03 (d, J = 6.40 Hz, 3H)

Procedures for the preparation of Compound 8p

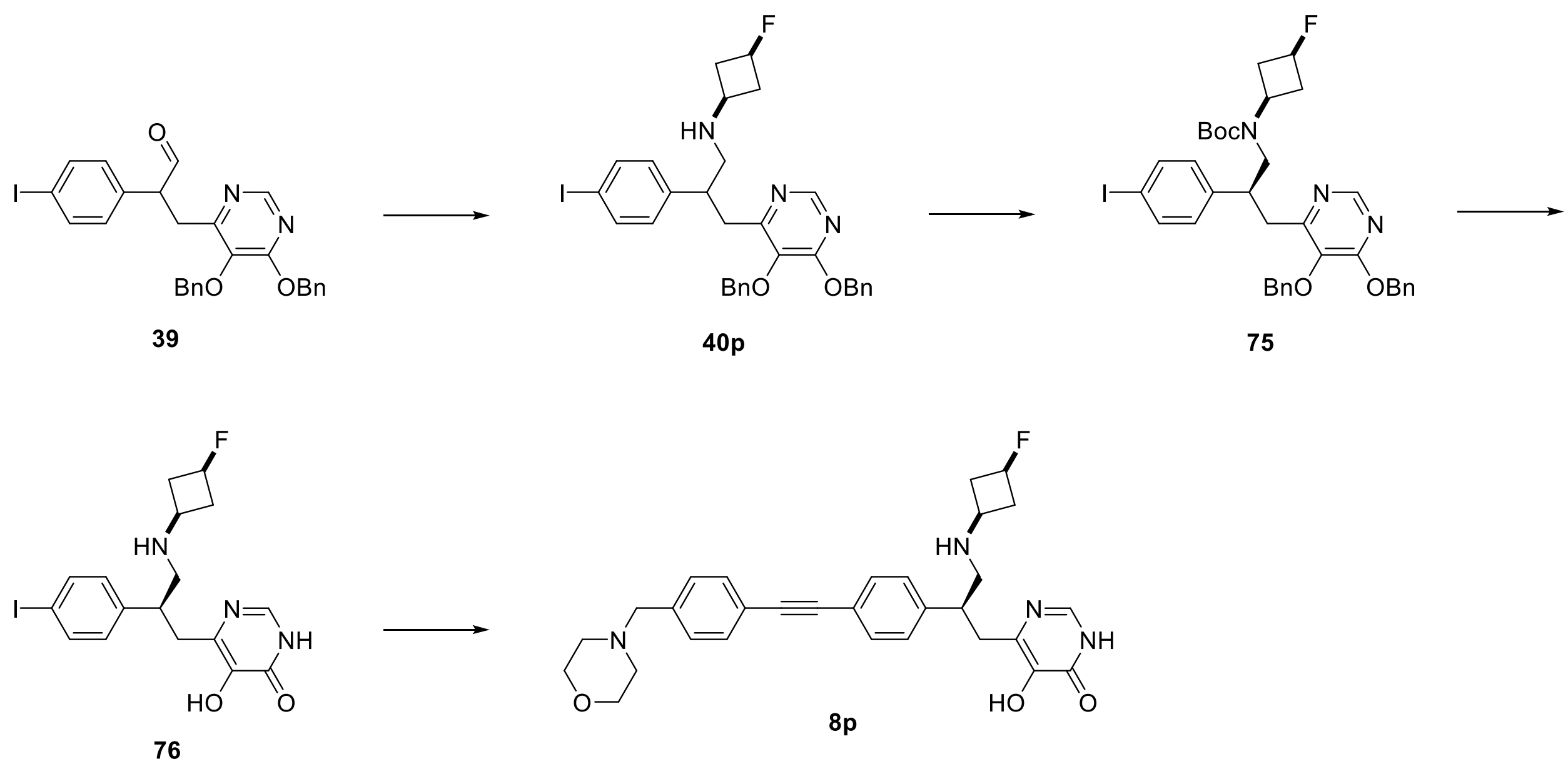

***cis*-*N*-{3-[5,6-bis(benzyloxy)pyrimidin-4-yl]-2-[4-iodophenyl]propyl}-3-fluorocyclobutan-1-amine (40p):**

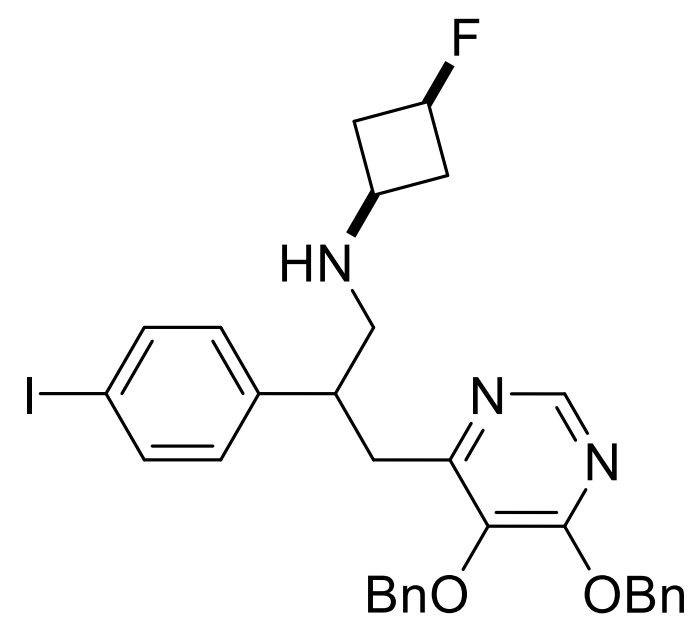

40p

Compound **40p** was prepared from **39** and *cis*-3-fluorocyclobutan-1-amine hydrochloride utilizing General Procedure E (2.2 g, 49%). LC-MS (m/z): 624.2 $[M+H]^+$.

***tert*-butyl-{(*S*)-3-[5,6-bis(benzyloxy)pyrimidin-4-yl]-2-[4-iodophenyl]propyl}(*cis*-3-fluorocyclobutyl)carbamate (75):**

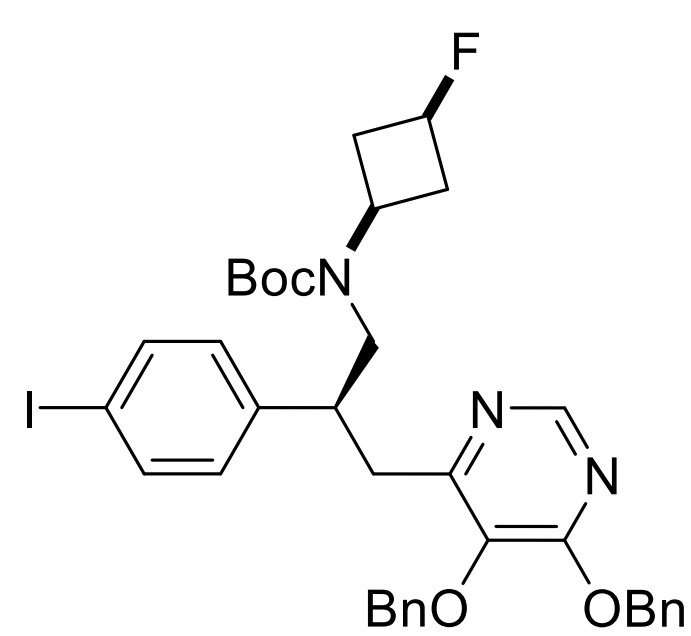

75

Compound **75** was prepared from **40p** utilizing General Procedure G (2.3 g racemate, 90%) followed by SFC separation. LC-MS (m/z): 724.2 $[M+H]^+$.

6-{(*S*)-3-[(*cis*-3-fluorocyclobutyl)amino]-2-[4-iodophenyl]propyl}-5-hydroxypyrimidin-4(3H)-one (76):

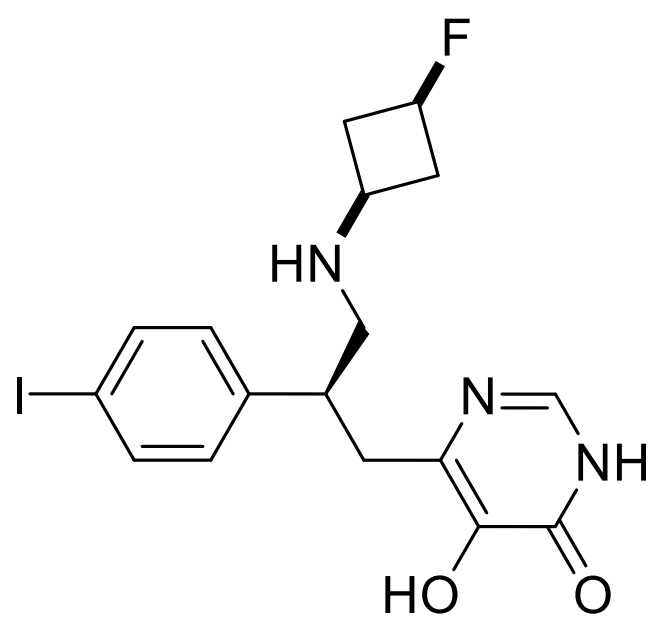

76

Compound **75** was converted to **76** utilizing General Procedure D (120 mg, 95%). LC-MS (m/z): 444.0 $[M+H]^+$.

6-[(*S*)-3-({*cis*-3-fluorocyclobutyl}amino)-2-(4-{[4-(morpholinomethyl)phenyl]ethynyl}-phenyl)propyl]-5-hydroxypyrimidin-4(3H)-one (8p):

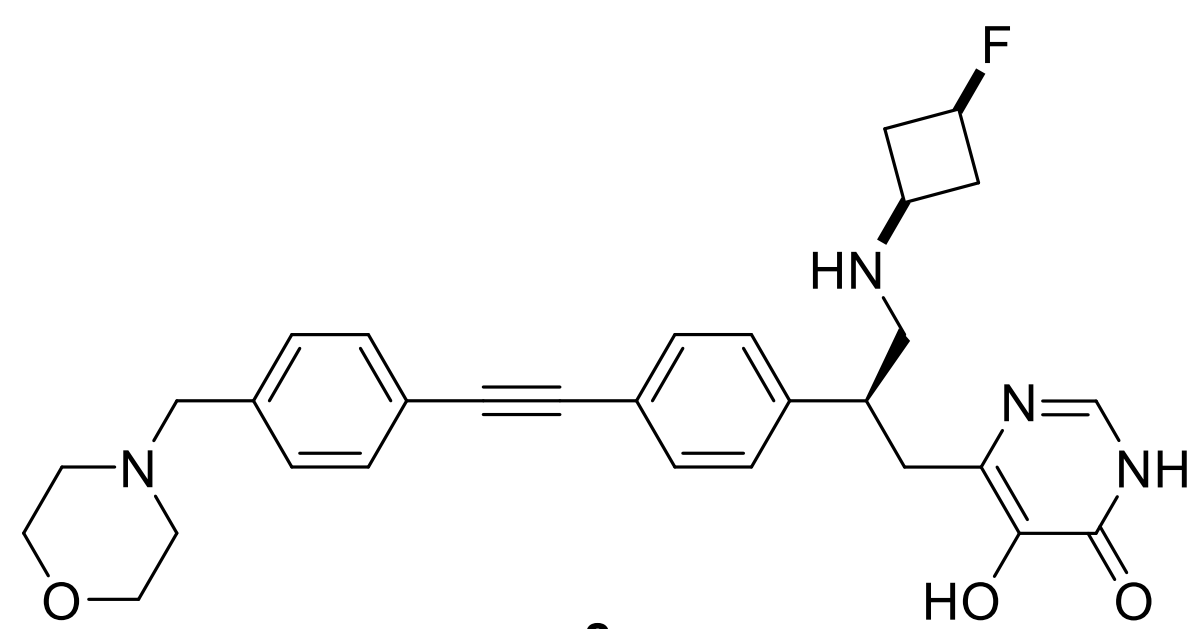

8p

Compound **8p** was prepared from **30** and **76** utilizing General Procedure B (30 mg, 21%). LC-MS (m/z): 517.2 $[M+H]^+$. ^{1}H-NMR (DMSO-d_6): δ 12.47 (br s, 1H), 9.22 (br s, 1H), 8.83 (br s, 1H), 7.69 (s, 1H), 7.69-7.40 (m, 4H), 7.33-6.99 (m, 4H), 4.97-4.79 (m, 1H), 3.70-3.50 (m, 6H), 3.35-3.20 (m, 4H), 3.00-2.80 (m, 2H), 2.70-2.60 (m, 3H), 2.40-2.25 (m, 5H).

Procedures for the preparation of Compound 8q

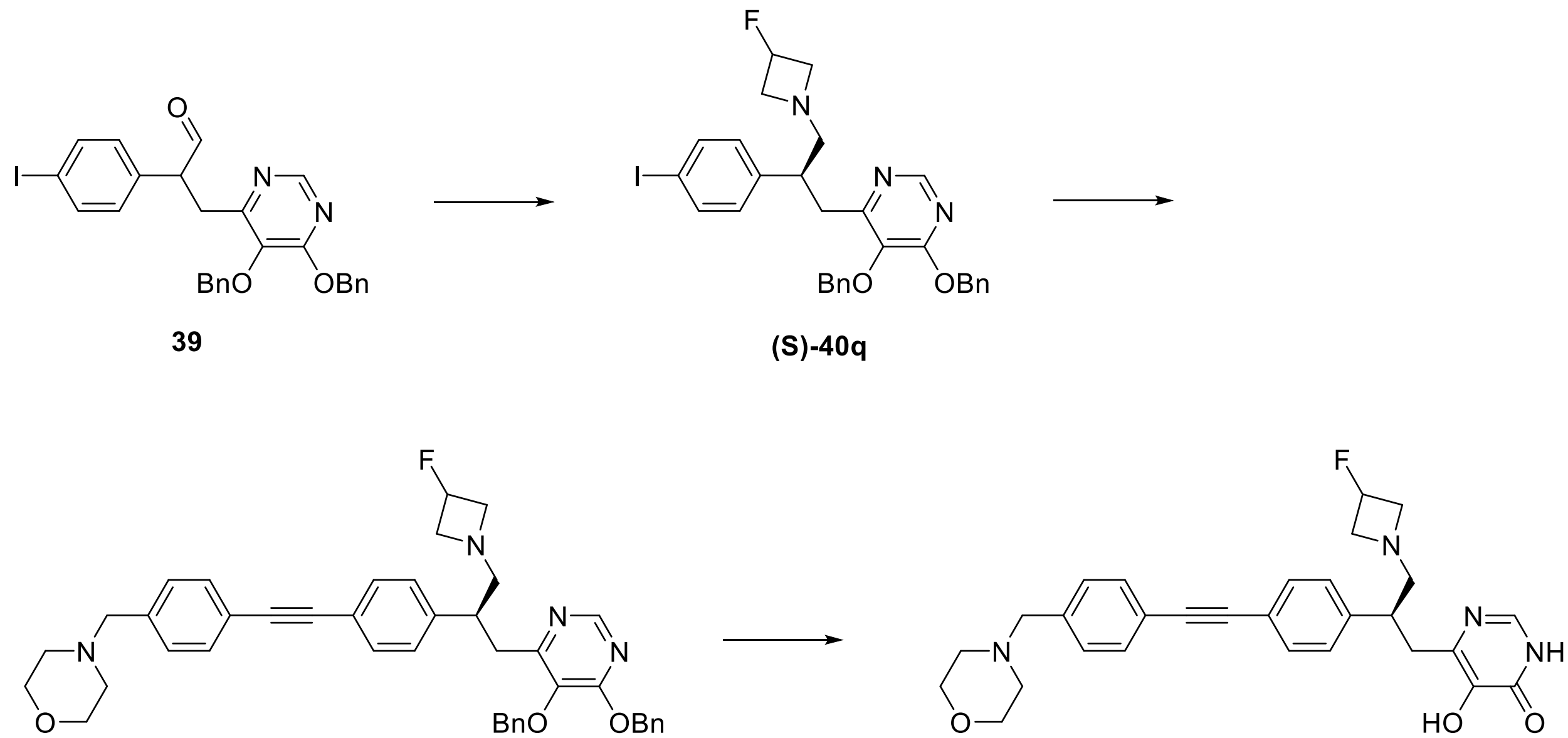

39 (S)-40q 77 8q

(*S*)-4,5-bis(benzyloxy)-6-[3-(3-fluoroazetidin-1-yl)-2-(4-iodophenyl)propyl]pyrimidine ((*S*)-40q):

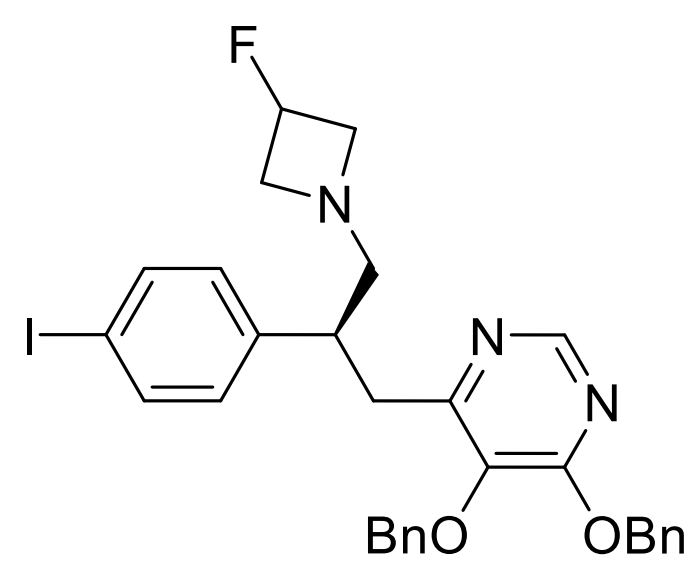

(S)-40q

Compound **(*S*)-40q** was prepared from **39** and 3-fluoroazetidine hydrochloride utilizing General Procedure F (11 g racemate, 68%) followed by SFC separation. LC-MS (m/z): 610.1 $[M+H]^+$.

(*S*)-4-{4-[(4-{1-[5,6-bis(benzyloxy)pyrimidin-4-yl]-3-[3-fluoroazetidin-1-yl]propan-2-yl}-phenyl)ethynyl]benzyl}morpholine (77):

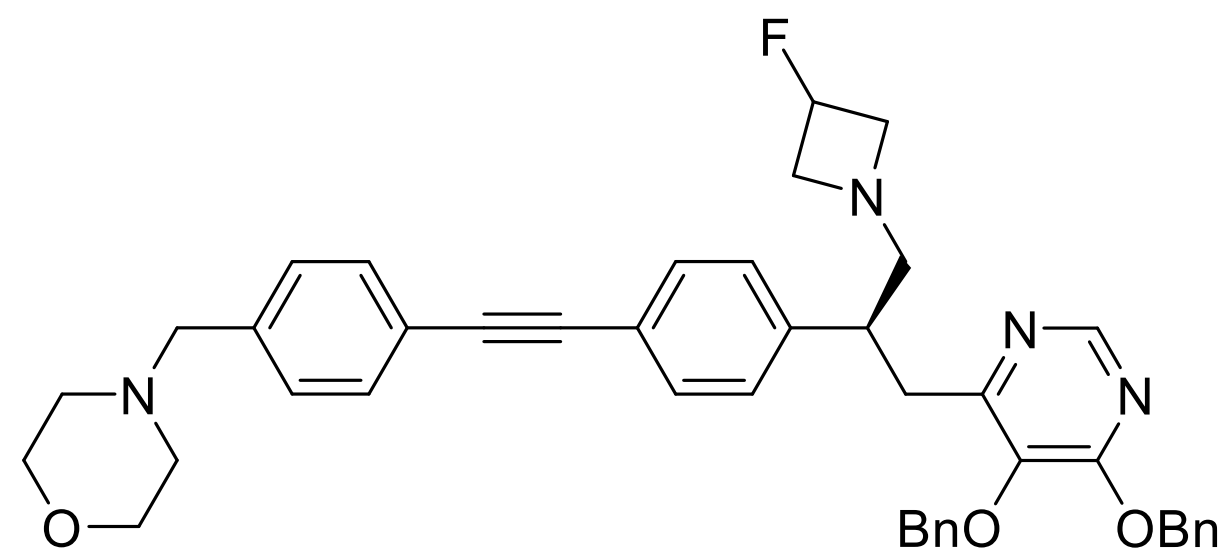

77

Compound **77** was prepared from **30** and **(*S*)-40q** utilizing General Procedure B (4.0 g, 81%). LC-MS (m/z): 683.3 $[M+H]^+$.

(*S*)-6-[3-(3-fluoroazetidin-1-yl)-2-(4-{[4-(morpholinomethyl)phenyl]ethynyl}phenyl)propyl]-5-hydroxypyrimidin-4(3H)-one (8q):

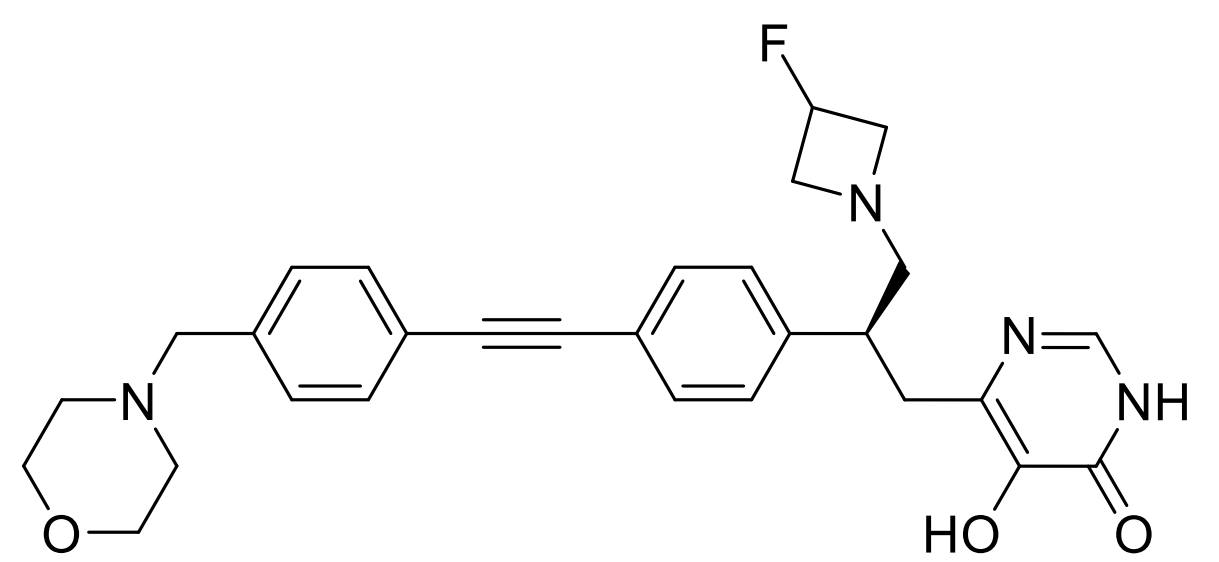

8q

Compound **77** was converted to **8q** utilizing General Procedure D (1.2 g, 61%). LC-MS (m/z): 501.2 $[M+H]^+$. ^{1}H-NMR (DMSO-d_6): δ 12.36 (s, 1H), 8.36 (s, 1H), 7.66 (s, 1H), 7.48 (d, J = 8.12 Hz, 2H), 7.40-7.34 (m, 4H), 7.22 (d, J = 8.0 Hz, 2H), 5.14-4.98 (m, 1H), 3.59-3.57 (m, 4H), 3.49-3.43 (m, 2H), 2.70-2.68 (m, 3H), 2.70-2.68 (m, 4H), 2.70-2.68 (m, 2H), 2.36-2.34 (m, 4H).

Procedures for the preparation of Compound 8r

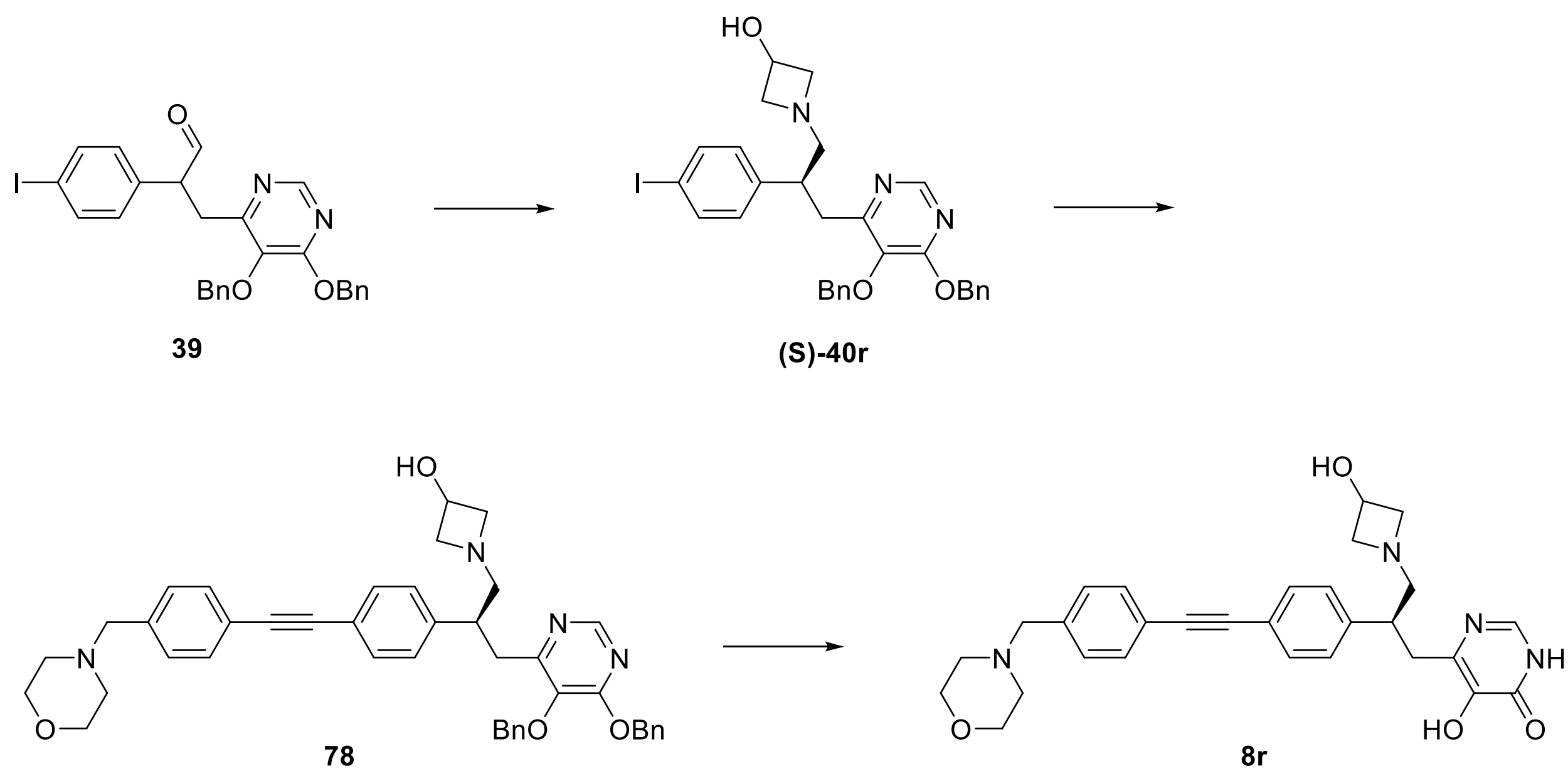

(*S*)-1-{3-[5,6-bis(benzyloxy)pyrimidin-4-yl]-2-[4-iodophenyl]propyl}azetidin-3-ol ((*S*)-40r):

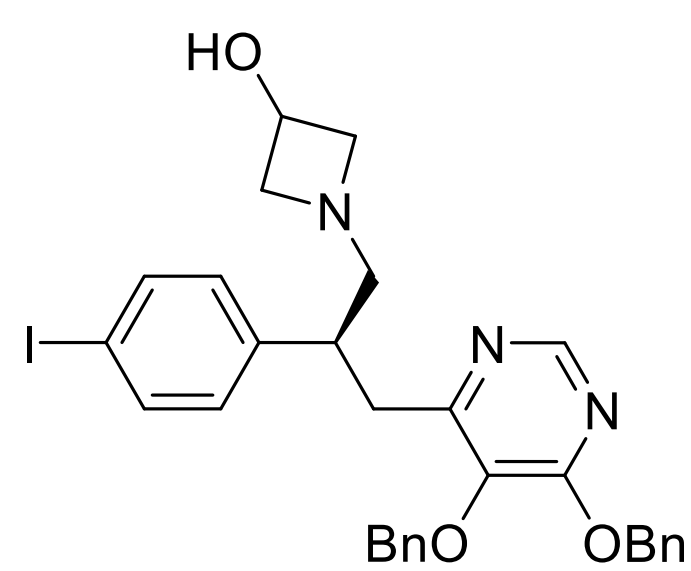

(S)-40r

Compound **(*S*)-40r** was prepared from **39** and 3-hydroxyazetidine hydrochloride utilizing General Procedure F (1.5 g racemate, 66%) followed by SFC separation. LC-MS (m/z): 608.0 $[M+H]^+$.

(*S*)-1-[3-(5,6-bis{benzyloxy}pyrimidin-4-yl)-2-(4-{[4-(morpholinomethyl)phenyl]ethynyl}-phenyl)propyl]azetidin-3-ol (78):

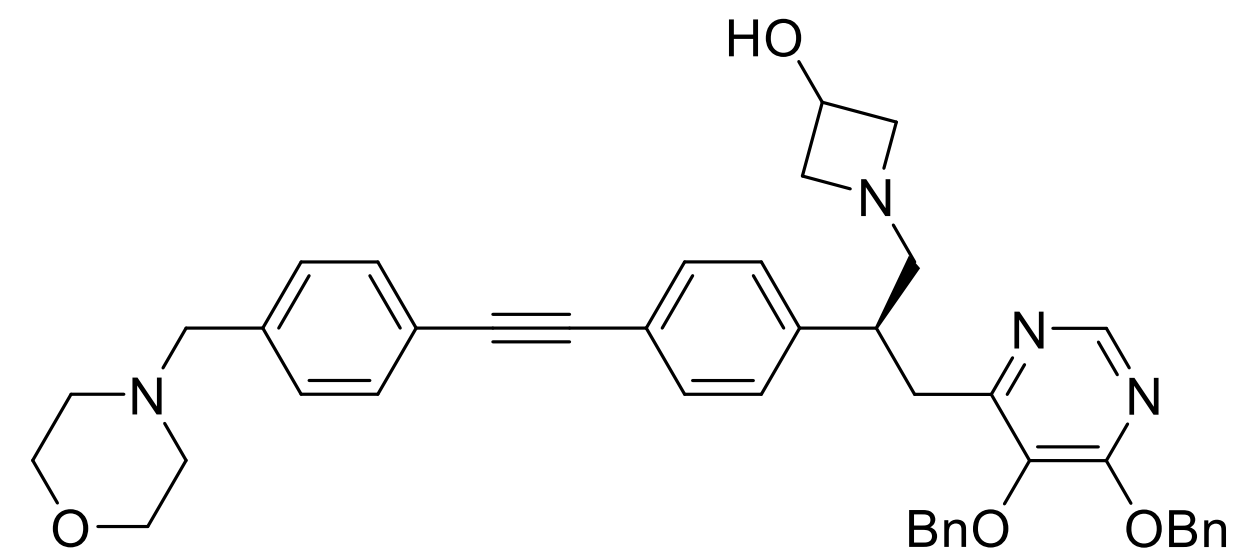

78

Compound 7**8** was prepared from **30** and **(*S*)-40r** utilizing General Procedure B (0.38 g, 75%). LC-MS (m/z): 681.3 $[M+H]^+$.

(*S*)-5-hydroxy-6-[3-(3-hydroxyazetidin-1-yl)-2-(4-{[4-(morpholinomethyl)phenyl]ethynyl}-phenyl)propyl]pyrimidin-4(3H)-one (8r):

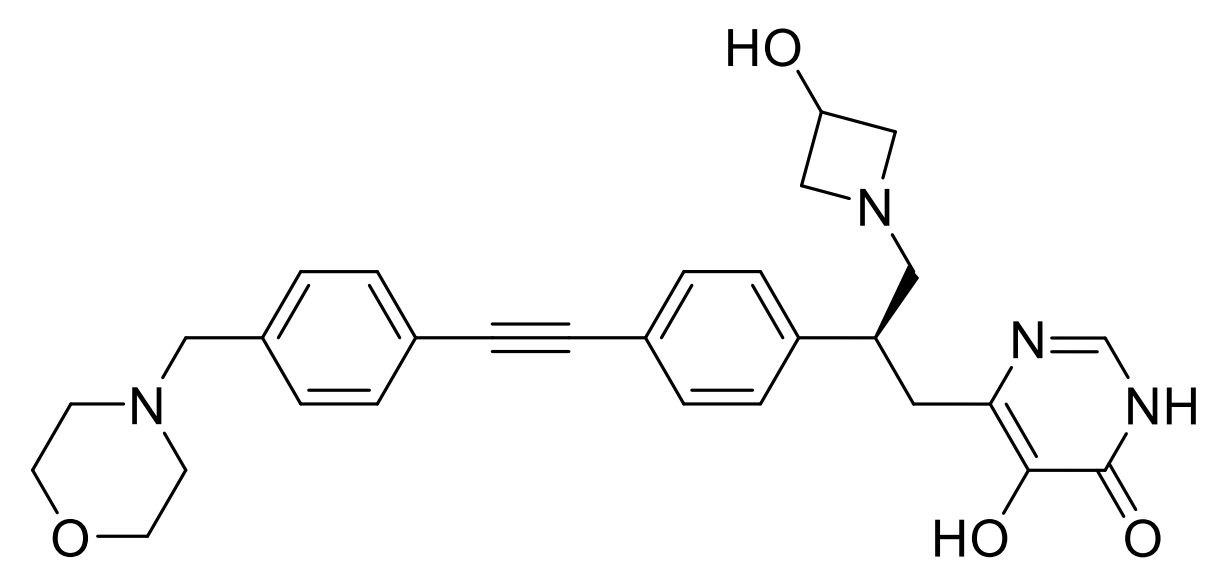

8r

Compound **78** was converted to **8r** utilizing General Procedure D (0.13 g, 45%). LC-MS (m/z): 501.3 $[M+H]^+$. ^{1}H-NMR (DMSO-d_6): δ 8.17 (s, 1H), 7.65 (s, 1H), 7.48 (d, J = 7.60 Hz, 2H), 7.39-7.34 (m, 4H), 7.20 (d, J = 8.00 Hz, 2H), 4.07-4.07 (m, 1H), 3.57-3.49 (m, 5H), 3.48-3.40 (m, 4H), 3.48-3.40 (m, 2H), 2.82-2.75 (m, 2H), 2.75-2.64 (m, 4H), 2.61-2.35 (m, 4H).

Procedures for the preparation of Compound 8s

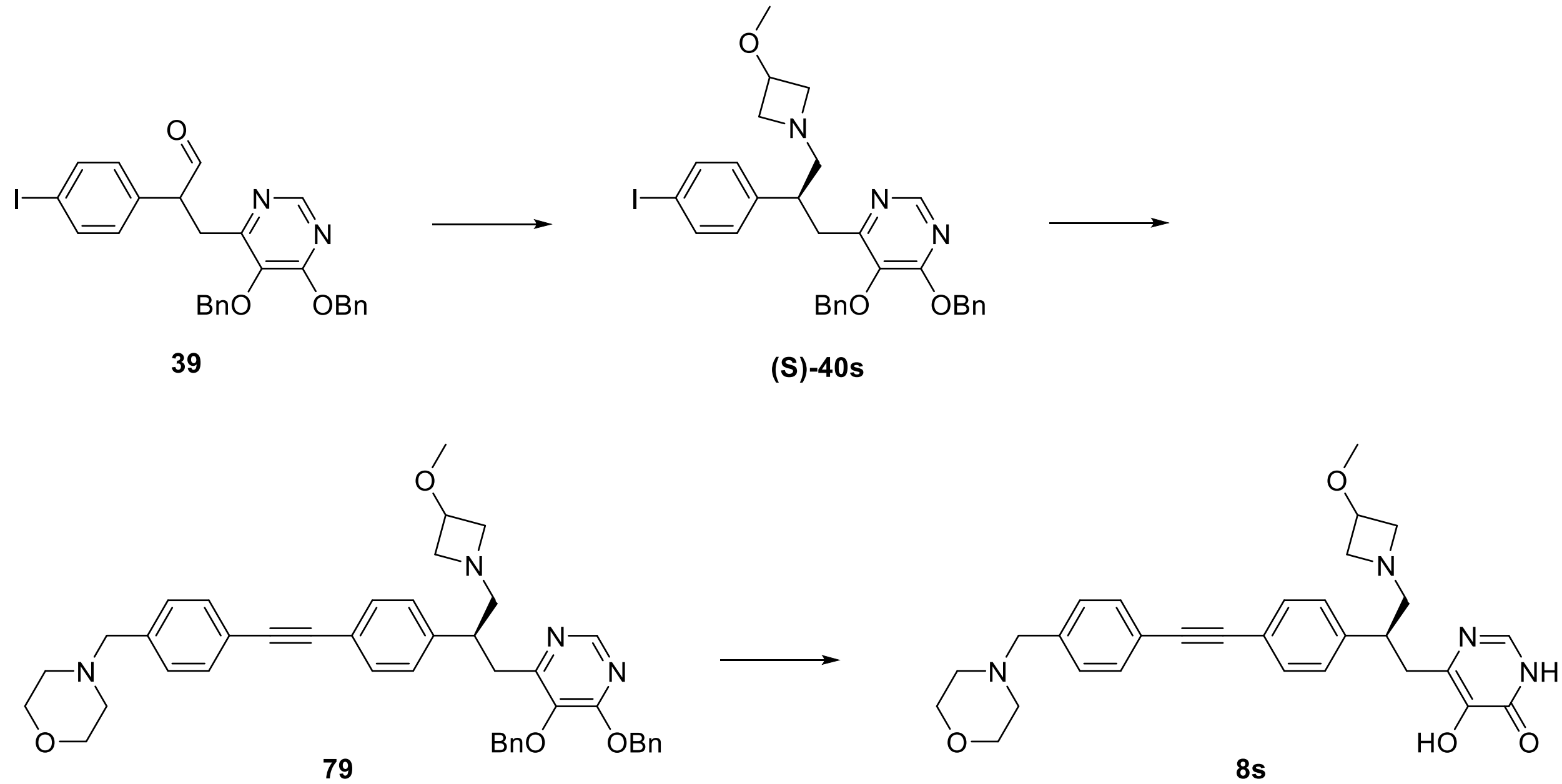

(*S*)-4,5-bis(benzyloxy)-6-[2-(4-iodophenyl)-3-(3-methoxyazetidin-1-yl)propyl]pyrimidine ((*S*)-40s):

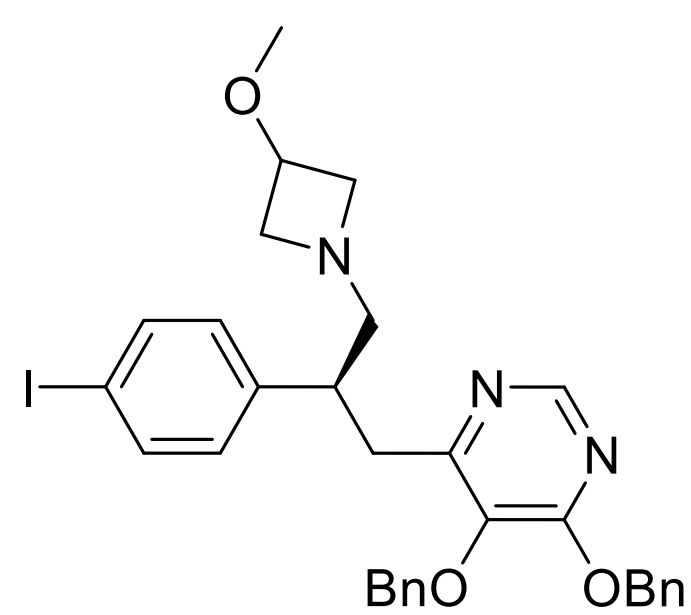

(S)-40s

Compound **(*S*)-40s** was prepared from **39** and 3-methoxyazetidine hydrochloride utilizing General Procedure F (19 g racemate, 67%) followed by SFC separation. LC-MS (m/z): 621.2 $[M+H]^+$.

(*S*)-4-{4-[(4-{1-[5,6-bis(benzyloxy)pyrimidin-4-yl]-3-[3-methoxyazetidin-1-yl]propan-2-yl}phenyl)ethynyl]benzyl}morpholine (79):

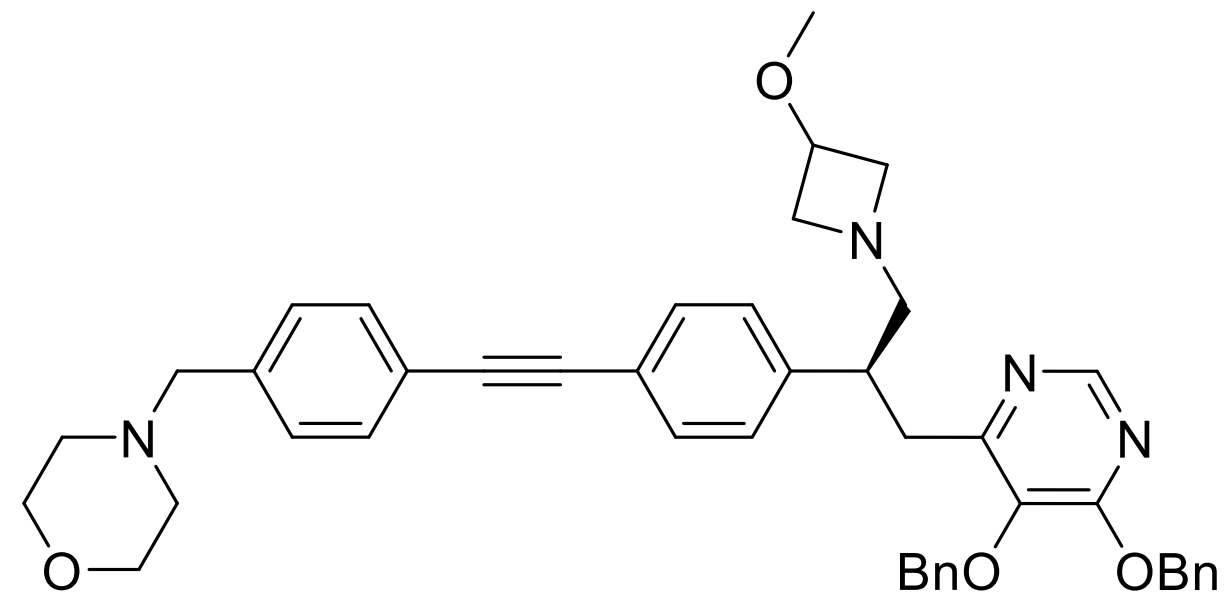

79

Compound **79** was prepared from **30** and **(*S*)-40s** utilizing General Procedure B (6.5 g, 85%). LC-MS (m/z): 695.3 $[M+H]^+$.

(*S*)-5-hydroxy-6-[3-(3-methoxyazetidin-1-yl)-2-(4-{[4-(morpholinomethyl)phenyl]ethynyl}-phenyl)propyl]pyrimidin-4(3H)-one (8s):

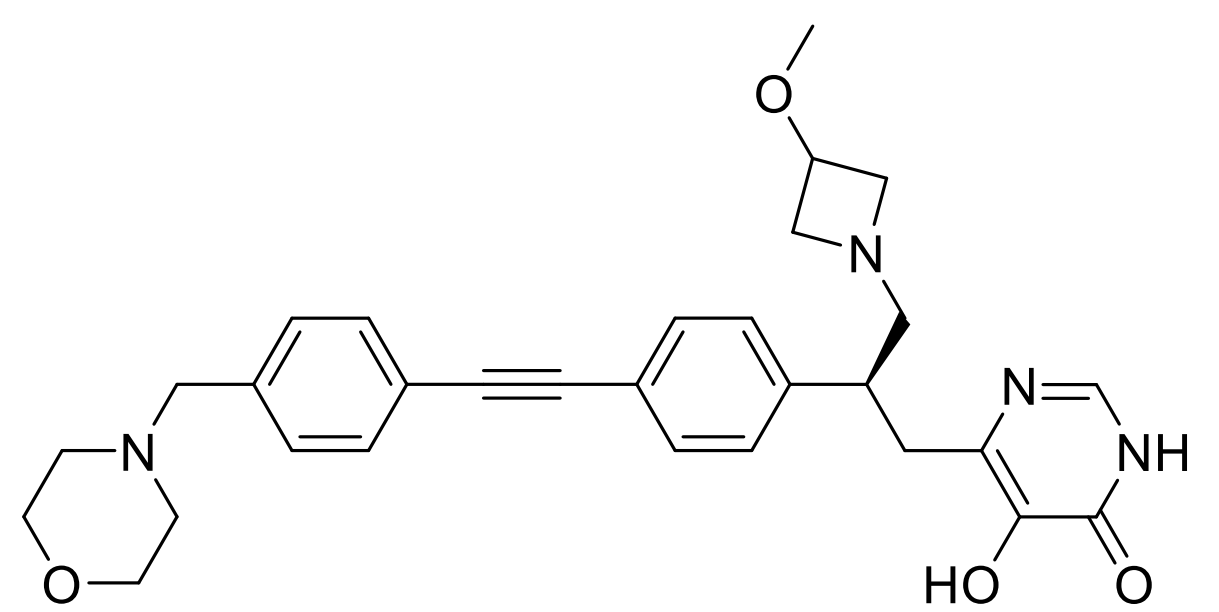

8s

Compound **79** was converted to **8s** utilizing General Procedure D (2.8 g, 58%). LC-MS (m/z): 515.3 $[M+H]^+$. ^{1}H-NMR (DMSO-d_6): δ 12.27 (br s, 1H), 7.65 (br s, 1H), 7.48 (d, J = 8.0 Hz, 2H), 7.39-7.34 (m, 4H), 7.20 (d, J = 8.0 Hz, 2H), 3.87-3.84 (m, 1H), 3.59-3.57 (m, 4H), 3.57 (s, 2H), 3.45-3.42 (m, 1H), 3.42-3.27 (m, 4H), 3.19-3.15 (m, 1H), 3.09 (s, 3H), 2.87-2.82 (m, 2H), 2.75-2.69 (m, 3H), 2.40-2.35 (m, 4H).

Procedures for the preparation of Compound 8t

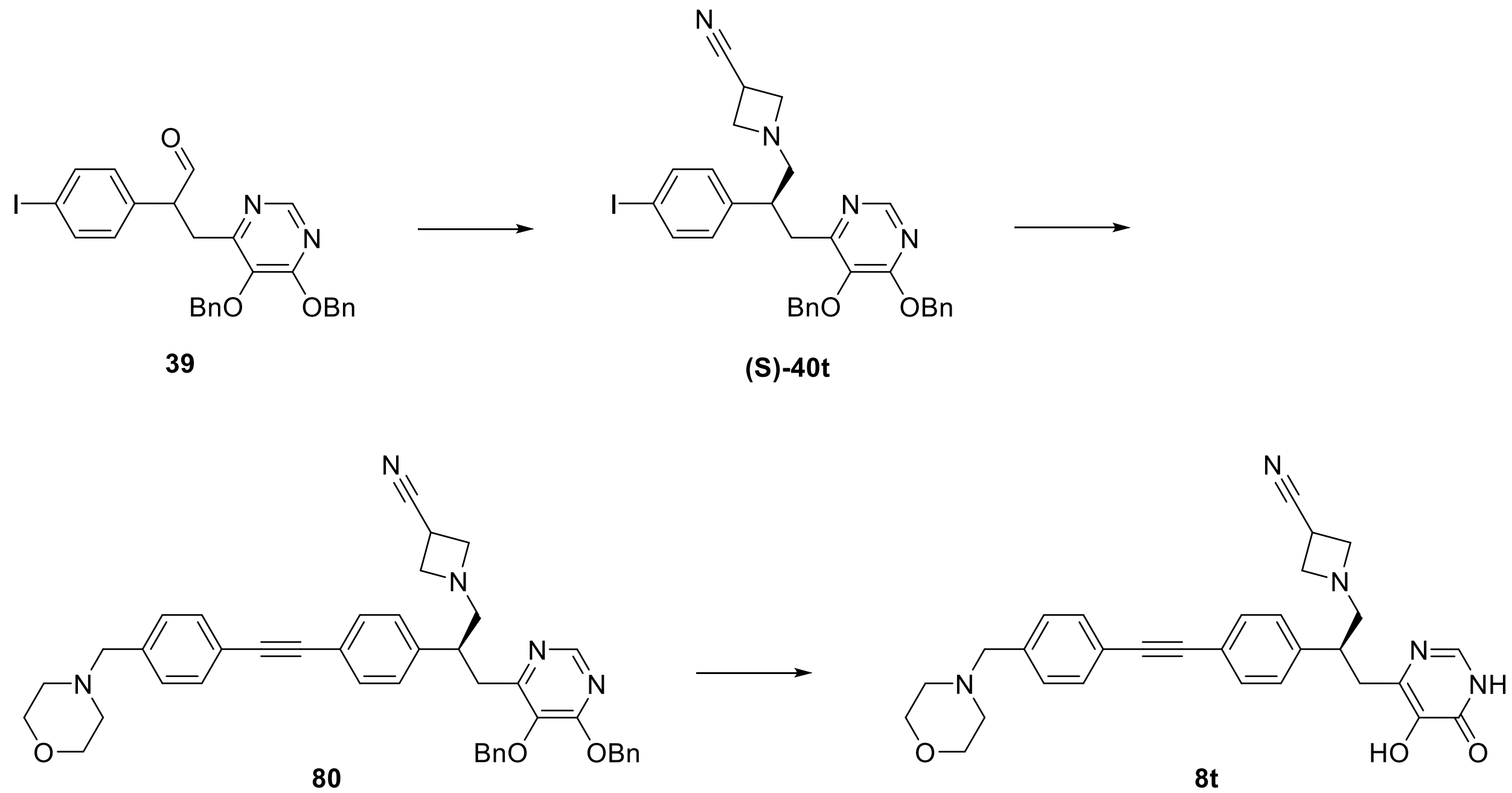

(*S*)-1-{3-[5,6-bis(benzyloxy)pyrimidin-4-yl]-2-[4-iodophenyl]propyl}azetidine-3-carbonitrile ((*S*)-40t):

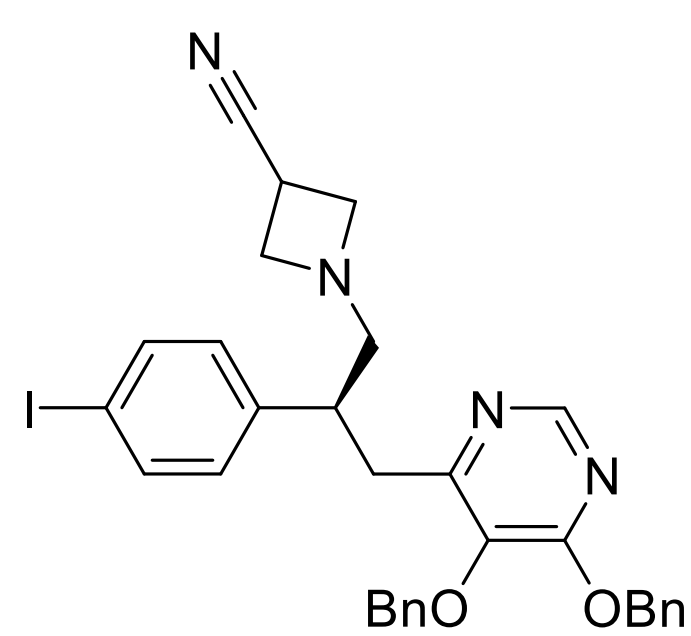

(S)-40t

Compound **(S)-40t** was prepared from **39** and azetidine-3-carbonitrile hydrochloride utilizing General Procedure F (3.8 g racemate, 68%) followed by SFC separation. LC-MS (m/z): 617.2 $[M+H]^+$.

(*S*)-1-[3-(5,6-bis{benzyloxy}pyrimidin-4-yl)-2-(4-{[4-(morpholinomethyl)phenyl]ethynyl}-phenyl)propy]azetidine-3-carbonitrile (80):

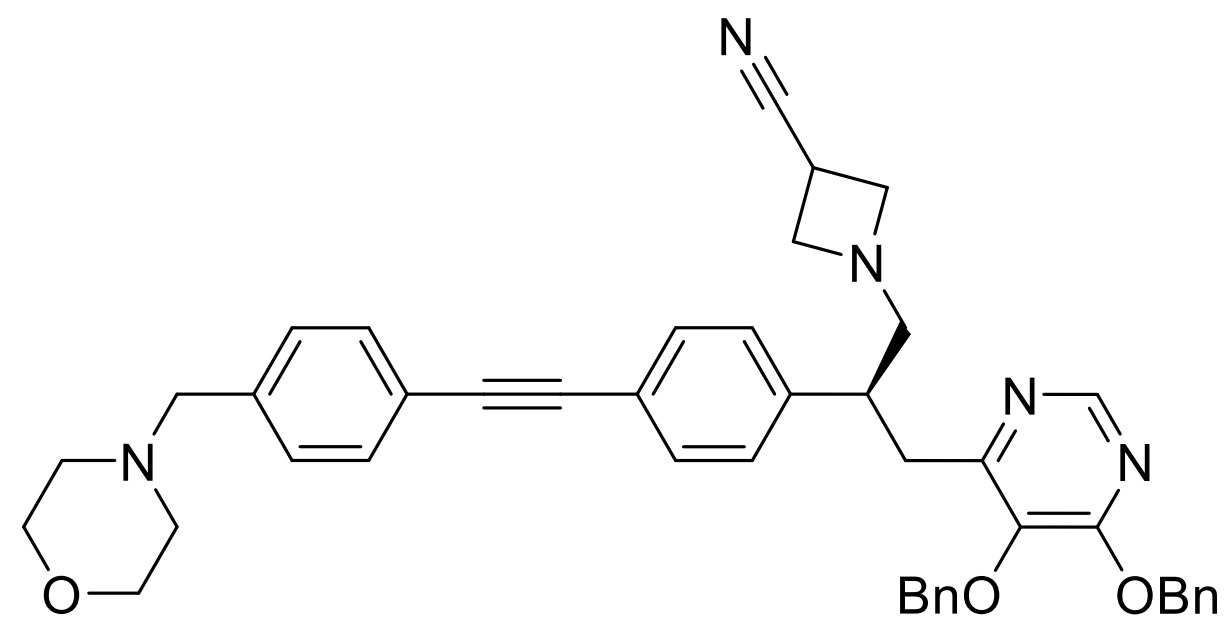

80

Compound **80** was prepared from **30** and **(*S*)-40t** utilizing General Procedure B (0.17 g, crude). LC-MS (m/z): 689.3 $[M+H]^+$.

(*S*)-1-[3-(5-hydroxy-6-oxo-1,6-dihydropyrimidin-4-yl)-2-(4-{[4-(morpholinomethyl)phenyl]-ethynyl}phenyl)propyl]azetidine-3-carbonitrile (8t):

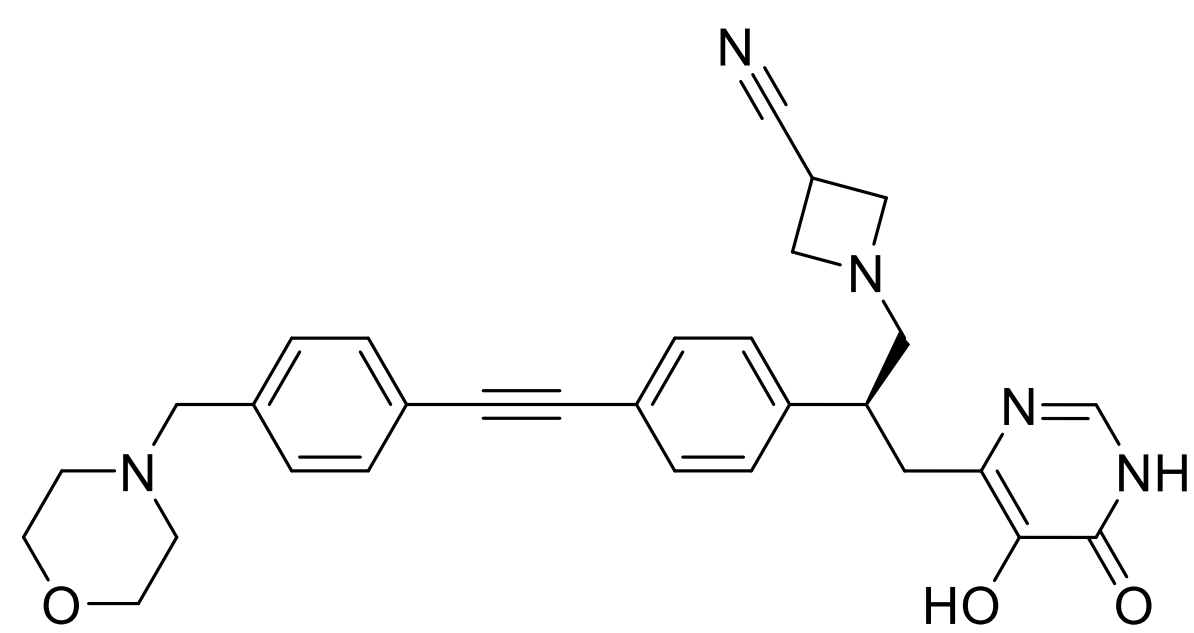

8t

Compound **80** was converted to **8t** utilizing General Procedure C (47 mg, 38% over two steps). LC-MS (m/z): 510.3 $[M+H]^+$. ^{1}H-NMR (DMSO-d_6): δ 12.38 (br s, 1H), 9.07 (br s, 1H), 8.14 (s, 1H), 7.66 (s, 1H), 7.49 (d, J = 8.0 Hz, 2H), 7.38 (d, J = 8.0 Hz, 4H), 7.22 (d, J = 8.0 Hz, 2H), 3.60-3.59 (m, 3H), 3.60-3.50 (m, 2H), 3.45 (s, 2H), 3.35-3.25 (m, 1H), 3.20-3.05 (m, 3H), 2.90-2.80 (m, 1H), 2.80-2.70 (m, 1H), 2.70-2.60 (m, 2H), 2.40-2.30 (m, 4H).

Procedures for the preparation of Compound 8u

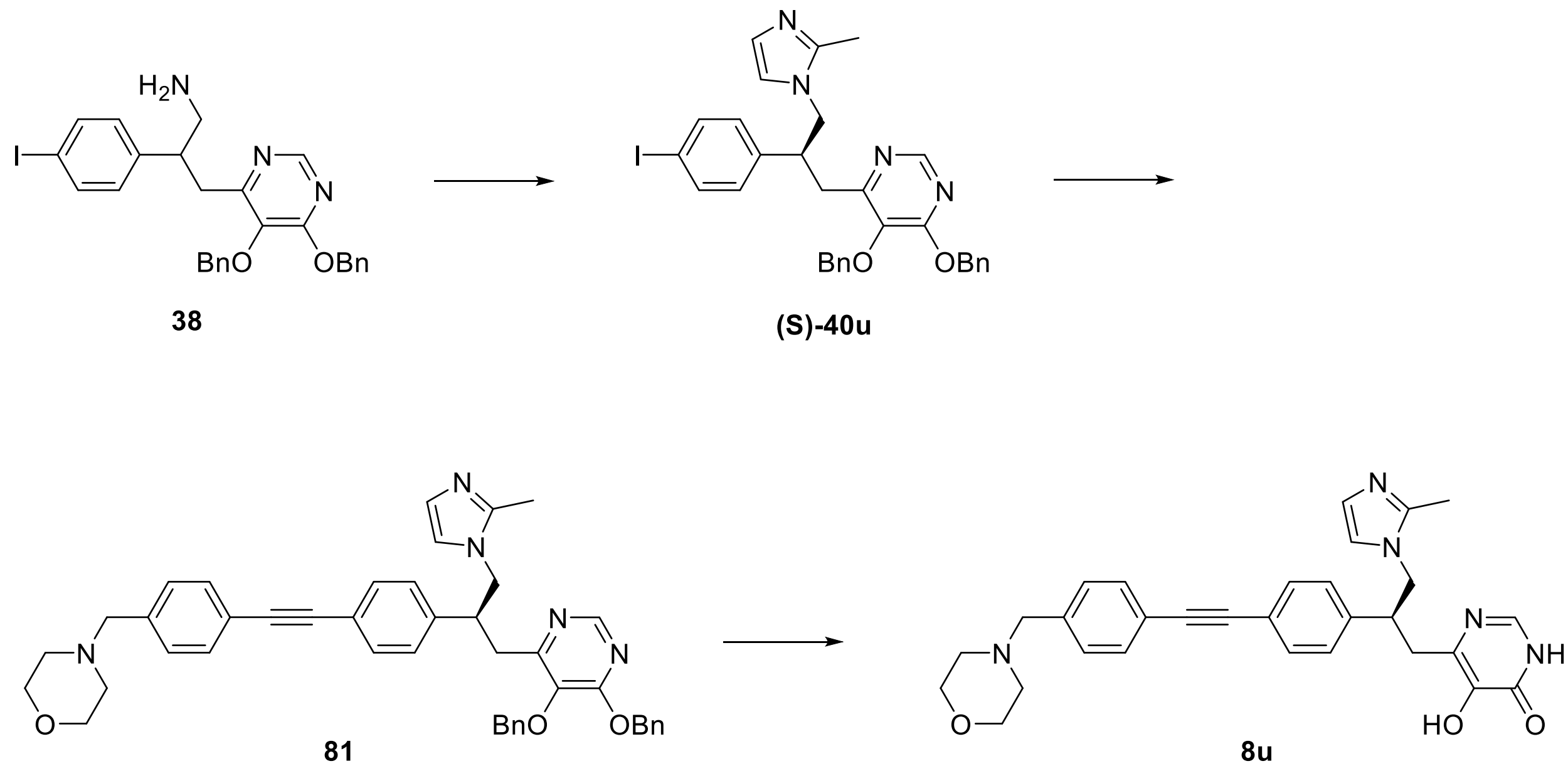

(*S*)-4,5-bis(benzyloxy)-6-[2-(4-iodophenyl)-3-(2-methyl-1H-imidazol-1-yl)propyl]pyrimidine ((S)-40u):

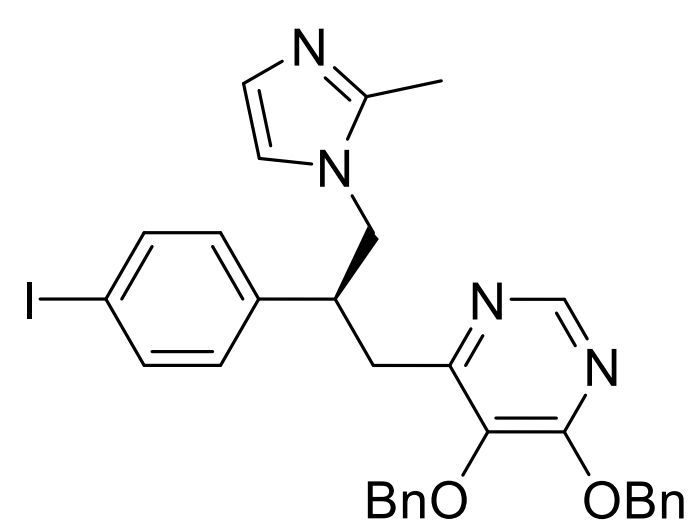

(S)-40u

To a solution of acetaldehyde (50 mg, 1.2 mmol) in MeOH (5 mL), **38** (600 mg, 1.0 mmol), ammonium acetate (85 mg, 1.2 mmol) and aqueous 40% glyoxal solution (150 mg, 1.2 mmol) were added and stirred at room temperature for 16 h. After consumption of starting material, the solvent was removed under reduced pressure. The residue dissolved in water (20 mL) and extracted with EtOAc (2 x 10 mL). The combined organic layers were washed with brine. The organic layer was dried over anhydrous Na_2SO_4 and concentrated under reduced pressure. The crude product was purified silica gel chromatography (50 - 60% EtOAc in pet. ether) to get **40u**. The two

enantiomers were separated by SFC before moving on to the next step. LC-MS (m/z): 617.2 $[M+H]^+$.

(*S*)-4-{4-[(4-{1-[5,6-bis(benzyloxy)pyrimidin-4-yl]-3-[2-methyl-1H-imidazol-1-yl]propan-2-yl}phenyl)ethynyl]benzyl}morpholine (81):

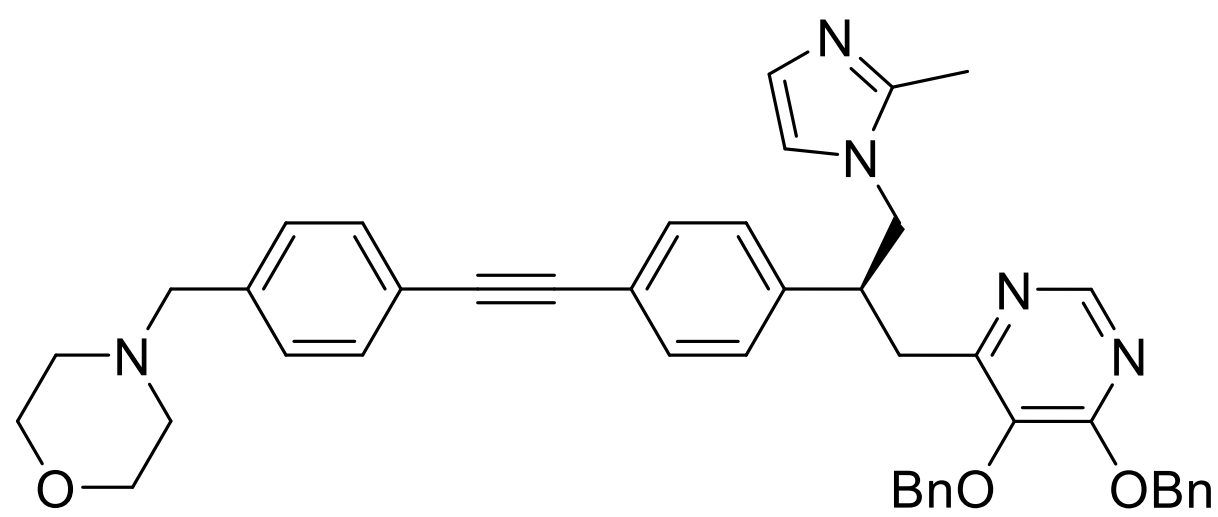

81

Compound **81** was prepared from **30** and **(*S*)-40u** utilizing General Procedure B (100 mg, 76%). LC-MS (m/z): 689.3 $[M+H]^+$.

(*S*)-5-hydroxy-6-[3-(2-methyl-1H-imidazol-1-yl)-2-(4-{[4-(morpholinomethyl)phenyl]-ethynyl}phenyl)propyl]pyrimidin-4(3H)-one (8u):

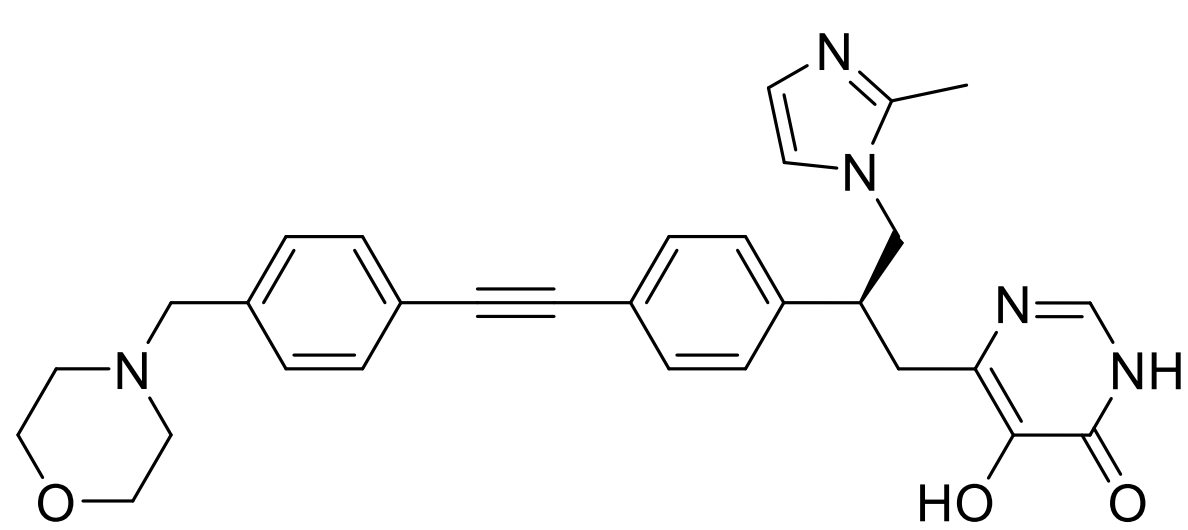

8u

Compound **81** was converted to **8u** utilizing General Procedure D (15 mg, 17%). LC-MS (m/z): 510.3 $[M+H]^+$. ^{1}H-NMR (DMSO-d_6): δ 12.35 (br s, 1H), 9.05 (br s, 1H), 8.19 (s, 1H), 7.67 (s, 1H), 7.47 (d, J = 7.6 Hz, 2H), 7.37-7.33 (m, 4H), 7.15 (d, J = 8.4 Hz, 2H), 6.88 (s, 1H), 6.62 (s, 1H), 4.42-4.09 (m, 2H), 3.58-3.56 (m, 5H), 3.50-3.40 (m, 2H), 3.05-2.95 (m, 1H), 2.85-2.75 (m, 1H), 2.35-2.25 (m, 4H), 1.97 (s, 3H).

Procedures for the preparation of Compound 8v

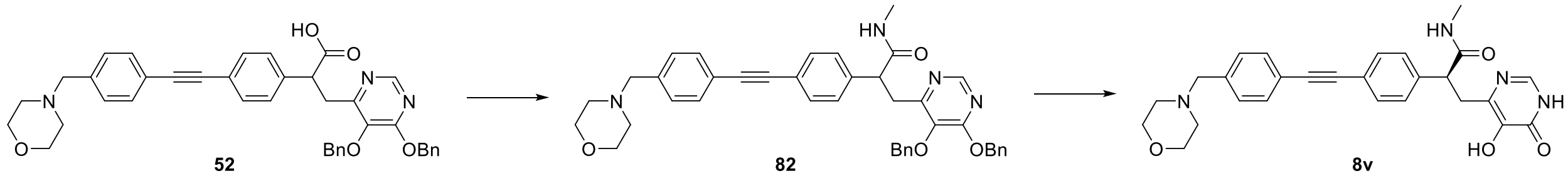

3-[5,6-bis(benzyloxy)pyrimidin-4-yl]-*N*-methyl-2-(4-{[4-(morpholinomethyl)phenyl]-ethynyl}phenyl)propanamide (82):

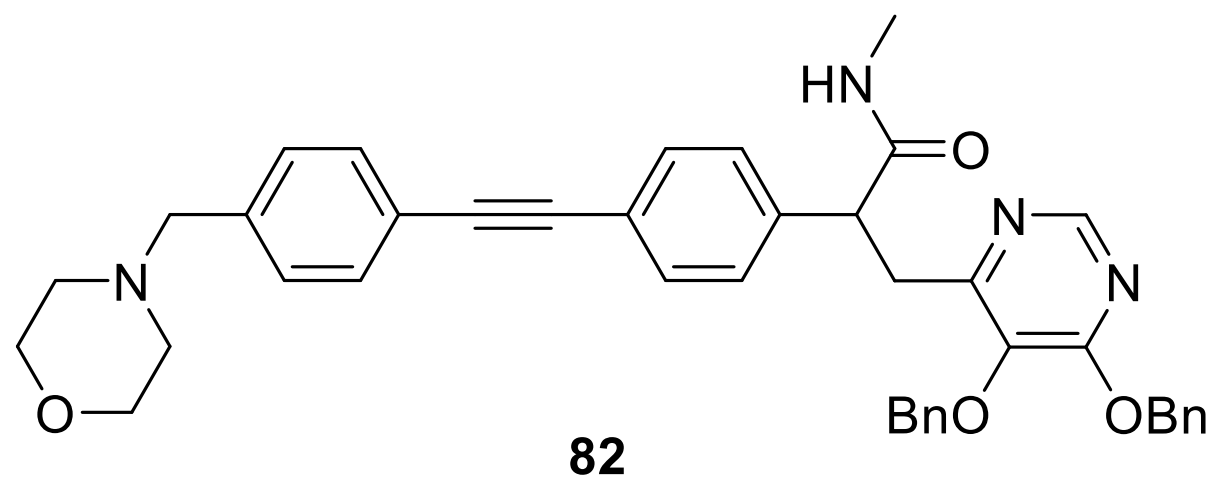

To a solution of **52** (0.25 g, 0.00039 mol) in DMF (5 mL), methyl amine (2 M in THF 0.097 mL, 0.22 g, 0.0058 mol), HATU (0.22 g, 0.0058 mol) and DIPEA (0.10 g, 0.00078 mol) were added at 25 °C and stirred for 2 h. After completion of the reaction, the reaction mixture was diluted with water and extracted with EtOAc (2 x 10 mL). The combined organic layers were dried over Na_2SO_4, filtered, concentrated under reduced pressure (0.3 g, crude). LC-MS (m/z): 653.3 $[M+H]^+$.

3-(5-hydroxy-6-oxo-1,6-dihydropyrimidin-4-yl)-*N*-methyl-2-(4-{[4-(morpholinomethyl)-phenyl]ethynyl}phenyl)propanamide (8v):

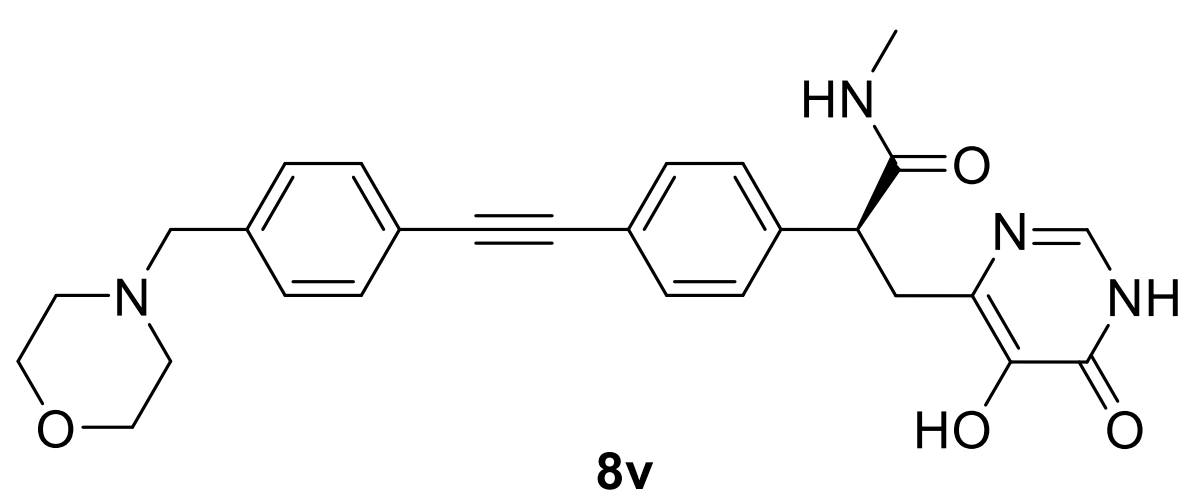

Crude **82** was converted to **8v** utilizing General Procedure C (21 mg, 11% over two steps). LC-MS (m/z): 473.2 $[M+H]^+$. ^{1}H-NMR (DMSO-d_6): δ 12.71 (br s, 1H), 12.39 (br s, 1H), 9.08 (m, 1H), 8.13 (s, 1H), 8.02 (d, J = 4.56 Hz, 1H), 7.64 (s, 1H), 7.48 (d, J = 7.8 Hz, 2H), 7.41 (d, J = 8.0 Hz,

2H), 7.33 (d, J = 8.1 Hz, 4H), 4.07 (t, J = 7.8 Hz, 1H), 5.65-3.55 (m, 4H), 3.55-3.45 (m, 2H), 3.10-2.90 (m, 2H), 2.45-2.30 (m, 4H).

Procedures for the preparation of Compound 8w

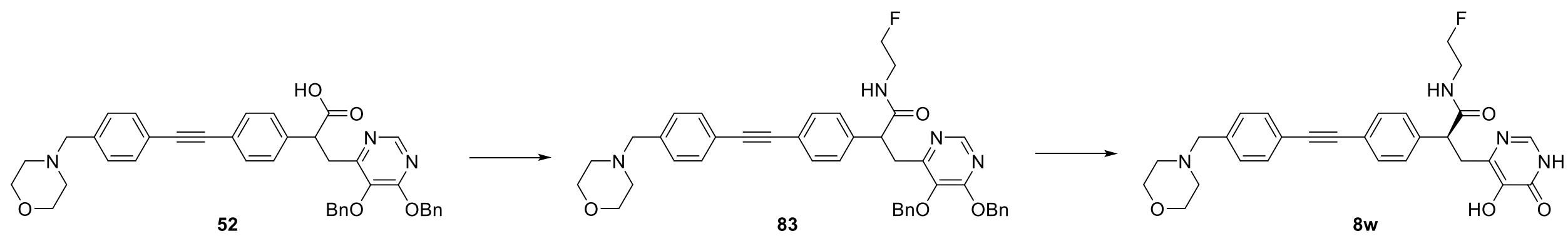

3-[5,6-bis(benzyloxy)pyrimidin-4-yl]-N-(2-fluoroethyl)-2-(4-{[4-(morpholinomethyl)-phenyl]ethynyl}phenyl)propanamide (83):

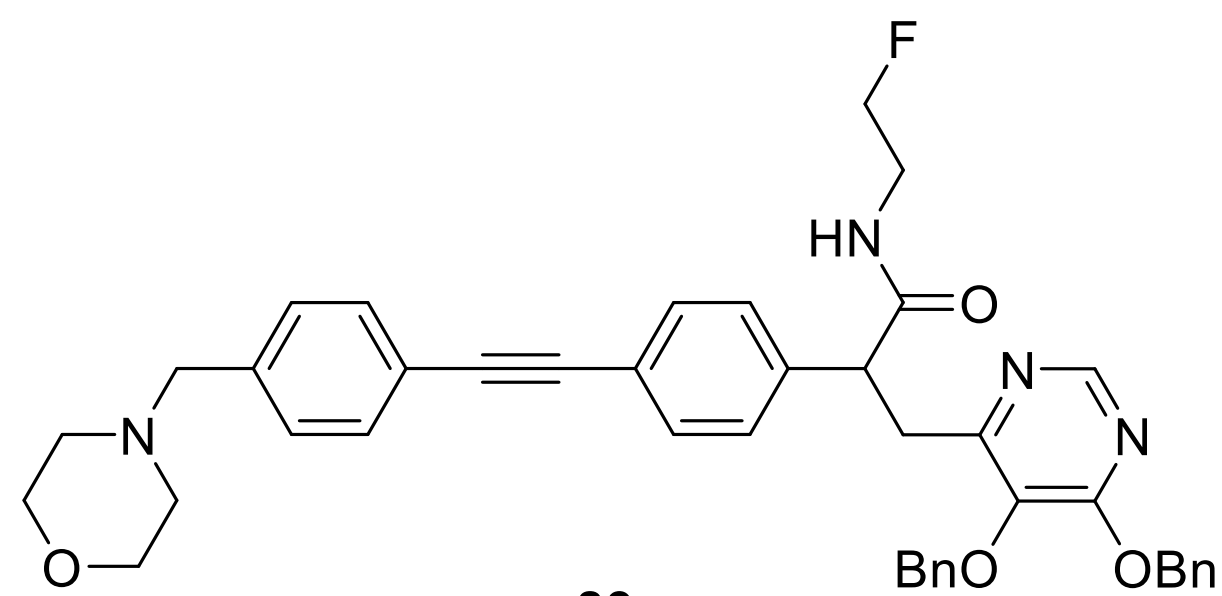

83

To a solution of **52** (0.25 g, 0.00039 mol) in DMF (5 mL), 2-fluoroethan-1-amine hydrochloride (0.077 g, 0.00078 mol), HATU (0.22g, 0.0058 mol), and DIPEA (0.10 g, 0.00078 mol) were added at 25 °C and stirred for 2 h. After completion of the reaction, the reaction mixture was diluted with water and extracted with EtOAc (2 x 10 mL). The combined organic layers were dried over Na_2SO_4, filtered, concentrated under reduced pressure (0.3 g, crude). LC-MS (m/z): 685.2 $[M+H]^+$.

(*S*)-N-(2-fluoroethyl)-3-(5-hydroxy-6-oxo-1,6-dihydropyrimidin-4-yl)-2-(4-{[4-(morpholinomethyl)phenyl]ethynyl}phenyl)propanamide (8w):

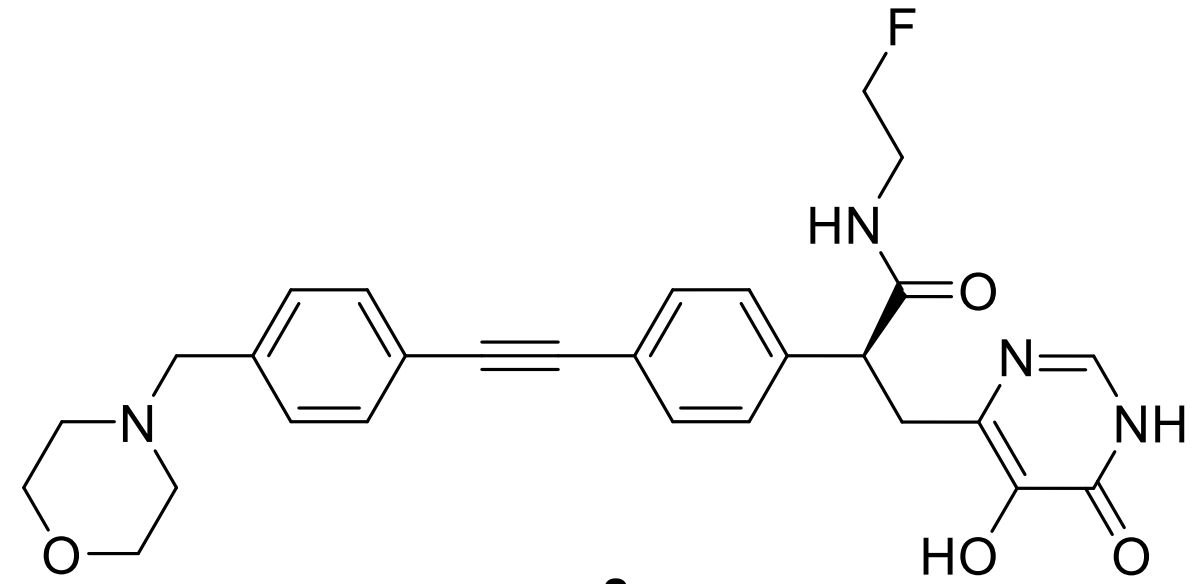

8w

Crude **83** was converted to **rac-8w** utilizing General Procedure C (61 mg, 25% over two steps). The mixture was separated by SFC to yield **8w**. LC-MS (m/z): 505.3 $[M+H]^+$. ^{1}H-NMR (DMSO-d_6): δ 8.41 (br s, 1H), 7.62 (br s, 1H), 7.55-7.45 (m, 3H), 7.45-7.40 (m, 2H), 7.40-7.30 (m, 5H), 4.41 (d, J = 5.2 Hz, 1H), 4.29 (d, J = 4.8 Hz, 1H), 4.20-4.10 (m, 1H), 3.93 (d, J = 5.7 Hz, 1H), 3.65-3.55 (m, 3H), 3.55-3.45 (m, 2H), 3.05-2.95 (m, 2H), 2.40-2.25 (m, 6H).

Procedures for the preparation of Compound 9k

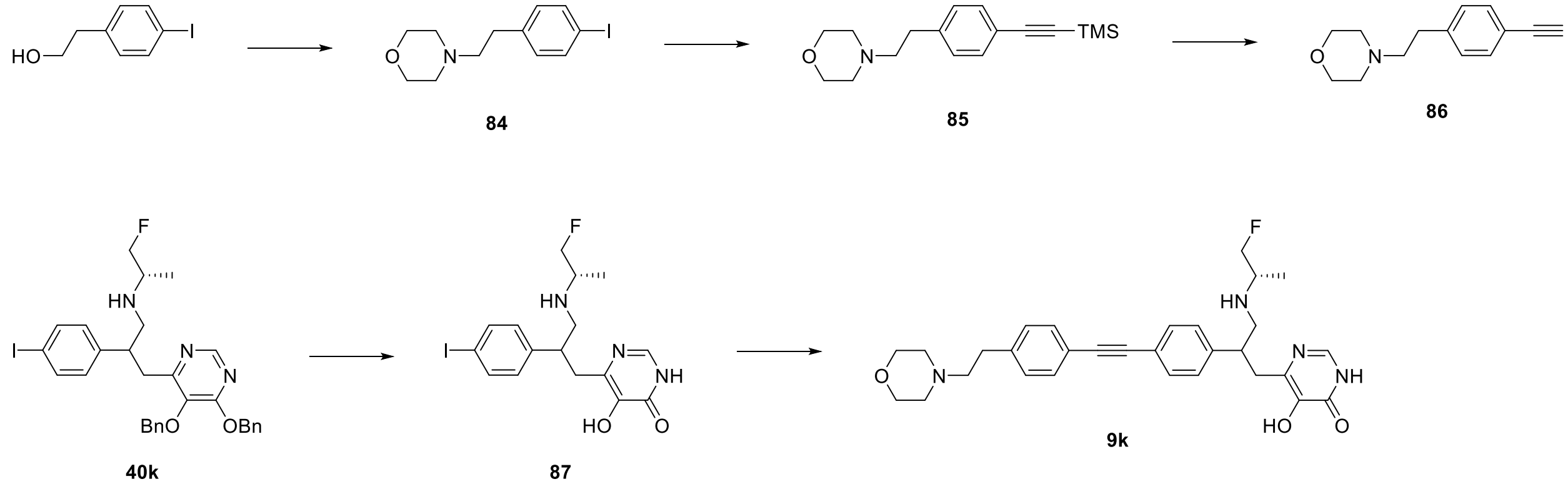

4-(4-iodophenethyl)morpholine (84):

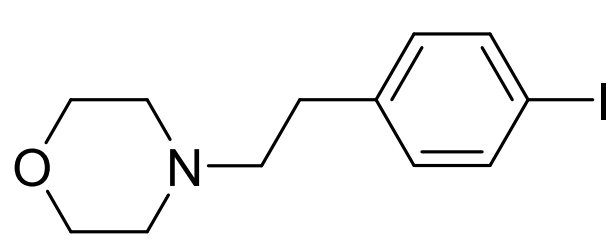

84

To a 0 °C cooled solution of 2-(4-iodophenyl)ethan-1-ol (2.00 g, 8.06 mmol) in dry DCM (40 mL), was added Et_3N (3.36 mL, 24.2 mmol), mesyl chloride (0.93 mL, 12 mmol) and the reaction

mixture was stirred for 1 h. After completion of the reaction, water (50 mL) was added and organic layer was separated, dried over Na_2SO_4, filtered and concentrated under reduced pressure to obtain crude mesylate. This residue was then taken up in DMF (40 mL), followed by the addition of K_2CO_3 (2.2 g, 15 mmol) and morpholine (0.80 g, 9.6 mmol), and the reaction mixture was stirred at 25 °C for 3 h. After the consumption of SM by TLC, ice was added to the reaction mass and the obtained solid was filtered and dried under reduced pressure to get **84** (2.1 g, 88%). LC-MS (m/z): 318.2 $[M+H]^+$.

4-(4-[(trimethylsilyl)ethynyl)phenethyl]morpholine (85):

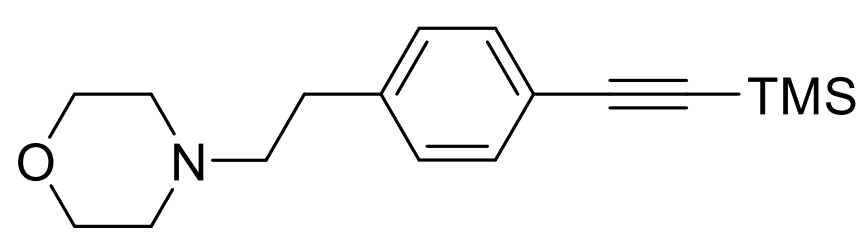

85

Compound **85** was obtained from **84** and ethynyltrimethylsilane utilizing General Procedure B (1.4 g, 78%). LC-MS: product did not ionize.

4-(4-ethynylphenethyl)morpholine (86):

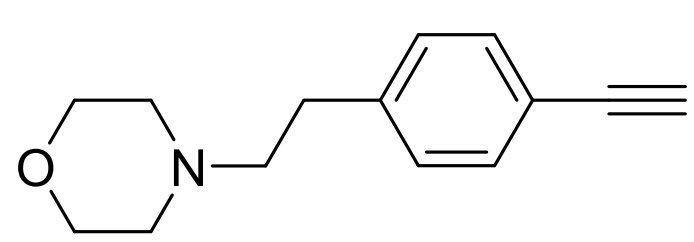

86

Compound **86** was obtained from **85** utilizing General Procedure I (0.40 g, 81%). LC-MS (m/z): 216.3 $[M+H]^+$.

6-{3-[((*S*)-1-fluoropropan-2-yl)amino]-2-[4-iodophenyl]propyl}-5-hydroxypyrimidin-4(3H)-one (87):

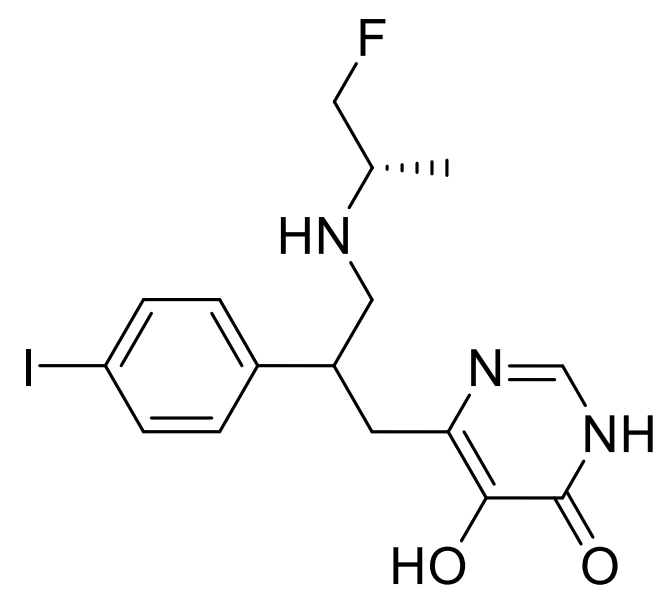

87

Compound **87** was obtained from **40k** utilizing General Procedure C (0.20 g, 94%). LC-MS (m/z): 432.1 $[M+H]^+$.

6-[3-({(*S*)-1-fluoropropan-2-yl}amino)-2-(4-{[4-(2-morpholinoethyl)phenyl]ethynyl}-phenyl)propyl]-5-hydroxypyrimidin-4(3H)-one (9k):

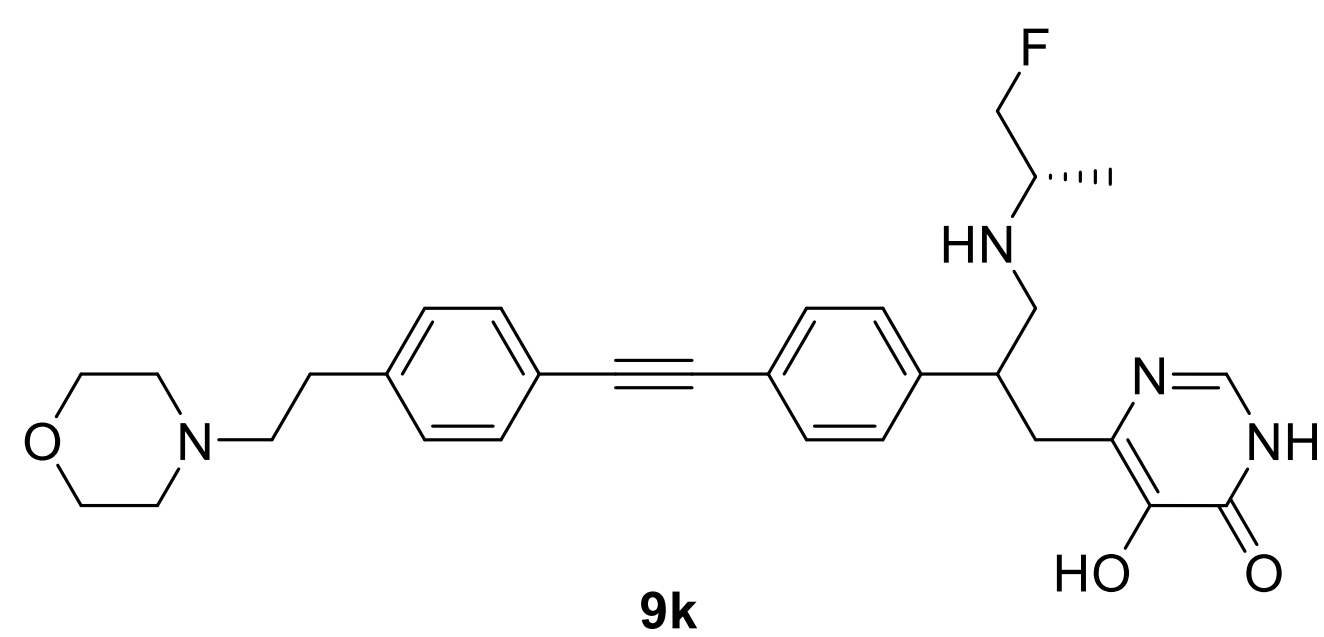

9k

Compound **9k** was obtained from **86** and **87** utilizing General Procedure B with DMF as the solvent (95 mg, 40%). LC-MS (m/z): 519.3 $[M+H]^+$. ^{1}H-NMR (DMSO-d_6): δ 12.42 (br s, 1H), 9.18 (br s, 1H), 8.14 (br s, 1H), 7.69 (s, 1H), 7.45 (d, J = 7.68 Hz, 4H), 7.30 (t, J = 7.76 Hz, 4H), 4.63-4.42 (m, 2H), 3.61 (m, 6H), 3.51 (m, 2H), 2.91-2.90 (m, 2H), 2.81-2.79 (m, 3H), 2.55-2.51 (m, 4H), 1.16 (d, J = 5.92 Hz, 3H).

Procedures for the preparation of Compound 10k

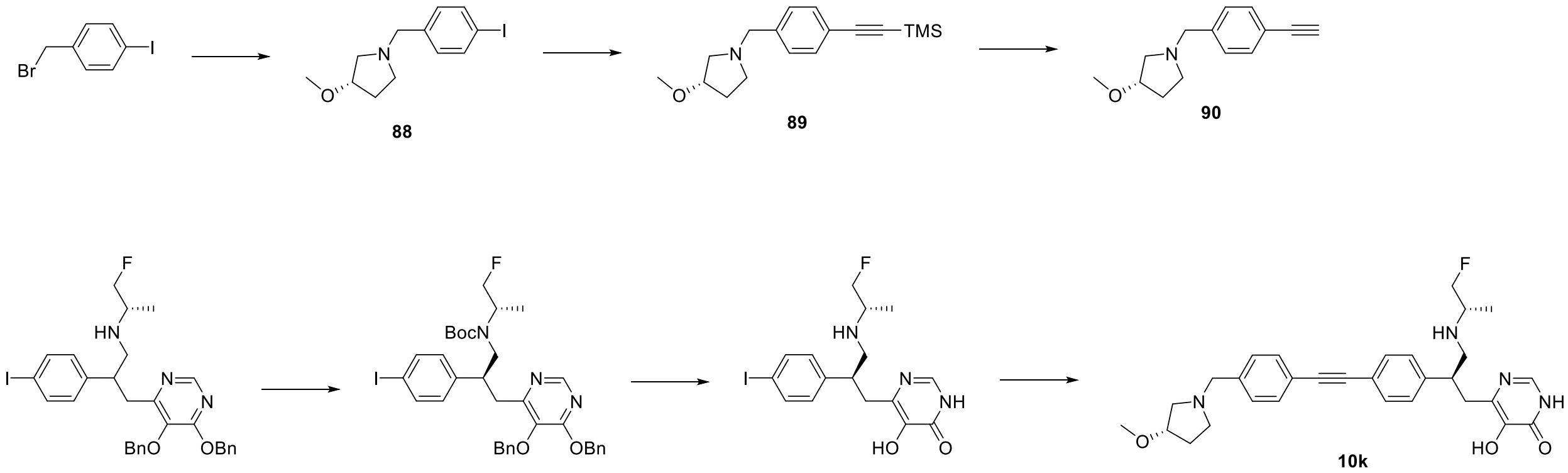

(*S*)-1-(4-iodobenzyl)-3-methoxypyrrolidine (88):

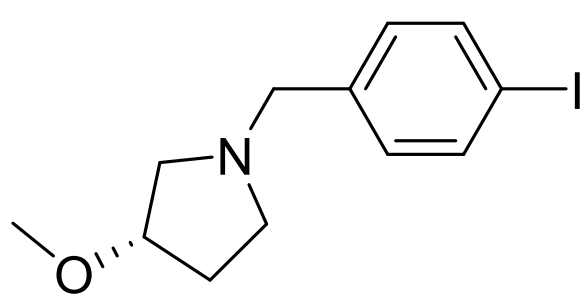

88

Compound **88** was generated from (*S*)-3-methoxypyrrolidine hydrochloride and 1-(bromomethyl)-4-iodobenzene and utilizing General Procedure J (1.8 g, 85%). LC-MS (m/z): 318.3 $[M+H]^+$.

(*S*)-3-methoxy-1-{4-[(trimethylsilyl)ethynyl]benzyl}pyrrolidine (89):

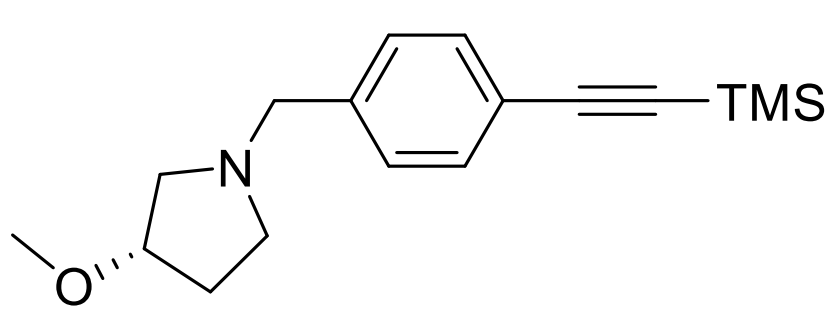

89

Compound **89** was obtained from **88** and ethynyltrimethylsilane utilizing General Procedure B (1.2 g, 74%). LC-MS: product did not ionize.

(*S*)-1-(4-ethynylbenzyl)-3-methoxypyrrolidine (90):

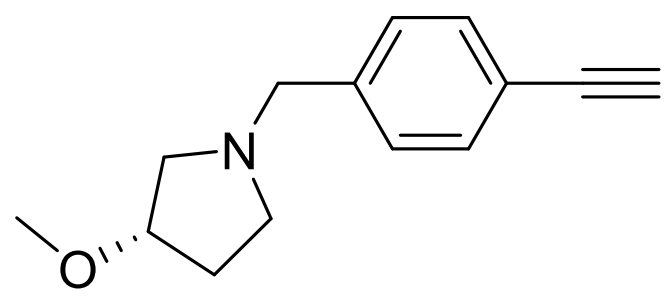

90

Compound **90** was obtained from **89** utilizing General Procedure I (0.26 g, 73%). LC-MS (m/z): 216.2 $[M+H]^+$.

tert-butyl-{(*S*)-3-[5,6-bis(benzyloxy)pyrimidin-4-yl]-2-[4-iodophenyl]propyl}((*S*)-1-fluoropropan-2-yl)carbamate (91):

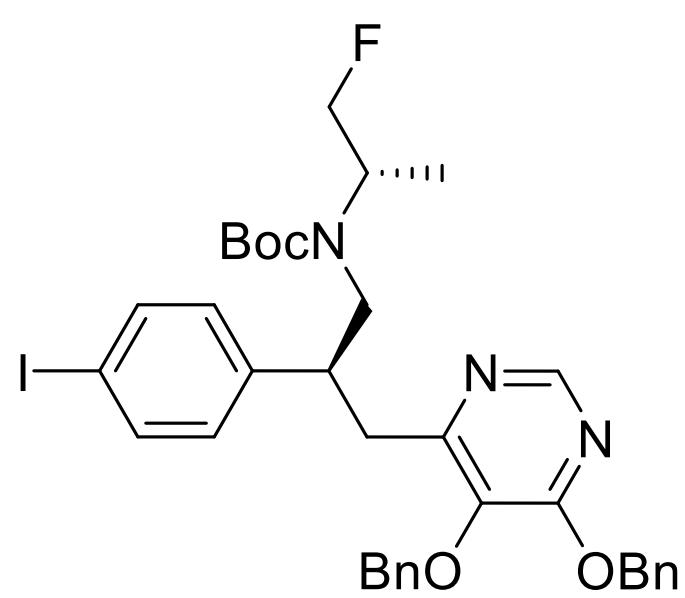

91

Compound **91** was prepared from **40k** utilizing General Procedure G (42 g racemate, 58%) followed by SFC separation. LC-MS (m/z): 712.2 $[M+H]^+$.

6-{(*S*)-3-[((*S*)-1-fluoropropan-2-yl)amino]-2-[4-iodophenyl]propyl}-5-hydroxypyrimidin-4(3H)-one (92):

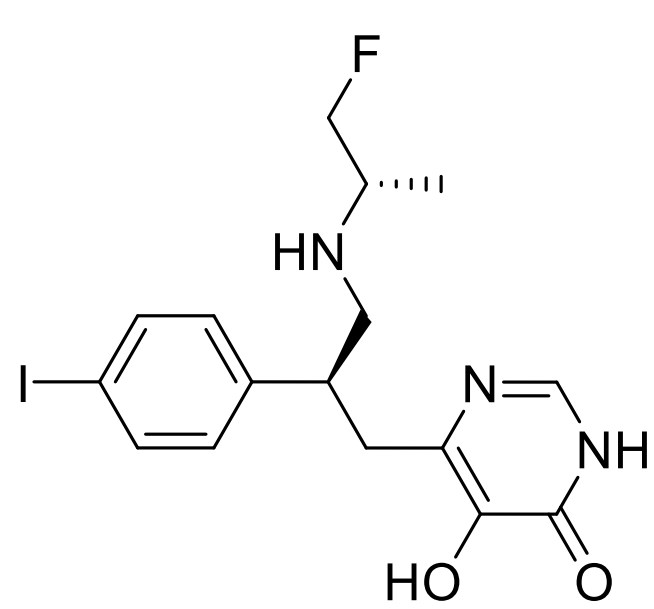

92

Compound **92** was prepared from **91** utilizing General Procedure H (65 g racemate, 75%) followed by SFC separation. LC-MS (m/z): 432.1 $[M+H]^+$.

6-{(S)-3-[((S)-1-fluoropropan-2-yl)amino]-2-[4-({4-[((S)-3-methoxypyrrolidin-1-yl)methyl]-phenyl}ethynyl)phenyl]propyl}-5-hydroxypyrimidin-4(3H)-one (10k):

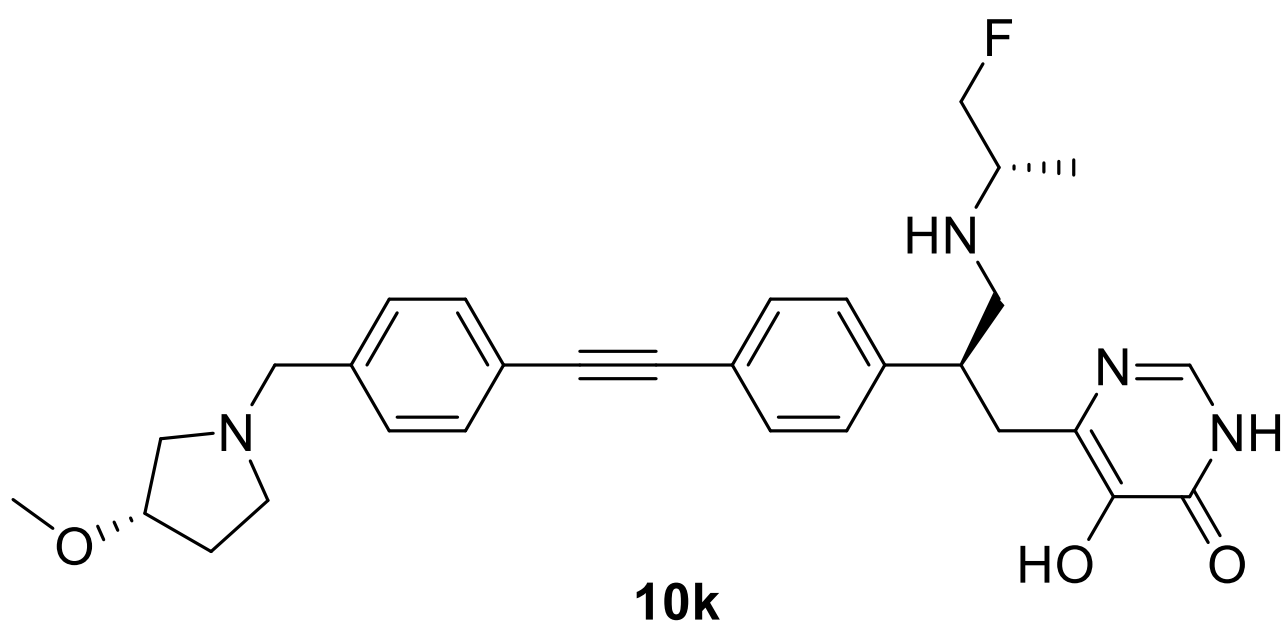

10k

Compound **10k** was obtained from **90** and **92** utilizing General Procedure B with DMF as the solvent (55 mg, 23%). LC-MS (m/z): 519.2 $[M+H]^+$. ^{1}H-NMR (DMSO-d_6): δ 7.66 (br s, 1H), 7.48 (d, J = 8.00 Hz, 2H), 7.41 (d, J = 8.00 Hz, 2H), 7.34 (d, J = 8.00 Hz, 2H), 7.24 (d, J = 8.00 Hz, 2H), 4.26-4.24 (m, 1H), 4.14-4.12 (m, 1H), 3.90-3.86 (m, 1H), 3.58 (d, J = 2.00 Hz, 2H), 3.30-3.27 (m, 2H), 3.15 (s, 1H), 2.91-2.78 (m, 5H), 2.68-2.64 (m, 1H), 2.50-2.39 (m, 2H), 2.01-1.97 (m, 1H), 1.67-1.65 (m, 1H), 0.92 (d, J = 6.00 Hz, 3H).

Procedures for the preparation of Compound 11k

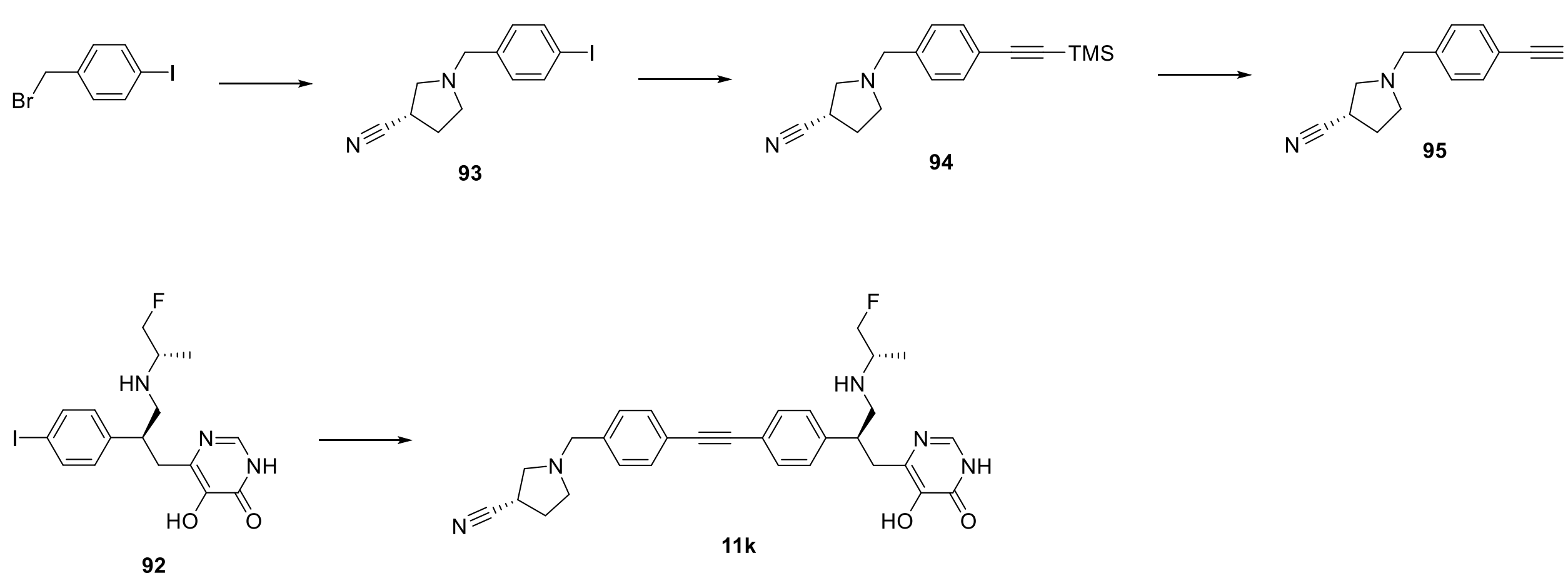

(*S*)-1-(4-iodobenzyl)pyrrolidine-3-carbonitrile (93):

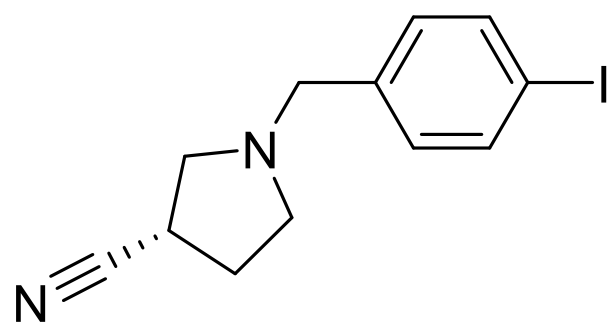

93

Compound **93** was generated from (*S*)-3-pyrrolidine-3-carbonitrile hydrochloride and 1-(bromomethyl)-4-iodobenzene and utilizing General Procedure J (730 mg, 70%). LC-MS (m/z): 313.0 $[M+H]^+$.

(*S*)-1-{4-[(trimethylsilyl)ethynyl]benzyl}pyrrolidine-3-carbonitrile (94):

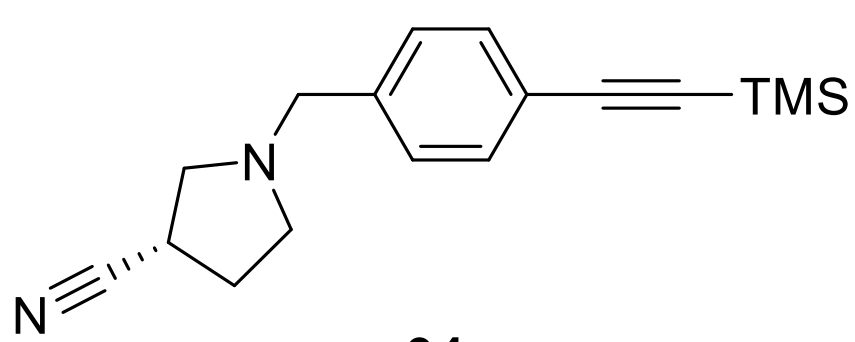

94

Compound **94** was obtained from **93** and ethynyltrimethylsilane utilizing General Procedure B (280 mg, 78%). LC-MS (m/z): 283.1 $[M+H]^+$.

(*S*)-1-(4-ethynylbenzyl)pyrrolidine-3-carbonitrile (95):

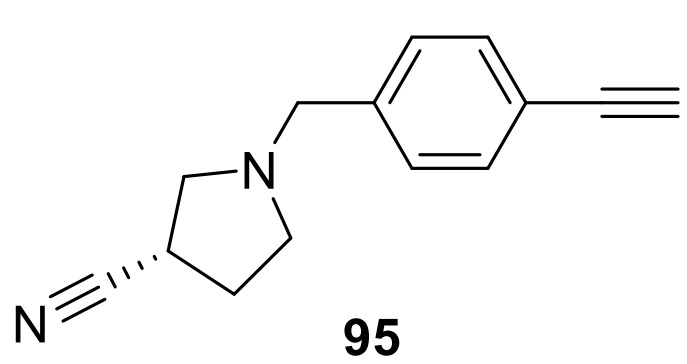

95

Compound **95** was obtained from **94** utilizing General Procedure I (0.14 g, crude). LC-MS (m/z): 211.1 $[M+H]^+$.

(*S*)-1-{4-[(4-{(*S*)-1-[((*S*)-1-fluoropropan-2-yl)amino]-3-[5-hydroxy-6-oxo-1,6-dihydropyrimidin-4-yl]propan-2-yl}phenyl)ethynyl]benzyl}pyrrolidine-3-carbonitrile (11k):

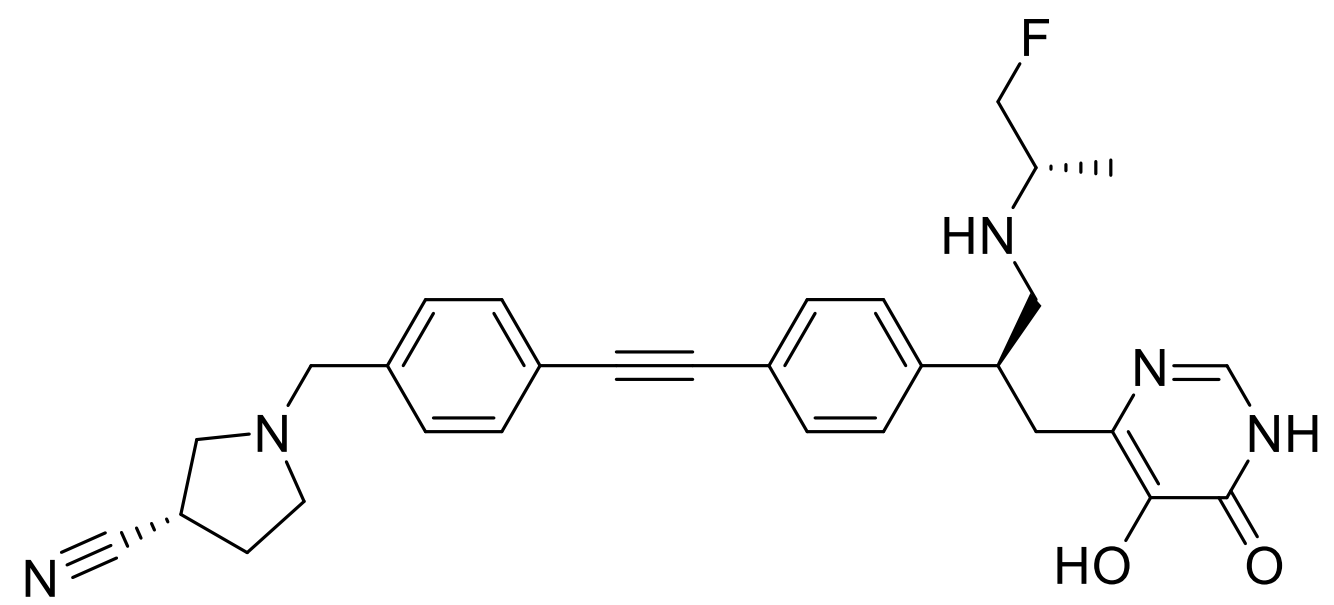

11k

Compound **11k** was obtained from **92** and **95** utilizing General Procedure B with DMF as the solvent (35 mg, 7% over two steps). LC-MS (m/z): 514.3 $[M+H]^+$. ^{1}H-NMR (DMSO-d_6): δ 12.33 (s, 1H), 9.13 (s, 1H), 8.13 (s, 1H), 7.69 (s, 1H), 7.50-7.45 (m, 4H), 7.37-7.30 (m, 6H), 4.65-4.41 (m, 2H), 3.65-3.55 (m, 5H), 2.96-2.91 (m, 3H), 2.89-2.70 (m, 3H), 2.20-2.16 (m, 2H), 1.97-1.91 (m, 2H), 1.10 (d, J = 7.00 Hz, 3H)

Procedures for the preparation of Compound 11t

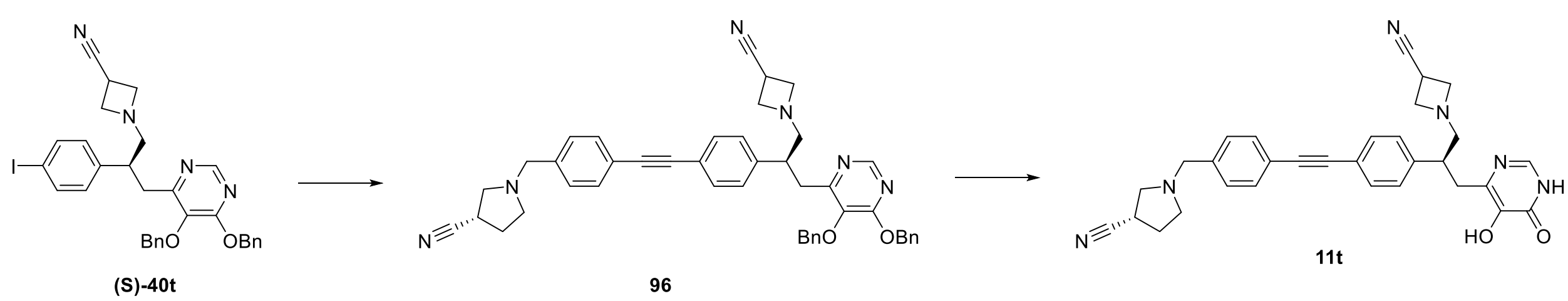

(*S*)-1-{4-[(4-{(S)-1-[5,6-bis(benzyloxy)pyrimidin-4-yl]-3-[3-cyanoazetidin-1-yl]propan-2-yl}phenyl)ethynyl]benzyl}pyrrolidine-3-carbonitrile (96):

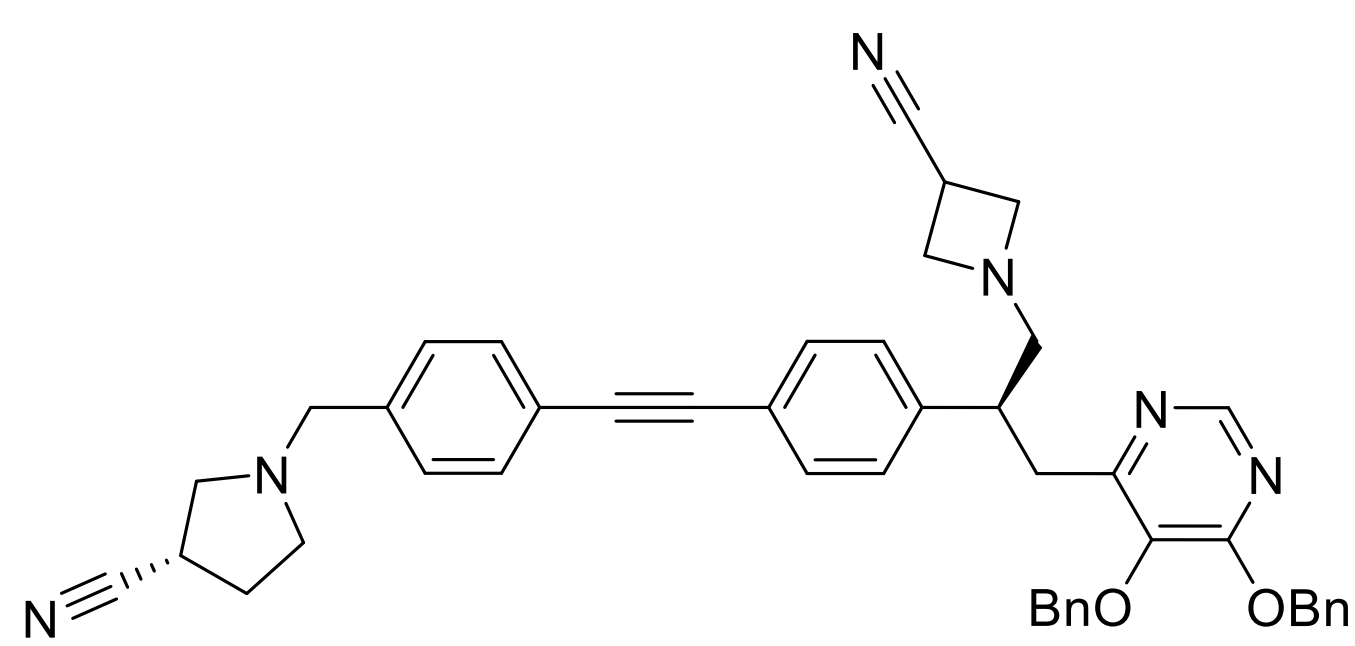

96

Compound **96** was obtained from **(*S*)-40t** and **95** utilizing General Procedure B (0.08 g, crude). LC-MS (m/z): 699.2 $[M+H]^+$.

(*S*)-1-[4-({4-[(*S*)-1-(3-cyanoazetidin-1-yl)-3-(5-hydroxy-6-oxo-1,6-dihydropyrimidin-4-yl)propan-2-yl]phenyl}ethynyl)benzyl]pyrrolidine-3-carbonitrile (11t):

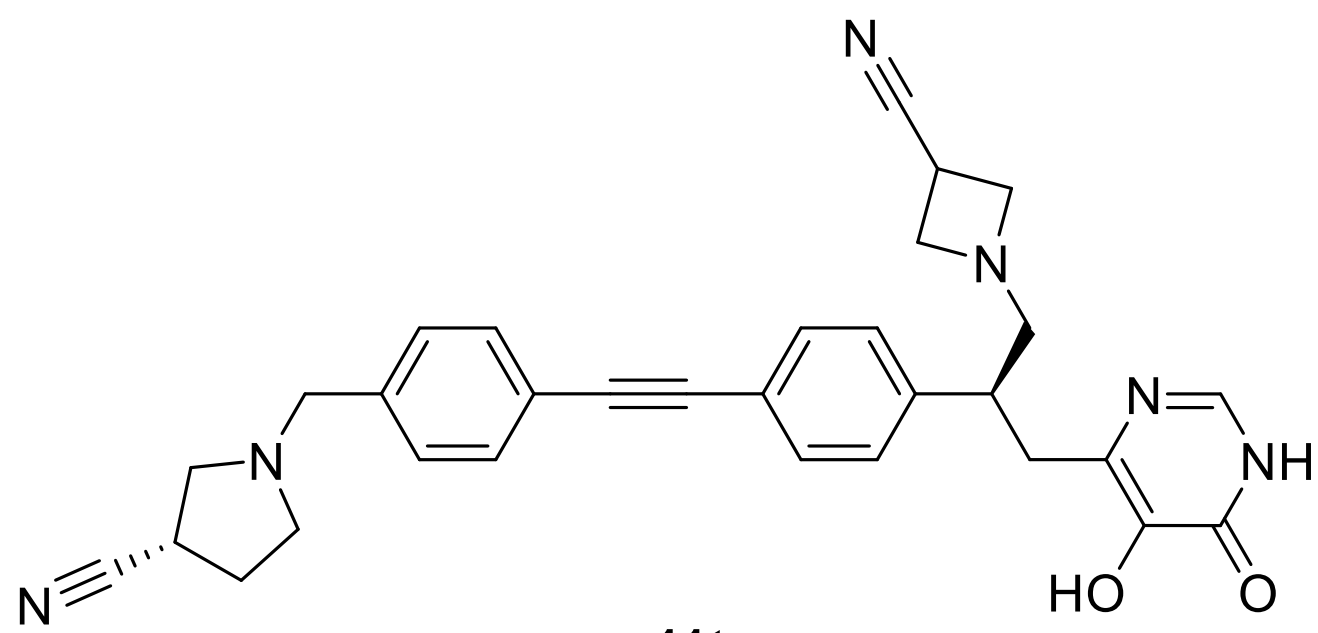

11t

Compound **11t** was obtained from **96** utilizing General Procedure C (5 mg, 8% yield over two steps). LC-MS (m/z): 519.2 $[M+H]^+$. ^{1}H-NMR (DMSO-d_6): δ 8.26 (s, 1H), 7.63 (s, 1H), 7.48 (d, J = 2.3 Hz, 2H), 7.46-7.33 (m, 4H), 7.20 (d, J = 5.7 Hz, 2H), 3.70-3.55 (m, 3H), 3.35-3.25 (m, 2H), 3.35-3.15 (m, 2H), 3.10-3.00 (m, 2H), 1.90-1.70 (m, 2H), 1.70-1.60 (m, 5H), 2.25-2.10 (m, 2H), 1.95-1.85 (m, 1H).

Procedures for the preparation of Compound 12k

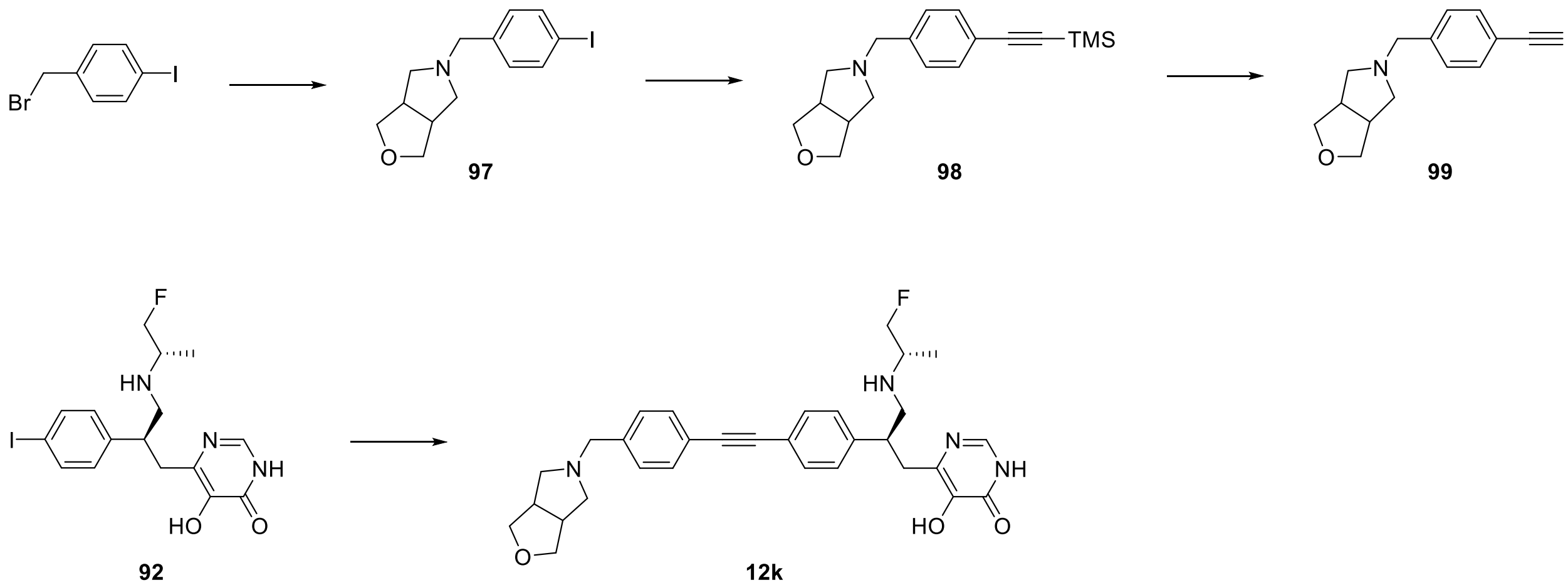

5-(4-iodobenzyl)hexahydro-1H-furo[3,4-c]pyrrole (97):

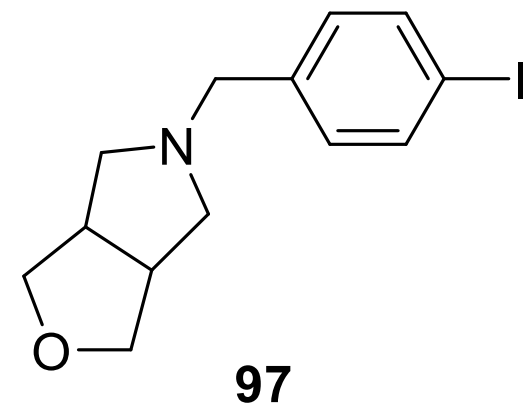

97

Compound **97** was generated from hexahydro-1H-furo[3,4-c]pyrrole hydrochloride and 1-(bromomethyl)-4-iodobenzene and utilizing General Procedure J (400 mg, 72%). LC-MS (m/z): 330.0 $[M+H]^+$.

5-{4-[(trimethylsilyl)ethynyl]benzyl}hexahydro-1H-furo[3,4-c]pyrrole (98):

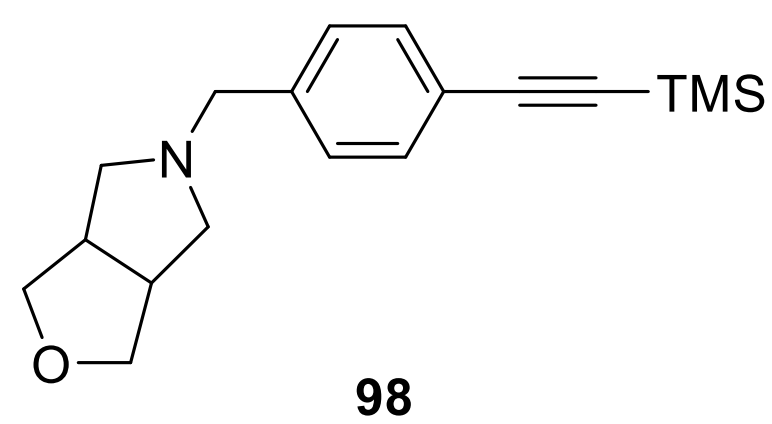

98

Compound **98** was obtained from **97** and ethynyltrimethylsilane utilizing General Procedure B (280 mg, 78%). LC-MS (m/z): 300.2 $[M+H]^+$.

5-(4-ethynylbenzyl)hexahydro-1H-furo[3,4-c]pyrrole (99):

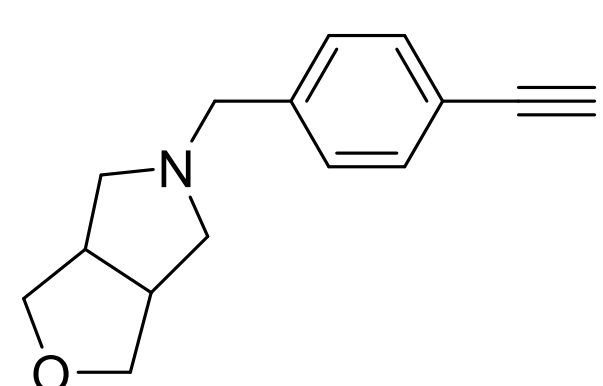

99

Compound **99** was obtained from **98** utilizing General Procedure I (0.20 g, crude). LC-MS (m/z): 228.1 $[M+H]^+$.

(*S*)-1-{4-[(4-{(*S*)-1-[((*S*)-1-fluoropropan-2-yl)amino]-3-[5-hydroxy-6-oxo-1,6-dihydropyrimidin-4-yl]propan-2-yl}phenyl)ethynyl]benzyl}pyrrolidine-3-carbonitrile (12k):

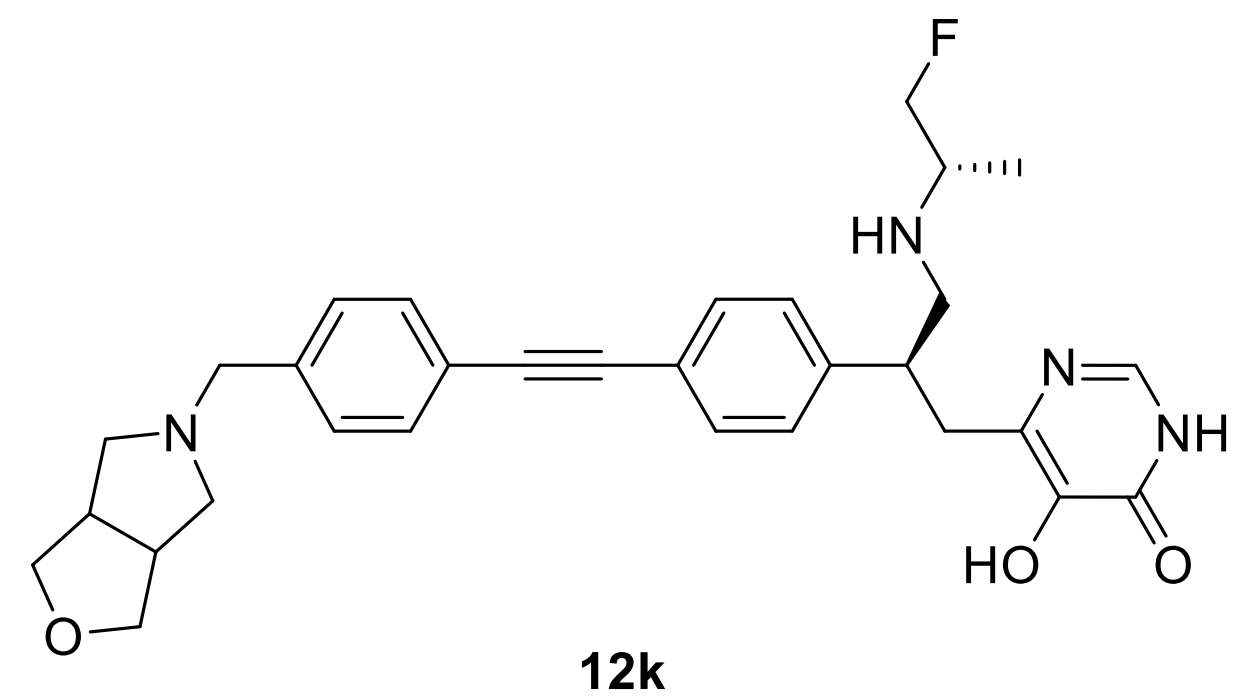

12k

Compound **12k** was obtained from **92** and **99** utilizing General Procedure B with DMF as the solvent (24 mg, 10% over two steps). LC-MS (m/z): 531.3 $[M+H]^+$. ^{1}H-NMR (DMSO-d_6): δ 8.16 (s, 1H), 7.67 (s, 1H), 7.48 (d, J = 7.72 Hz, 2H), 7.42 (d, J = 7.56 Hz, 2H), 7.33 (d, J = 7.64 Hz, 2H), 7.26 (d, J = 7.44 Hz, 2H), 4.33-4.21 (m, 2H), 3.71 (s, 2H), 3.66-3.62 (m, 3H), 3.33-3.23 (m, 6H), 2.90-2.70 (m, 5H), 2.02 (t, J = 6.92 Hz, 2H), 0.98 (d, J = 5.24 Hz, 3H).

Procedures for the preparation of Compound 13b

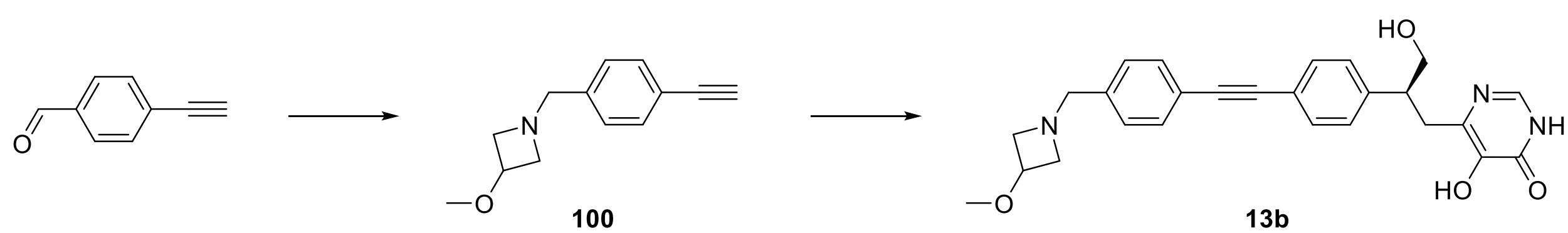

1-(4-ethynylbenzyl)-3-methoxyazetidine (100):

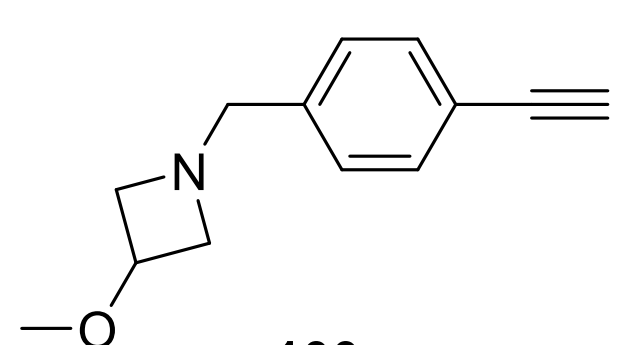

100

Compound **100** was synthesized from 4-ethynylbenzaldehyde and 3-methoxyazetidine hydrochloride utilizing General Procedure A (180 mg, 12%). LC-MS (m/z): 202.1 $[M+H]^+$.

(*S*)-5-hydroxy-6-{3-hydroxy-2-[4-({4-[(3-methoxyazetidin-1-yl)methyl]phenyl}ethynyl)-phenyl]propyl}pyrimidin-4(3H)-one (13b):

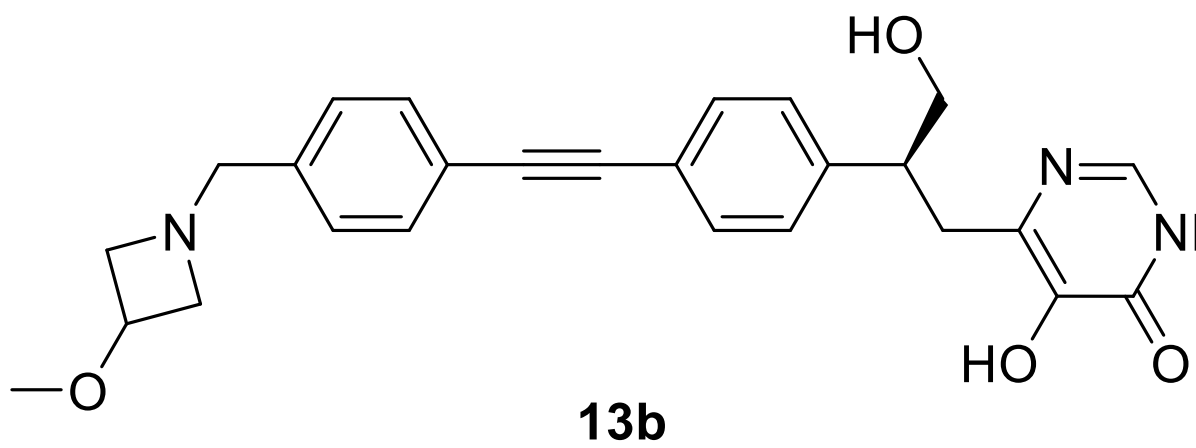

13b

Compound **13b** was synthesized from **(*S*)-37** and **100** utilizing General Procedure B with DMF as the solvent (9 mg, ~5%). LC-MS (m/z): 446.1 $[M+H]^+$. ^{1}H-NMR (DMSO-d_6): δ 12.37 (br s, 1H), 9.02 (br s, 1H), 7.64 (br s, 1H), 7.52-7.28 (m, 8H), 4.70 (m, 1H), 3.98-3.94 (m, 1H), 3.58-3.57 (m, 5H), 3.48-3.42 (m, 3H), 3.14 (s, 3H), 2.85-2.83 (m, 5H)

Procedures for the preparation of Compound 13g

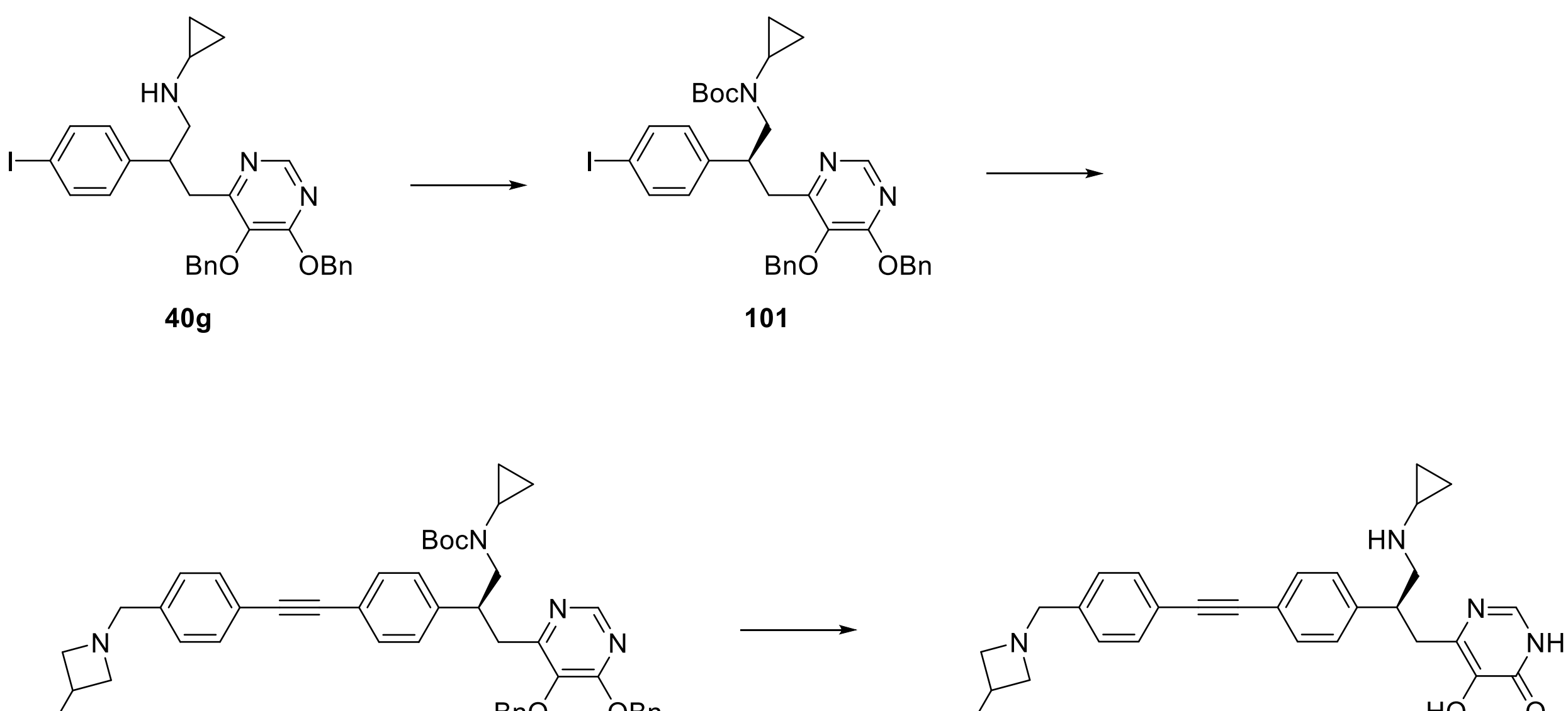

40g **101** **102** **13g**

tert-butyl-(S)-{3-[5,6-bis(benzyloxy)pyrimidin-4-yl]-2-[4-iodophenyl]propyl}cyclopropyl-carbamate (101):

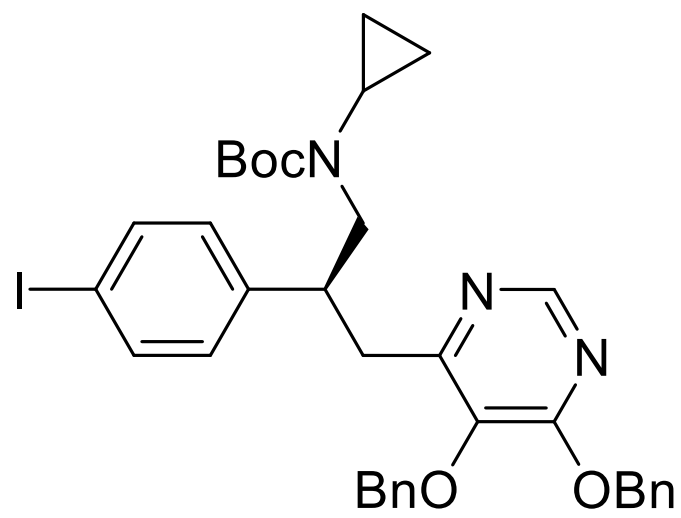

101

Compound **101** was prepared from **40g** utilizing General Procedure G (1.1 g racemate, 67%) followed by SFC separation. LC-MS (m/z): 690.0 [M+H]$^+$.

***tert*-butyl-(*S*)-{3-[5,6-bis(benzyloxy)pyrimidin-4-yl]-2-[4-({4-[(3-methoxyazetidin-1-yl)methyl]phenyl}ethynyl)phenyl]propyl}(cyclopropyl)carbamate (102):**

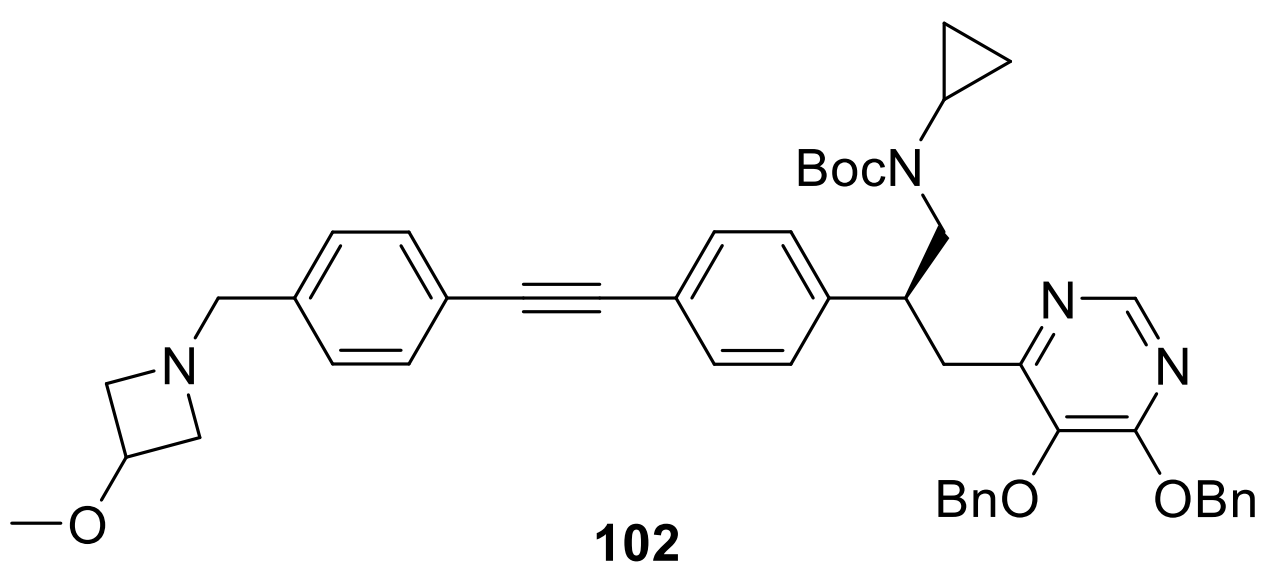

102

Compound **102** was prepared from **100** and **101** utilizing General Procedure B (0.40 g, 69%). LC-MS (m/z): 765.3 [M+H]$^+$.

(*S*)-6-{3-[cyclopropylamino]-2-[4-({4-[(3-methoxyazetidin-1-yl)methyl]phenyl}ethynyl)-phenyl]propyl}-5-hydroxypyrimidin-4(3H)-one (13g):

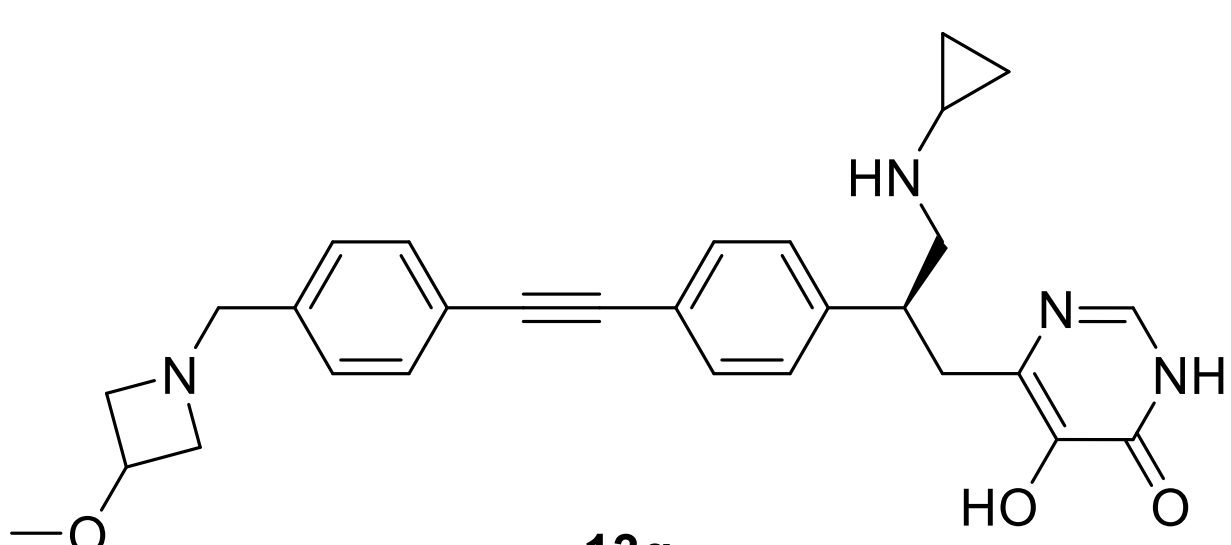

13g

Compound **102** was converted to **13g** utilizing General Procedure D (110 mg, 41%). LC-MS (m/z): 485.3 $[M+H]^+$. ^{1}H-NMR (DMSO-d_6): δ 8.20 (s, 1H), 7.66 (s, 1H), 7.46 (d, J = 8.0 Hz, 2H), 7.40 (d, J = 8.1 Hz, 2H), 7.30 (d, J = 8.0 Hz, 2H), 7.22 (d, J = 8.1 Hz, 2H), 3.98 (t, J = 5.7 Hz, 1H), 3.65-3.55 (m, 3H), 3.50-3.40 (m, 2H), 3.15 (s, 3H), 2.90-2.75 (m, 6H), 2.05-2.02 (m, 1H), 0.32 (t, J = 4.7 Hz, 3H), 0.16 (t, J = 3.2 Hz, 3H).

Procedures for the preparation of Compound 13h

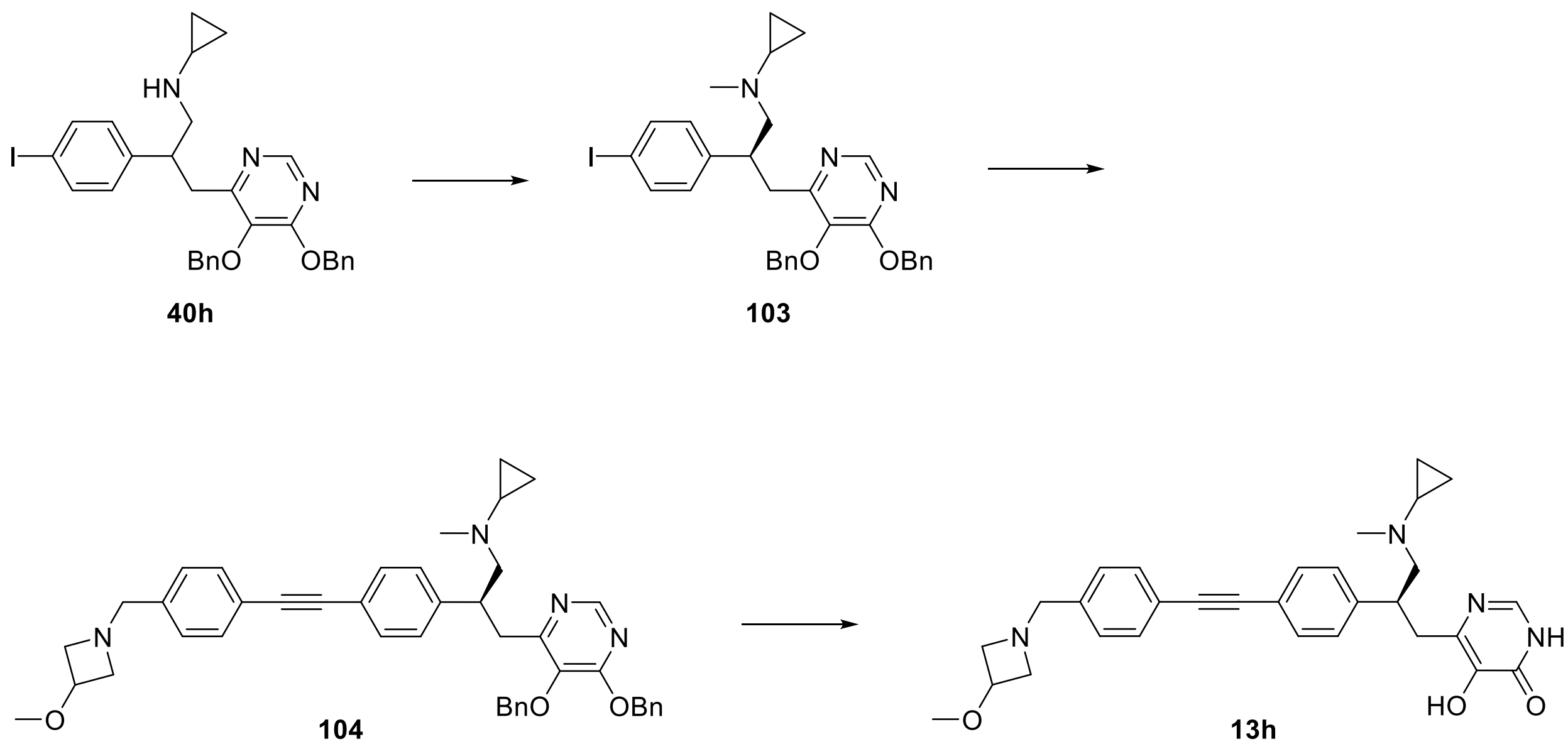

(*S*)-*N*-{3-[5,6-bis(benzyloxy)pyrimidin-4-yl]-2-[4-iodophenyl]propyl}-*N*-methylcyclopropanamine (103):

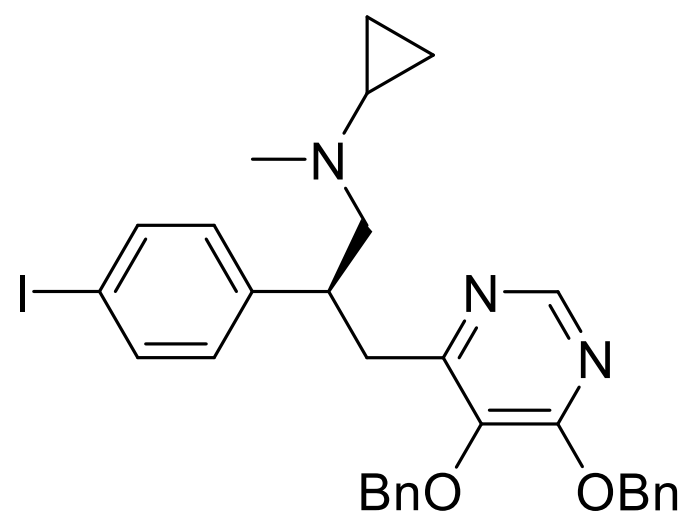

103

Compound **103** was prepared from **40h** utilizing General Procedure E (1.0 g racemate, 57%) followed by SFC separation. LC-MS (m/z): 592.1 $[M-CH_3]^+$.

(*S*)-N-{3-[5,6-bis(benzyloxy)pyrimidin-4-yl]-2-[4-({4-[(3-methoxyazetidin-1-yl)methyl]-phenyl}ethynyl)phenyl]propyl}-N-methylcyclopropanamine (104):

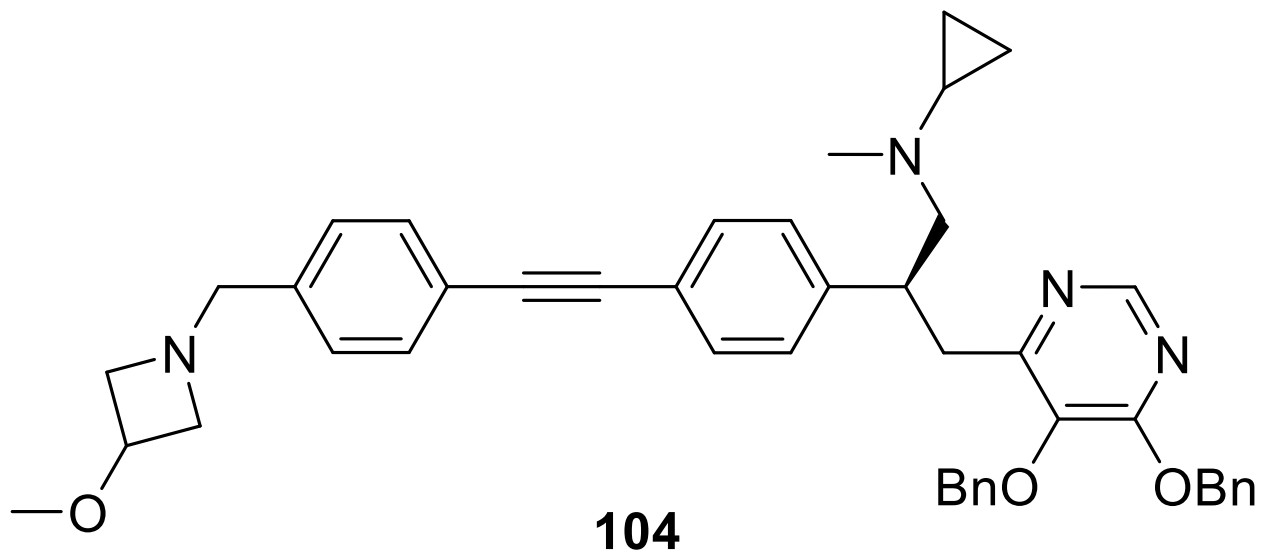

104

Compound **104** was prepared from **100** and **103** utilizing General Procedure B (0.40 g, 52%). LC-MS (m/z): 779.3 $[M+H]^+$.

(*S*)-6-{3-[cyclopropyl(methyl)amino]-2-[4-({4-[(3-methoxyazetidin-1-yl)methyl]phenyl}-ethynyl)phenyl]propyl}-5-hydroxypyrimidin-4(3H)-one (13h):

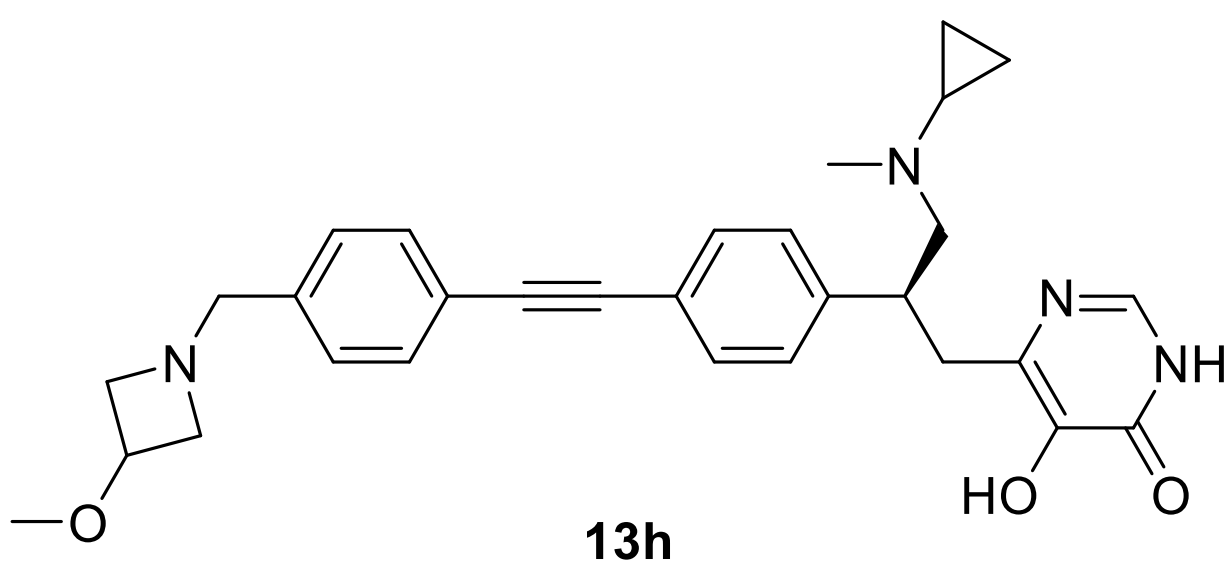

13h

Compound **104** was converted to **13h** utilizing General Procedure D (65 mg, 36%). LC-MS (m/z): 497.2 $[M-H]^+$. ^{1}H-NMR (DMSO-d_6): δ 12.19 (s, 1H), 8.81 (s, 1H), 7.49 (s, 1H), 7.30 (d, J = 8.12 Hz, 2H), 7.21 (d, J = 8.20 Hz, 2H), 7.14 (d, J = 8.20 Hz, 2H), 7.03 (d, J = 8.24 Hz, 2H), 3.83-3.80 (m, 1H), 3.43 (s, 2H), 3.33-3.29 (m, 4H), 2.98 (s, 3H), 2.70-2.67 (m, 3H), 2.65-2.57 (m, 3H), 2.07 (s, 3H), 1.46-1.45 (m, 1H), 0.21-0.19 (m, 2H), 0.02-0.01 (m, 2H).

Procedure for the preparation of Compound 13k

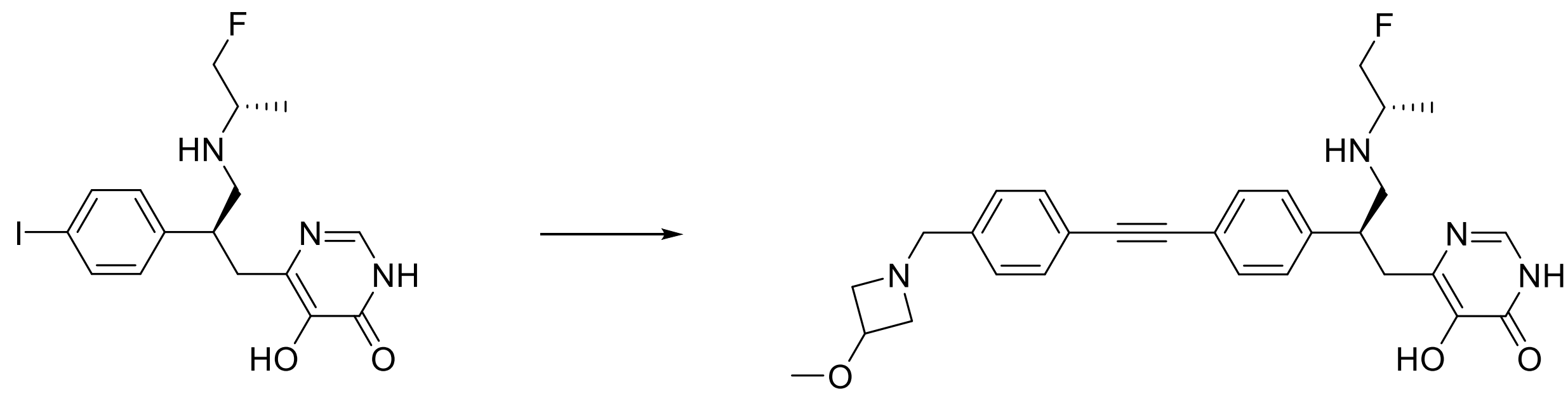

92 **13k**

6-{3-[((*S*)-1-fluoropropan-2-yl)amino]-2-[4-({4-[(3-methoxyazetidin-1-yl)methyl]phenyl}-ethynyl)phenyl]propyl}-5-hydroxypyrimidin-4(3H)-one (13k):

Compound **13k** was synthesized from **92** and **100** utilizing General Procedure B with DMF as the solvent (31 mg, 13%). LC-MS (m/z): 505.3 $[M+H]^+$. ^{1}H-NMR (DMSO-d_6): δ 8.24 (br s, 1H), 7.65 (br s, 1H), 7.46-7.39 (m, 4H), 7.30-7.23 (m, 4H), 4.24 (d, J = 2.00 Hz, 2H), 4.12-3.95 (m, 1H), 3.58-3.45 (m, 3H), 3.39-3.37 (m, 3H), 3.30-3.26 (m, 2H), 3.14 (s, 3H), 2.87-2.50 (m, 7H), 0.91 (d, J = 6.00 Hz, 3H).

Procedure for the preparation of Compound 13p

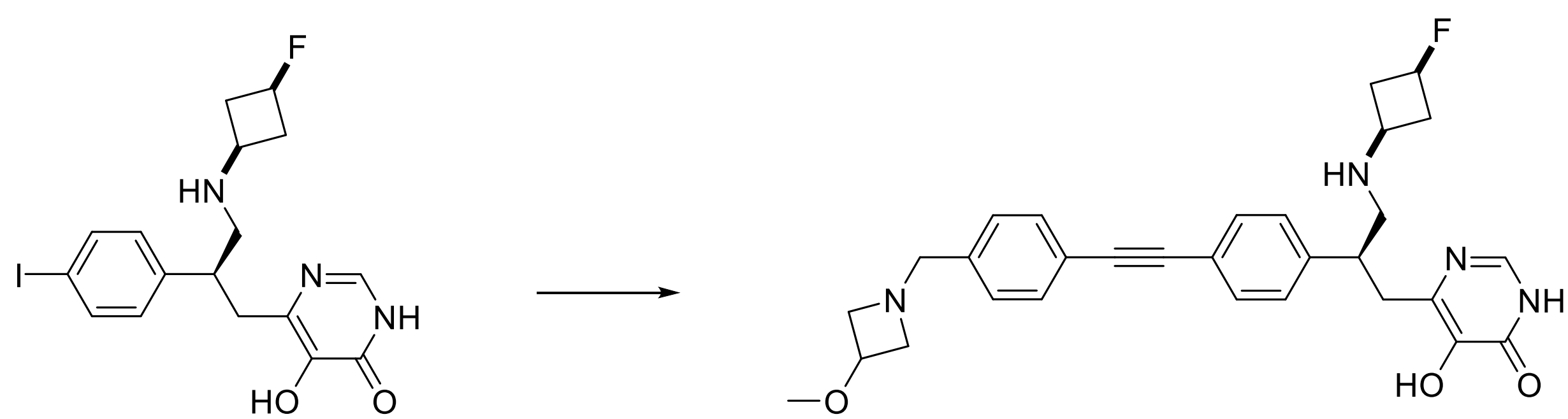

76 **13p**

6-{3-[((S)-1-fluoropropan-2-yl)amino]-2-[4-({4-[(3-methoxyazetidin-1-yl)methyl]phenyl}-ethynyl)phenyl]propyl}-5-hydroxypyrimidin-4(3H)-one (13p):

Compound **13p** was synthesized from **76** and **100** utilizing General Procedure B with DMF as the solvent (5 mg, 15%). LC-MS (m/z): 515.2 $[M-H]^-$. ^{1}H-NMR (DMSO-d_6): δ 12.45 (br s, 1H),

9.15 (br s, 1H), 8.13 (br s, 1H), 7.67 (s, 1H), 7.49-7.44 (m, 6H), 7.35-7.27 (m, 2H), 4.90-4.75 (m, 1H), 4.02 (s, 1H), 3.74-3.62 (m, 2H), 3.46 (m, 2H), 3.21 (m, 2H), 3.16-3.08 (m, 10H), 2.87 (d, J = 6.80 Hz, 2H), 2.67-2.32 (m, 2H), 2.18-2.13 (m, 2H).

Procedure for the preparation of Compound 13s

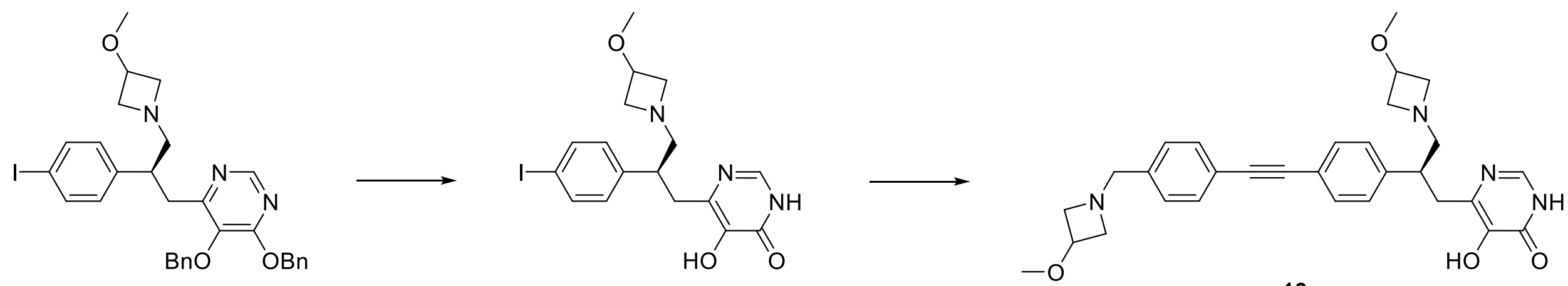

(S)-40s 105 13s

(*S*)-5-hydroxy-6-[2-(4-iodophenyl)-3-(3-methoxyazetidin-1-yl)propyl]pyrimidin-4(3H)-one (105):

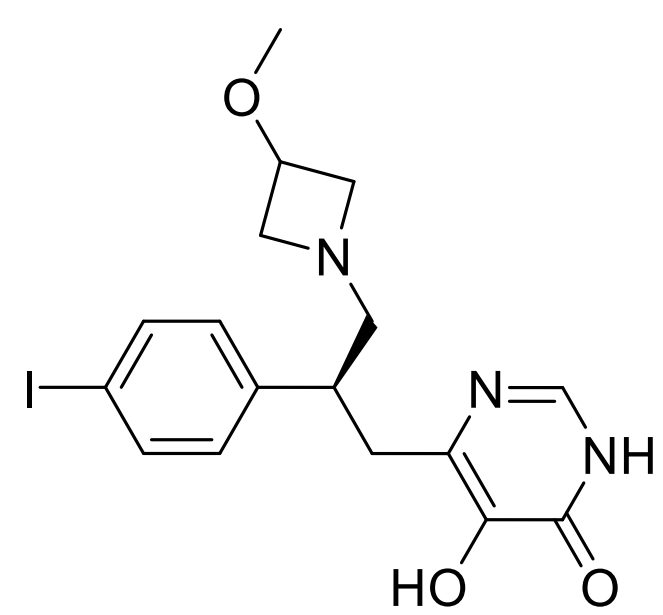

105

Compound **105** was synthesized from **(*S*)-40s** utilizing General Procedure D (0.40 g, 70%). LC-MS (m/z): 442.0 $[M+H]^+$

5-hydroxy-6-{(*S*)-3-[3-methoxyazetidin-1-yl]-2-[4-({4-[(3-methoxyazetidin-1-yl)methyl]phenyl}ethynyl)phenyl]propyl}pyrimidin-4(3H)-one (13s):

Compound **13s** was synthesized from **100** and **105** utilizing General Procedure B (45 mg, 20%). LC-MS (m/z): 515.3 $[M+H]^+$. ^{1}H-NMR (DMSO-*d*$_6$): δ 12.32 (br s, 1H), 7.65 (s, 1H), 7.46 (d, J = 8.0 Hz, 2H), 7.38 (d, J = 8.0 Hz, 2H), 7.30 (d, J = 8.0 Hz, 2H), 7.20 (d, J = 8.0 Hz, 2H), 3.98 (t, J

= 5.2 Hz, 1H), 3.87-3.86 (m, 1H), 3.70-3.50 (m, 2H), 3.50-3.40 (m, 4H), 3.20-3.10 (m, 2H), 3.15 (s, 3H), 3.13 (s, 3H), 2.90-2.80 (m, 4H), 2.70-2.55 (m, 3H).

Procedure for the preparation of Compound 13t

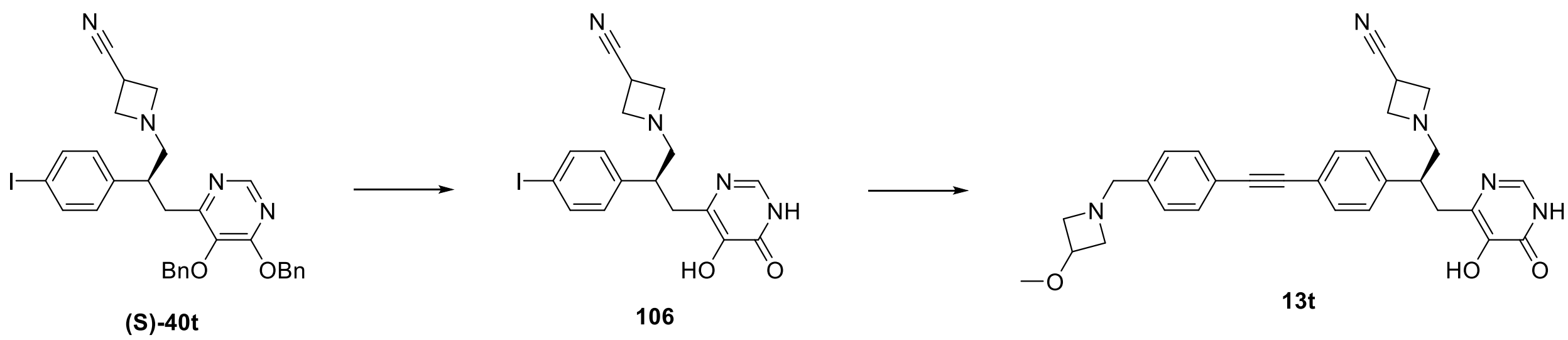

(*S*)-1-[3-(5-hydroxy-6-oxo-1,6-dihydropyrimidin-4-yl)-2-(4-iodophenyl)propyl]azetidine-3-carbonitrile (106):

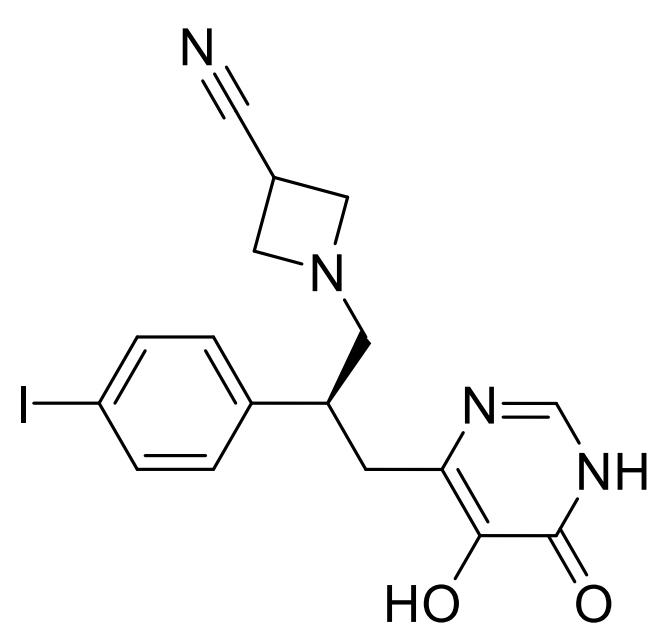

106

Compound **106** was synthesized from **(*S*)-40t** utilizing General Procedure C (0.18 g, 51%). LC-MS (m/z): 437.0 [M+H]$^+$

(*S*)-1-{3-[5-hydroxy-6-oxo-1,6-dihydropyrimidin-4-yl]-2-[4-({4-[(3-methoxyazetidin-1-yl)-methyl]phenyl}ethynyl)phenyl]propyl}azetidine-3-carbonitrile (13t):

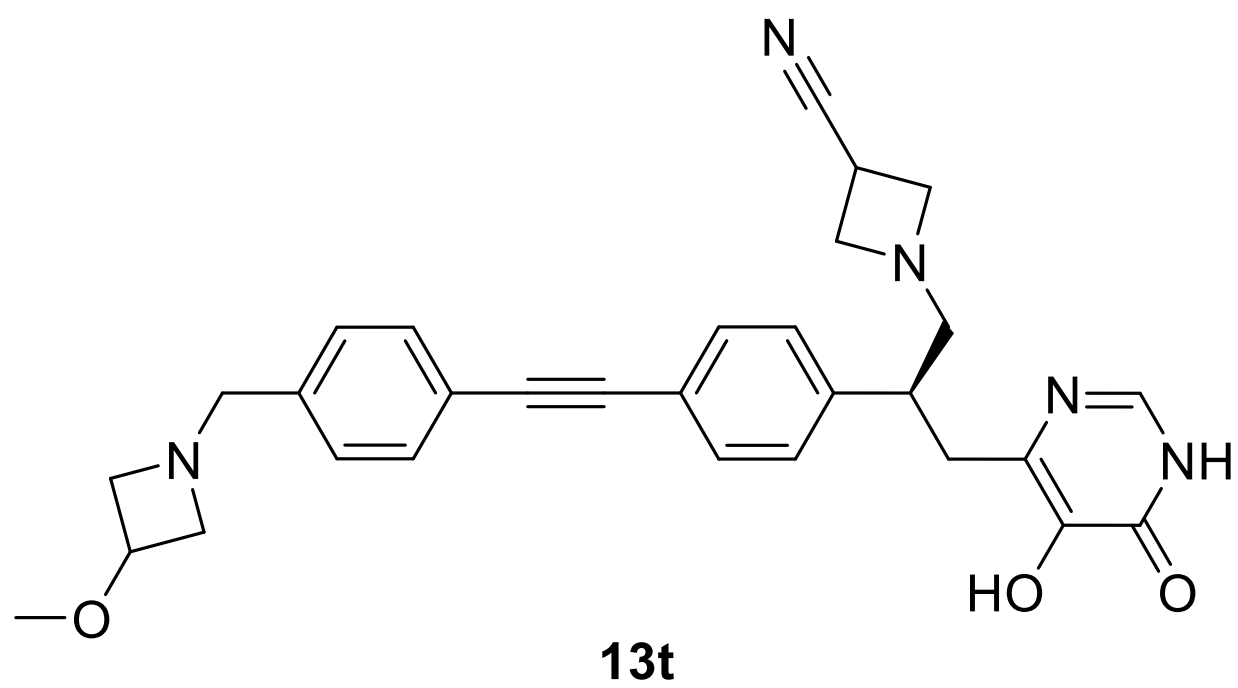

13t

Compound **13t** was synthesized from **100** and **106** utilizing General Procedure B with DMF as the solvent (74 mg, 35%). LC-MS (m/z): 510.3 $[M+H]^+$. ^{1}H-NMR (DMSO-d_6): δ 12.38 (br s, 1H), 9.05 (br s, 1H), 8.15 (s, 1H), 7.66 (s, 1H), 7.49 (d, J = 7.6 Hz, 2H), 7.34 (d, J = 6.8 Hz, 2H), 7.40 (d, J = 8.0 Hz, 2H), 7.23 (d, J = 8.0 Hz, 2H), 4.10-3.90 (m, 2H), 3.80-3.50 (m, 5H), 3.17 (s, 3H), 3.10-2.90 (m, 5H), 2.90-2.70 (m, 2H), 2.70-2.55 (m, 1H), 2.50-2.35 (m, 1H).

Procedure for the preparation of Compound 14k

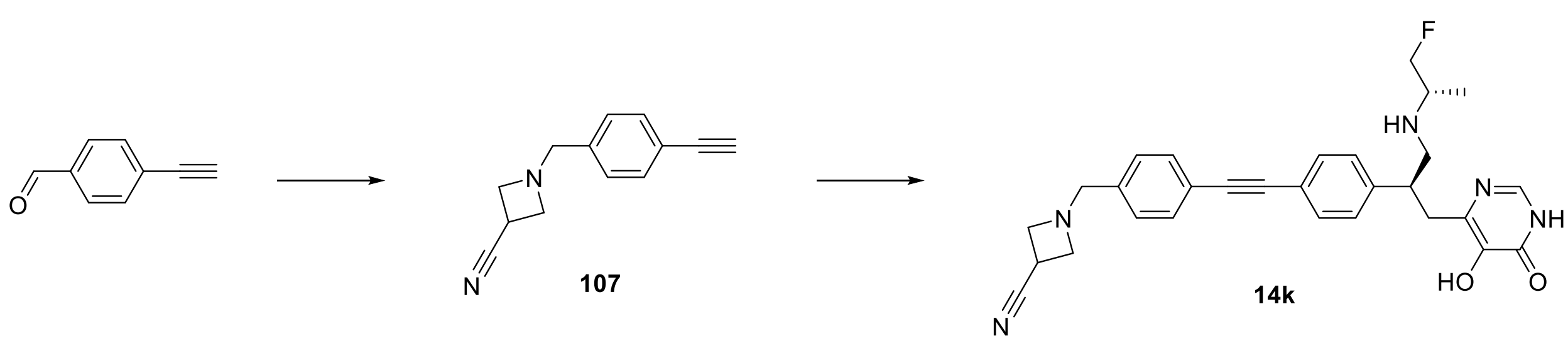

1-(4-ethynylbenzyl)azetidine-3-carbonitrile (107):

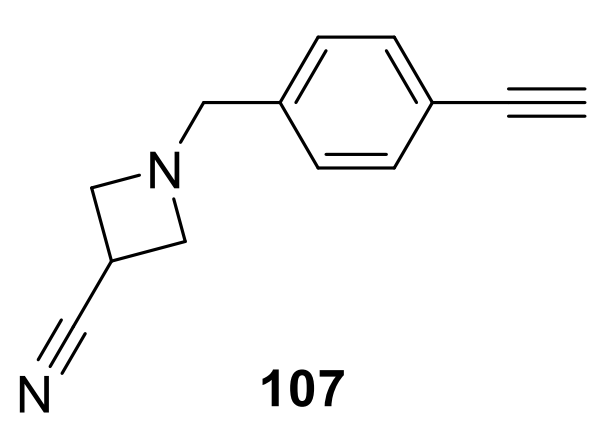

107

Compound **107** was obtained from 4-ethynylbenzaldehyde and azetidine-3-carbonitrile hydrochloride utilizing General Procedure A (0.30 g, 28%). LC-MS (m/z): 197.0 $[M+H]^+$.

1-{4-[(4-{(*S*)-1-[((*S*)-1-fluoropropan-2-yl)amino]-3-[5-hydroxy-6-oxo-1,6-dihydropyrimidin-4-yl]propan-2-yl}phenyl)ethynyl]benzyl}azetidine-3-carbonitrile (14k):

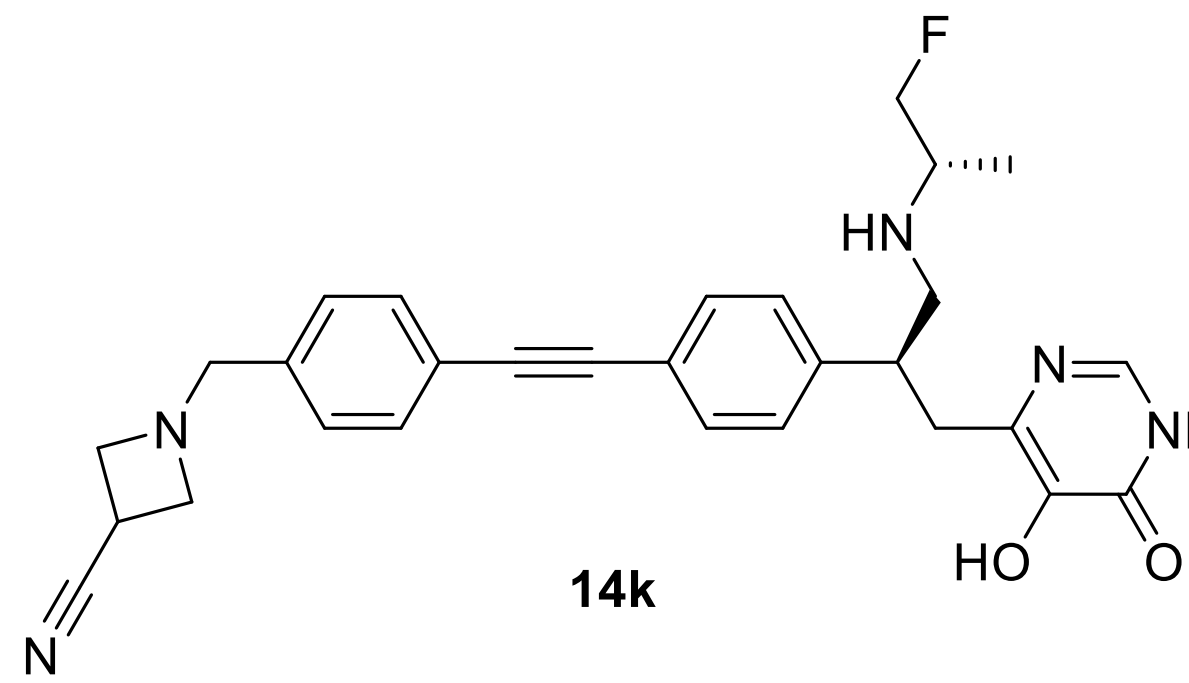

Compound **14k** was obtained from **92** and **107** utilizing General Procedure B with DMF as the solvent (68 mg, 17%). LC-MS (m/z): 500.2 $[M+H]^+$. ^{1}H-NMR (DMSO-d_6): δ 8.15 (br s, 1H), 7.65 (br s, 1H), 7.47 (d, J = 8.00 Hz, 2H), 7.41 (d, J = 8.00 Hz, 2H), 7.29 (d, J = 7.60 Hz, 2H), 7.25 (d, J = 8.00 Hz, 2H), 4.29 (d, J = 4.80 Hz, 1H), 4.17 (d, J = 5.20 Hz, 1H), 3.59 (s, 2H), 3.50-3.42 (m, 3H), 3.34-3.26 (m, 3H), 2.94-2.81 (m, 4H), 0.95 (d, J = 6.40 Hz, 3H)

Procedures for the preparation of Compound 15k:

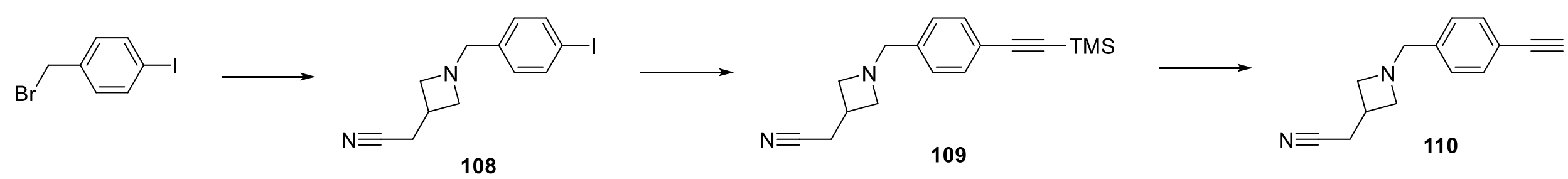

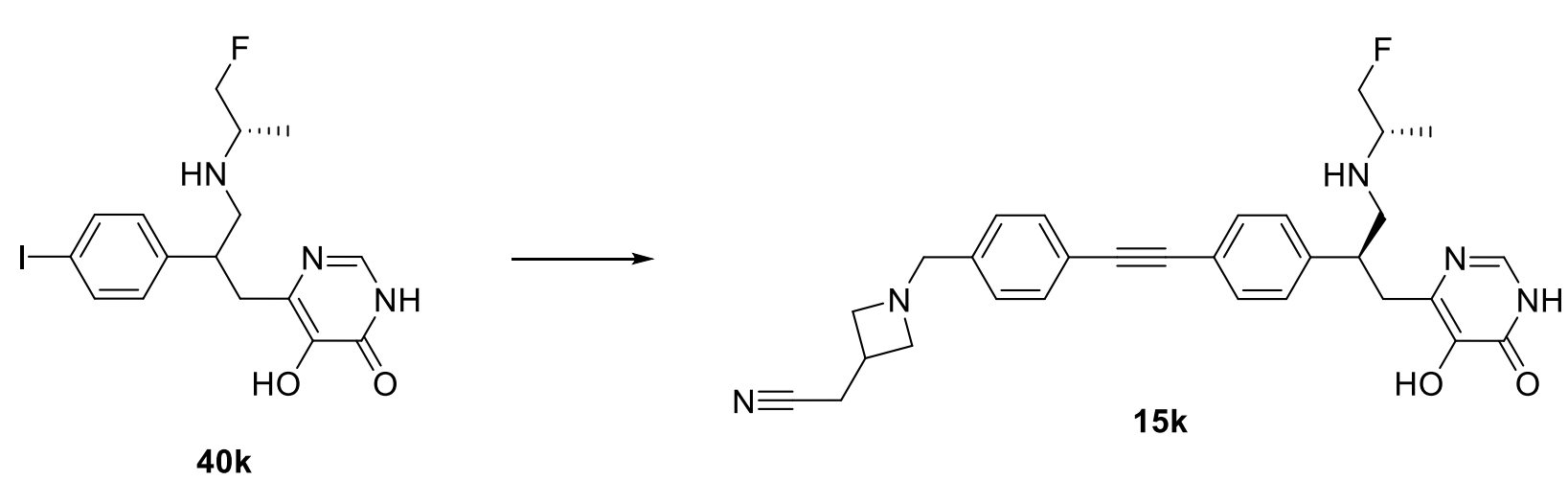

2-[1-(4-iodobenzyl)azetidin-3-yl]acetonitrile (108):

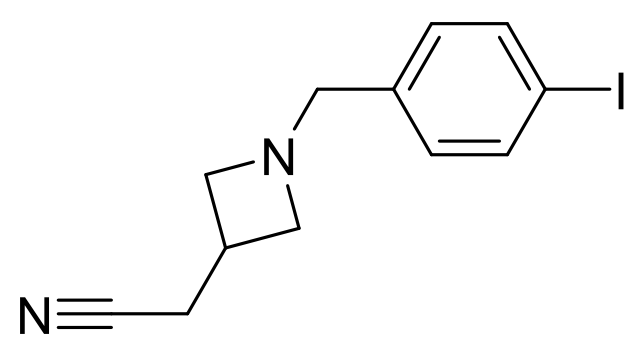

108

Compound **108** was generated from 2-(azetidin-3-yl)acetonitrile hydrochloride and 1-(bromomethyl)-4-iodobenzene and utilizing General Procedure J (300 mg, 71%). LC-MS (m/z): 313.1 $[M+H]^+$.

2-(1-{4-[(trimethylsilyl)ethynyl]benzyl}azetidin-3-yl)acetonitrile (109):

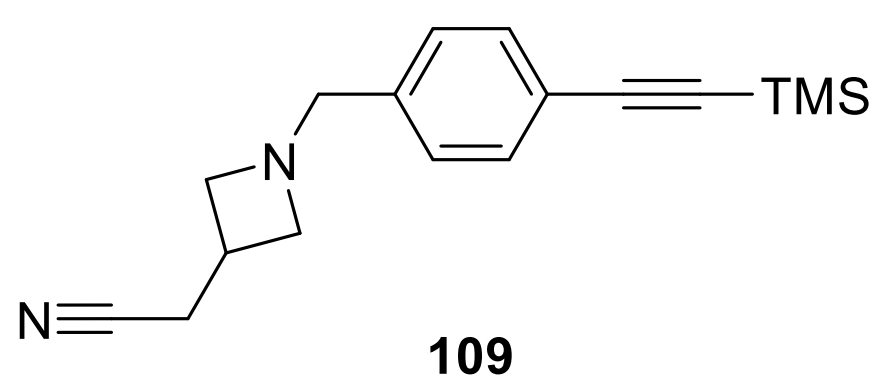

109

Compound **109** was prepared from **108** and ethynyltrimethylsilane utilizing General Procedure B (180 mg, 67%). LC-MS (m/z): 283.2 $[M+H]^+$.

2-[1-(4-ethynylbenzyl)azetidin-3-yl]acetonitrile (110):

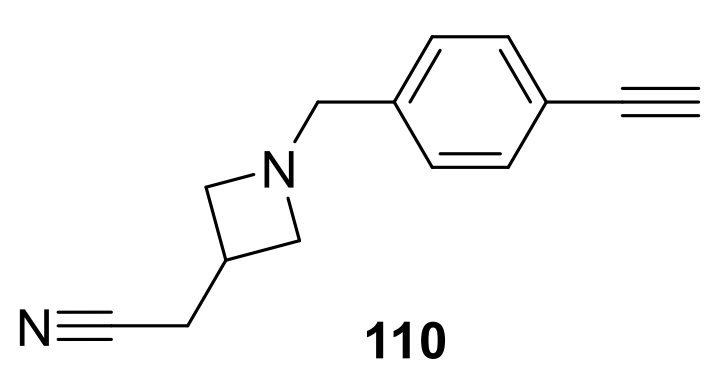

110

Compound **110** was prepared from **109** utilizing General Procedure I (92 mg, crude). LC-MS (m/z): 211.1 $[M+H]^+$.

2-(1-{4-[(4-{1-[((S)-1-fluoropropan-2-yl)amino)]-3-[5-hydroxy-6-oxo-1,6-dihydropyrimidin-4-yl]propan-2-yl}phenyl)ethynyl]benzyl}azetidin-3-yl)acetonitrile (15k):

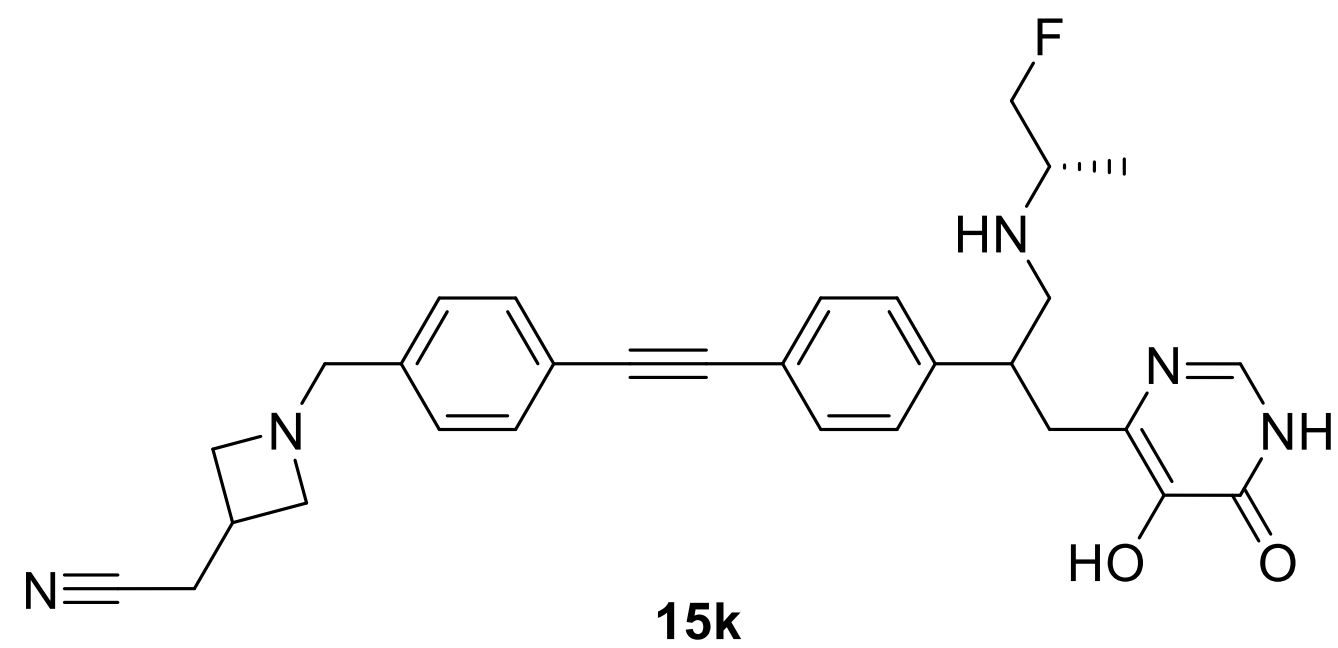

15k

Compound **15k** was obtained from **40k** and **110** utilizing General Procedure B with DMF as the solvent (30 mg, 9% over two steps). LC-MS (m/z): 514.2 $[M+H]^+$. ^{1}H-NMR (DMSO-d_6): δ 8.14 (br s, 1H), 7.69 (br s, 1H), 7.53-7.51 (m, 4H), 7.48-7.46 (m, 4H), 4.52-4.42 (m, 2H), 3.85-3.52 (m, 5H), 3.17-3.11 (m, 4H), 2.97-2.91 (m, 3H), 2.90-2.83 (m, 3H), 1.16 (d, J = 5.12 Hz, 3H).

Procedures for the preparation of Compound 16k

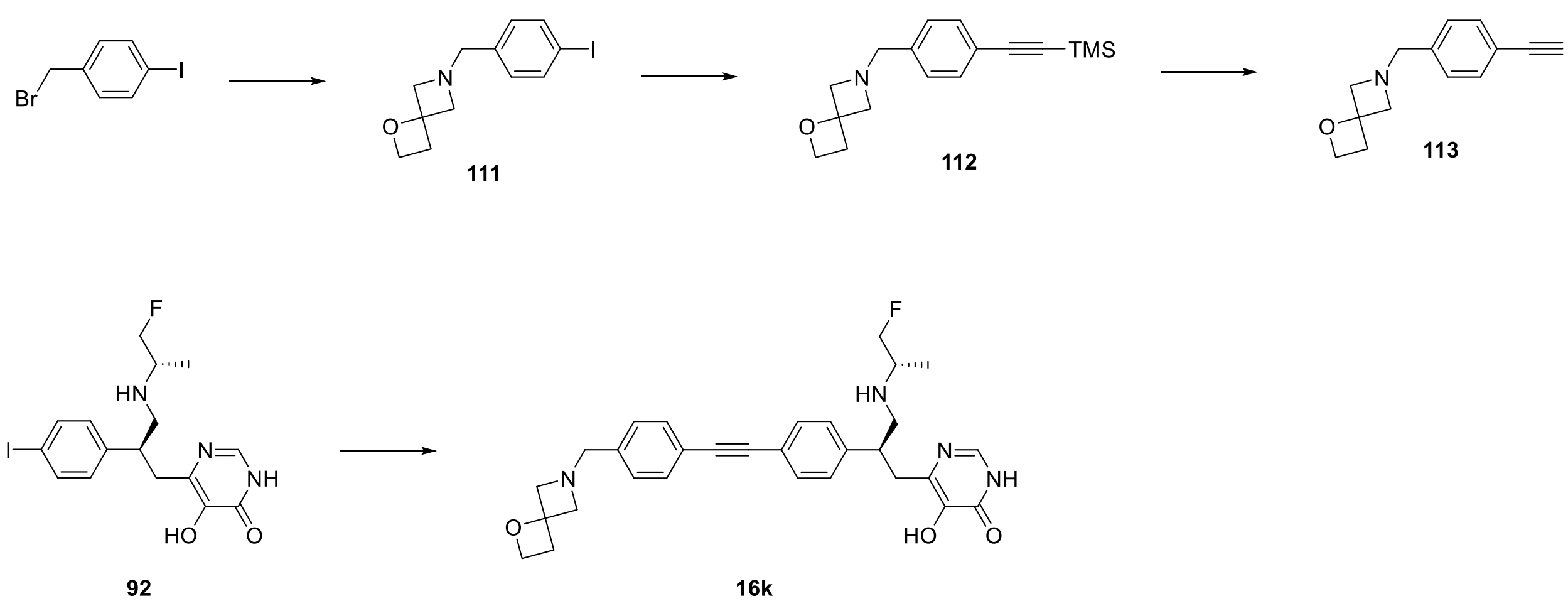

6-(4-iodobenzyl)-1-oxa-6-azaspiro[3.3]heptane (111):

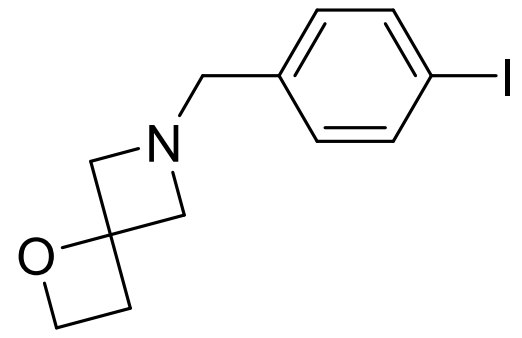

111

Compound **111** was generated from 1-oxa-6-azaspiro[3.3]heptane oxalate and 1-(bromomethyl)-4-iodobenzene and utilizing General Procedure J (300 mg, 71%). LC-MS (m/z): 316.0 $[M+H]^+$.

6-{4-[(trimethylsilyl)ethynyl]benzyl}-1-oxa-6-azaspiro[3.3]heptane (112):

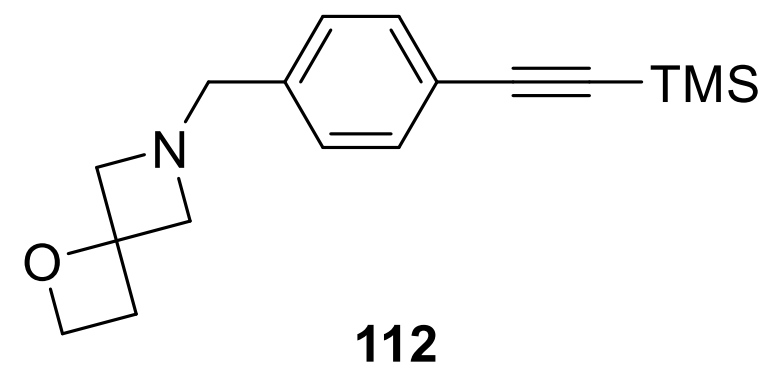

112

Compound **112** was prepared from **111** and ethynyltrimethylsilane utilizing General Procedure B (200 mg, 74%). LC-MS (m/z): 286.2 $[M+H]^+$.

6-(4-ethynylbenzyl)-1-oxa-6-azaspiro[3.3]heptane (113):

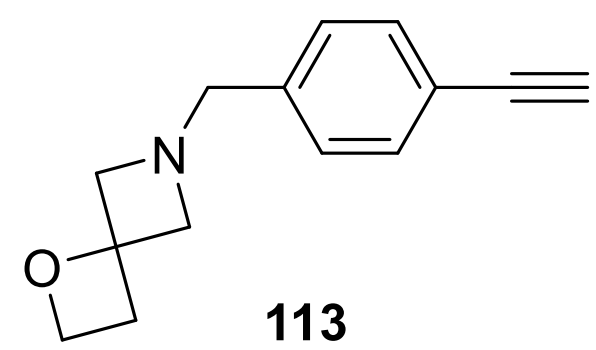

113

Compound **113** was prepared from **112** utilizing General Procedure I (120 mg, crude). LC-MS (m/z): 214.1 $[M+H]^+$.

6-{(*S*)-2-[4-({4-[(1-oxa-6-azaspiro[3.3]heptan-6-yl)methyl]phenyl}ethynyl)phenyl]-3-[((*S*)-1-fluoropropan-2-yl)amino]propyl}-5-hydroxypyrimidin-4(3H)-one (16k):

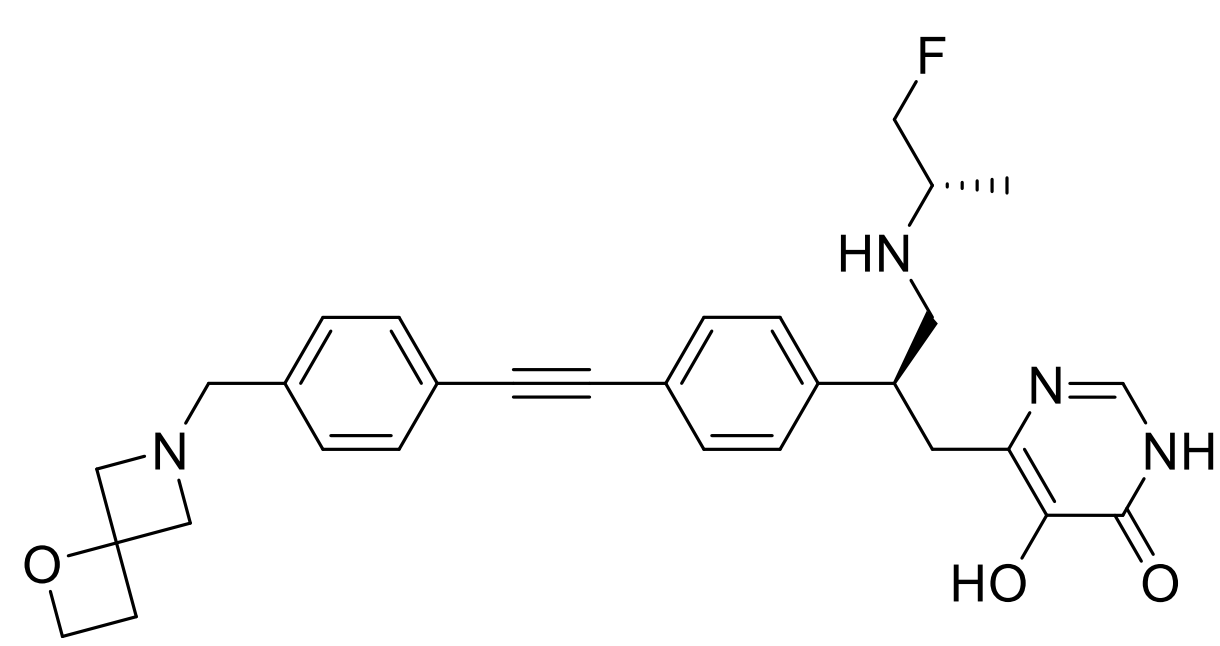

16k

Compound **16k** was obtained from **92** and **113** utilizing General Procedure B with DMF as the solvent (10 mg, 3% over two steps). LC-MS (m/z): 517.3 $[M+H]^+$. ^{1}H-NMR (DMSO-d_6): δ 12.37 (br s, 1H), 8.96 (br s, 1H), 8.13 (s, 1H), 7.67 (s, 1H), 7.50-7.44 (m, 4H), 7.35-7.29 (m, 4H), 4.49-

4.35 (m, 4H), 3.73-3.72 (m, 4H), 3.52-3.50 (m, 4H), 3.33-3.19 (m, 2H), 2.89 (d, J = 6.92 Hz, 2H), 2.77 (t, J = 7.48 Hz, 2H), 1.16 (d, J = 6.40 Hz, 3H).

Procedures for the preparation of Compound 17b

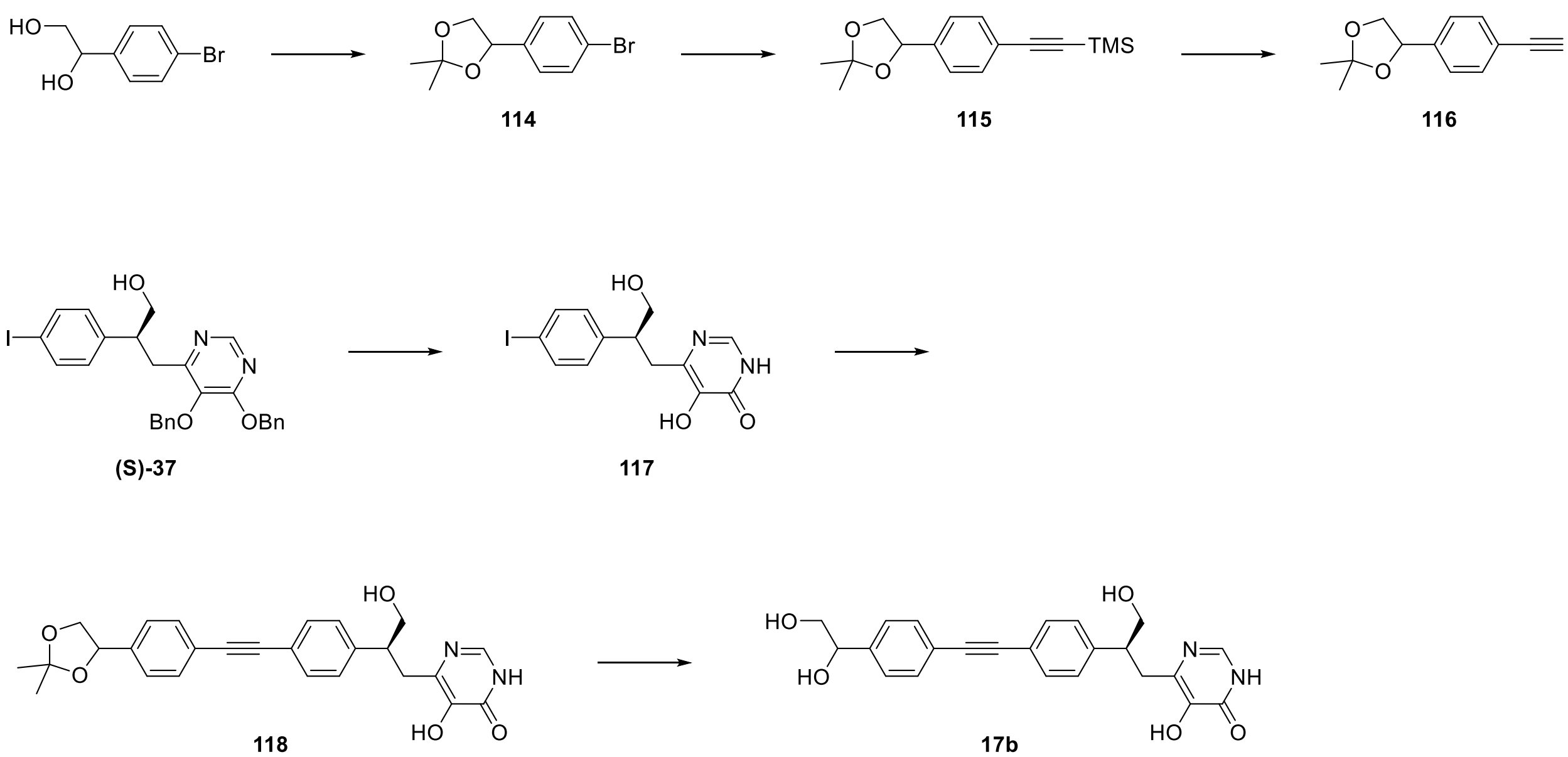

4-(4-bromophenyl)-2,2-dimethyl-1,3-dioxolane (114):

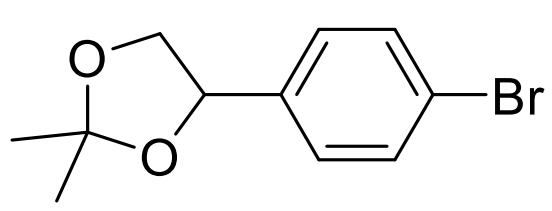

114

To a stirred solution of 1-(4-bromophenyl)ethane-1,2-diol (0.70 g, 3.2 mmol) in 2,2-dimethoxypropane (30 mL) under nitrogen, *p*-toluenesulfonic acid (70 mg) was added and the reaction mixture was heated at 50 °C for 4 h. After completion of the reaction, the reaction mixture was diluted with DCM and washed with 10% $NaHCO_3$, water and brine solution. The organic layer was dried over Na_2SO_4, filtered and concentrated under reduced pressure to afford **114** (0.82 g, 99%). LC-MS: Product did not ionize.

{[4-(2,2-dimethyl-1,3-dioxolan-4-yl)phenyl]ethynyl}trimethylsilane (115):

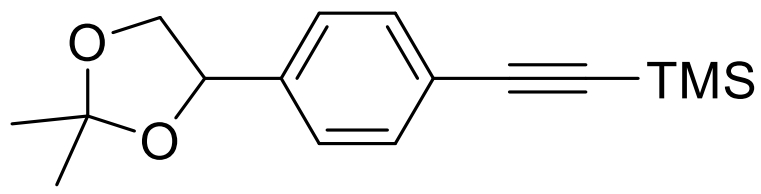

115

Compound **115** was prepared from **114** and ethynyltrimethylsilane utilizing General Procedure B with DMF as the solvent (650 mg, 76%). LC-MS: Product did not ionize.

4-(4-ethynylphenyl)-2,2-dimethyl-1,3-dioxolane (116):

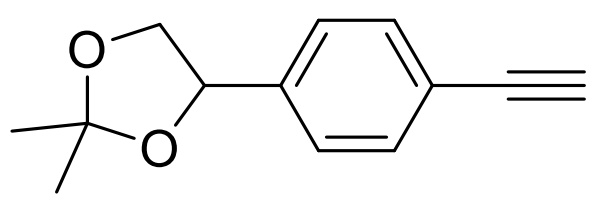

116

Compound **116** was prepared from **115** utilizing General Procedure I (470 mg, 99%). LC-MS: Product did not ionize.

(*S*)-5-hydroxy-6-[3-hydroxy-2-(4-iodophenyl)propyl]pyrimidin-4(3H)-one (117):

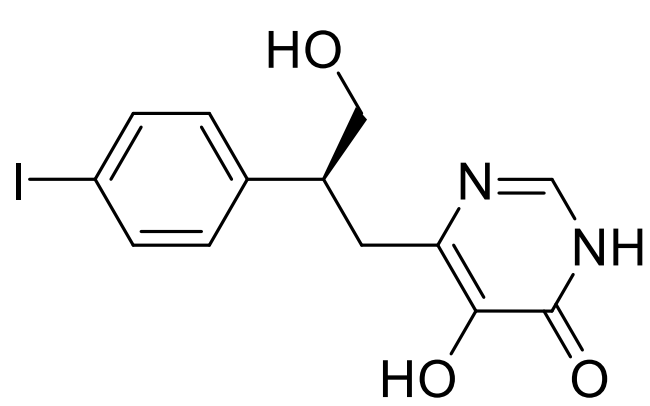

117

Compound **117** was generated from **(*S*)-37** utilizing General Procedure D (210 mg, 77%). LC-MS (m/z): 373.0 $[M+H]^+$.

6-[(2*S*)-2-(4-{[4-(2,2-dimethyl-1,3-dioxolan-4-yl)phenyl]ethynyl}phenyl)-3-hydroxypropyl]-5-hydroxypyrimidin-4(3H)-one (118):

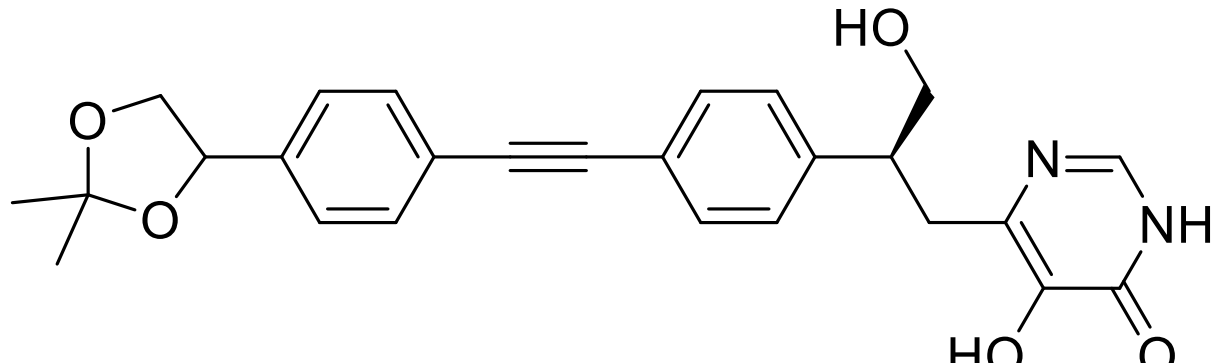

118

Compound **118** was generated from **116** and **117** utilizing General Procedure B with DMF as the solvent (210 mg, 77%). LC-MS (m/z): 447.2 $[M+H]^+$.

6-[(2*S*)-2-(4-{[4-(1,2-dihydroxyethyl)phenyl]ethynyl}phenyl)-3-hydroxypropyl]-5-hydroxypyrimidin-4(3H)-one (17b):

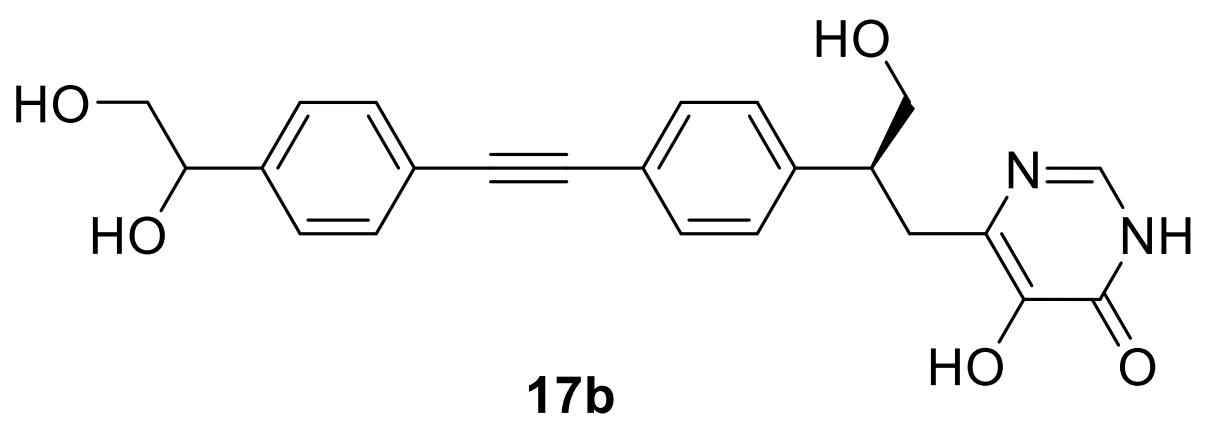

17b

To a solution of **118** (0.33 g, 0.78 mmol) in 1,4-dioxane (5 mL), 1.5 N HCl (5 mL) was added and the reaction mixture stirred at 25 °C for 2 h. After completion of the reaction, the solvent was removed under reduced pressure and the crude product purified by reverse phase HPLC to get **17b** (160 mg, 52%). LC-MS (m/z): 407.1 $[M+H]^+$. ^{1}H-NMR (DMSO-d_6): δ 12.37 (br s, 1H), 9.03 (br s, 1H), 7.65 (s, 1H), 7.47 (d, J = 8.0 Hz, 2H), 7.40-7.37 (m, 4H), 7.23 (d, J = 8.0 Hz, 2H), 5.34 (d, J = 4.40 Hz, 1H), 4.77-4.71 (m, 2H), 4.58-4.54 (m, 1H), 3.58 (s, 2H), 3.45-3.42 (m, 2H), 3.30-3.29 (m, 1H), 2.86-2.84 (m, 2H).

Procedures for the preparation of Compound 17k

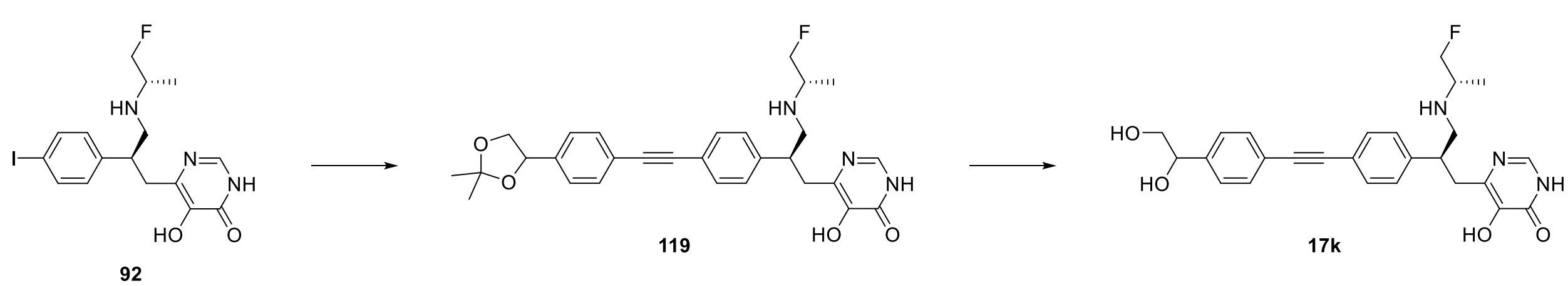

6-[(2*S*)-2-(4-{[4-(2,2-dimethyl-1,3-dioxolan-4-yl)phenyl]ethynyl}phenyl)-3-({(*S*)-1-fluoropropan-2-yl}amino)propyl]-5-hydroxypyrimidin-4(3H)-one (119):

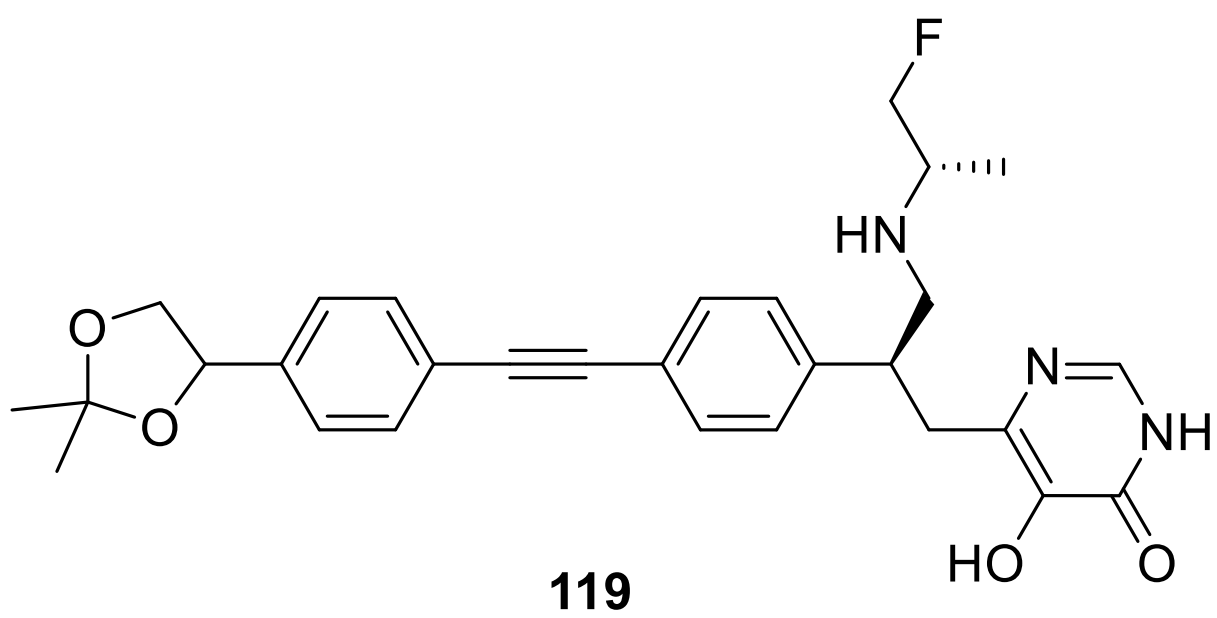

119

Compound **119** was generated from **92** and **116** utilizing General Procedure B with DMF as the solvent (200 mg, crude). LC-MS (m/z): 506.1 $[M+H]^+$.

6-[(2*S*)-2-(4-{[4-(1,2-dihydroxyethyl)phenyl]ethynyl}phenyl)-3-({(*S*)-1-fluoropropan-2-yl}amino)propyl]-5-hydroxypyrimidin-4(3H)-one (17k):

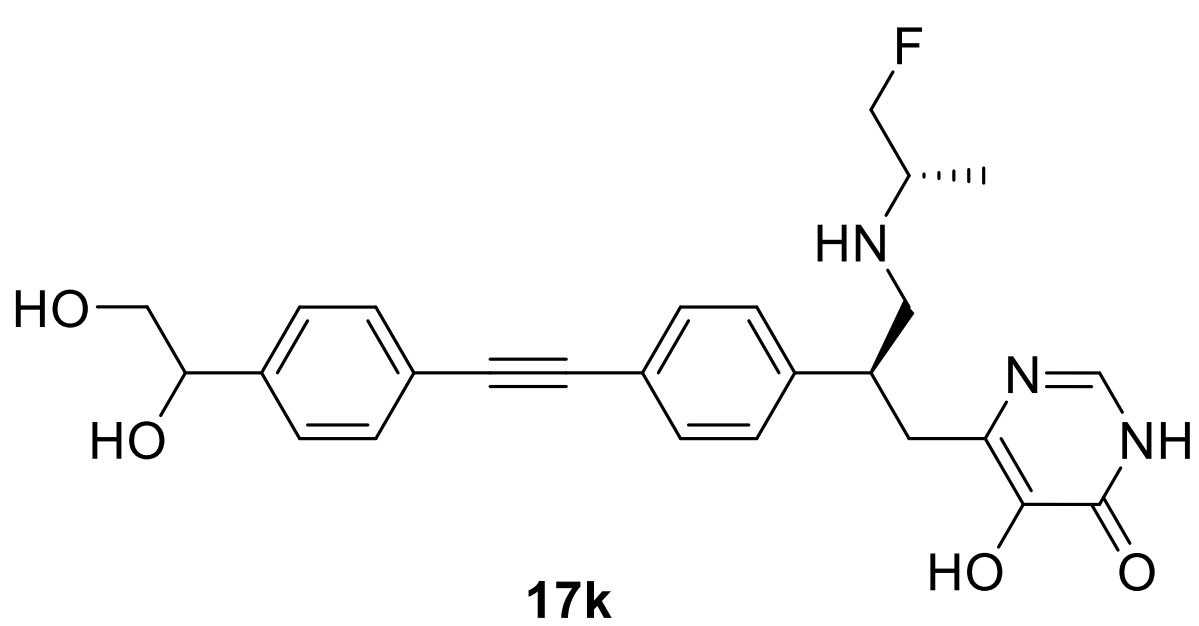

17k

To a solution of **119** (0.33 g, 0.78 mmol) in 1,4-dioxane (5 mL), 1.5 N HCl (5 mL) was added and the reaction mixture stirred at 25 °C for 2 h. After completion of the reaction, the solvent was removed under reduced pressure and the crude product purified by reverse phase HPLC to get **17k** (30 mg, 17% over two steps). LC-MS (m/z): 466.2 $[M+H]^+$. ^{1}H-NMR (DMSO-d_6): δ 7.65 (s, 1H), 7.47-7.41 (m, 4H), 7.36 (d, J = 8.4 Hz, 2H), 7.25 (d, J = 8.0 Hz, 2H), 4.55 (t, J = 6.0 Hz, 1H), 4.37-4.25 (m, 2H), 3.44-3.38 (m, 3H), 3.40-3.04 (m, 3H), 2.86 (d, J = 8.0 Hz, 2H), 1.03 (d, J = 6.40 Hz, 3H).

Procedures for the preparation of Compound 18b

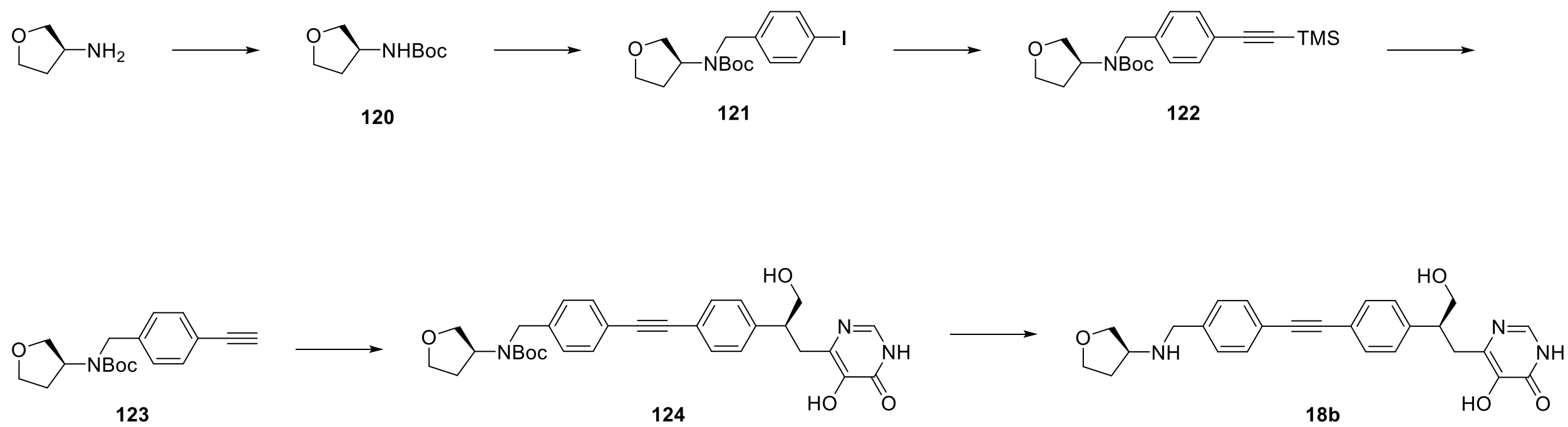

***tert*-butyl-(*S*)-(tetrahydrofuran-3-yl)carbamate (120):**

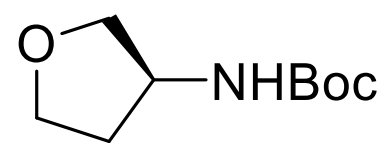

120

(*S*)-tetrahydrofuran-3-amine hydrochloride was converted to **120** utilizing General Procedure G (1.8 g, 79%). LC-MS: Product did not ionize.

***tert*-butyl-(*S*)-(4-iodobenzyl)(tetrahydrofuran-3-yl)carbamate (121):**

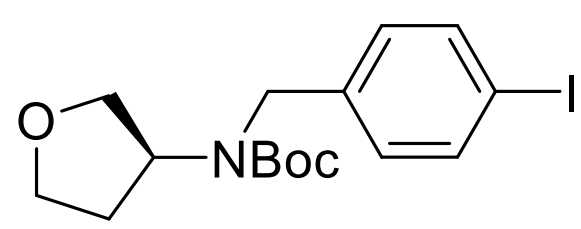

121

Compound **121** was generated from **120** and 1-(bromomethyl)-4-iodobenzene and utilizing General Procedure J (2.7 g, 79%). LC-MS (m/z): 304.0 [M-Boc$^-$]$^+$.

***tert*-butyl-(*S*)-(tetrahydrofuran-3-yl){4-[(trimethylsilyl)ethynyl]benzyl}carbamate (122):**

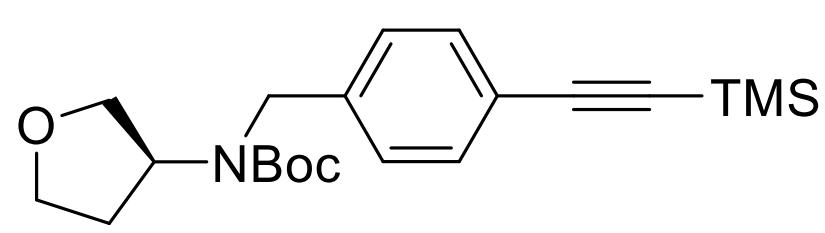

122

Compound **122** was prepared from **121** and ethynyltrimethylsilane utilizing General Procedure B (900 mg, 36%). LC-MS (m/z): 304.0 [M-Boc$^-$]$^+$.

tert-butyl-(S)-(4-ethynylbenzyl)(tetrahydrofuran-3-yl)carbamate (123):

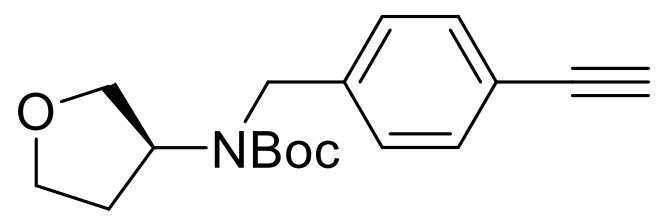

123

Compound **123** was prepared from **122** utilizing General Procedure I (440 mg, 60%). LC-MS (m/z): 200.1 $[M\text{-}Boc^{-}]^{+}$.

***tert*-butyl-[4-({4-[(*S*)-1-hydroxy-3-(5-hydroxy-6-oxo-1,6-dihydropyrimidin-4-yl)propan-2-yl]phenyl}ethynyl)benzyl]((*S*)-tetrahydrofuran-3-yl)carbamate (137):**

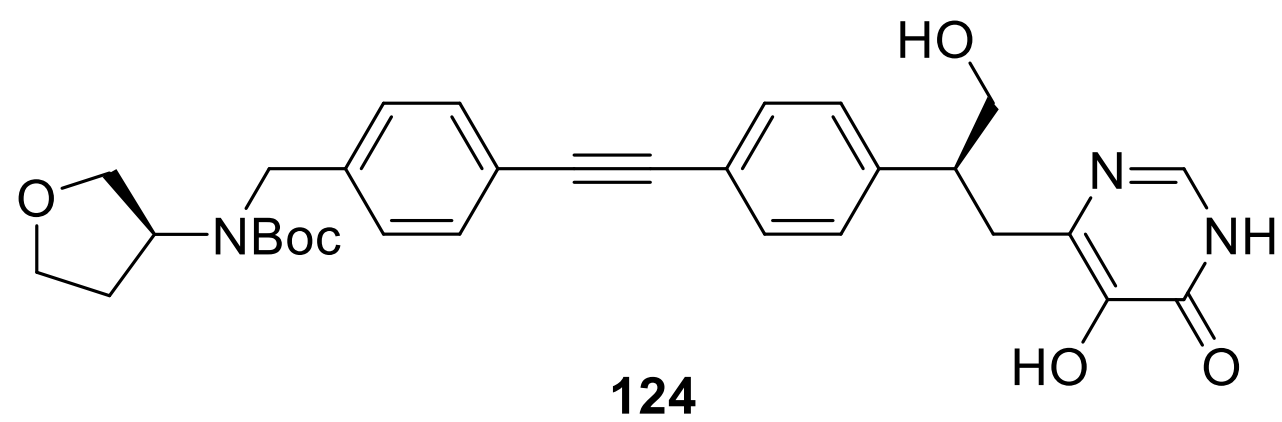

124

Compound **124** was prepared from **117** and **123** utilizing General Procedure B with DMF as the solvent (15 g, 74%). LC-MS (m/z): 546.3 $[M+H]^{+}$.

5-hydroxy-6-((*S*)-3-hydroxy-2-{4-[(4-{[((*S*)-tetrahydrofuran-3-yl)amino]methyl}phenyl)-ethynyl]phenyl}propyl)pyrimidin-4(3H)-one (18b):

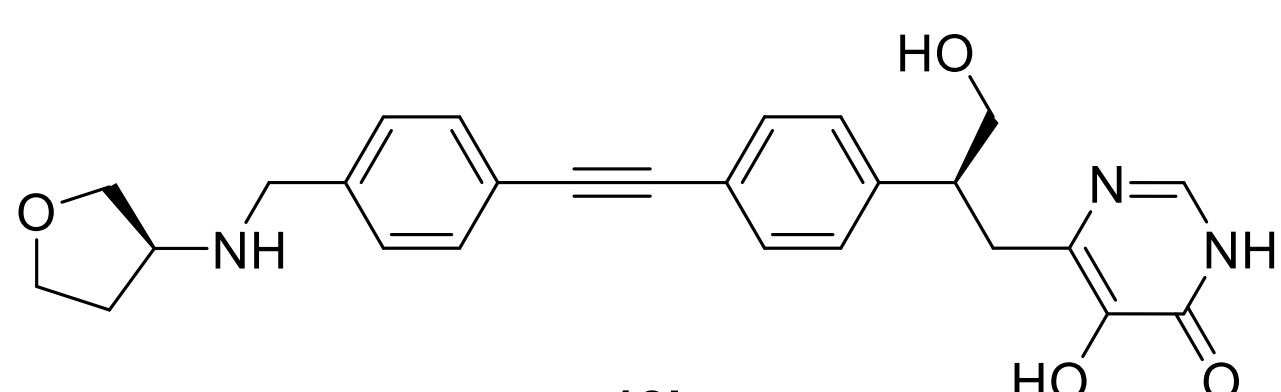

18b

To a solution of **124** (15 g, 0.028 mol) in DCM (150 mL) was added 4 M HCl in 1,4-dioxane (150 mL) and stirred at room temperature for 1 h. After completion of the reaction, the volatiles were removed under reduced pressure and the crude product was purified by reverse phase HPLC to get **18b** (9.0 g, 73%). LC-MS (m/z): 446.3 $[M+H]^{+}$. ^{1}H-NMR (DMSO-d_6): δ 9.58 (br s, 2H),

7.98 (br s, 1H), 7.64-7.59 (m, 4H), 7.43-7.41 (m, 2H), 7.27-7.25 (m, 2H), 4.20-4.19 (m, 2H), 3.95-3.91 (m, 2H), 3.89-3.81 (m, 2H), 3.80-3.59 (m, 3H), 3.33-3.29 (m, 1H), 2.92-2.86 (m, 2H), 2.24-2.22 (m, 2H).

Procedures for the preparation of Compound 18k

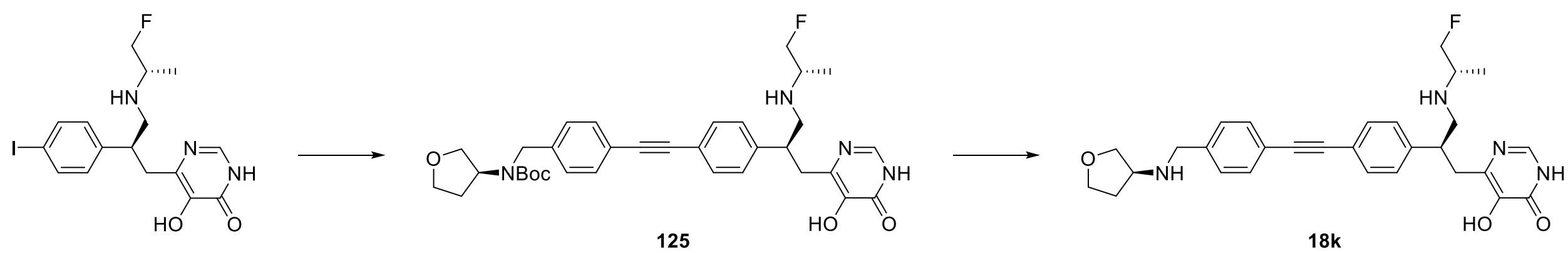

92 **125** **18k**

***tert*-butyl-{4-[(4-{(*S*)-1-[((*S*)-1-fluoropropan-2-yl)amino]-3-[5-hydroxy-6-oxo-1,6-dihydropyrimidin-4-yl]propan-2-yl}phenyl)ethynyl]benzyl}((*S*)-tetrahydrofuran-3-yl)carbamate (125):**

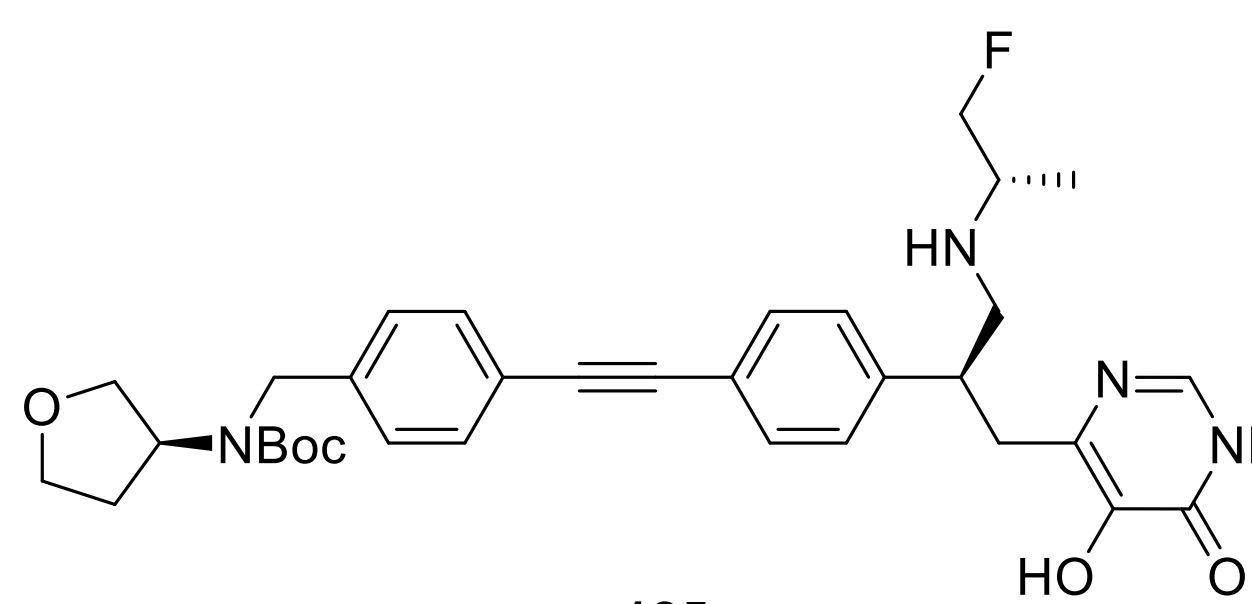

125

Compound **125** was prepared from **92** and **123** utilizing General Procedure B with DMF as the solvent (360 mg, crude). LC-MS (m/z): 605.3 $[M+H]^+$.

6-((*S*)-3-{[(*S*)-1-fluoropropan-2-yl)amino]-2-{4-[(4-{[((*S*)-tetrahydrofuran-3-yl)amino]-methyl}phenyl)ethynyl]phenyl}propyl)-5-hydroxypyrimidin-4(3H)-one (18k):

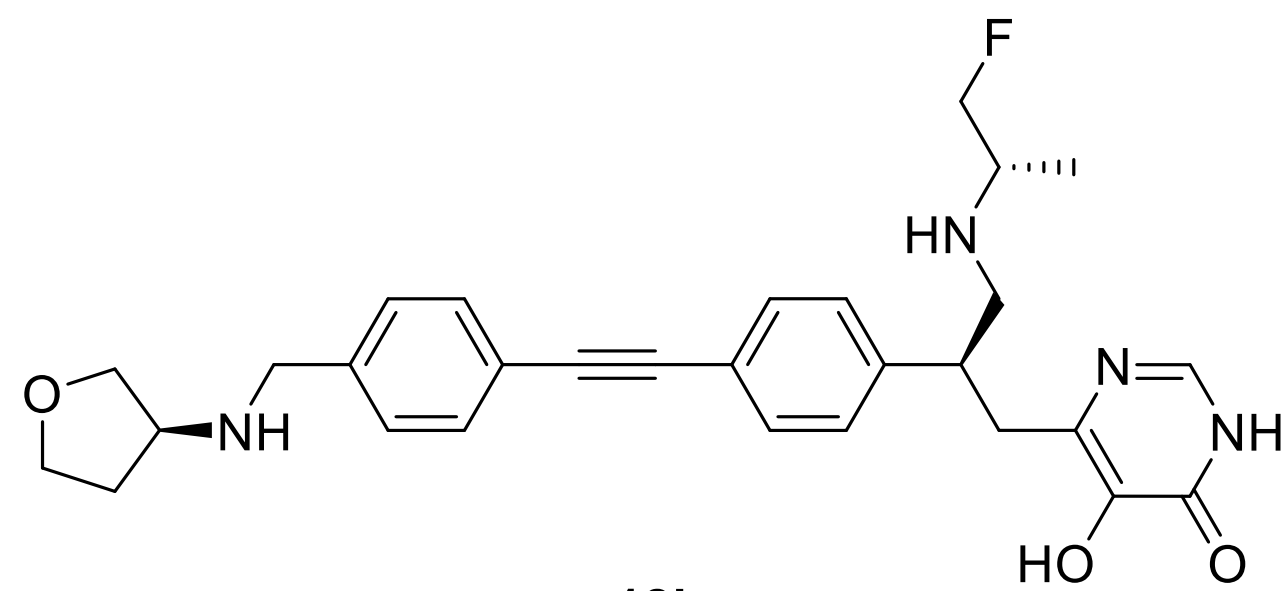

18k

To a solution of **125** (0.36 g, 0.60 mmol) in 1,2-dichloroethane (10 mL) was added 4 M HCl in 1,4-dioxane (3.6 mL) and stirred at room temperature for 1 h. After completion of the reaction, the volatiles were removed under reduced pressure and the crude product was purified by reverse phase HPLC to get **18k** (150 mg, 50% over two steps). LC-MS (m/z): 505.3 $[M+H]^+$. ^{1}H-NMR (DMSO-d_6): δ 8.15 (s, 1H), 7.68 (s, 1H), 7.56 (d, J = 8.1 Hz, 2H), 7.50-7.43 (m, 4H), 7.29 (d, J = 6.6 Hz, 2H), 4.40-4.20 (m, 2H), 4.10-3.90 (m, 2H), 3.85 (br s, 2H), 3.80-3.70 (m, 3H), 3.6.5-3.55 (m, 3H), 3.15-3.00 (m, 3H), 2.90-2.80 (m, 2H), 2.15-2.00 (m, 1H), 1.95-1.80 (m, 1H), 1.06 (d, J = 6.1 Hz, 3H).

LC/MS traces for Compounds 2, 8b, 8g, 8j, 8k, 8m, 8n, 8p-t, 10k, 11t, 12k, 13b, 13g, 13h, 13k, 13p, 13t, 14k, 16k, 18b, and 18k

```
Method info  : Column :Atlantis dC18 (50*4.6)5µ
               Mobile phase :A :0.1%Formic Acid in H2O B: ACN
               Flow Rate :1.5 ml/min
               Time (min)    %B
               0.0           05
               2.5           95
               4.0           95
               4.5           05
               6.0           05
```

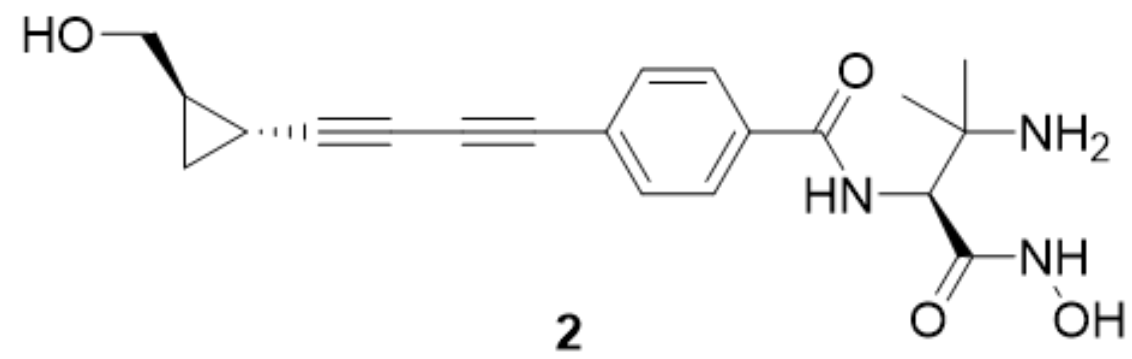

2

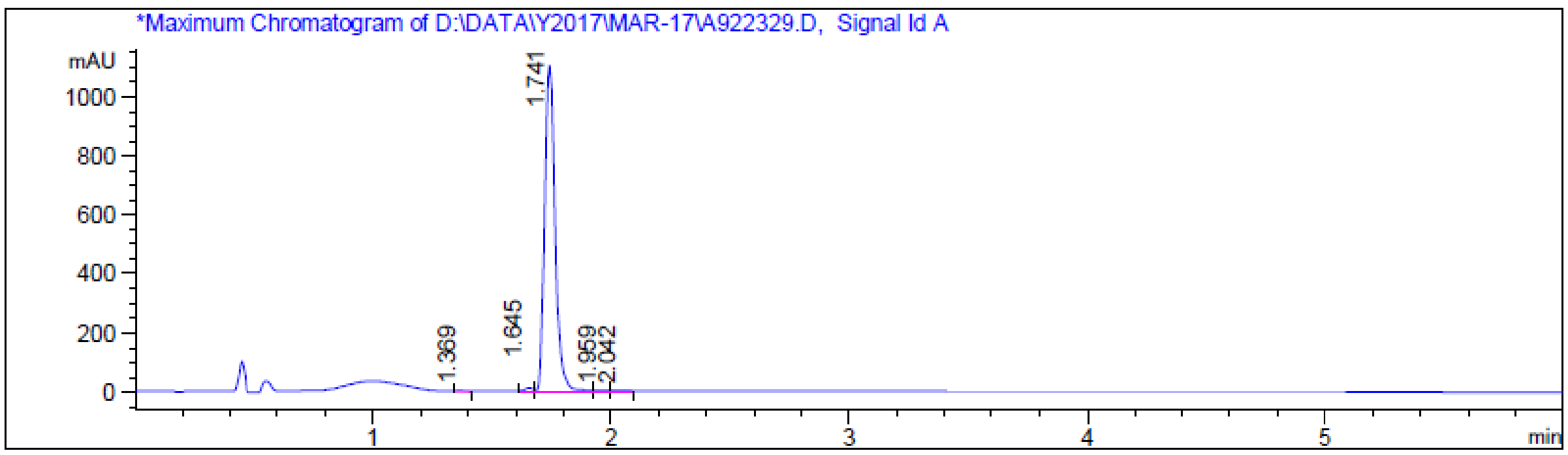

Peak No	RT min	Area	Area %
1	1.369	5.428e+000	0.158
2	1.645	2.824e+001	0.824
3	1.741	3.378e+003	98.532
4	1.959	8.315e+000	0.243
5	2.042	8.349e+000	0.244

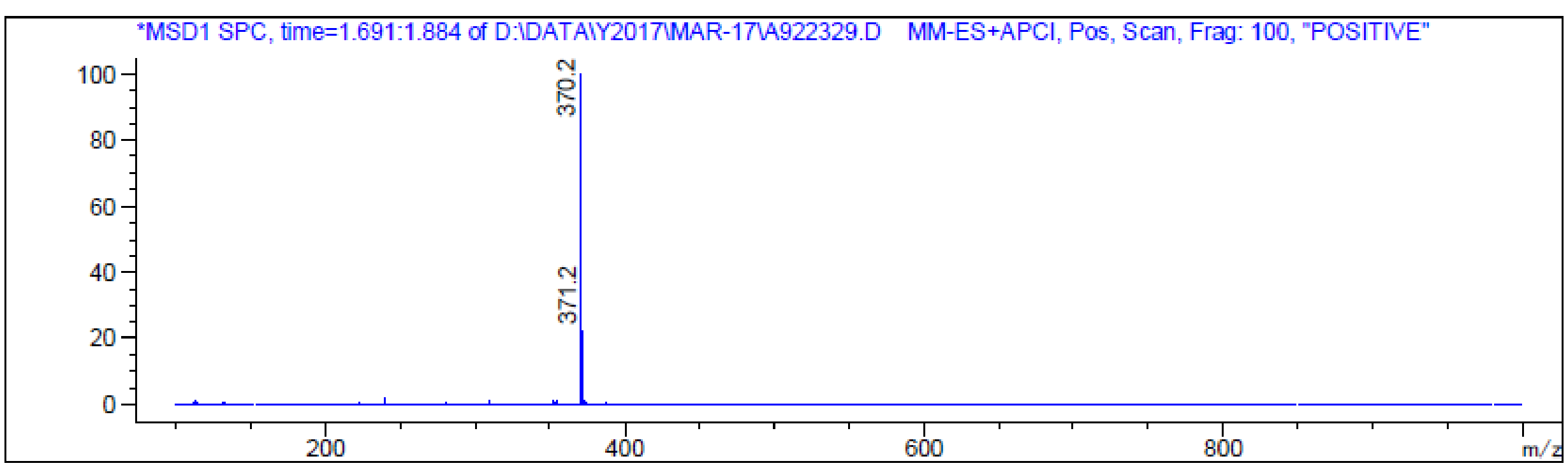

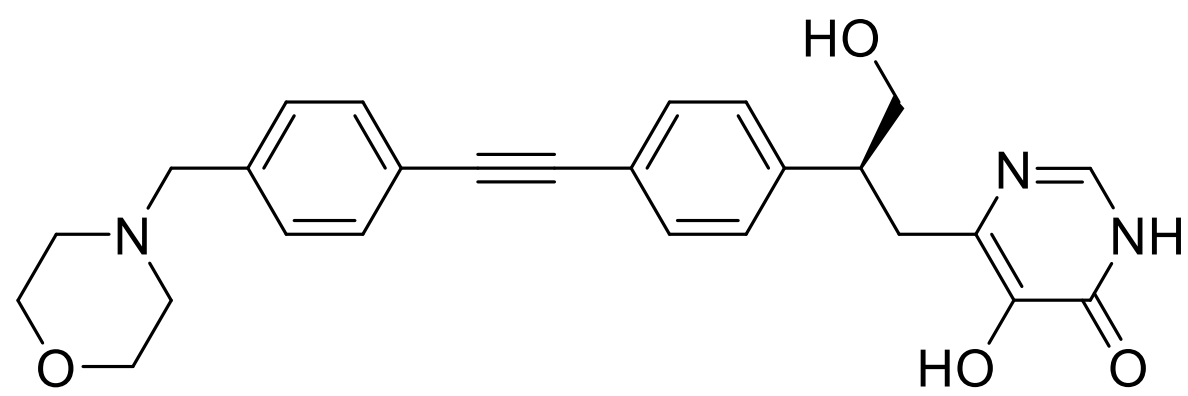

8b

```
Method info  :Column :Atlantis dC18 (50*4.6)5µ
              Mobile phase :A :0.1%Formic Acid in H2O B: ACN
              Flow Rate :1.5 ml/min
              Time(min)                  %B
              0.0                          05
              2.5                          95
              4.0                          95
              4.5                          05
              6.0                          05
```

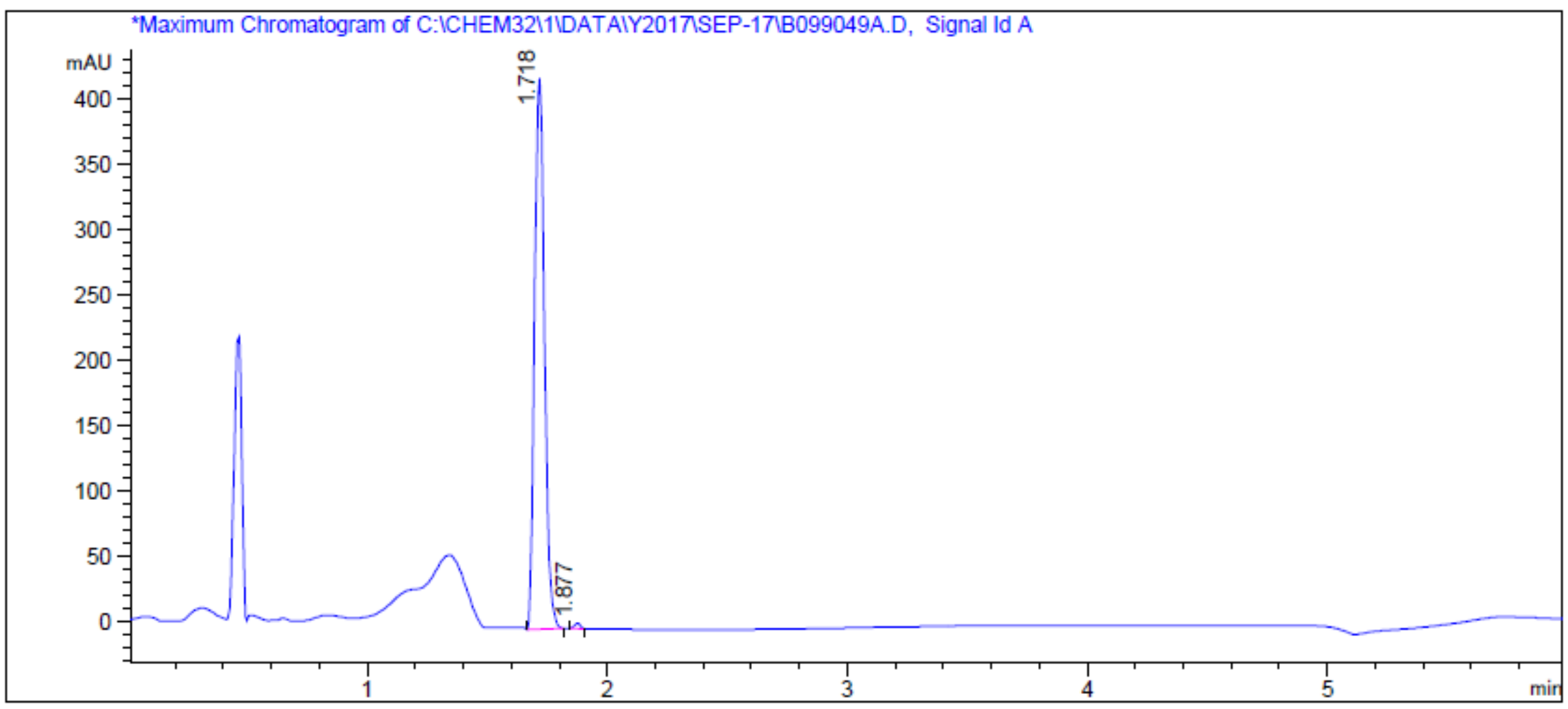

Peak No	RT min	Area	Area %
1	1.718	1.218e+003	99.414
2	1.877	7.184e+000	0.586

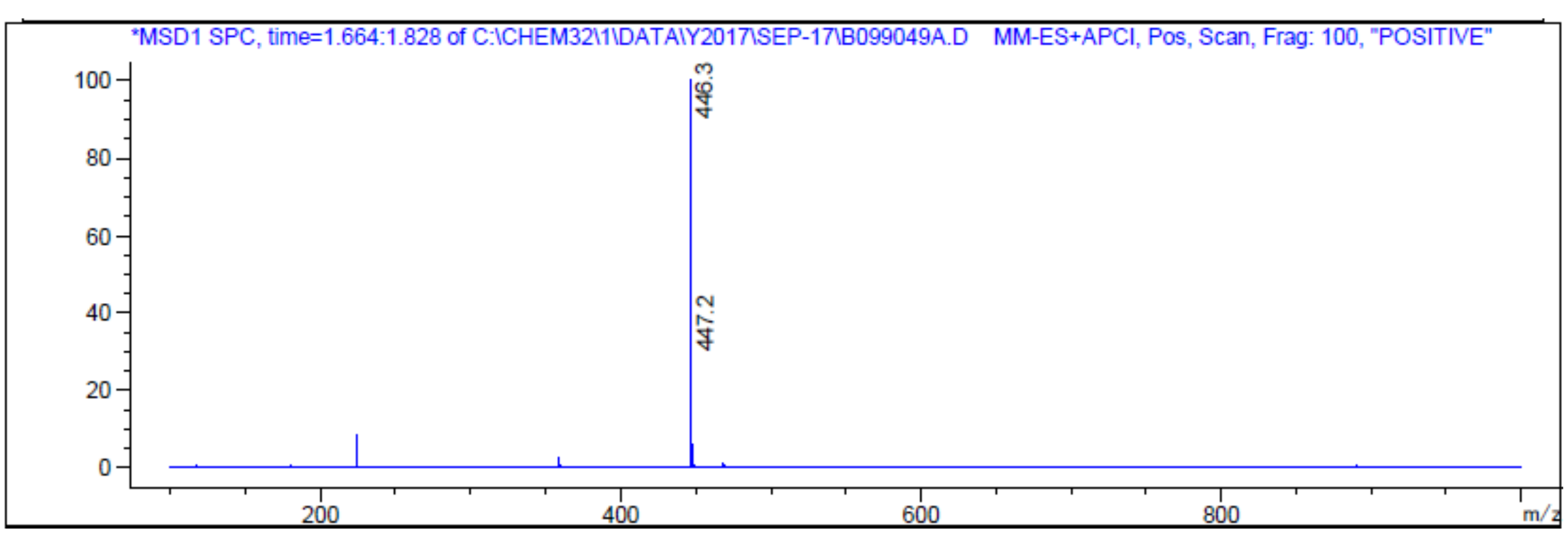

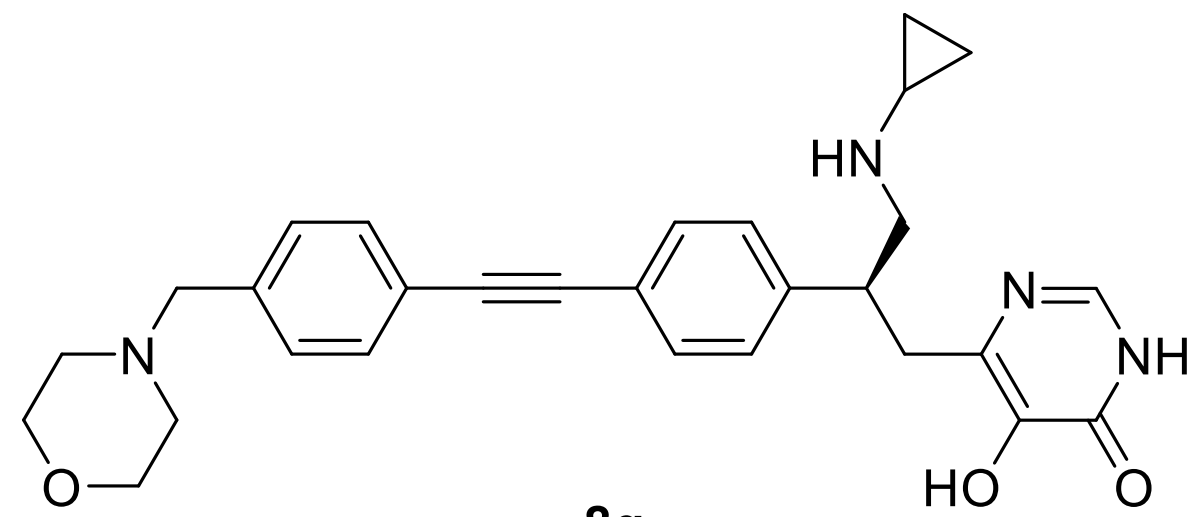

8g

```
Method info  : Column :Atlantis dC18 (50*4.6)5µ
               Mobile phase :A :0.1%Formic Acid in H2O B: ACN
               Flow Rate :1.5 ml/min
               Time (min)    %B
               0.0              05
               2.5              95
               4.0              95
               4.5              05
               6.0              05
```

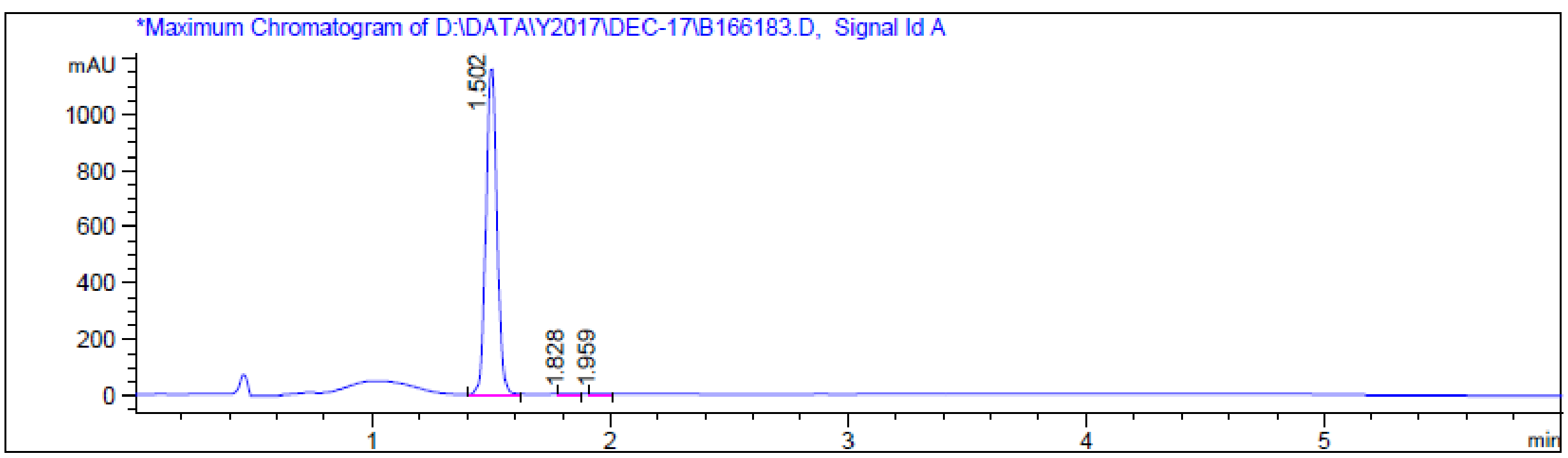

Peak No	RT min	Area	Area %
1	1.502	3.796e+003	99.683
2	1.828	6.320e+000	0.166
3	1.959	5.734e+000	0.151

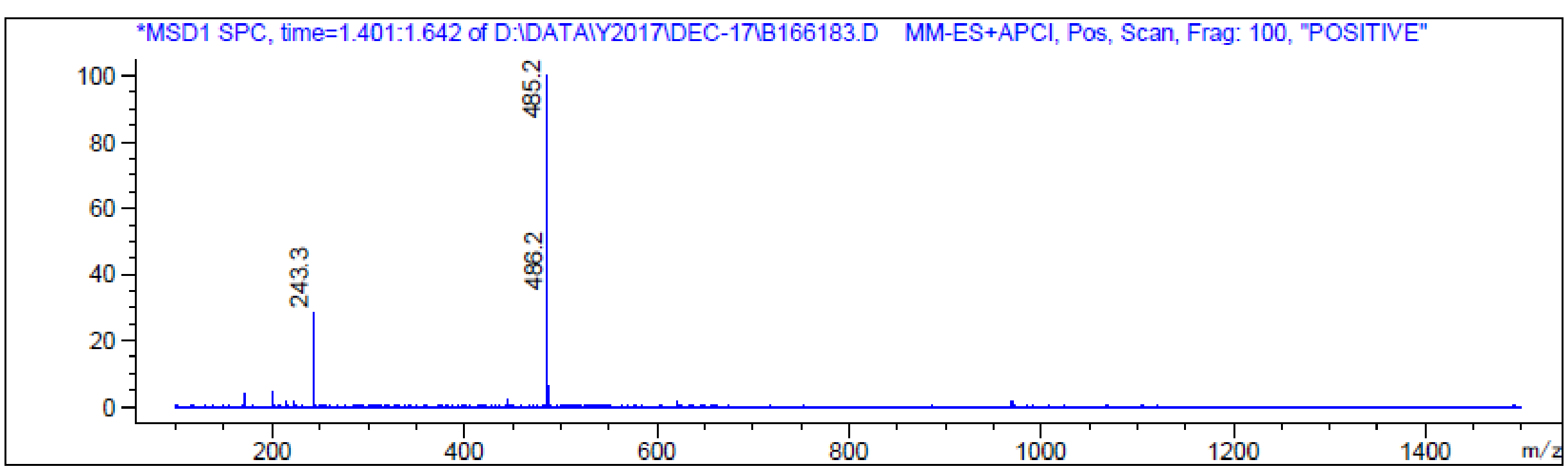

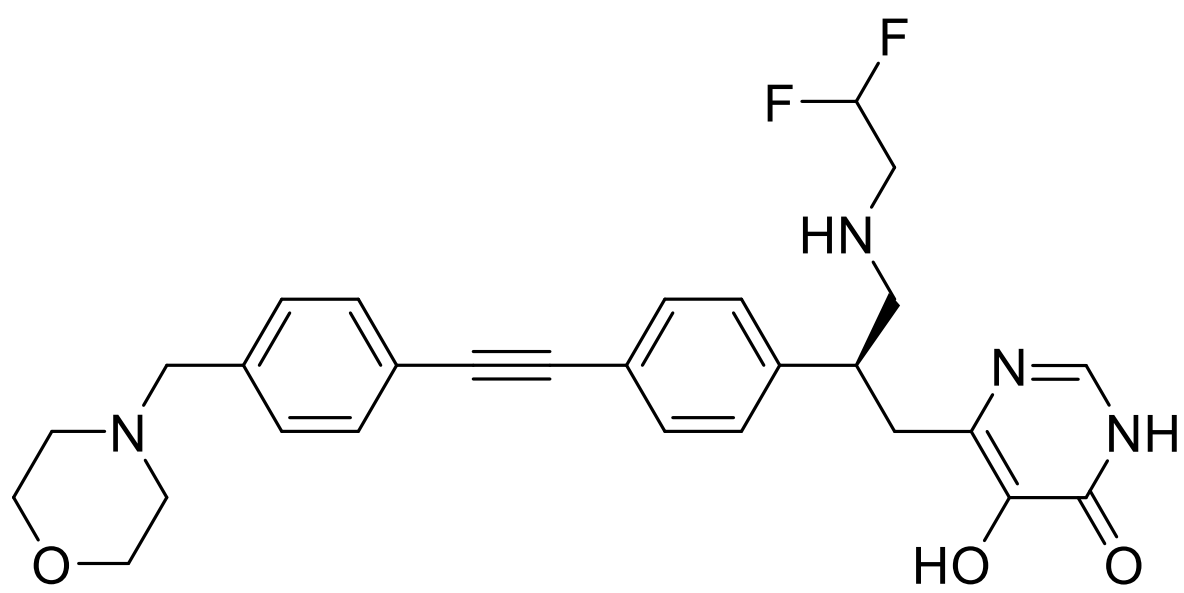

8j

```
Method info  :   Column :Atlantis dC18 (50*4.6)5
                 Mobile phase :A :0.1%Formic Acid in H2O B: ACN
                 Flow Rate :1.5 ml/min
                 Time(min) %B
                 0.0 05
                 2.5 95
                 4.0 95
                 4.5 05
                 6.0 05
```

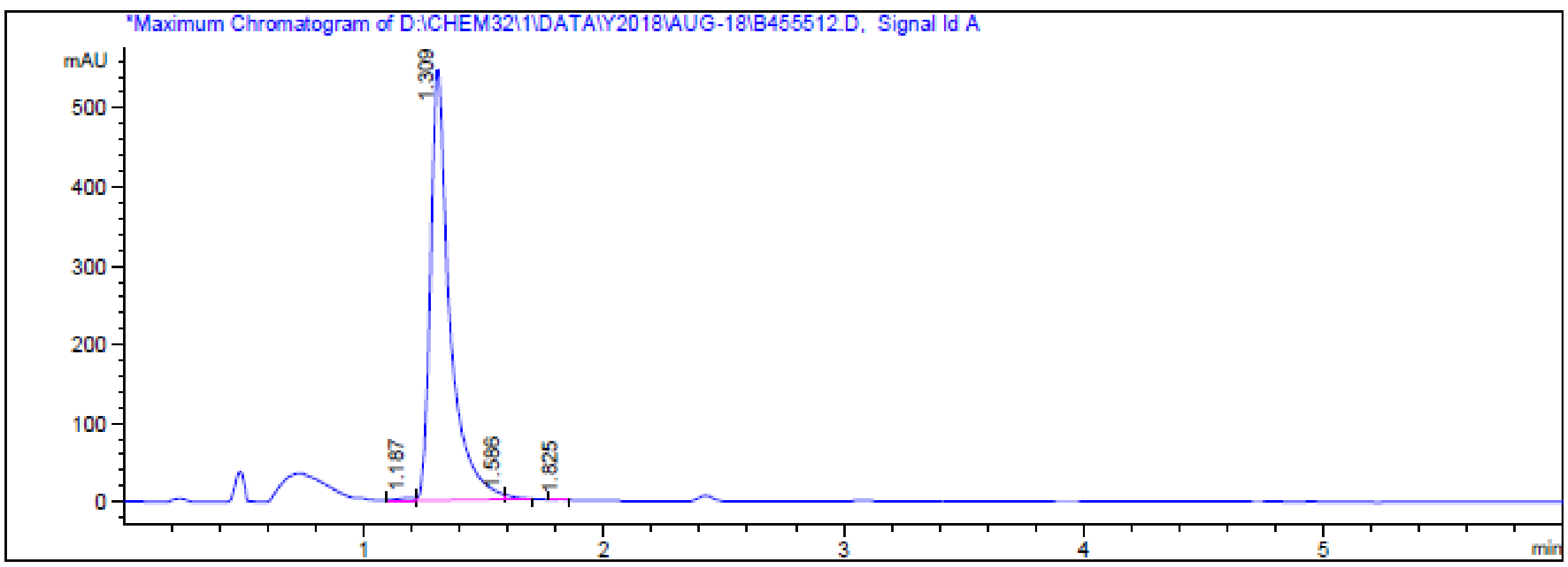

Peak No	RT min	Area	Area %
1	1.187	16.664	0.536
2	1.309	3069.922	98.820
3	1.586	17.746	0.571
4	1.825	2.239	0.072

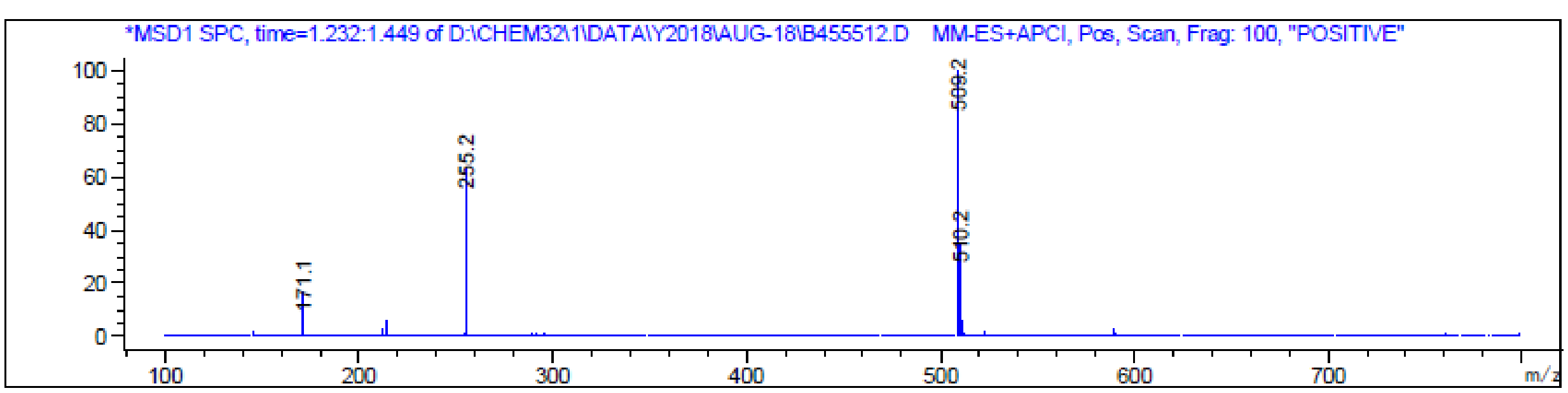

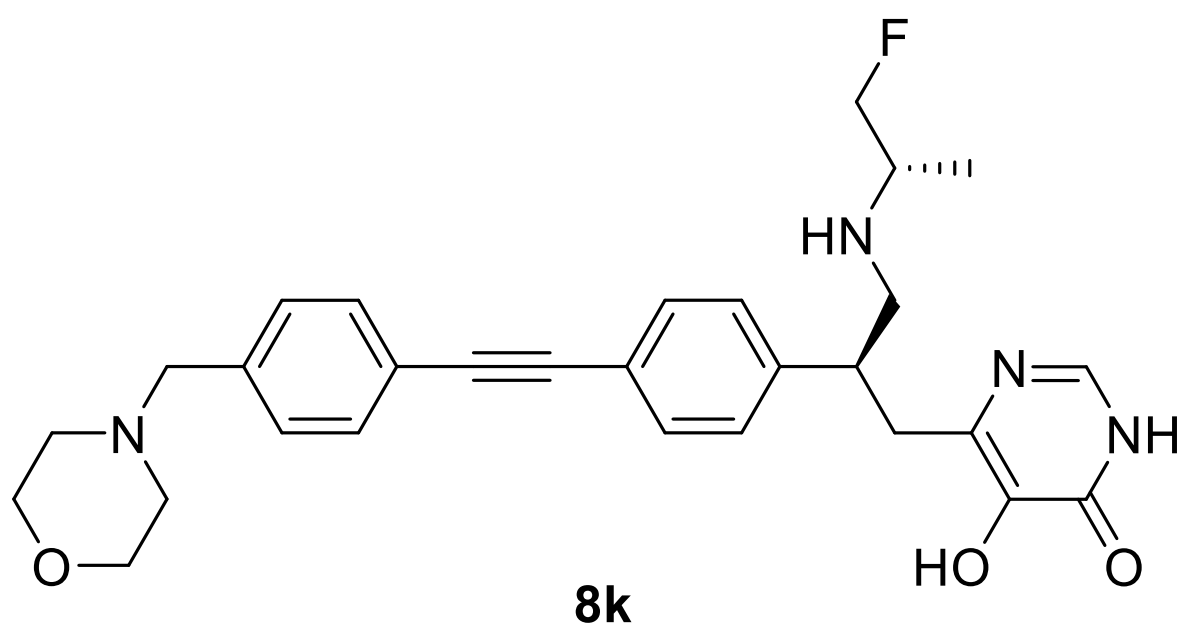

8k

```
Method info   : Column :Atlantis dC18 (50*4.6)5µ
                Mobile phase :A :0.1%Formic Acid in H2O B: ACN
                Flow Rate :1.5 ml/min
                Time (min)    %B
                0.0            05
                2.5            95
                4.0            95
                4.5            05
                6.0            05
```

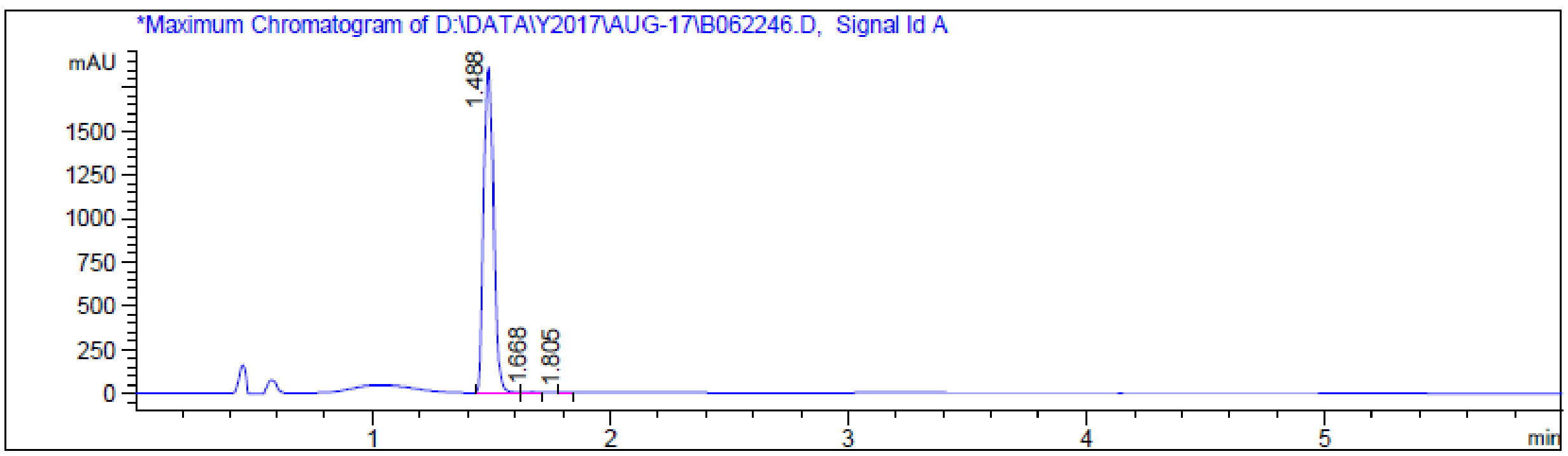

Peak No	RT min	Area	Area %
1	1.488	5.317e+003	99.697
2	1.668	1.342e+001	0.252
3	1.805	2.731e+000	0.051

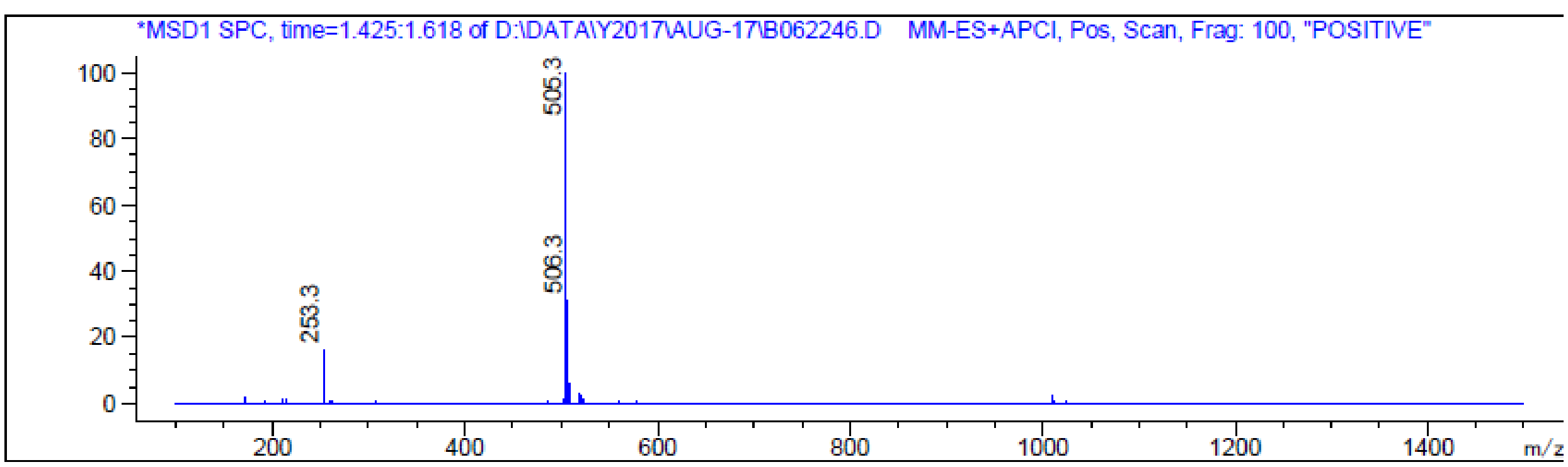

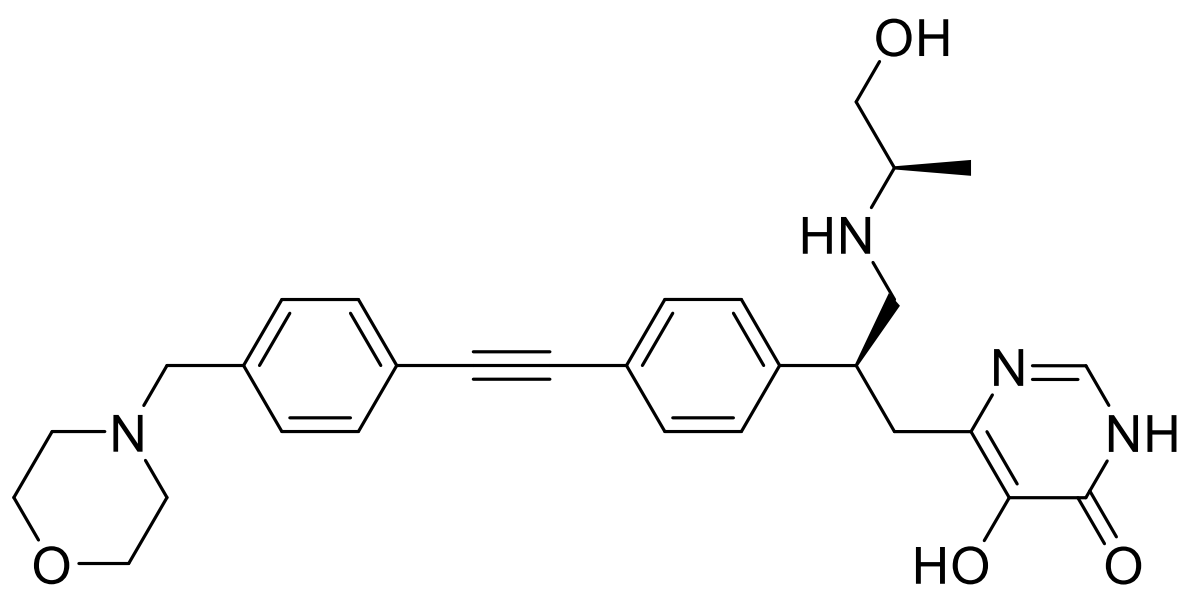

8m

```
Method info  :   Column :Atlantis dC18  (50*4.6)5µ
                 Mobile phase :A :0.1%Formic Acid in H2O B: ACN
                 Flow Rate :1.5 ml/min
                 Time(min)                 %B
                 0.0                          05
                 2.5                          95
                 4.0                          95
                 4.5                          05
                 6.0                          05
```

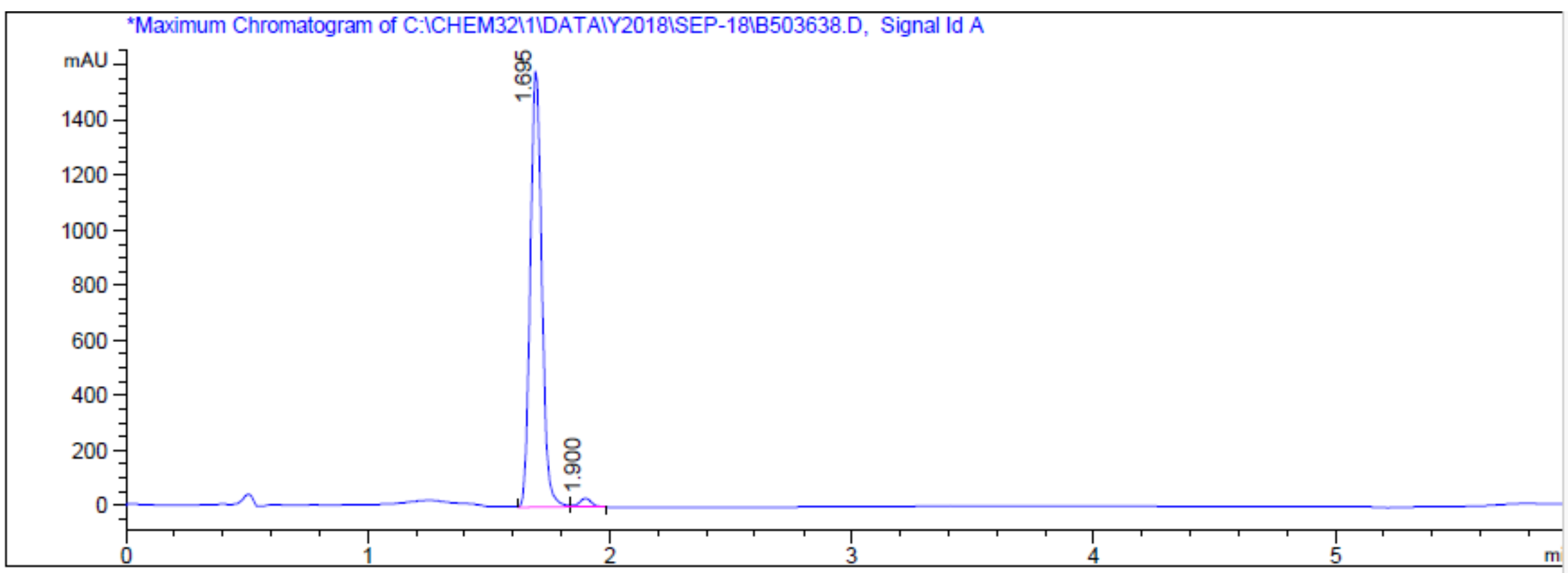

Peak No	RT min	Area	Area %
1	1.695	5263.950	98.000
2	1.900	107.403	2.000

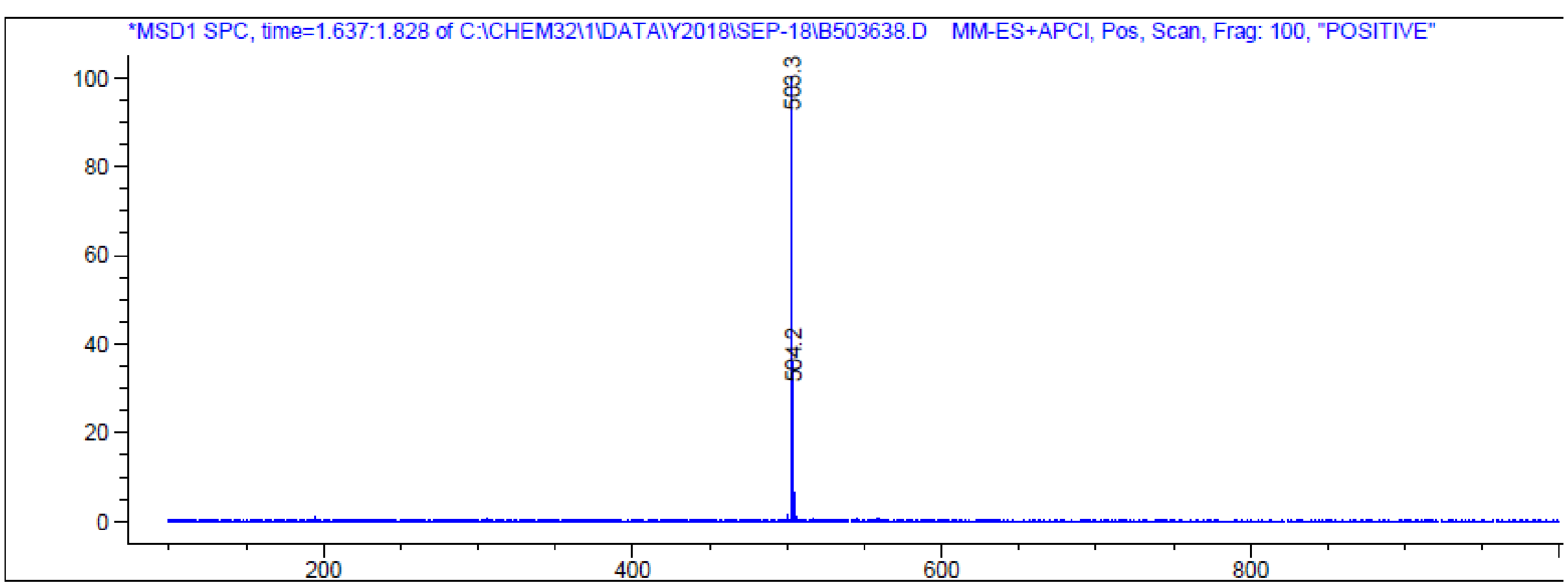

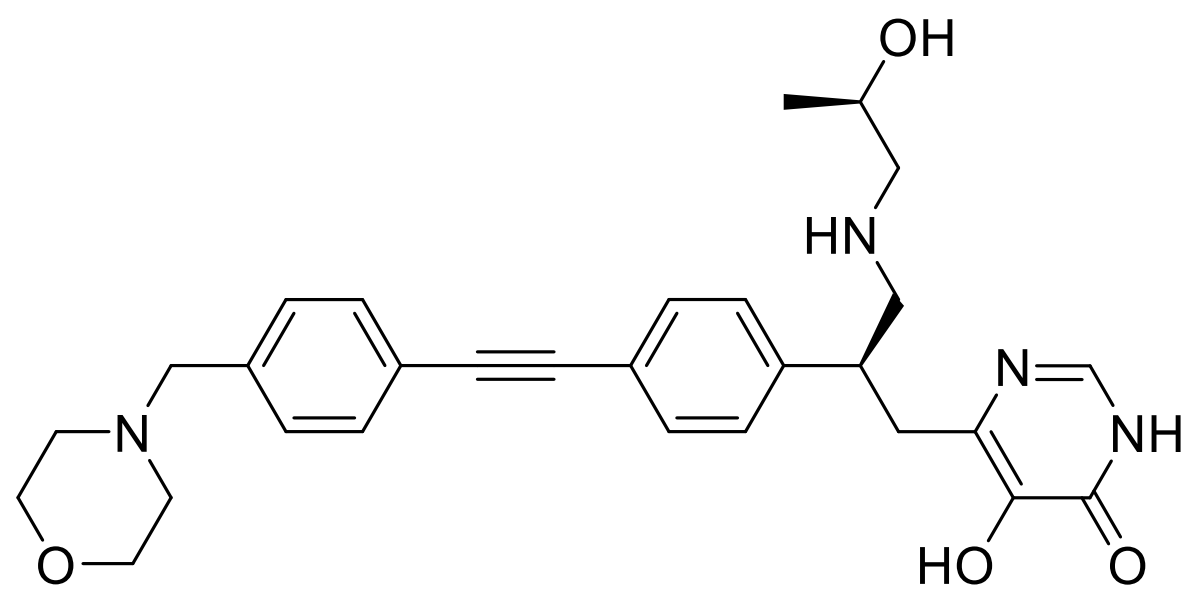

8n

```
Method info  :  Column :Atlantis dC18 (50*4.6)5µ
                Mobile phase :A :0.1%Formic Acid in H2O B: ACN
                Flow Rate :1.5 ml/min
                Time(min)                    %B
                0.0                           05
                2.5                           95
                4.0                           95
                4.5                           05
                6.0                           05
```

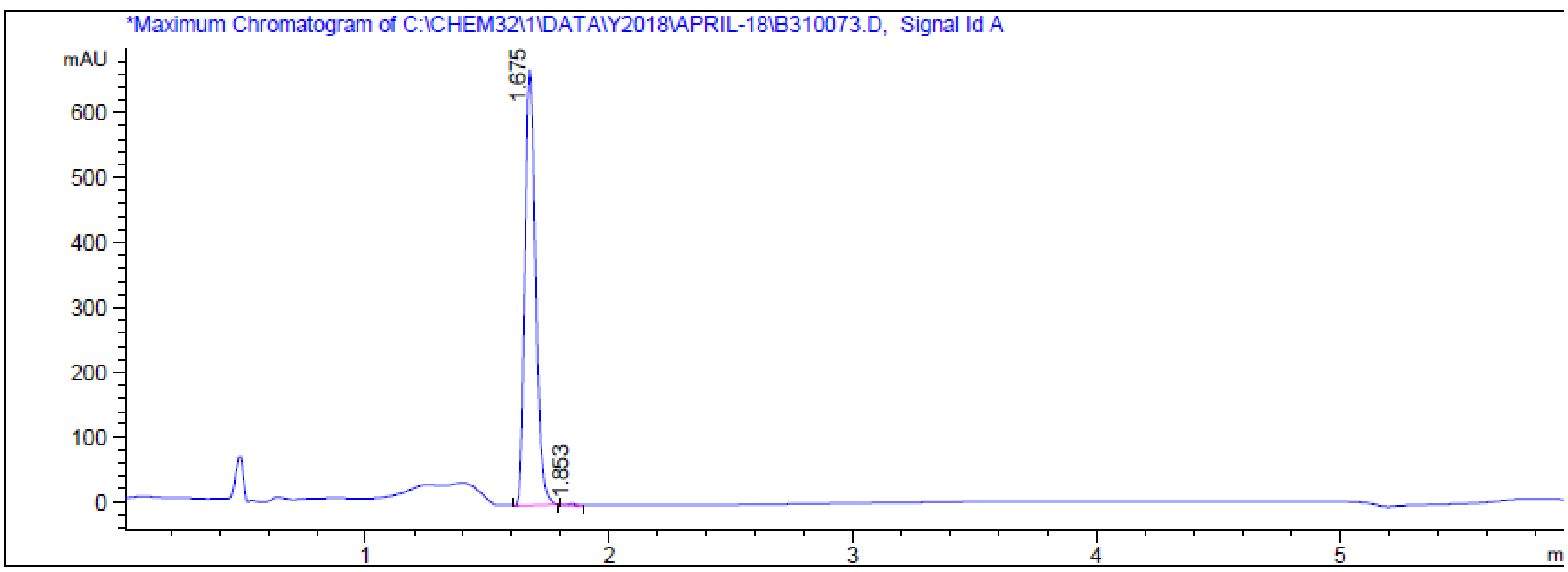

Peak No	RT min	Area	Area %
1	1.675	2122.825	99.503
2	1.853	10.599	0.497

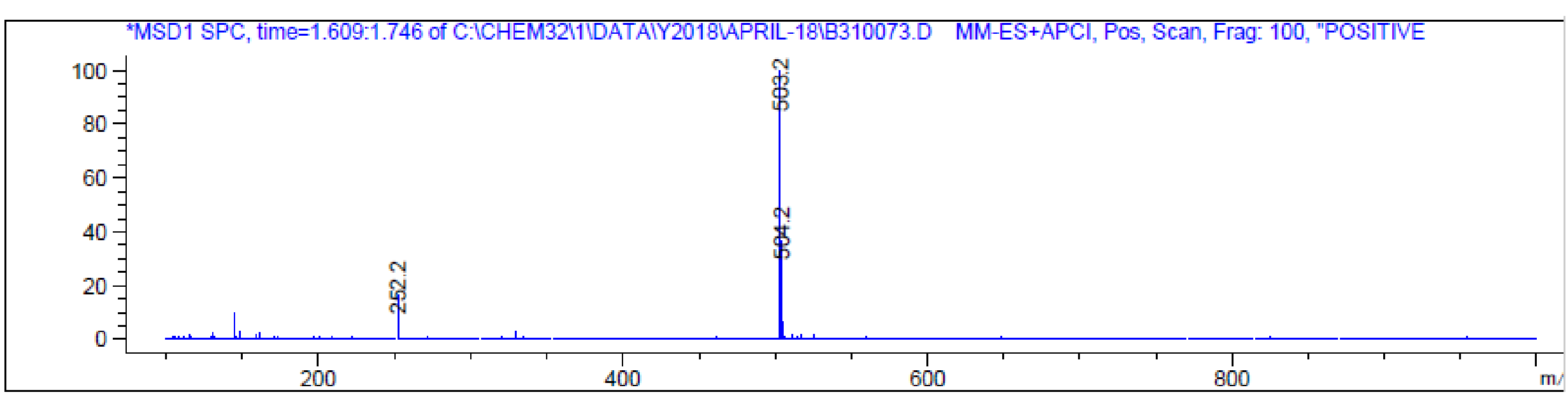

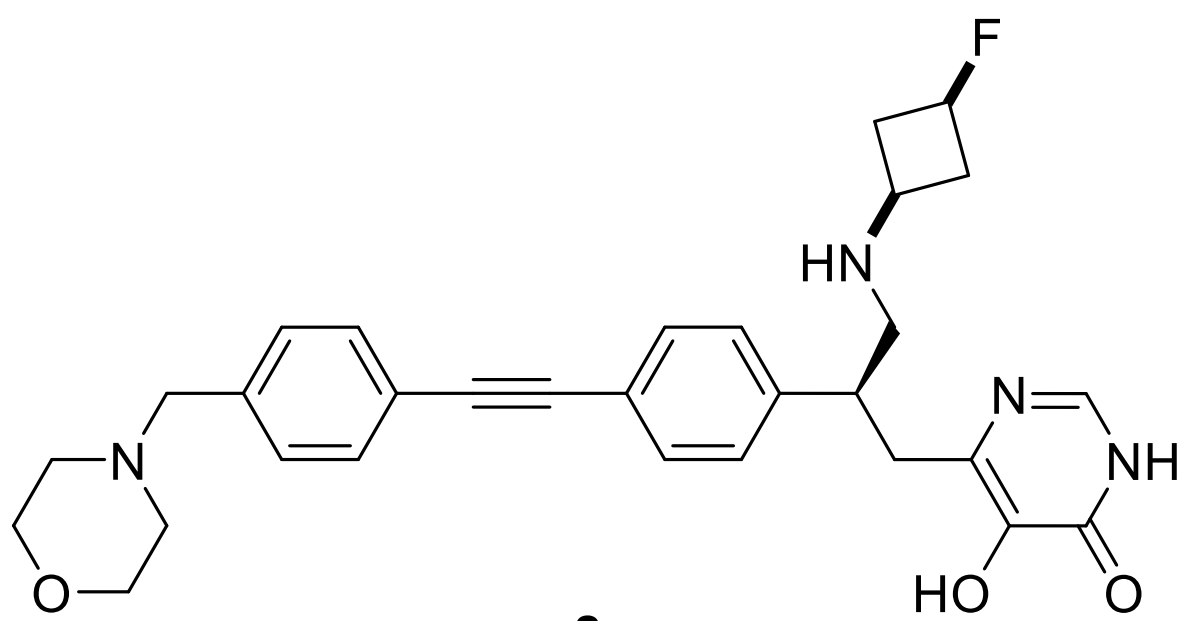

8p

```
Method info   : Column :Atlantis dC18 (50*4.6)5
                Mobile phase :A :0.1%Formic Acid in H2O B: ACN
                Flow Rate :1.5 ml/min
                Time(min) %B
                0.0 05
                2.5 95
                4.0 95
                4.5 05
                6.0 05
```

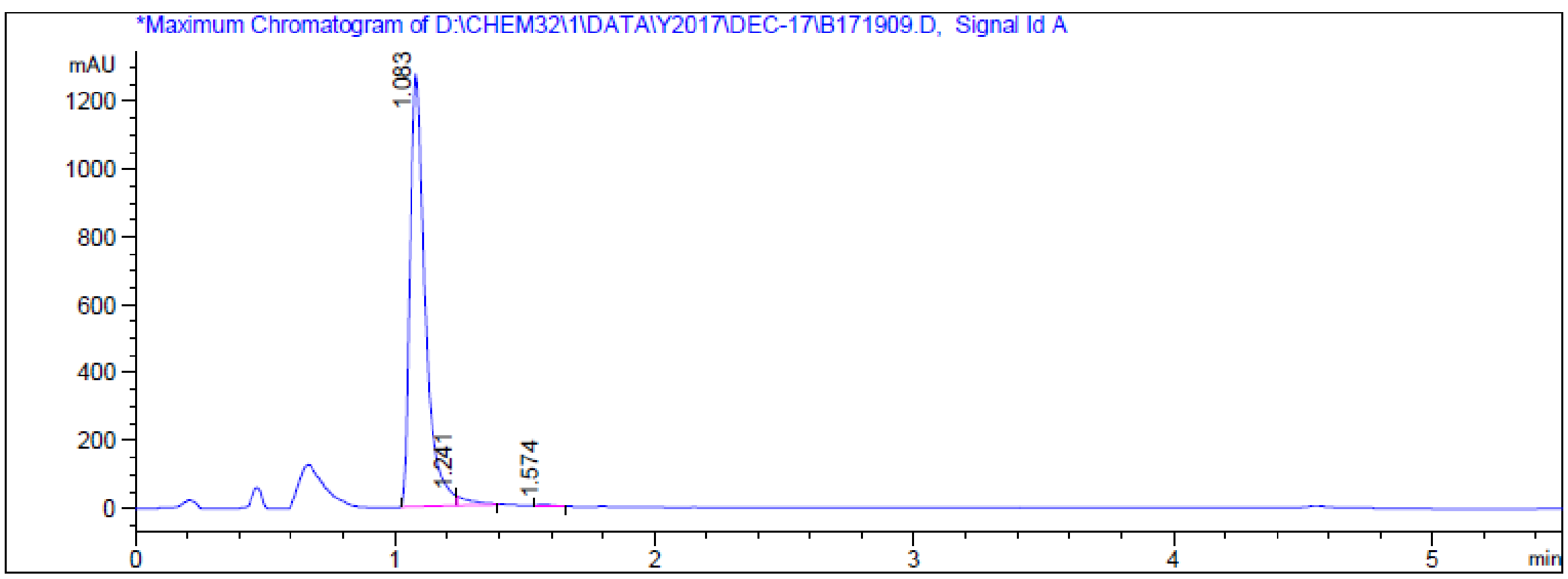

Peak No	RT min	Area	Area %
1	1.083	5.160e+003	97.524
2	1.241	1.053e+002	1.989
3	1.574	2.575e+001	0.487

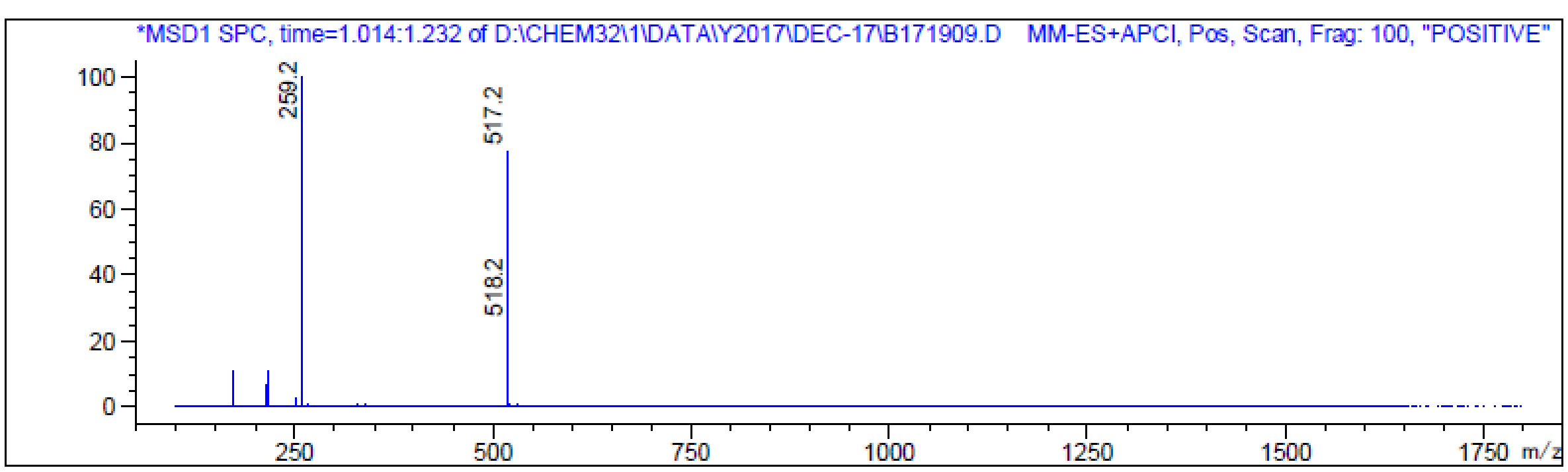

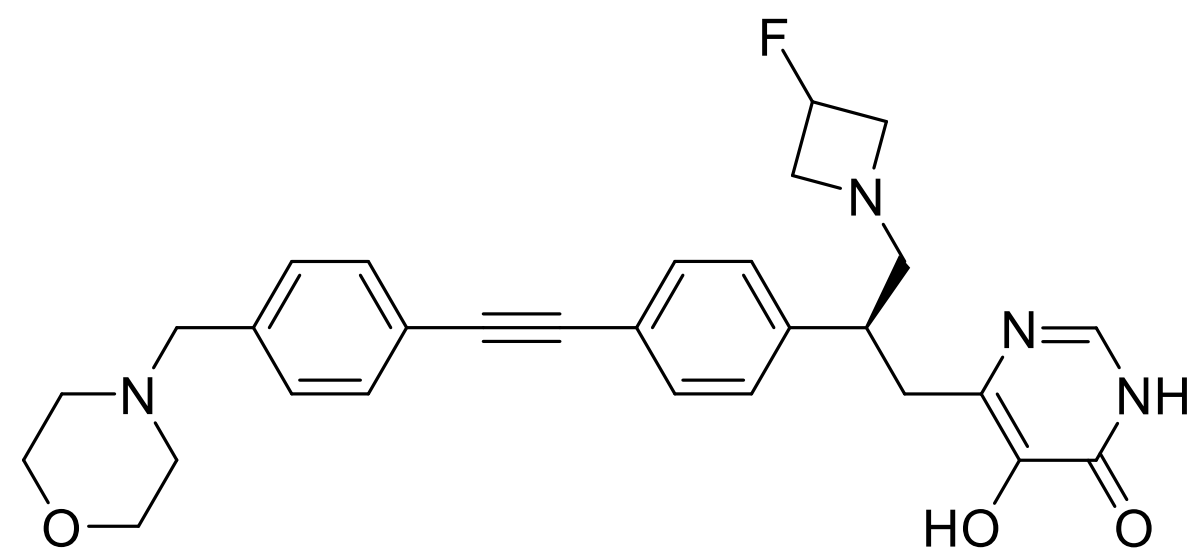

8q

```
Method info  :   Column :Atlantis dC18 (50*4.6)5µ
                 Mobile phase :A :0.1%Formic Acid in H2O B: ACN
                 Flow Rate :1.5 ml/min
                 Time (min)    %B
                 0.0            05
                 2.5            95
                 4.0            95
                 4.5            05
                 6.0            05
```

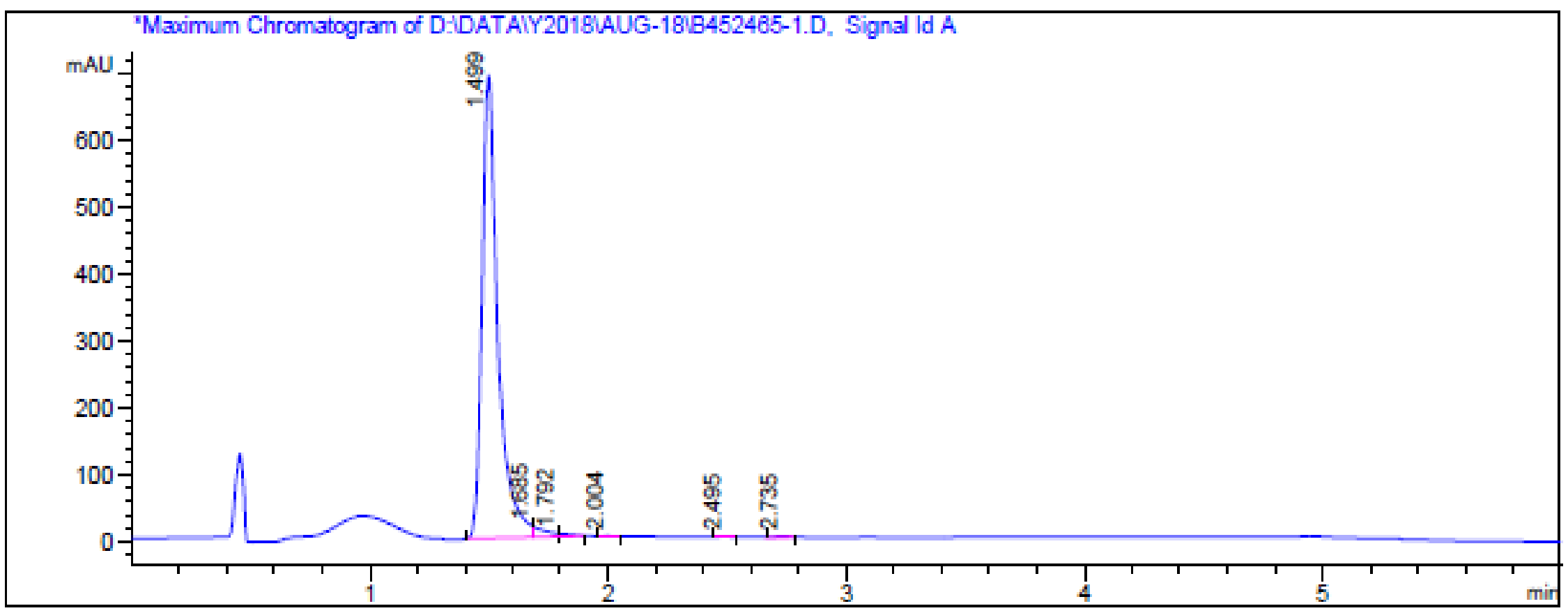

Peak No	RT min	Area	Area %
1	1.499	3198.522	97.331
2	1.685	57.660	1.755
3	1.792	16.617	0.506
4	2.004	2.837	0.086
5	2.495	3.077	0.094
6	2.735	7.512	0.229

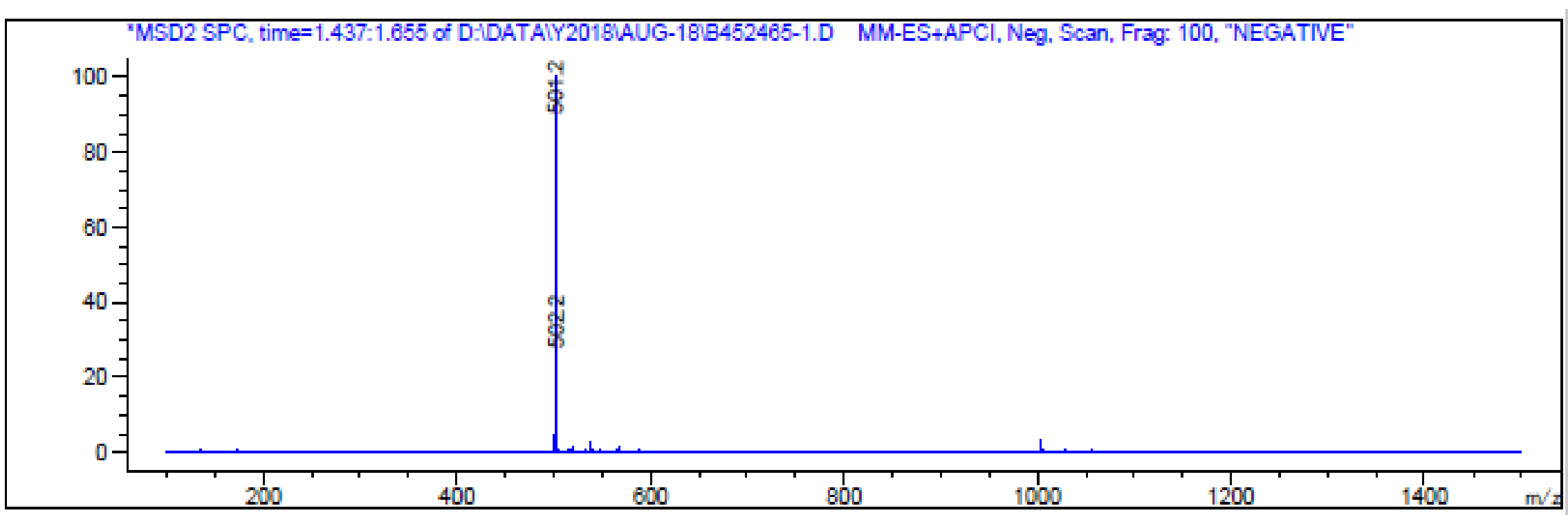

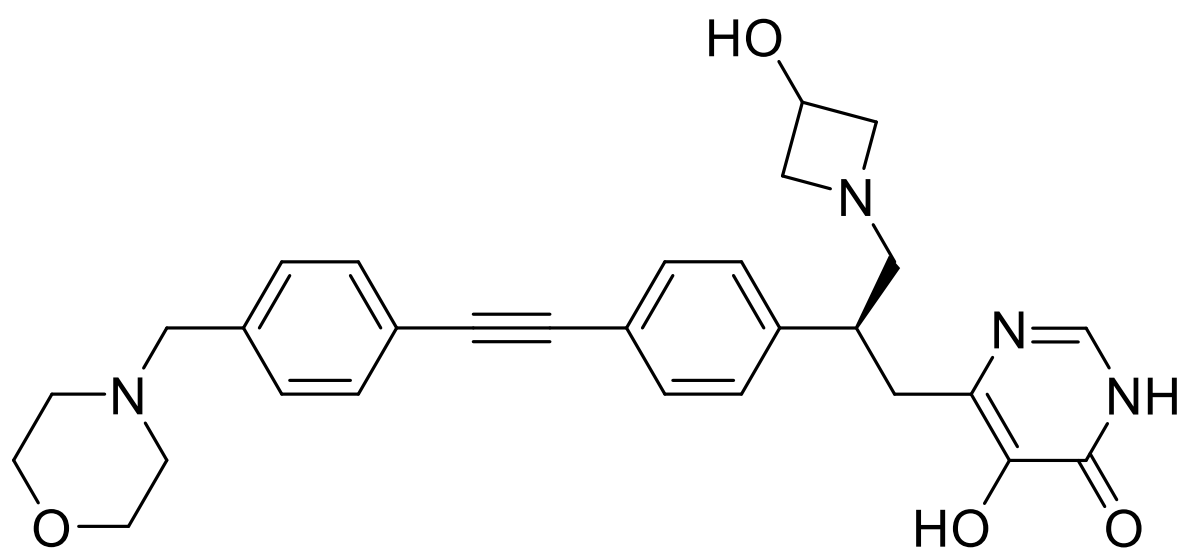

8r

```
Method info  :   Column :Atlantis dC18  (50*4.6)5µ
                 Mobile phase :A :0.1%Formic Acid in H2O B: ACN
                 Flow Rate :1.5 ml/min
                 Time  (min)      %B
                 0.0              05
                 2.5              95
                 4.0              95
                 4.5              05
                 6.0              05
```

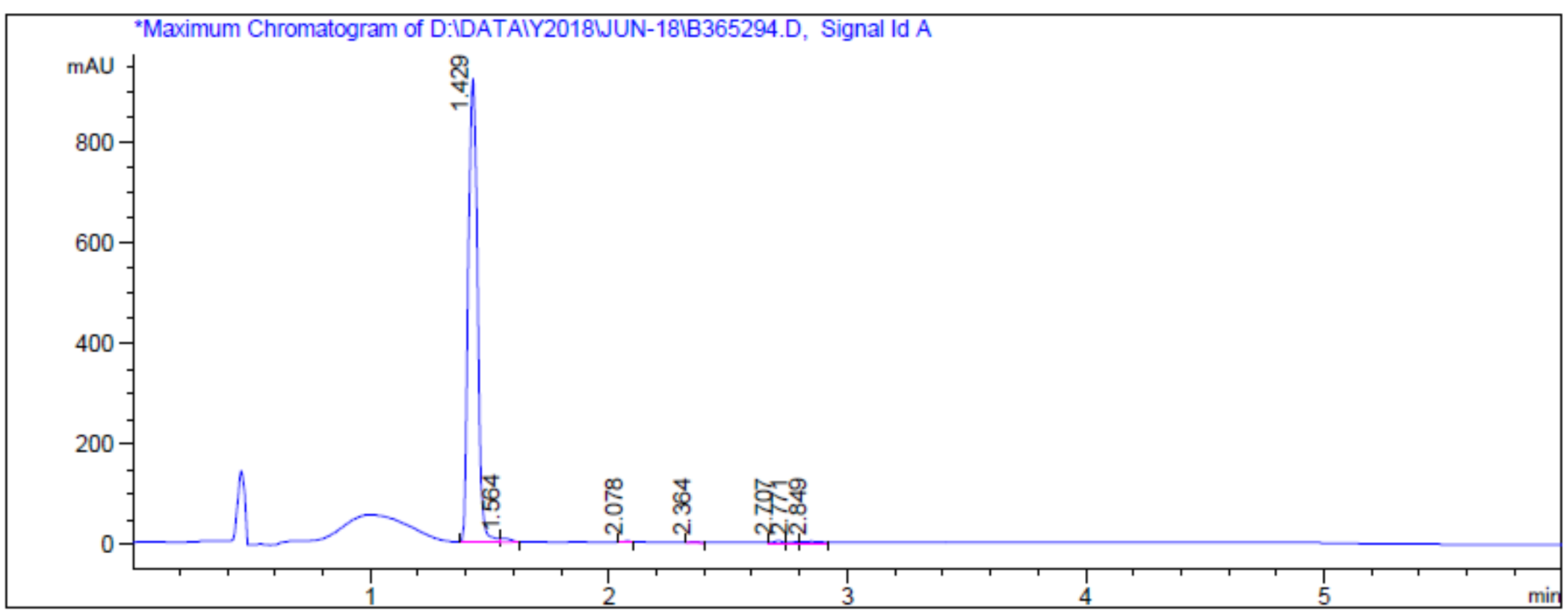

Peak No	RT min	Area	Area %
1	1.429	2487.904	97.350
2	1.564	22.804	0.892
3	2.078	6.406	0.251
4	2.364	8.490	0.332
5	2.707	9.328	0.365
6	2.771	4.996	0.195
7	2.849	15.697	0.614

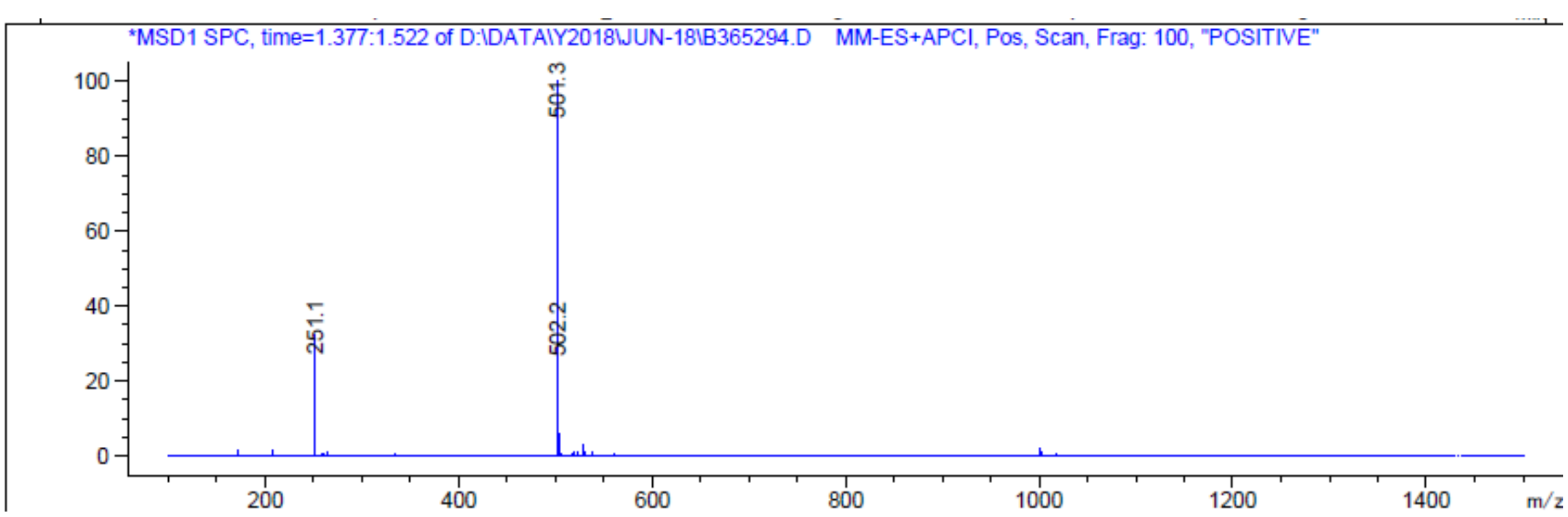

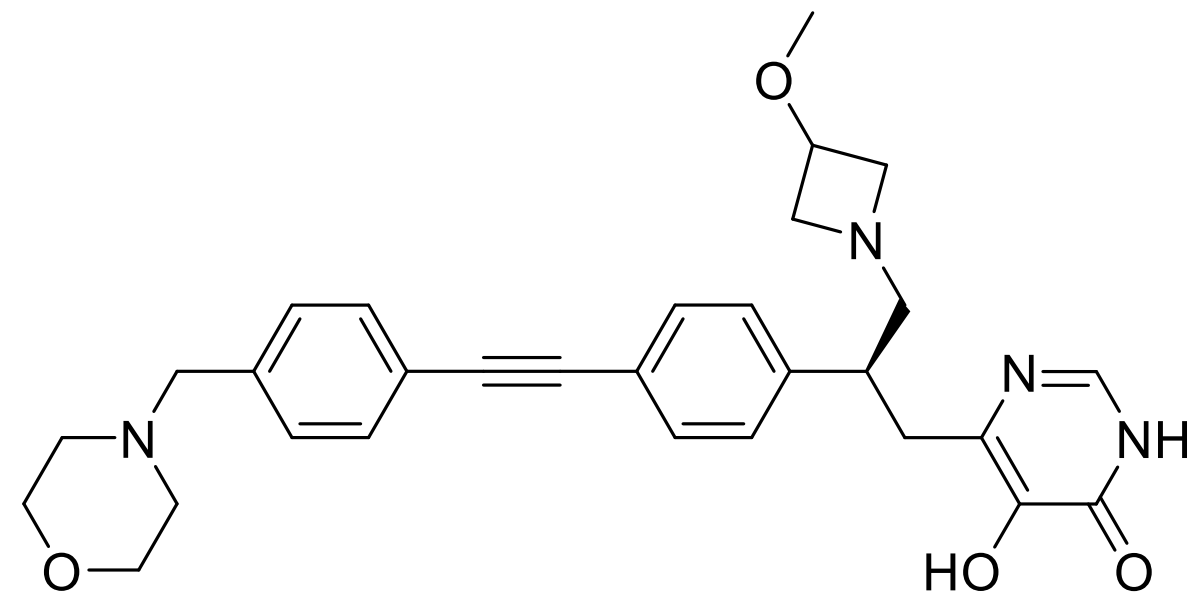

8s

```
Method info  :   Column :Atlantis dC18 (50*4.6)5µ
                 Mobile phase :A :0.1%Formic Acid in H2O B: ACN
                 Flow Rate :1.5 ml/min
                 Time (min)    %B
                 0.0              05
                 2.5              95
                 4.0              95
                 4.5              05
                 6.0              05
```

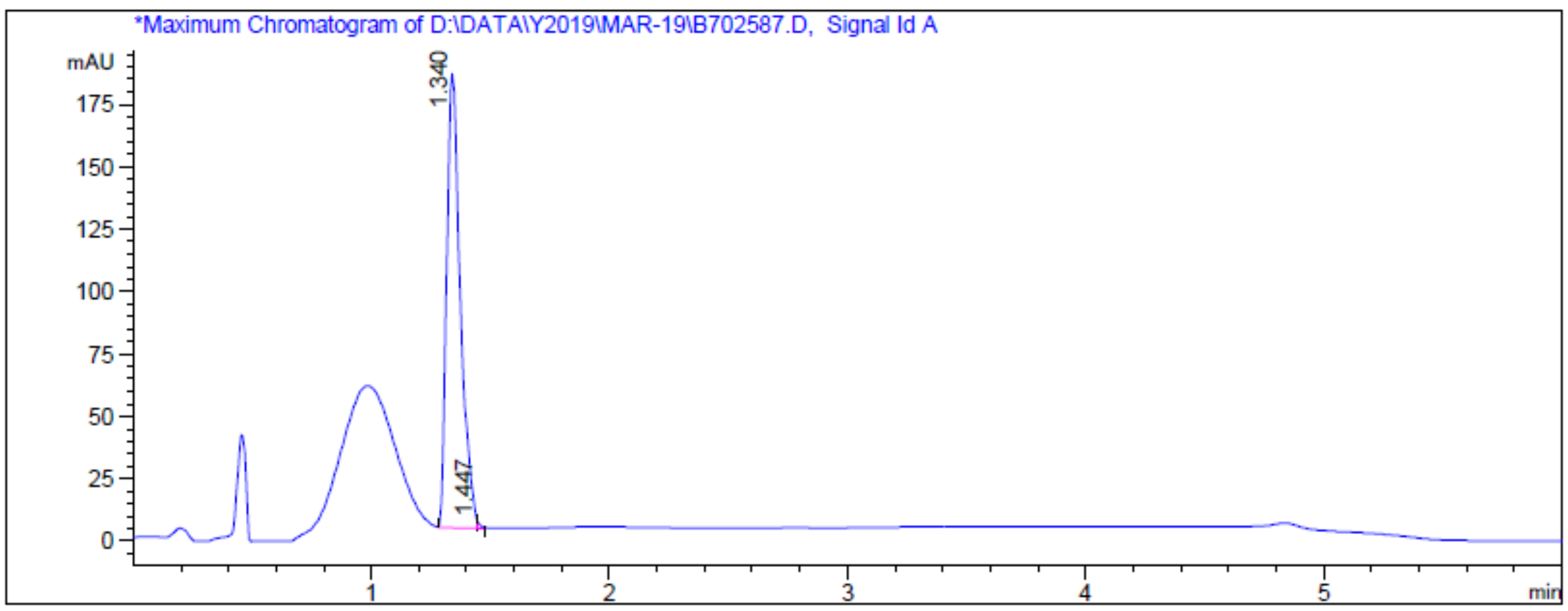

Peak No	RT min	Area	Area %
1	1.340	725.228	99.810
2	1.447	1.384	0.190

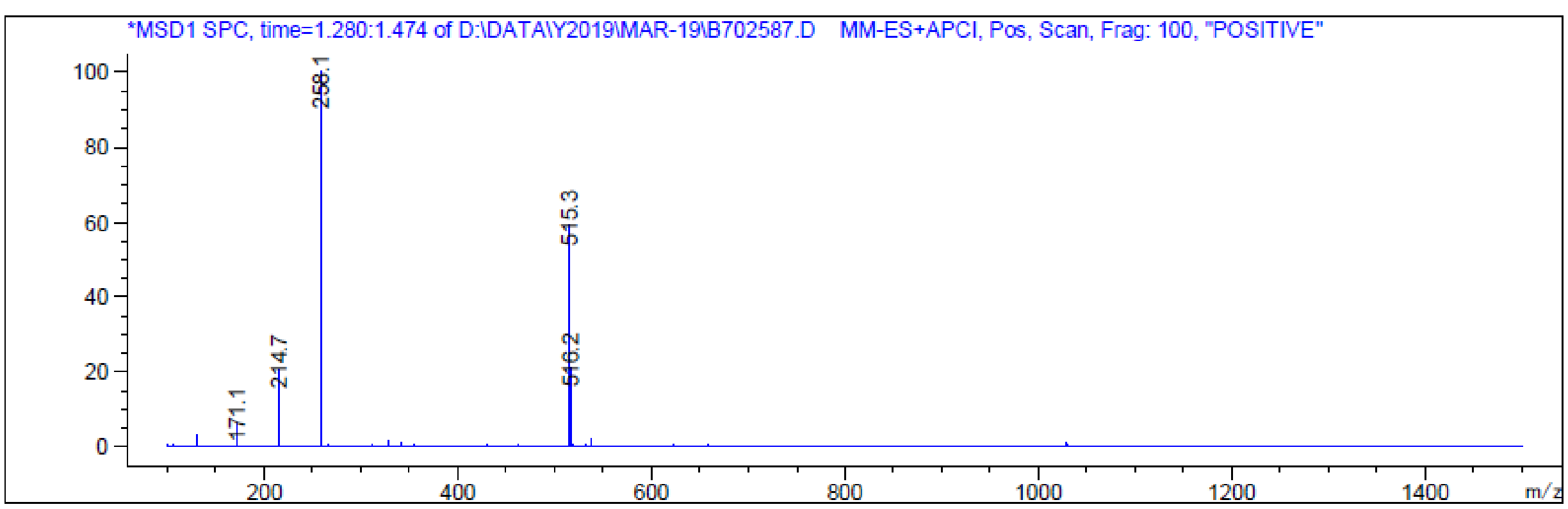

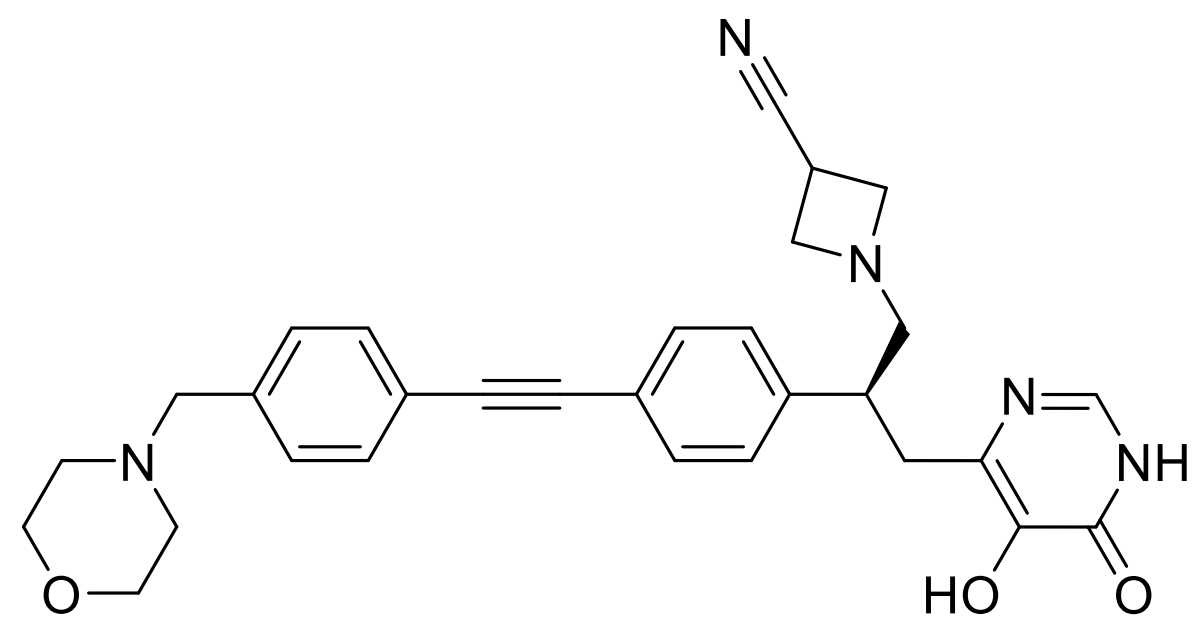

8t

```
Method info    :    Column :Atlantis dC18 (50*4.6)5µ
                    Mobile phase :A :0.1%Formic Acid in H2O B: ACN
                    Flow Rate :1.5 ml/min
                    Time (min)    %B
                    0.0           05
                    2.5           95
                    4.0           95
                    4.5           05
                    6.0           05
```

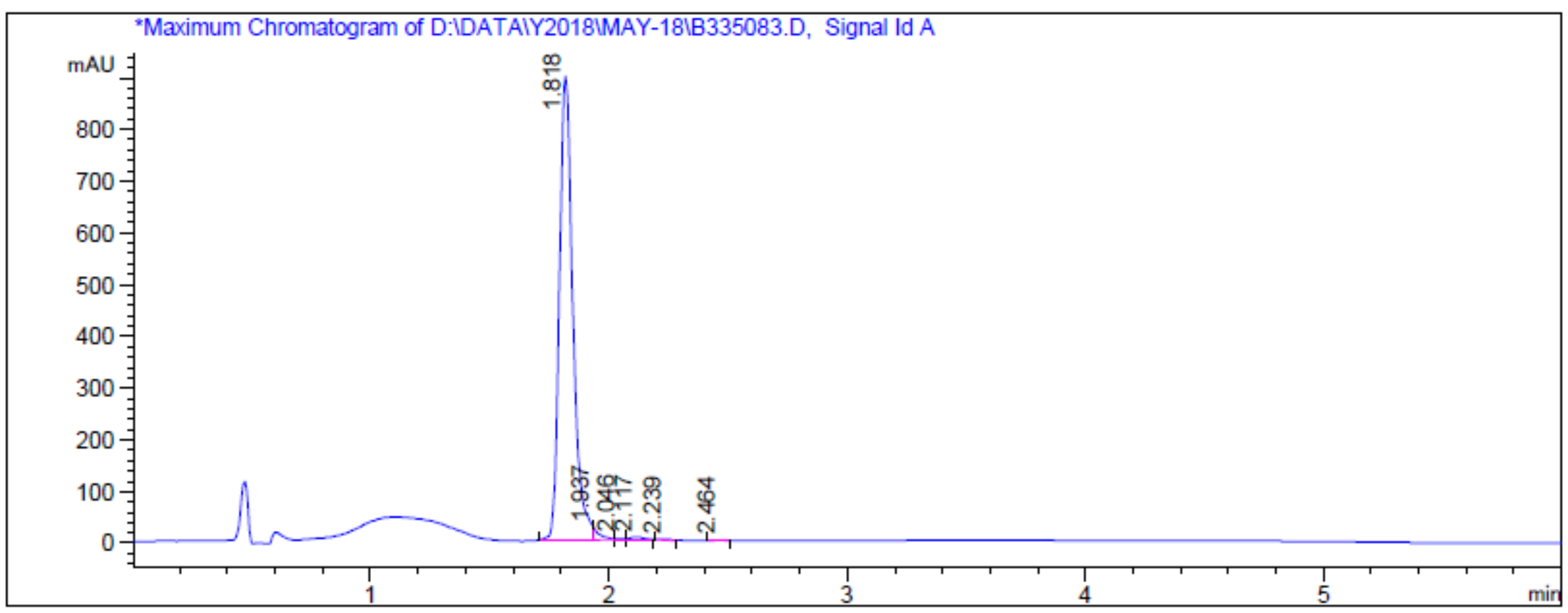

Peak No	RT min	Area	Area %
1	1.818	3515.933	97.868
2	1.937	35.344	0.984
3	2.046	6.769	0.188
4	2.117	21.782	0.606
5	2.239	7.478	0.208
6	2.464	5.226	0.145

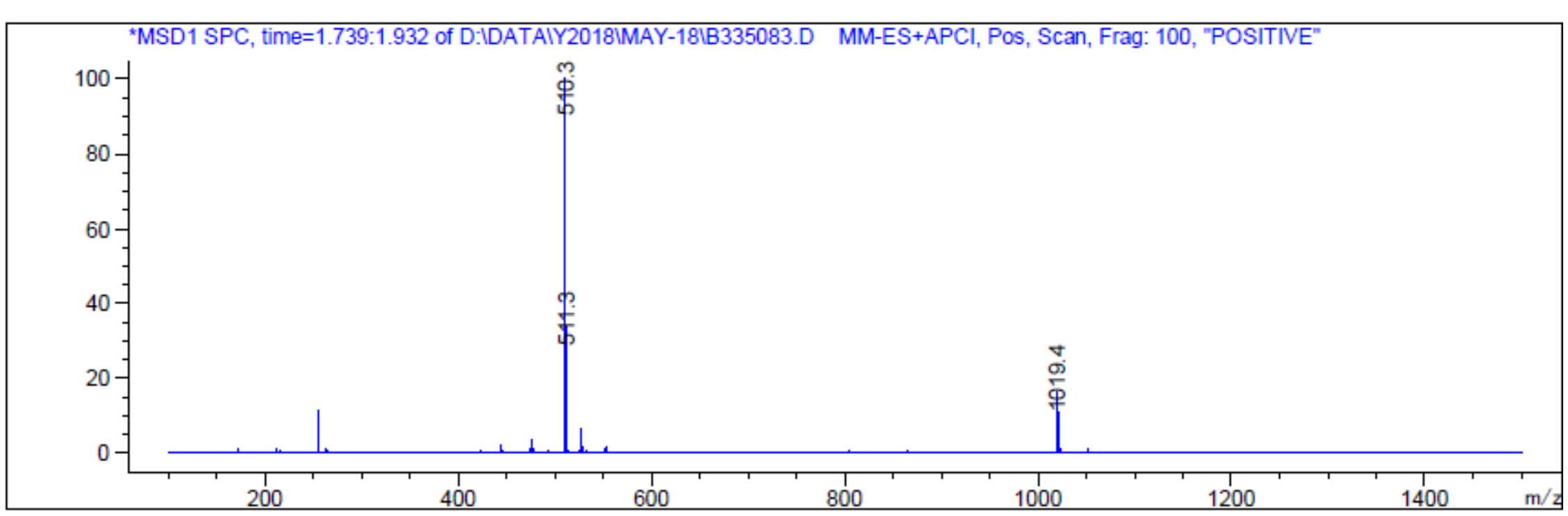

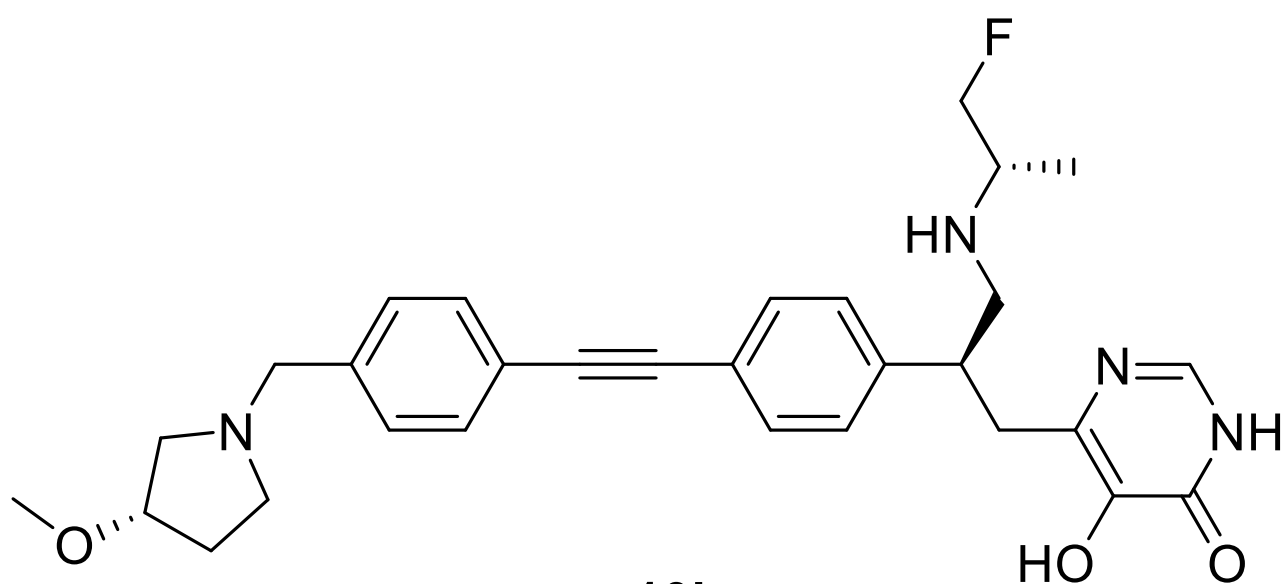

10k

```
Method info  :  Column :Atlantis dC18 (50*4.6)5
                Mobile phase :A :0.1%Formic Acid in H2O B: ACN
                Flow Rate :1.5 ml/min
                Time(min) %B
                0.0 05
                2.5 95
                4.0 95
                4.5 05
                6.0 05
```

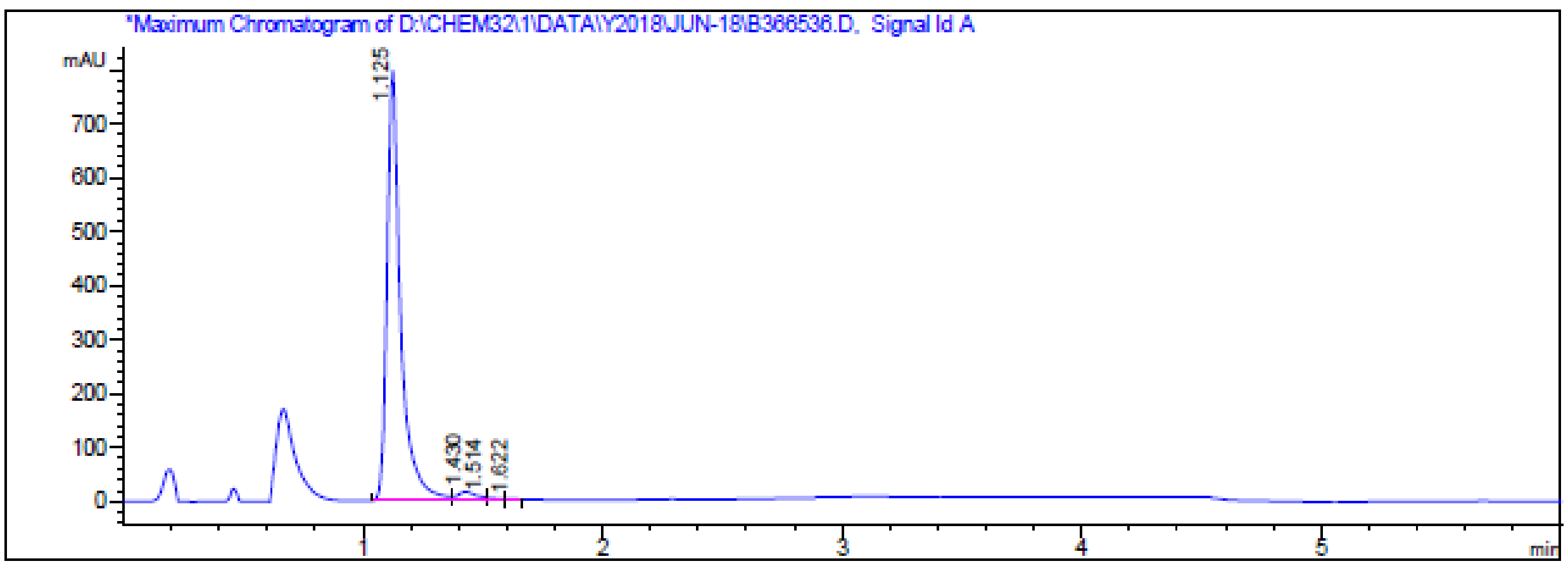

Peak No	RT min	Area	Area %
1	1.125	3220.082	97.143
2	1.430	78.083	2.356
3	1.514	10.837	0.327
4	1.622	5.788	0.175

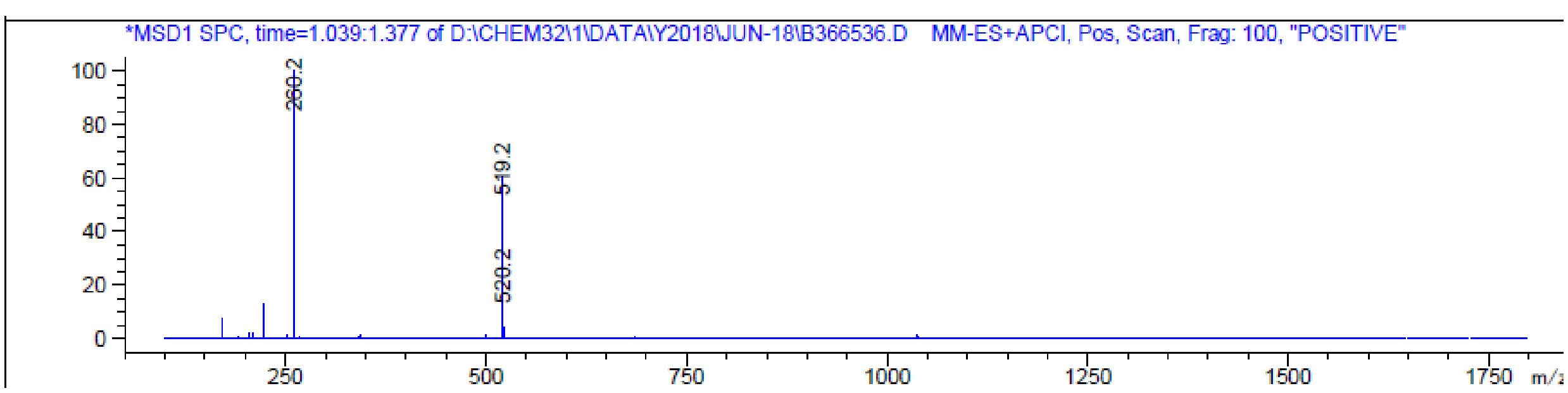

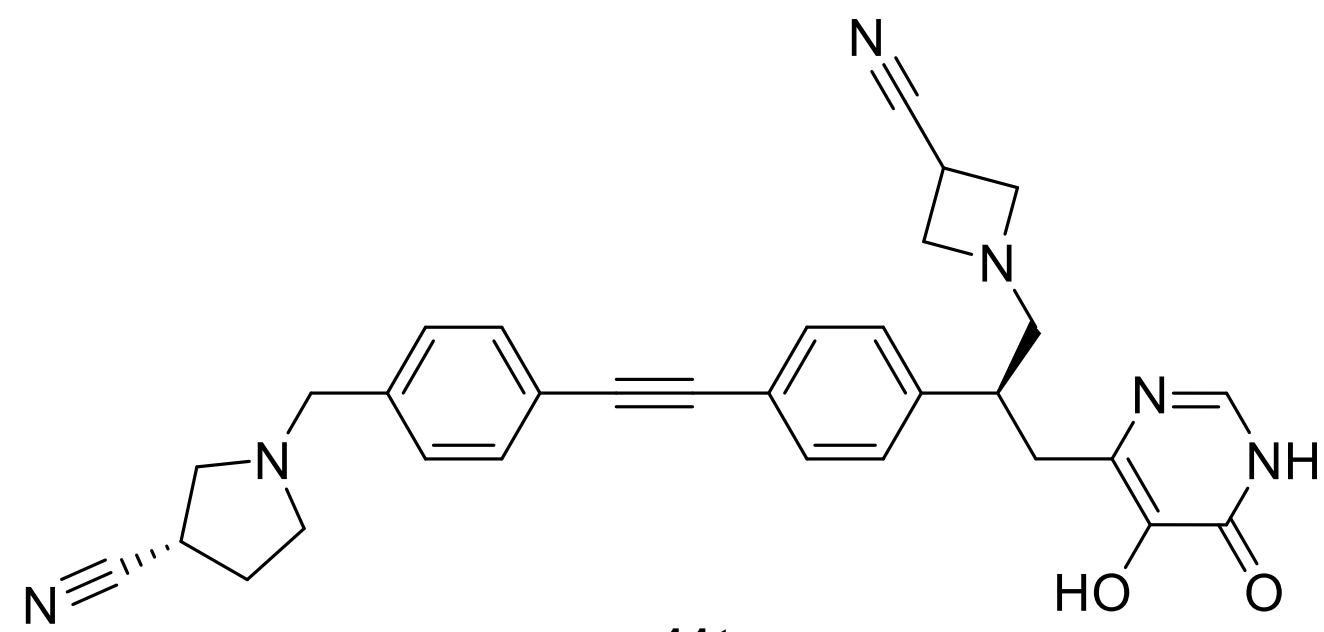

11t

```
Method info   :   Column :Atlantis dC18  (50*4.6)5µ
                  Mobile phase :A :0.1%Formic Acid in H2O B: ACN
                  Flow Rate :1.5 ml/min
                  Time  (min)    %B
                  0.0              05
                  2.5              95
                  4.0              95
                  4.5              05
                  6.0              05
```

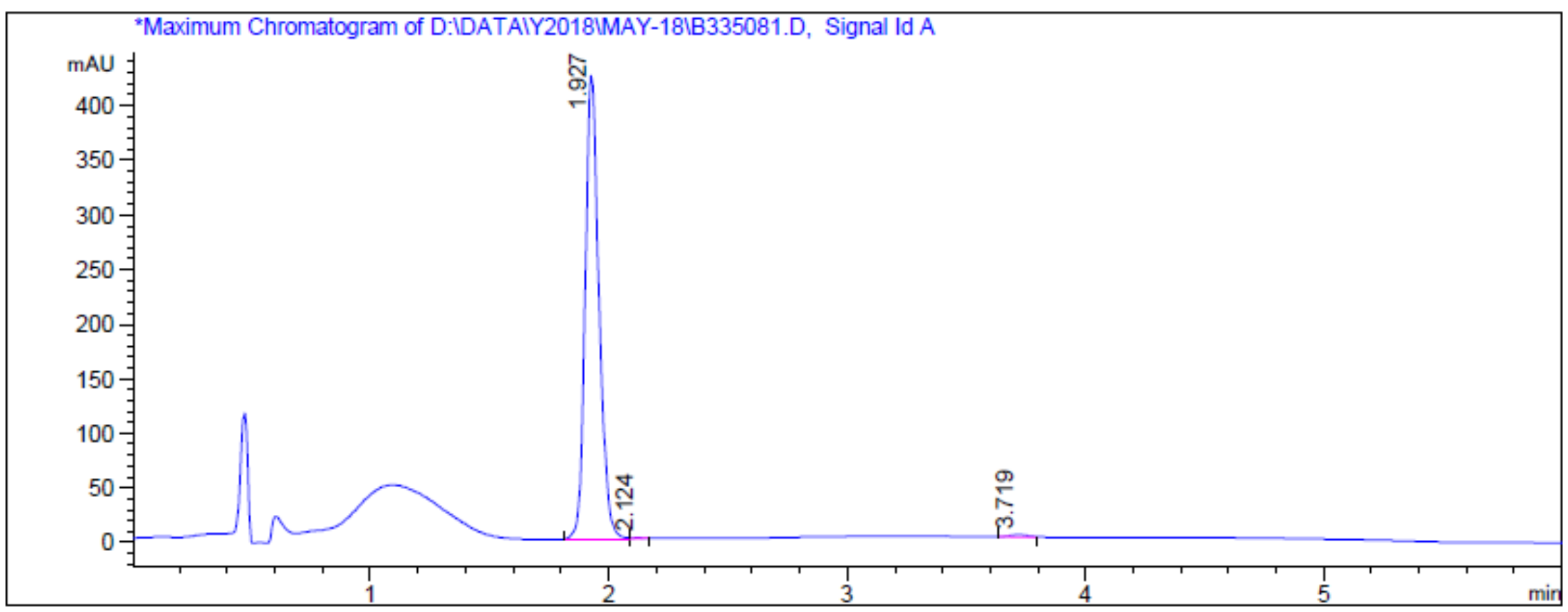

Peak No	RT min	Area	Area %
1	1.927	1708.748	99.117
2	2.124	2.284	0.133
3	3.719	12.946	0.751

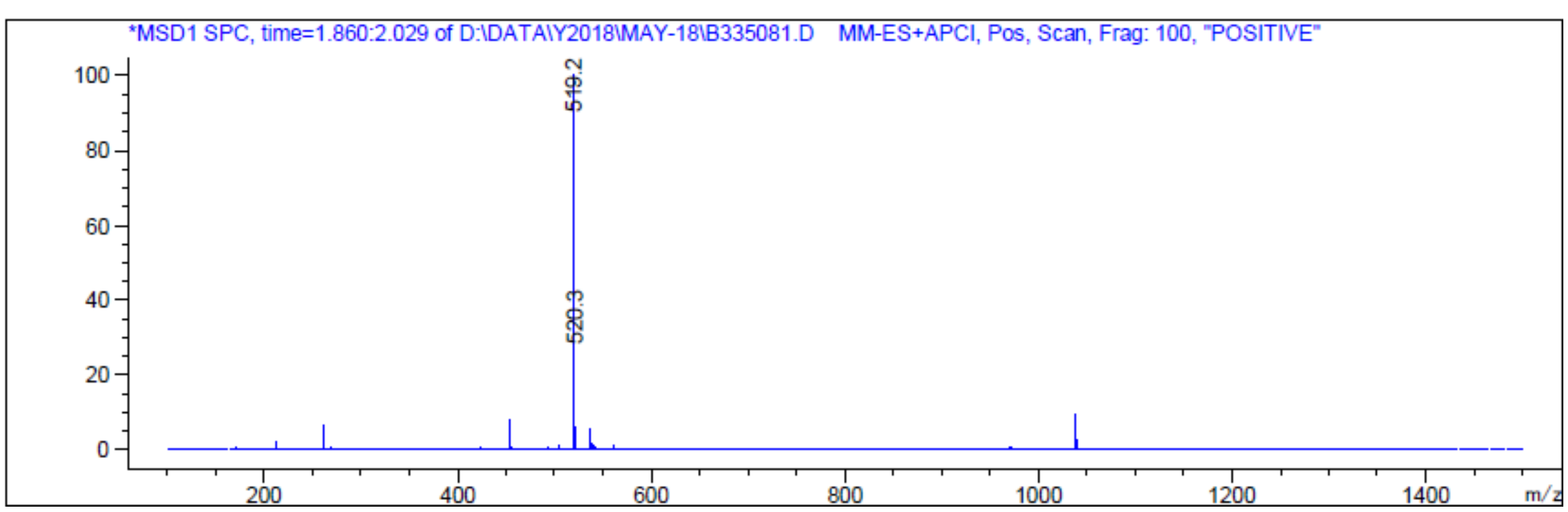

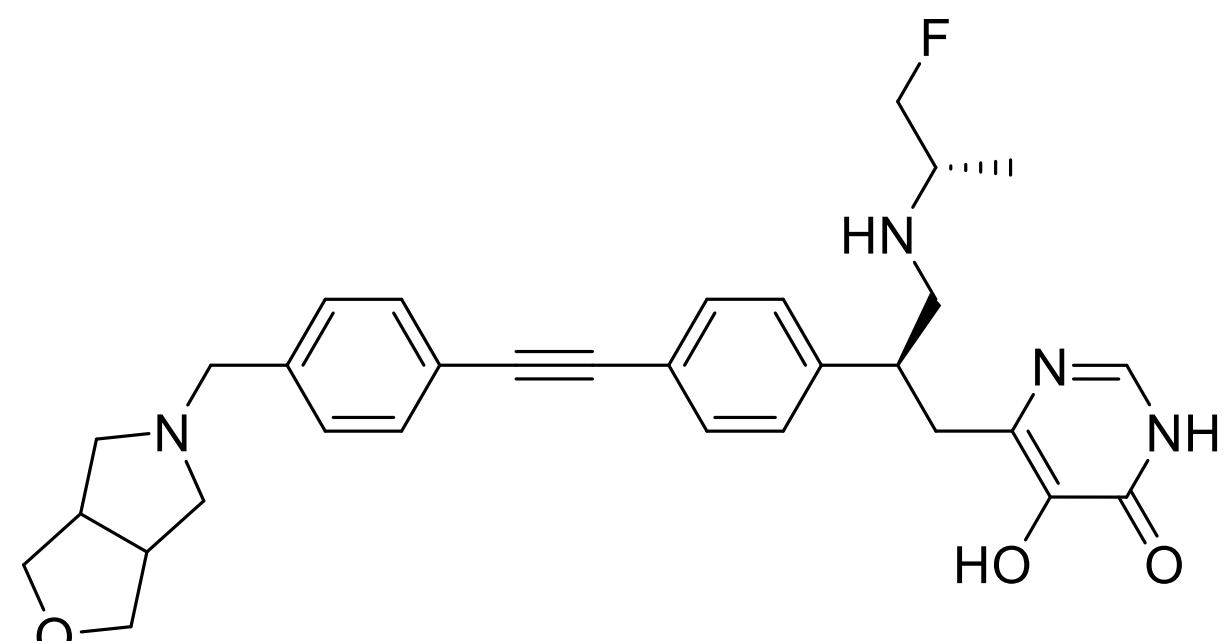

12k

```
Method info  :   Column :Atlantis dC18 (50*4.6)5µ
                 Mobile phase :A :0.1%Formic Acid in H2O B: ACN
                 Flow Rate :1.5 ml/min
                 Time (min)    %B
                 0.0              05
                 2.5              95
                 4.0              95
                 4.5              05
                 6.0              05
```

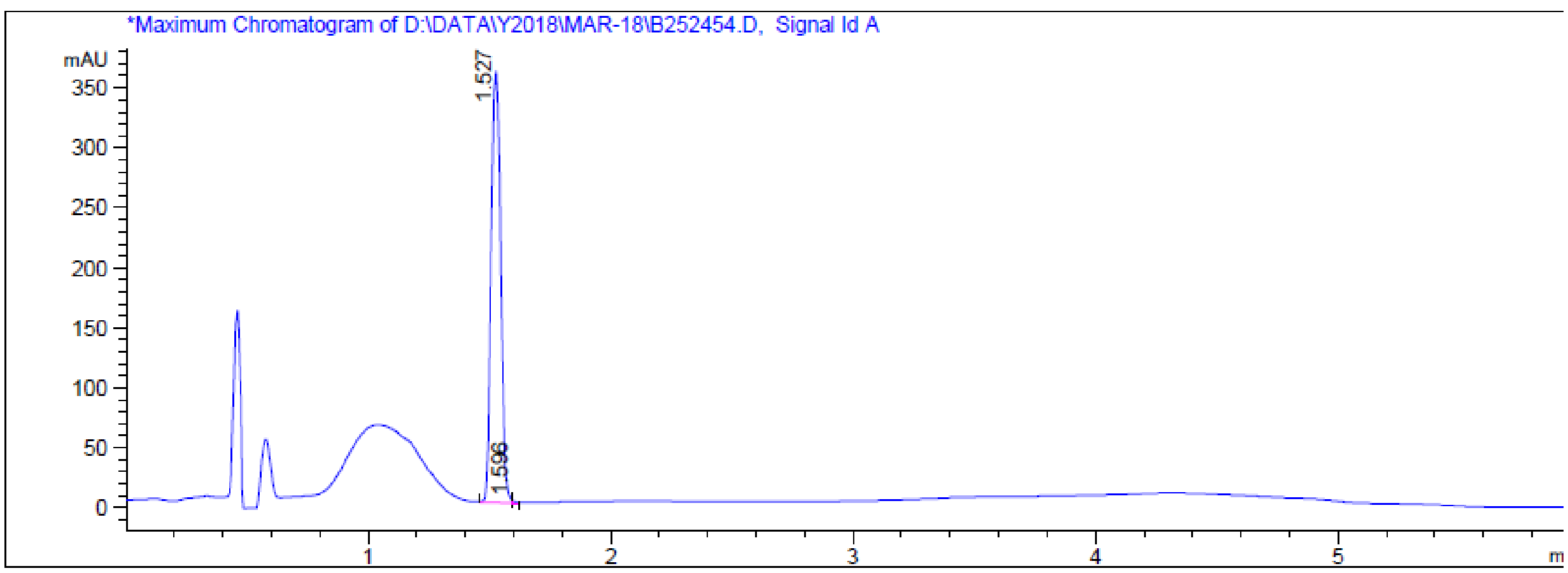

Peak No	RT min	Area	Area %
1	1.527	962.392	99.903
2	1.596	0.931	0.097

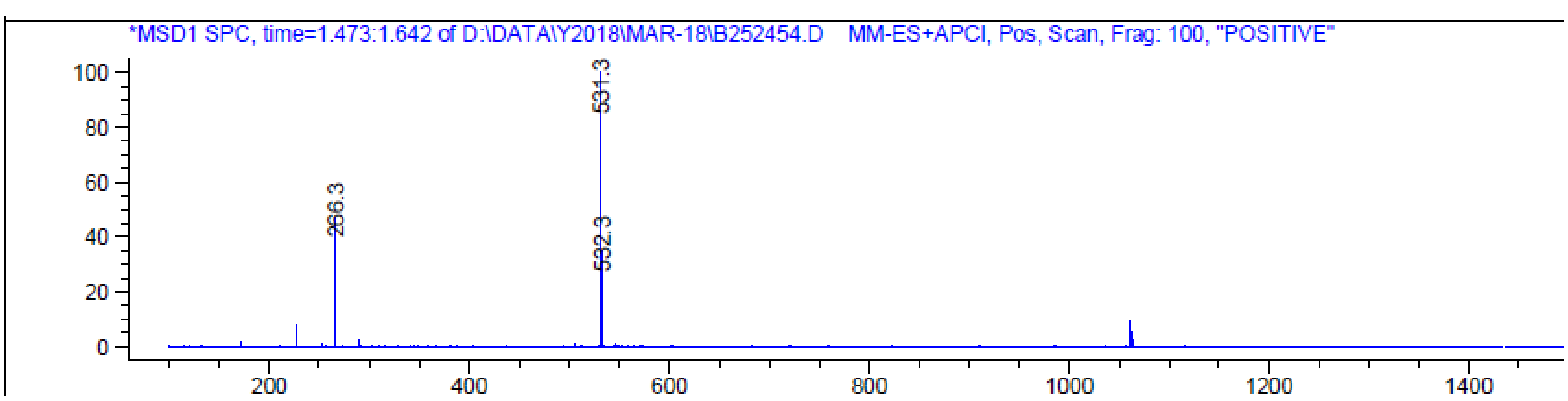

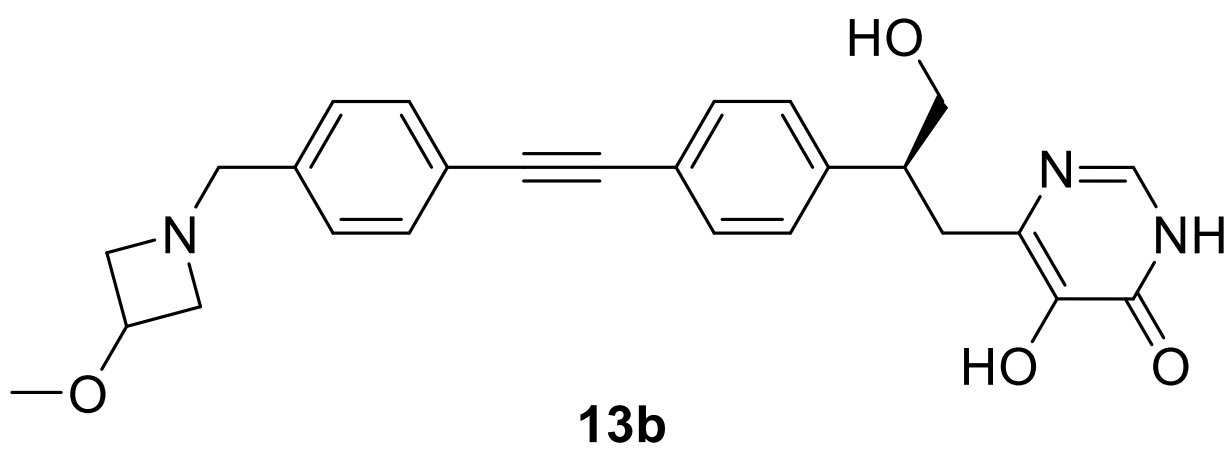

13b

```
Method info  :     Column :Atlantis dC18  (50*4.6)5µ
                   Mobile phase :A :0.1%Formic Acid in H2O B: ACN
                   Flow Rate :1.5 ml/min
                   Time(min)                    %B
                   0.0                               05
                   2.5                               95
                   4.0                               95
                   4.5                               05
                   6.0                               05
```

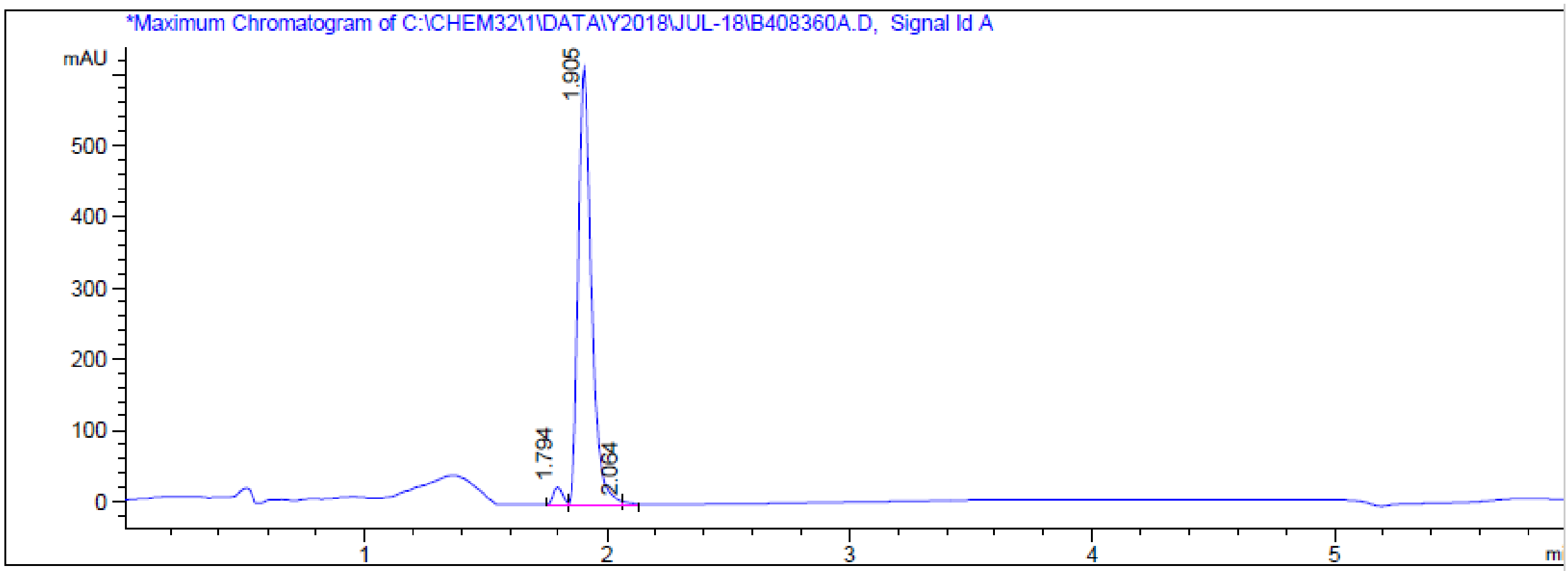

Peak No	RT min	Area	Area %
1	1.794	66.559	2.757
2	1.905	2340.936	96.982
3	2.064	6.278	0.260

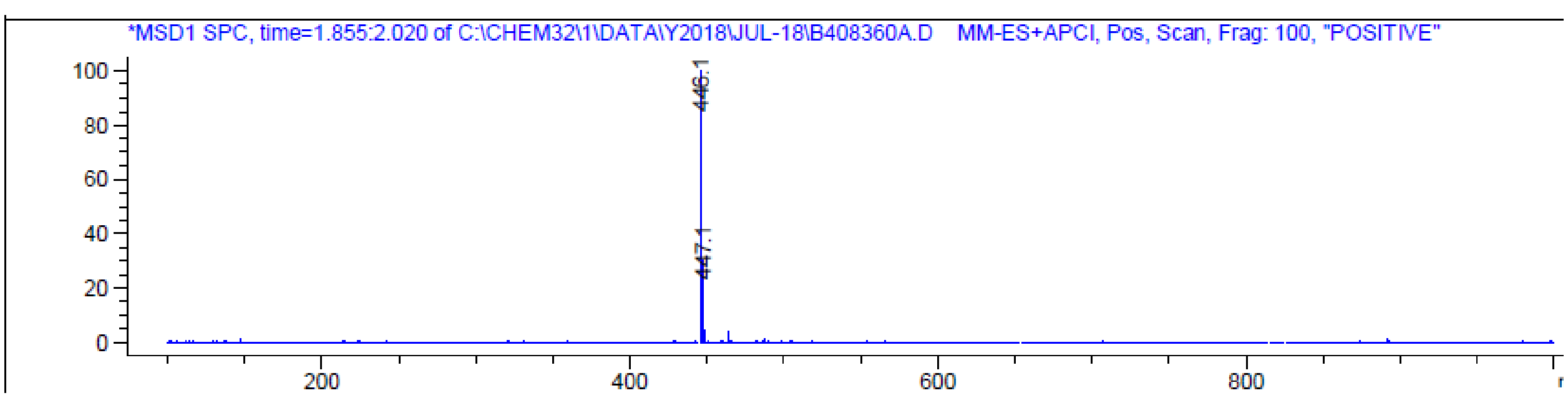

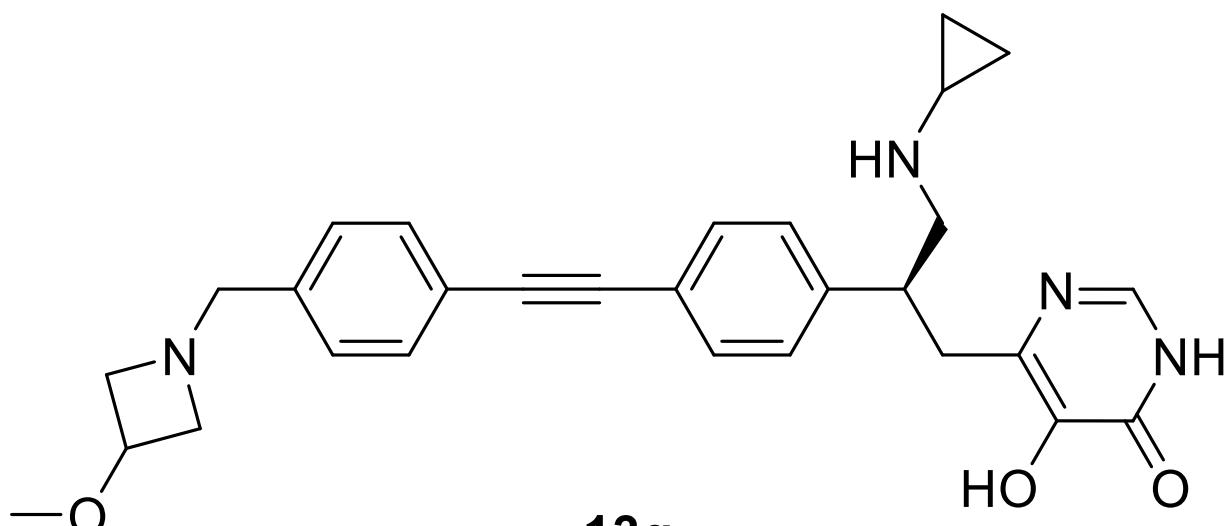

13g

```
Method info  :   Column :Atlantis dC18  (50*4.6)5µ
                 Mobile phase :A :0.1%Formic Acid in H2O B: ACN
                 Flow Rate :1.5 ml/min
                 Time  (min)    %B
                 0.0              05
                 2.5              95
                 4.0              95
                 4.5              05
                 6.0              05
```

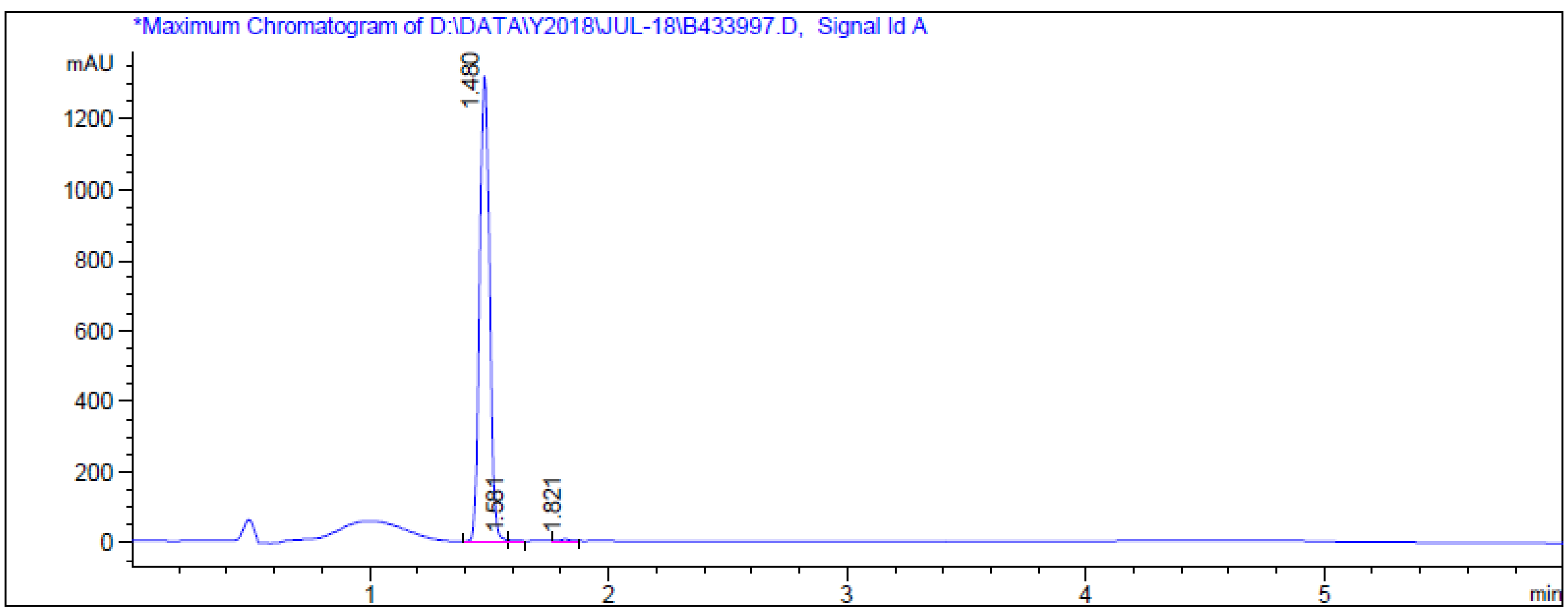

Peak No	RT min	Area	Area %
1	1.480	3815.411	99.416
2	1.581	5.765	0.150
3	1.821	16.665	0.434

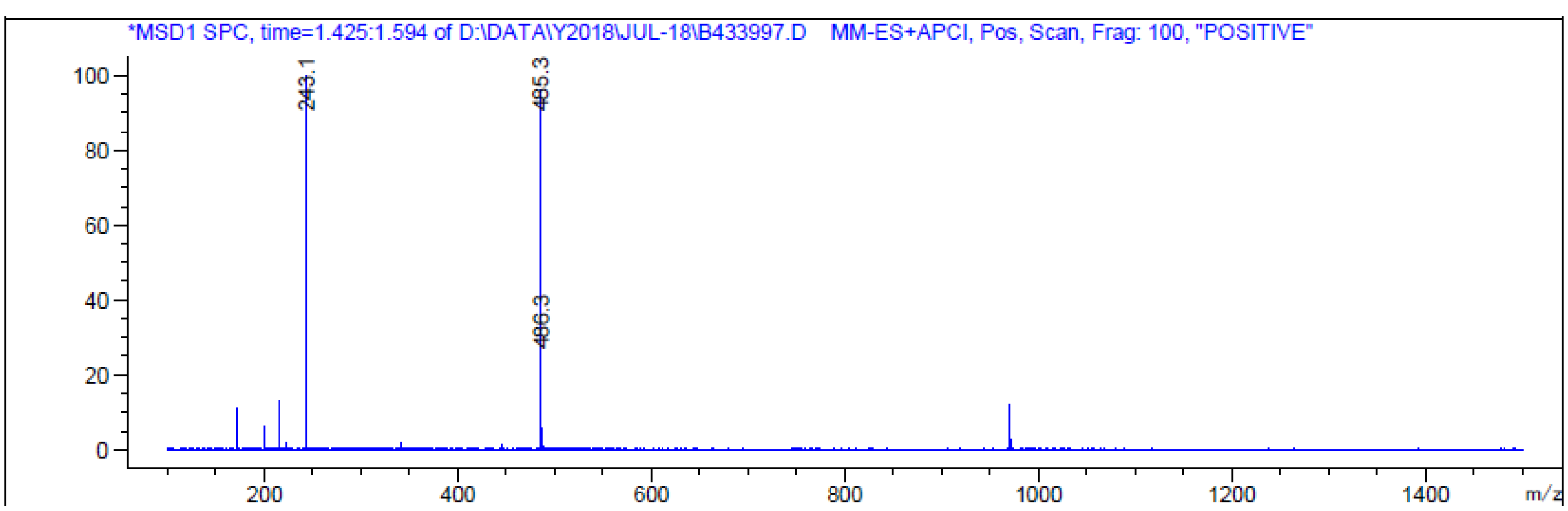

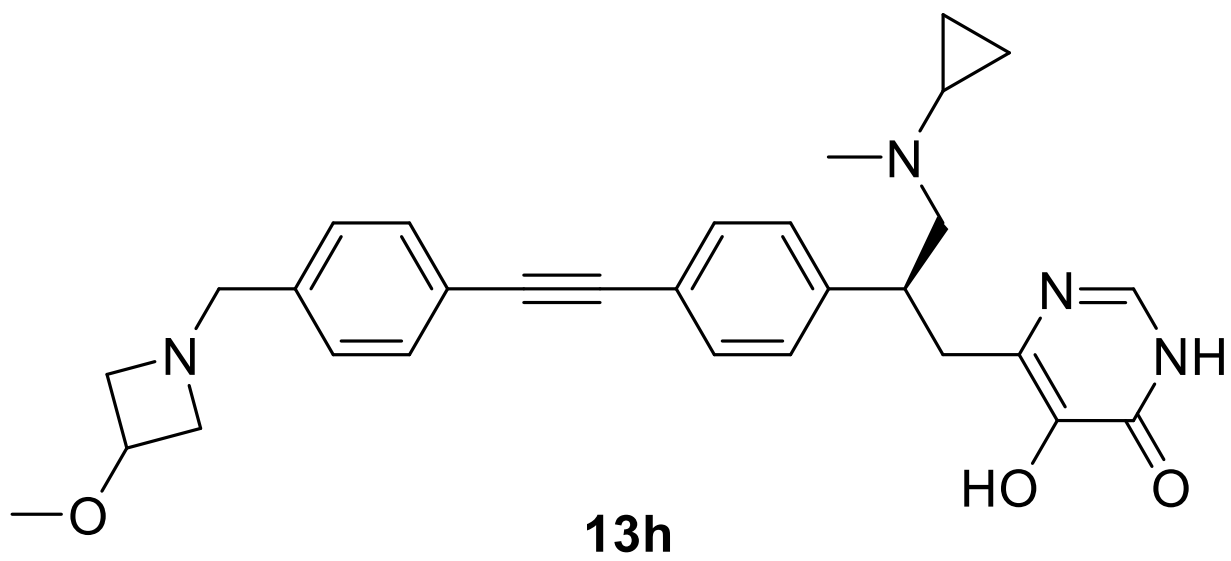

13h

```
Method info  :   Column :Atlantis dC18 (50*4.6)5µ
                 Mobile phase :A :0.1%Formic Acid in H2O B: ACN
                 Flow Rate :1.5 ml/min
                 Time (min)    %B
                 0.0              05
                 2.5              95
                 4.0              95
                 4.5              05
                 6.0              05
```

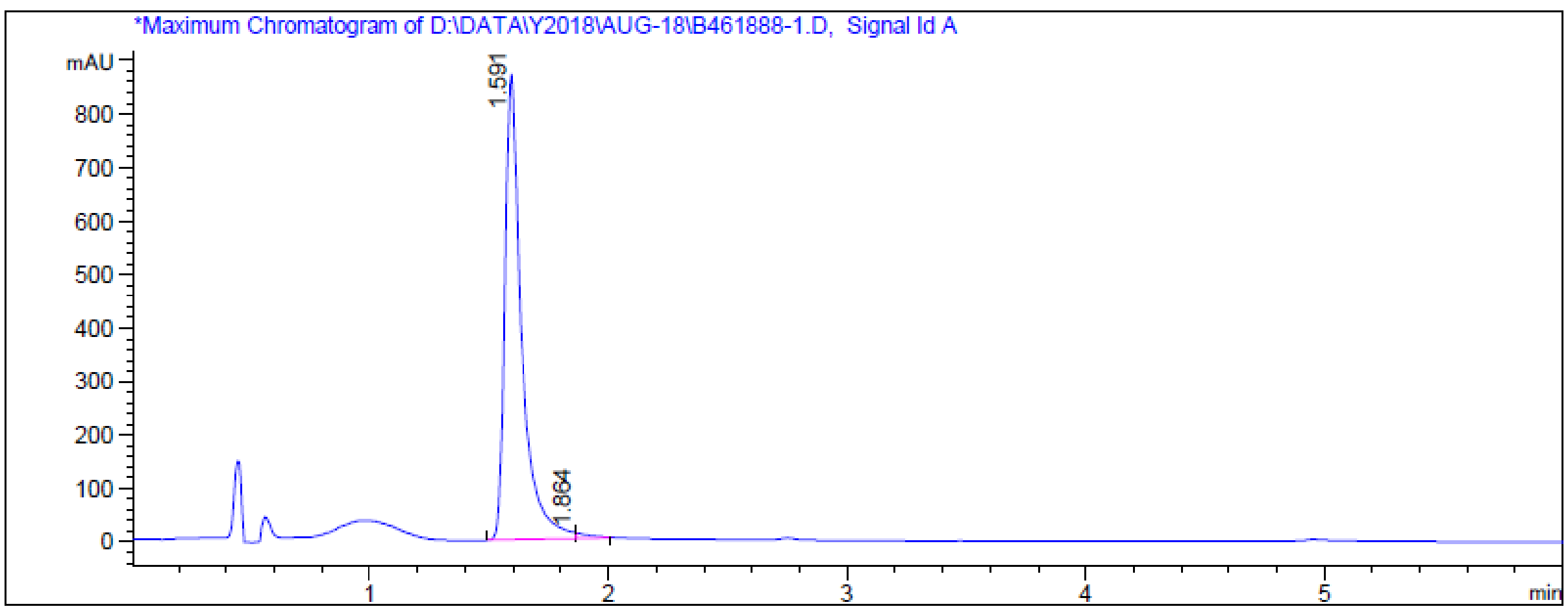

Peak No	RT min	Area	Area %
1	1.591	4298.686	99.189
2	1.864	35.161	0.811

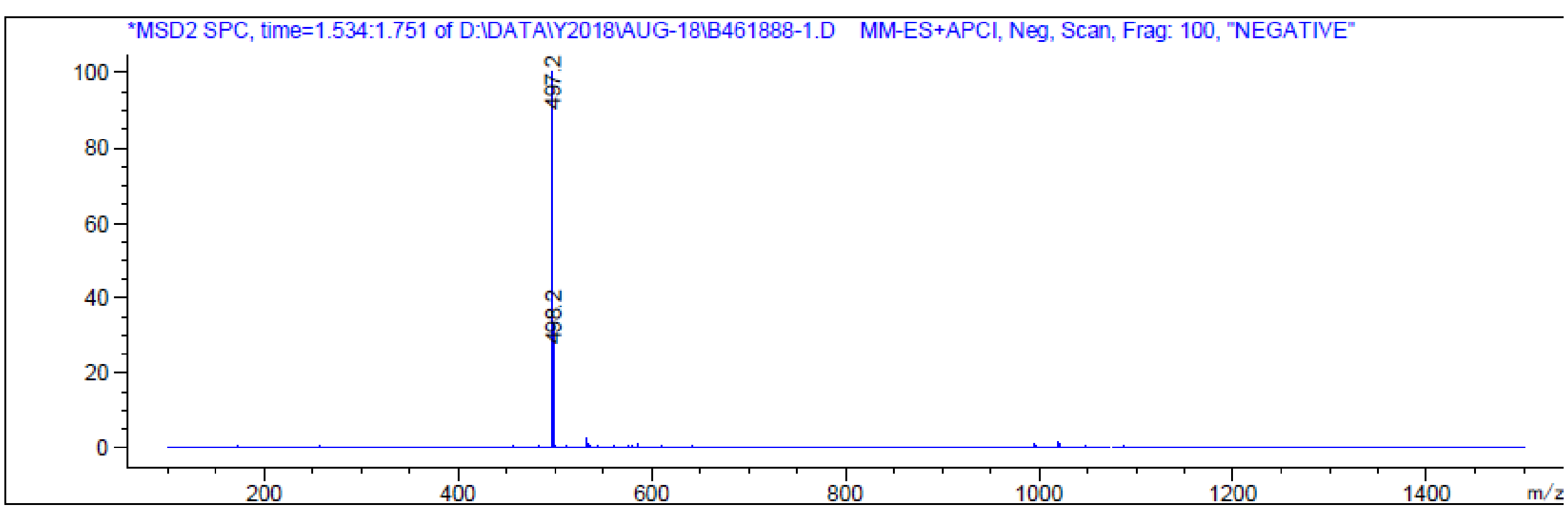

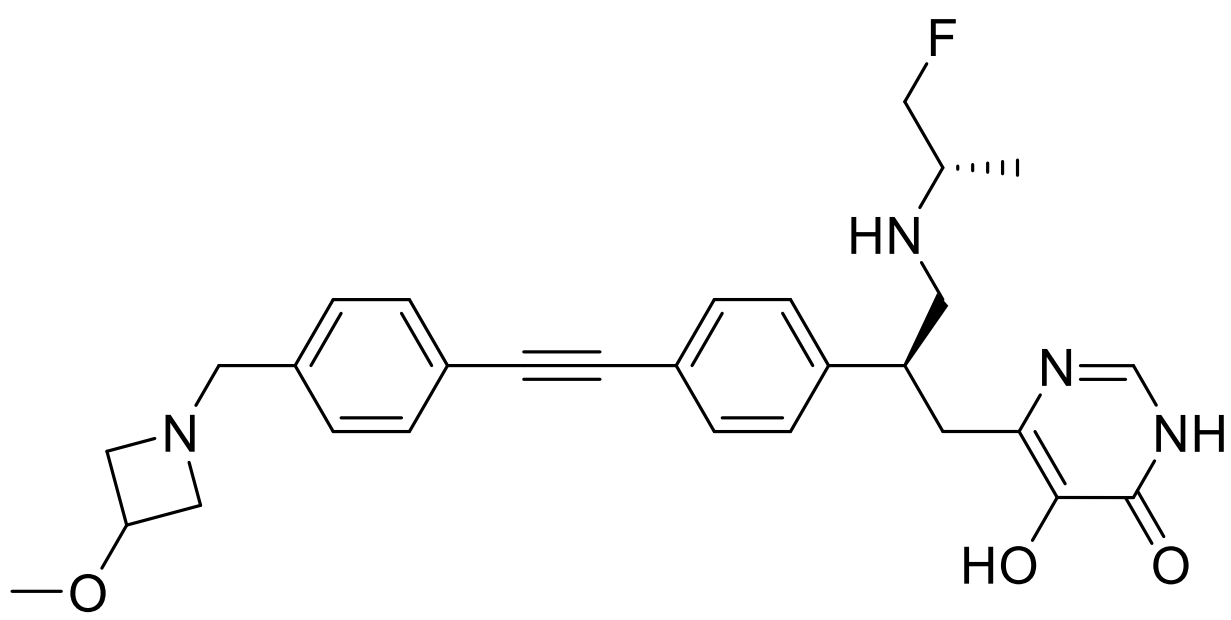

13k

```
Method info  :   Column :Atlantis dC18  (50*4.6)5µ
                 Mobile phase :A :0.1%Formic Acid in H2O B: ACN
                 Flow Rate :1.5 ml/min
                 Time  (min)    %B
                 0.0             05
                 2.5             95
                 4.0             95
                 4.5             05
                 6.0             05
```

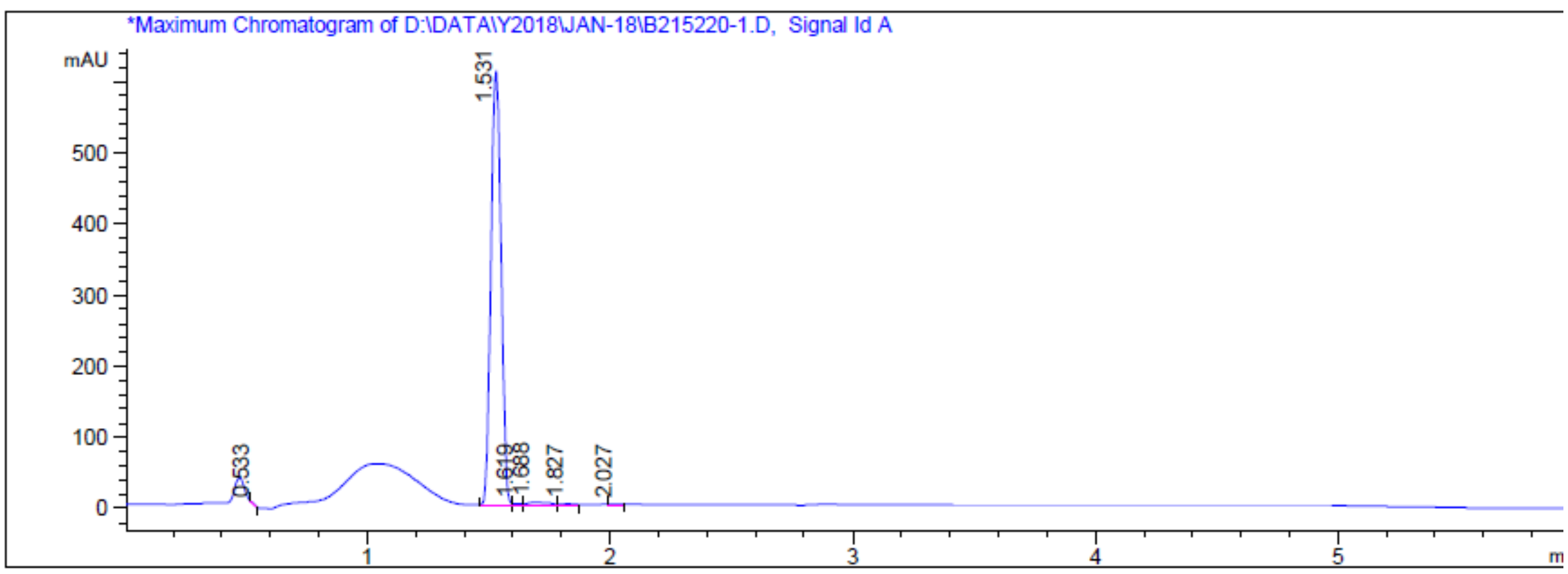

Peak No	RT min	Area	Area %
1	0.533	1.726	0.095
2	1.531	1771.066	97.917
3	1.619	4.258	0.235
4	1.688	23.930	1.323
5	1.827	5.577	0.308
6	2.027	2.179	0.120

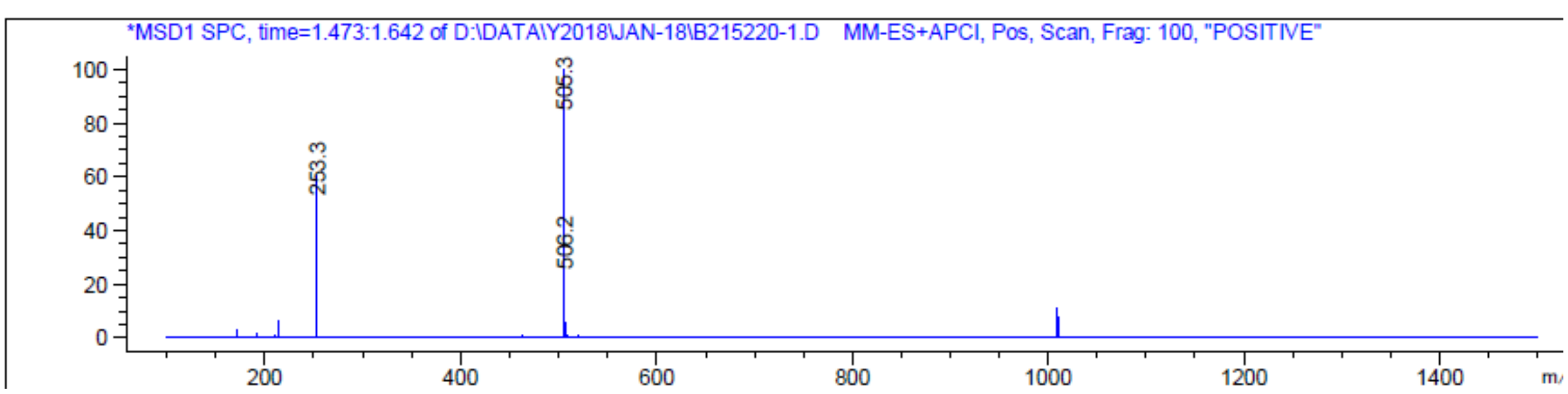

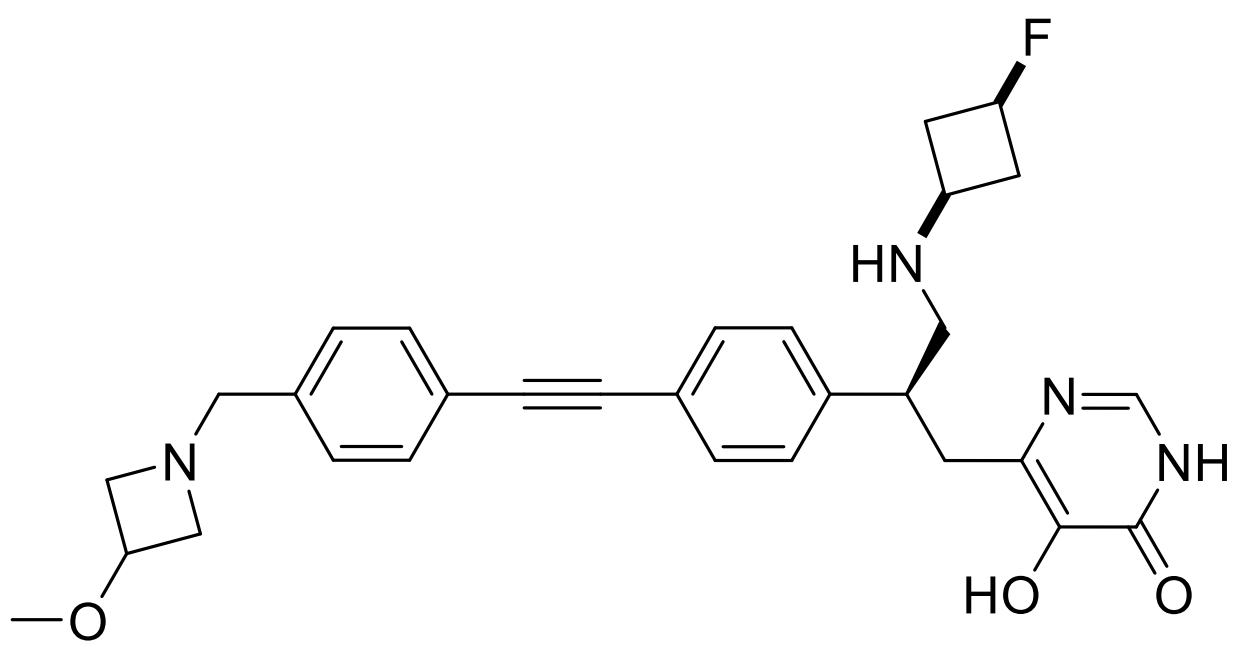

13p

```
Method info  :   Column :Atlantis dC18 (50*4.6)5µ
                 Mobile phase :A :0.1%Formic Acid in H2O B: ACN
                 Flow Rate :1.5 ml/min
                 Time (min)    %B
                 0.0              05
                 2.5              95
                 4.0              95
                 4.5              05
                 6.0              05
```

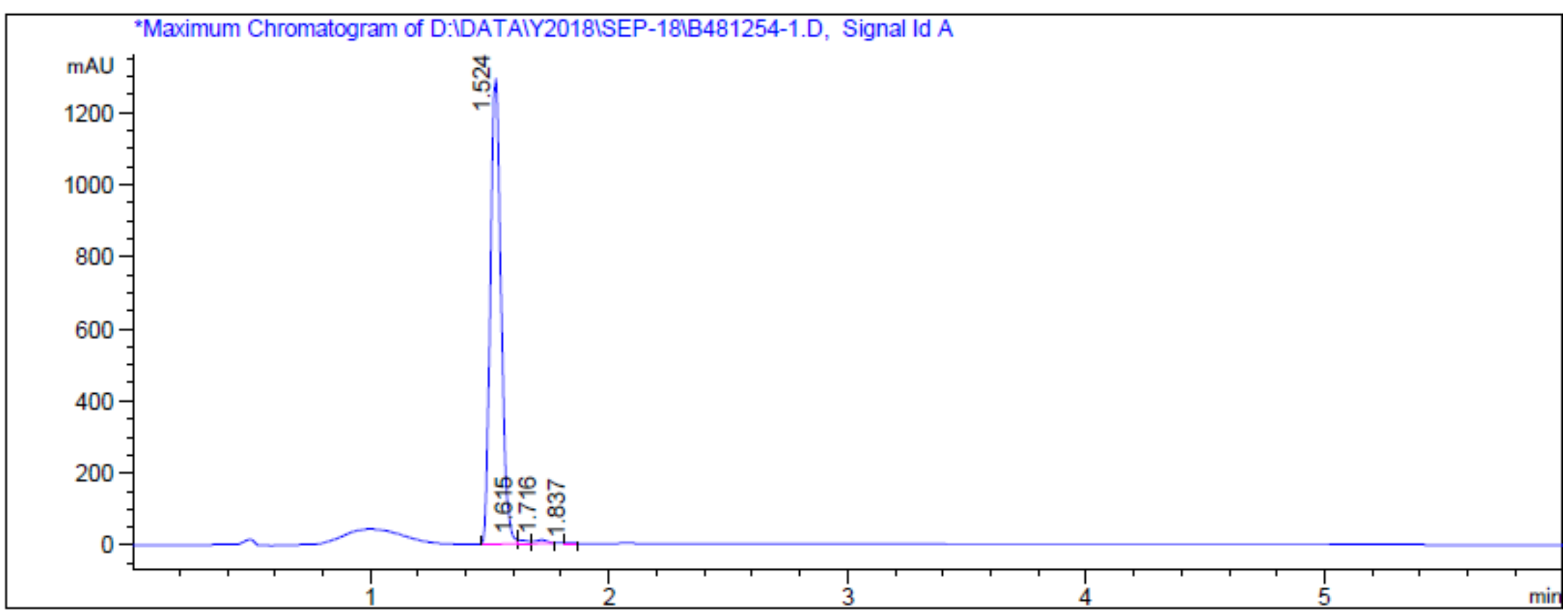

Peak No	RT min	Area	Area %
1	1.524	4028.892	98.270
2	1.615	30.124	0.735
3	1.716	36.724	0.896
4	1.837	4.074	0.099

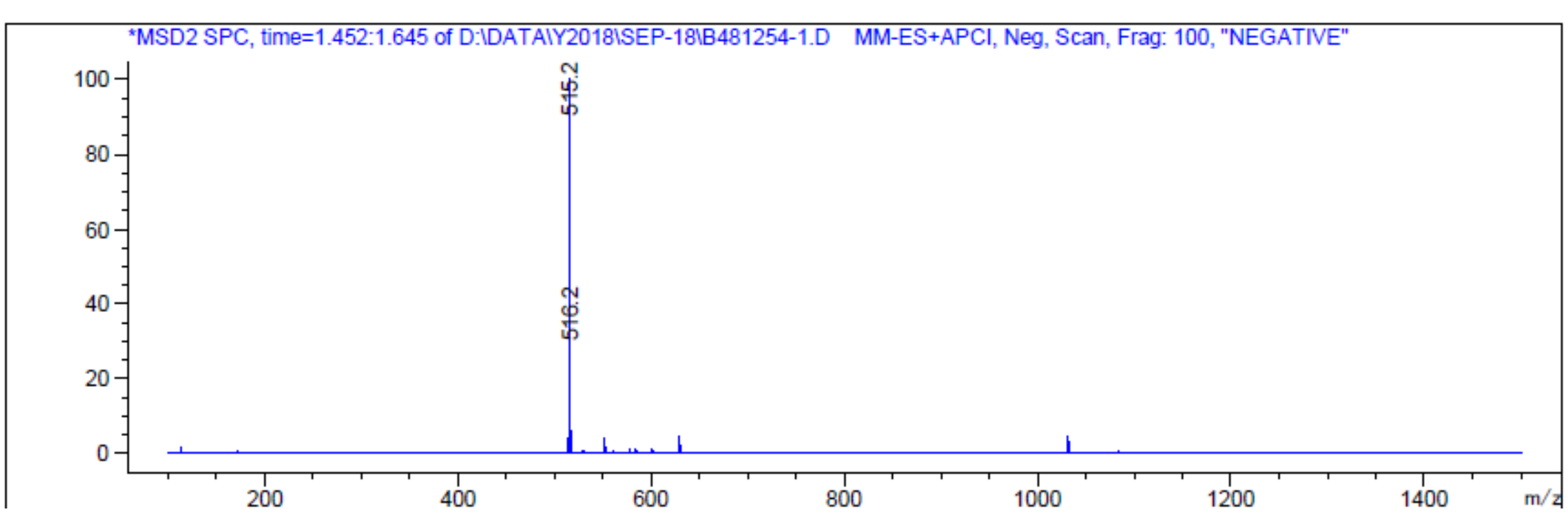

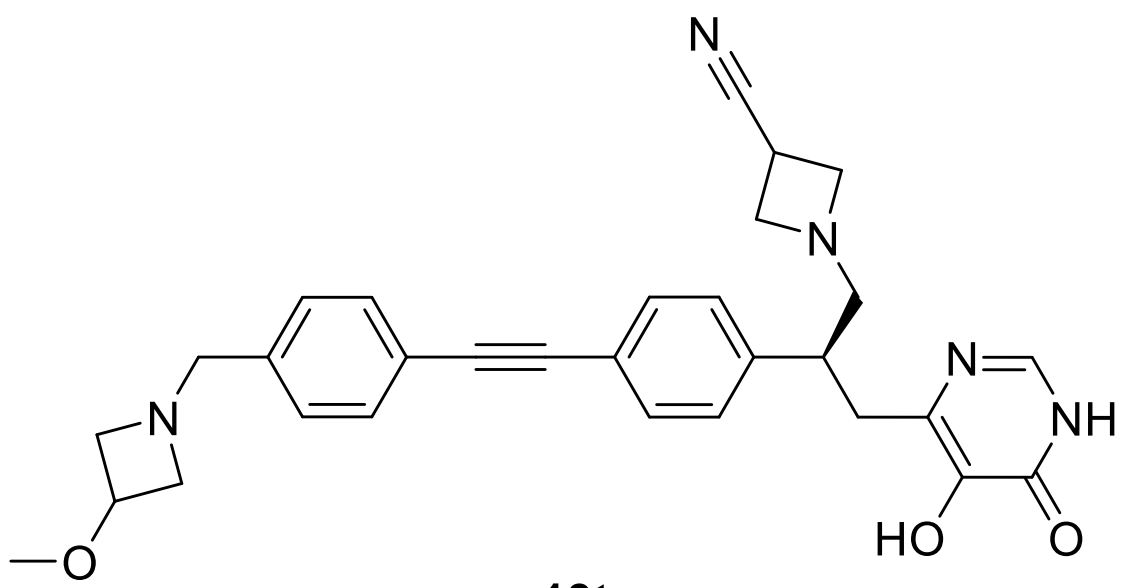

13t

```
Method info  :   Column :Atlantis dC18 (50*4.6)5μ
                 Mobile phase :A :0.1%Formic Acid in H2O B: ACN
                 Flow Rate :1.5 ml/min
                 Time (min)    %B
                 0.0            05
                 2.5            95
                 4.0            95
                 4.5            05
                 6.0            05
```

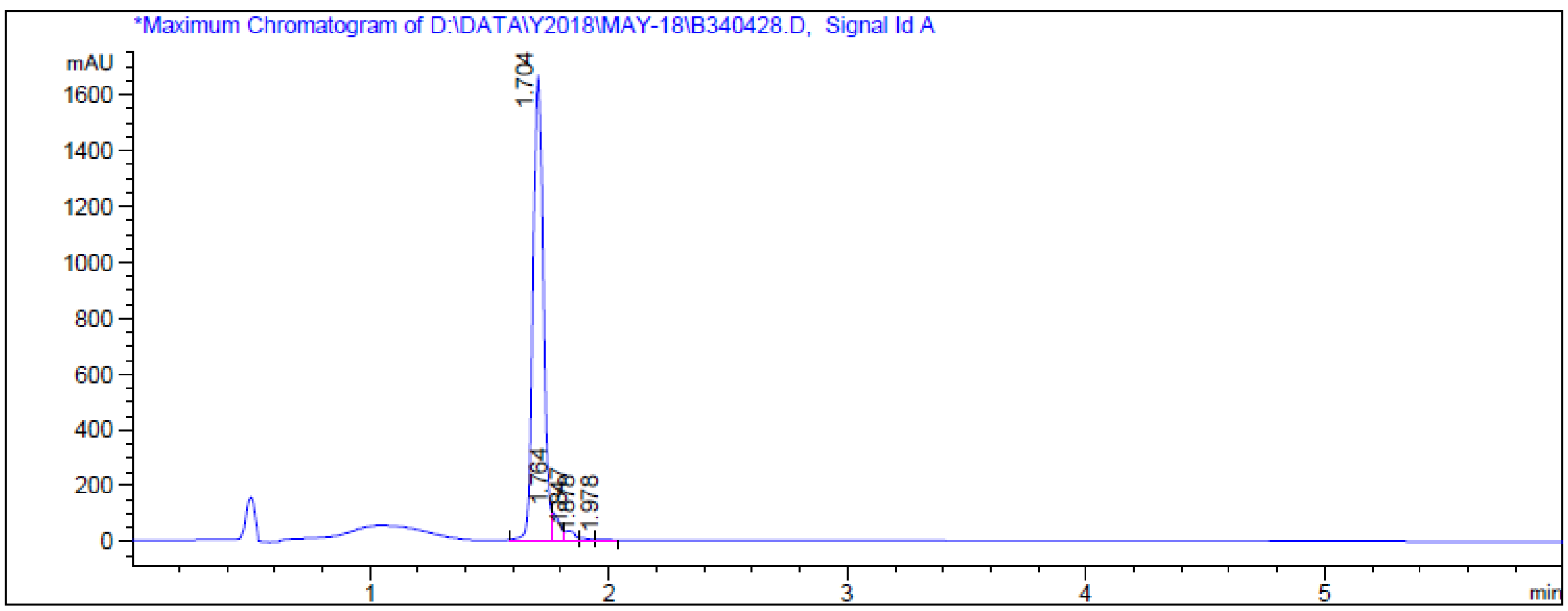

Peak No	RT min	Area	Area %
1	1.704	4992.974	94.264
2	1.764	189.165	3.571
3	1.847	86.390	1.631
4	1.878	15.804	0.298
5	1.978	12.445	0.235

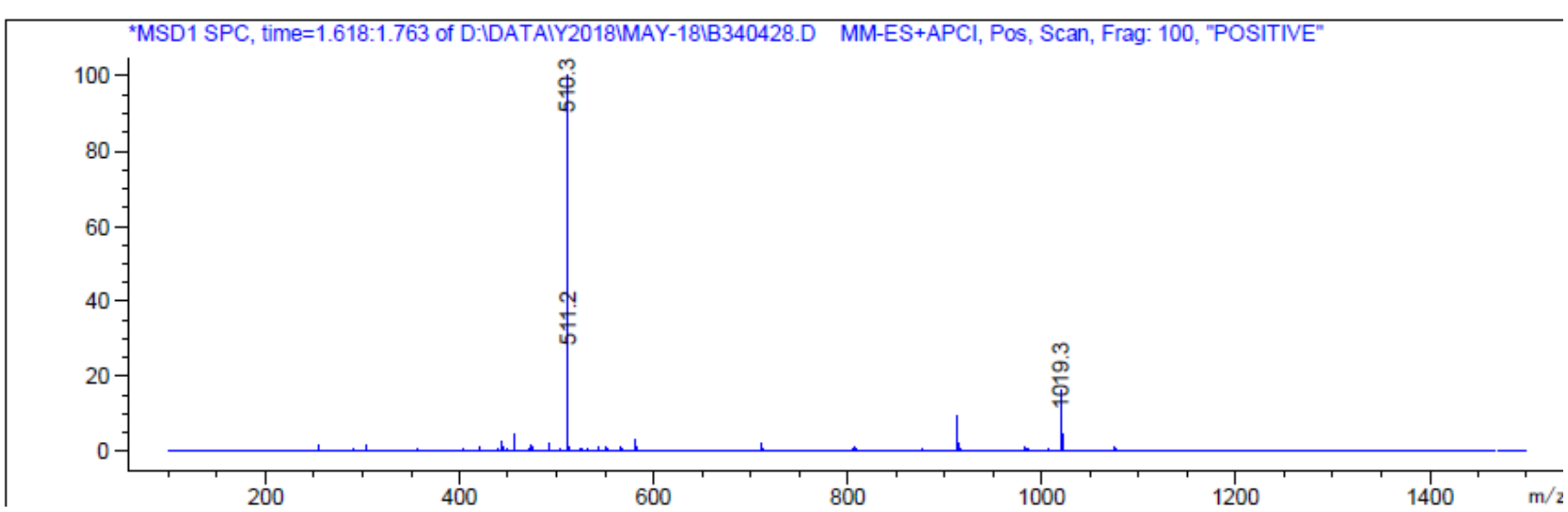

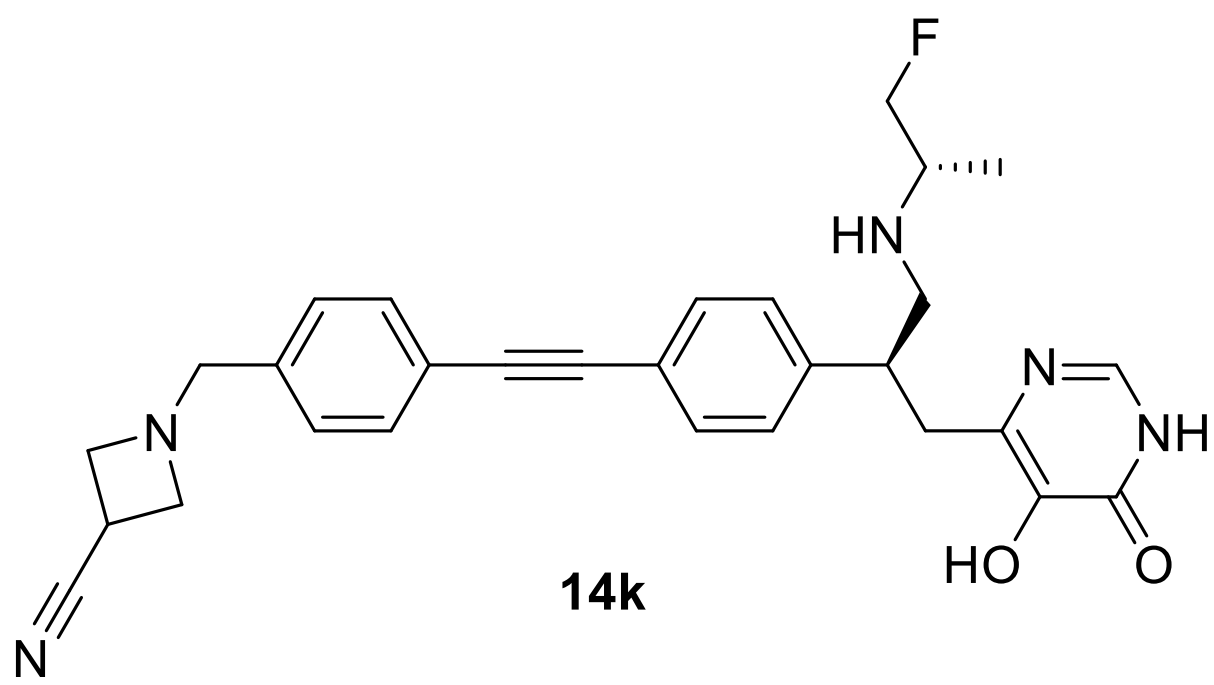

14k

```
Method info  :    Column :Atlantis dC18  (50*4.6)5µ
                  Mobile phase :A :0.1%Formic Acid in H2O B: ACN
                  Flow Rate :1.5 ml/min
                  Time  (min)    %B
                  0.0               05
                  2.5               95
                  4.0               95
                  4.5               05
                  6.0               05
```

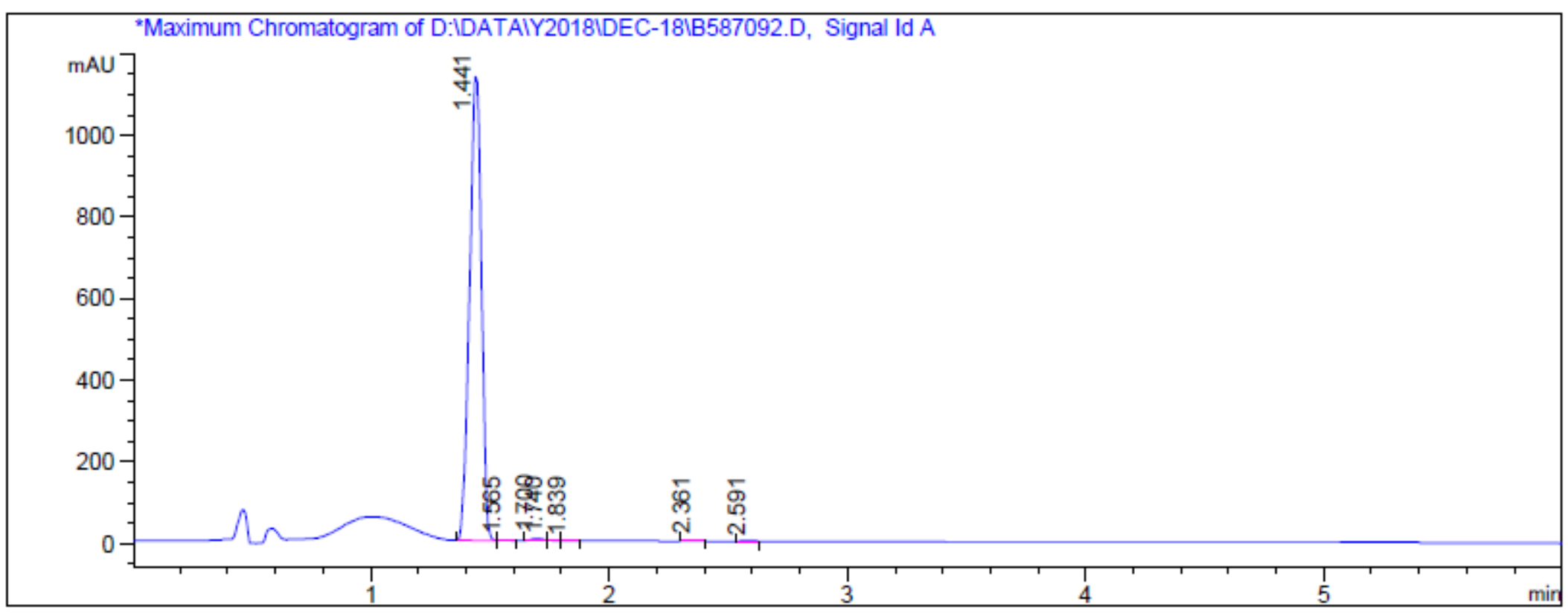

Peak No	RT min	Area	Area %
1	1.441	3934.570	98.950
2	1.565	4.611	0.116
3	1.700	22.779	0.573
4	1.740	3.412	0.086
5	1.839	1.311	0.033
6	2.361	5.398	0.136
7	2.591	4.253	0.107

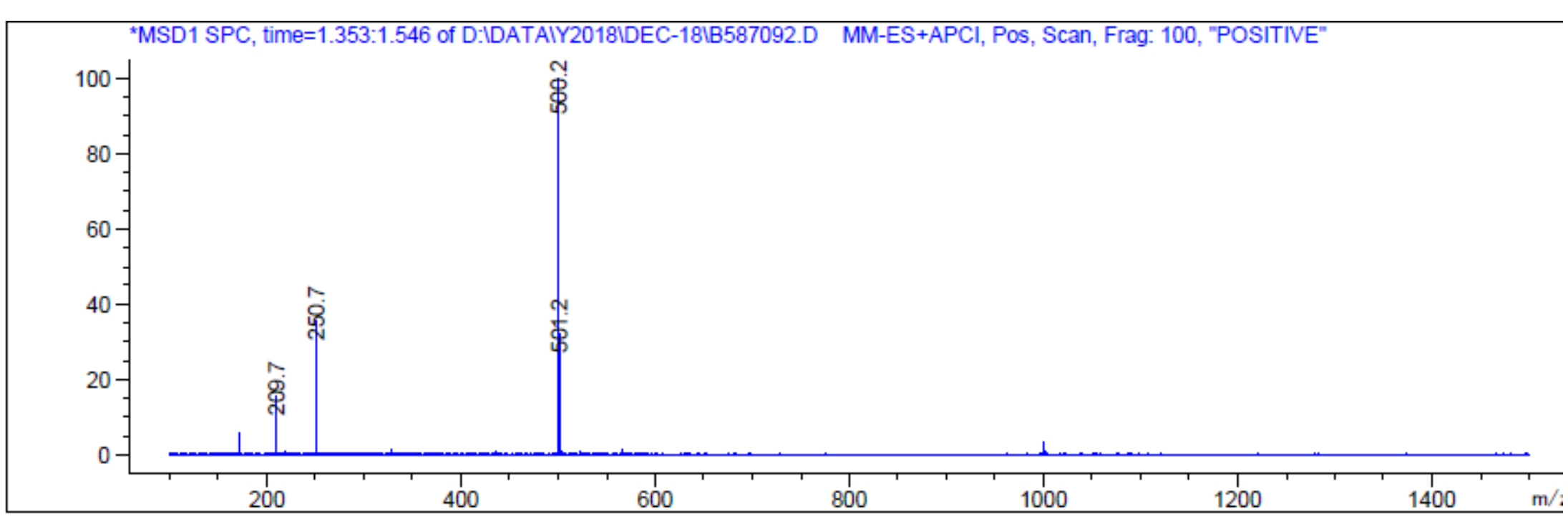

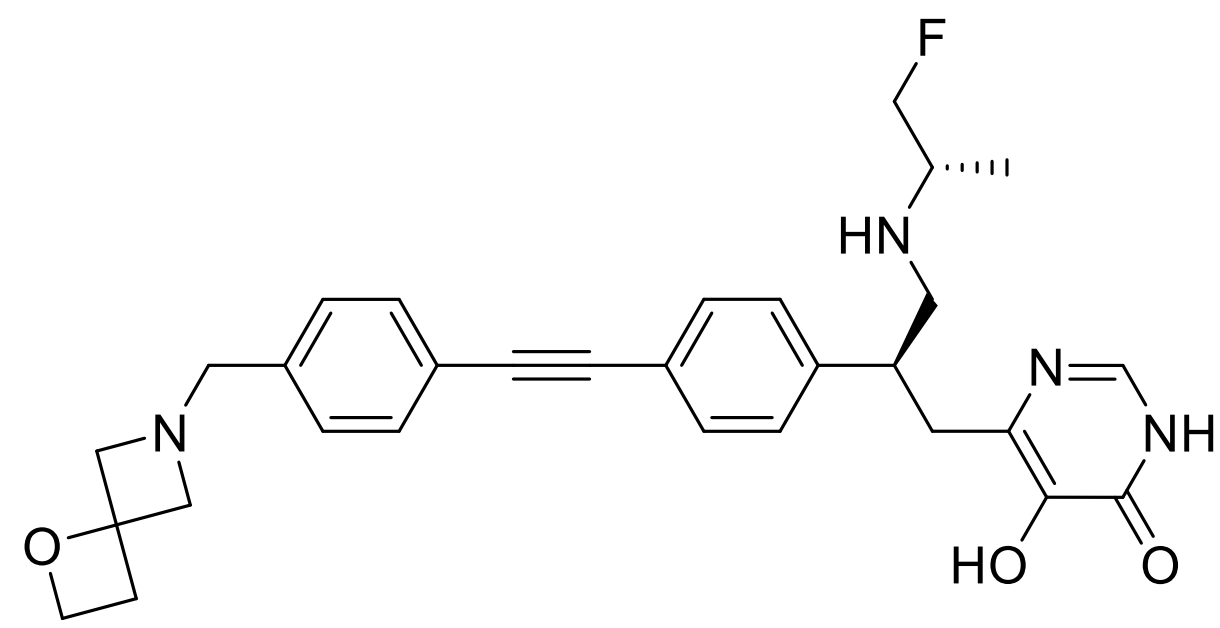

16k

```
Method info   : Column :Atlantis dC18 (50*4.6)5µ
                Mobile phase :A :0.1%Formic Acid in H2O B: ACN
                Flow Rate :1.5 ml/min
                Time (min)    %B
                0.0            05
                2.5            95
                4.0            95
                4.5            05
                6.0            05
```

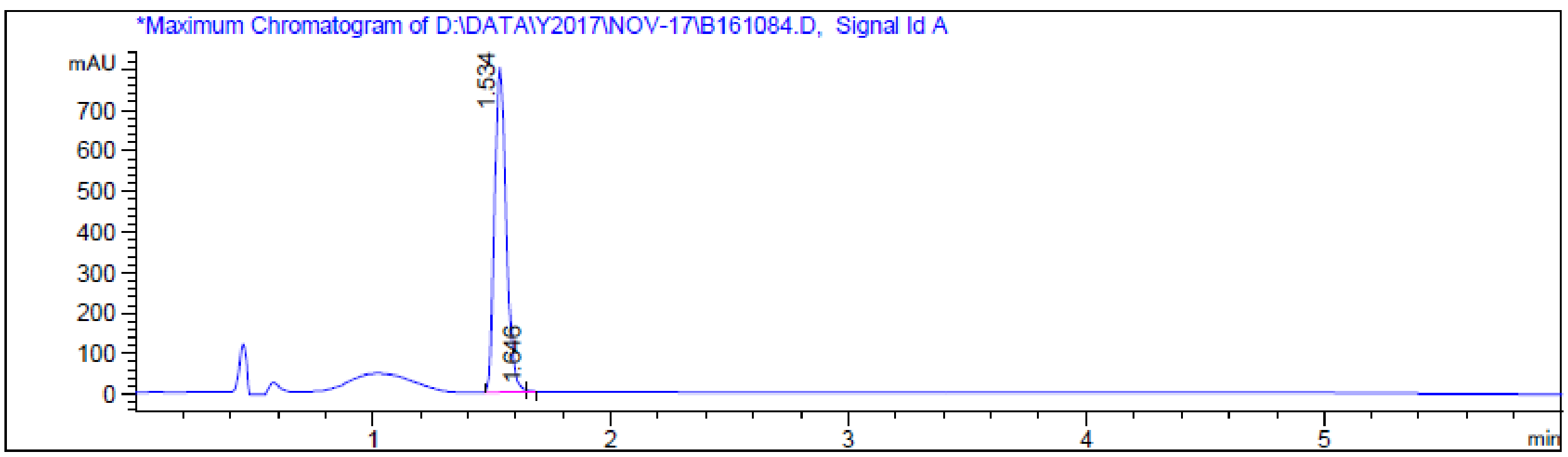

Peak No	RT min	Area	Area %
1	1.534	2.756e+003	99.796
2	1.646	5.637e+000	0.204

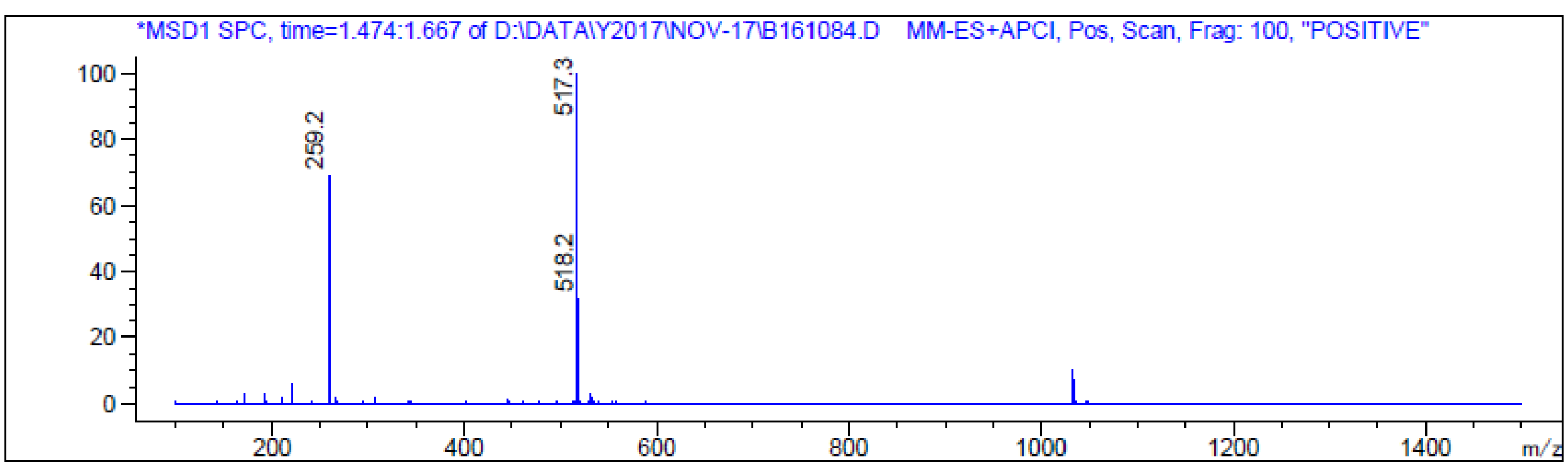

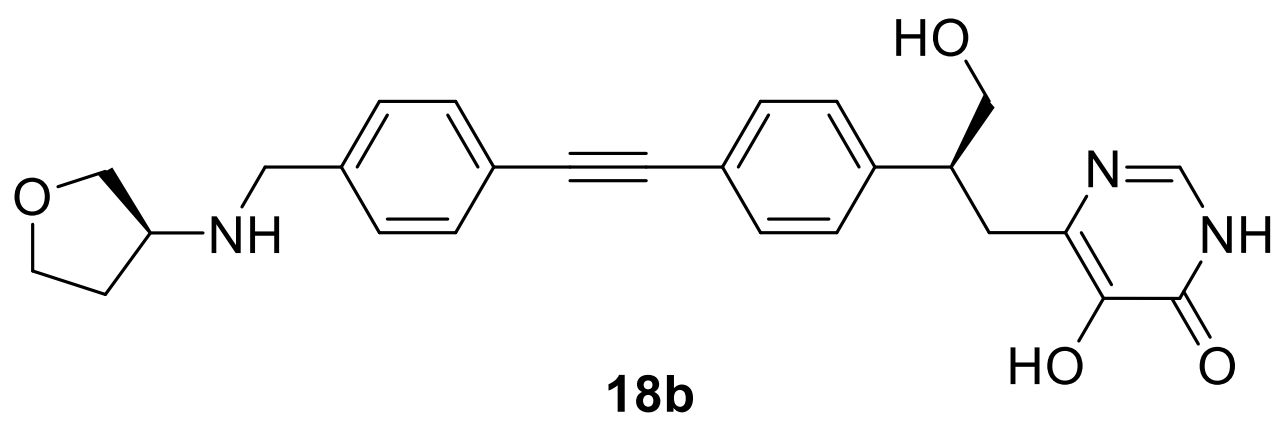

18b

Method info :Column :Atlantis dC18 (50*4.6)5µ
Mobile phase :A :0.1%Formic Acid in H2O B: ACN
Flow Rate :1.5 ml/min

Time (min)	%B
0.0	05
2.5	95
4.0	95
4.5	05
6.0	05

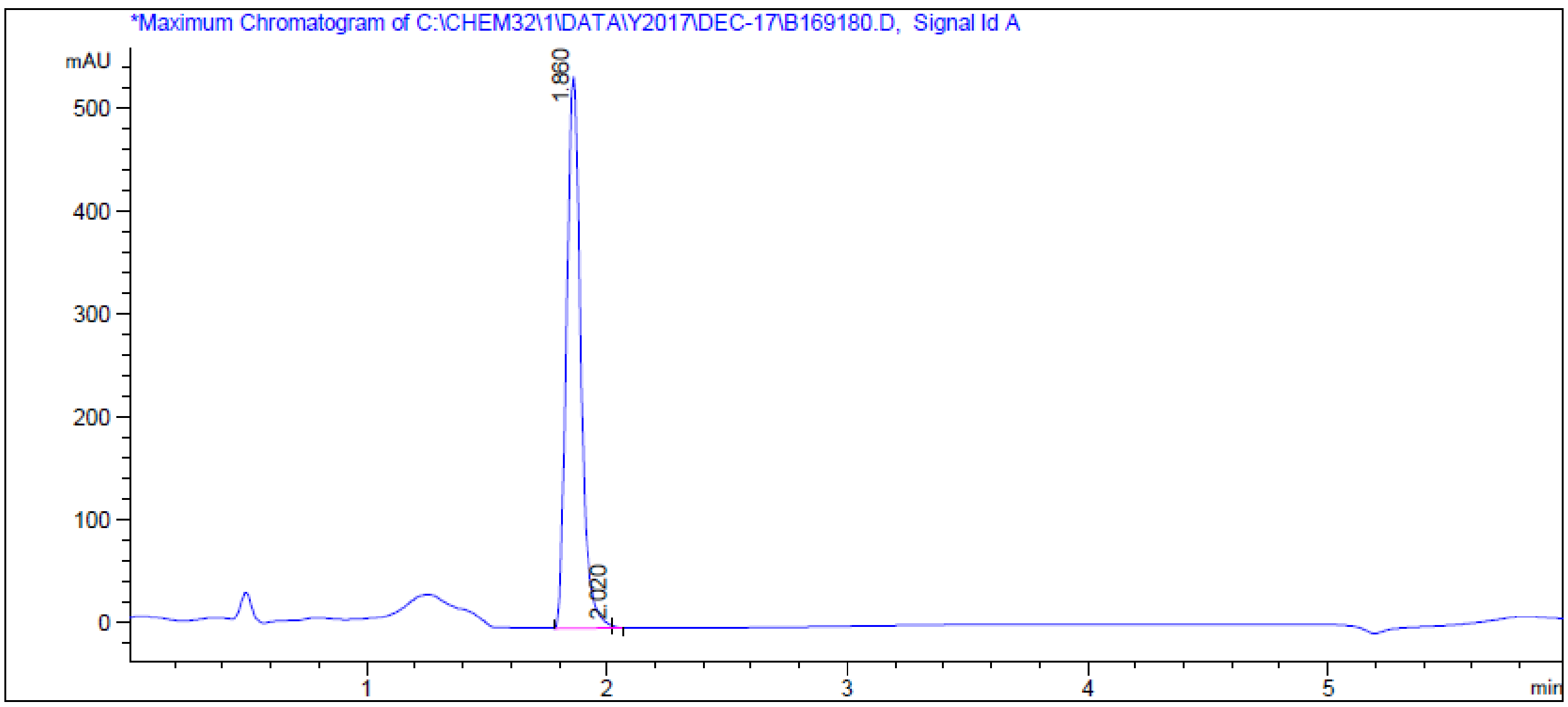

Peak No	RT min	Area	Area %
1	1.860	2.230e+003	99.899
2	2.020	2.260e+000	0.101

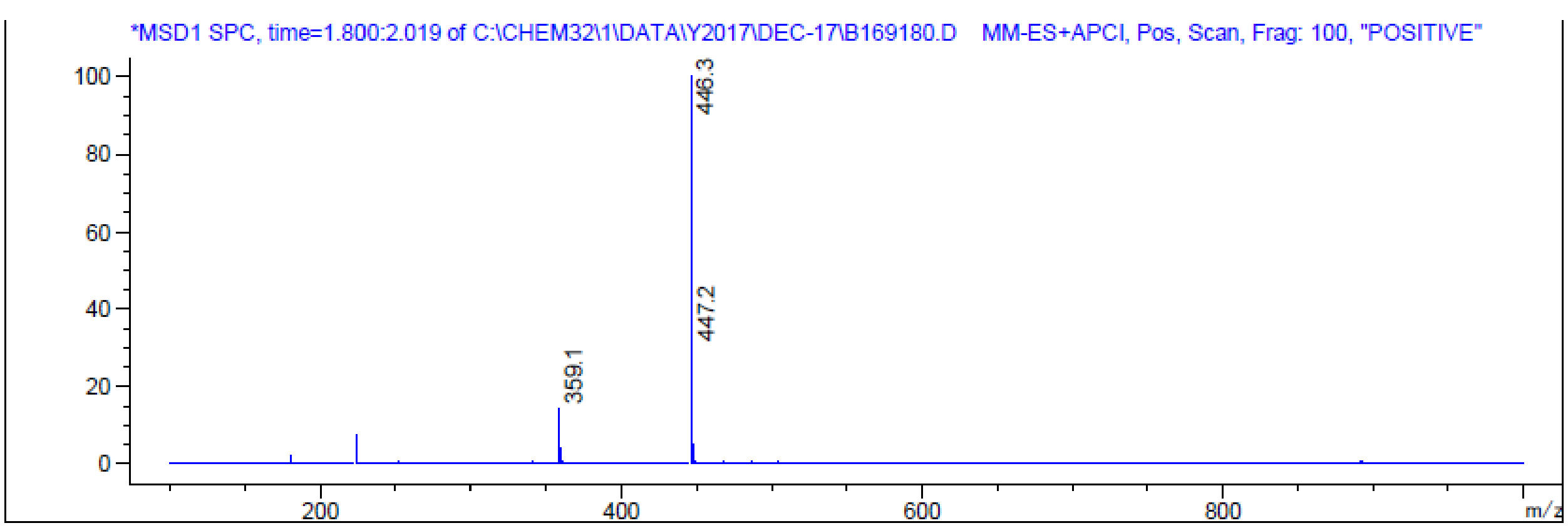

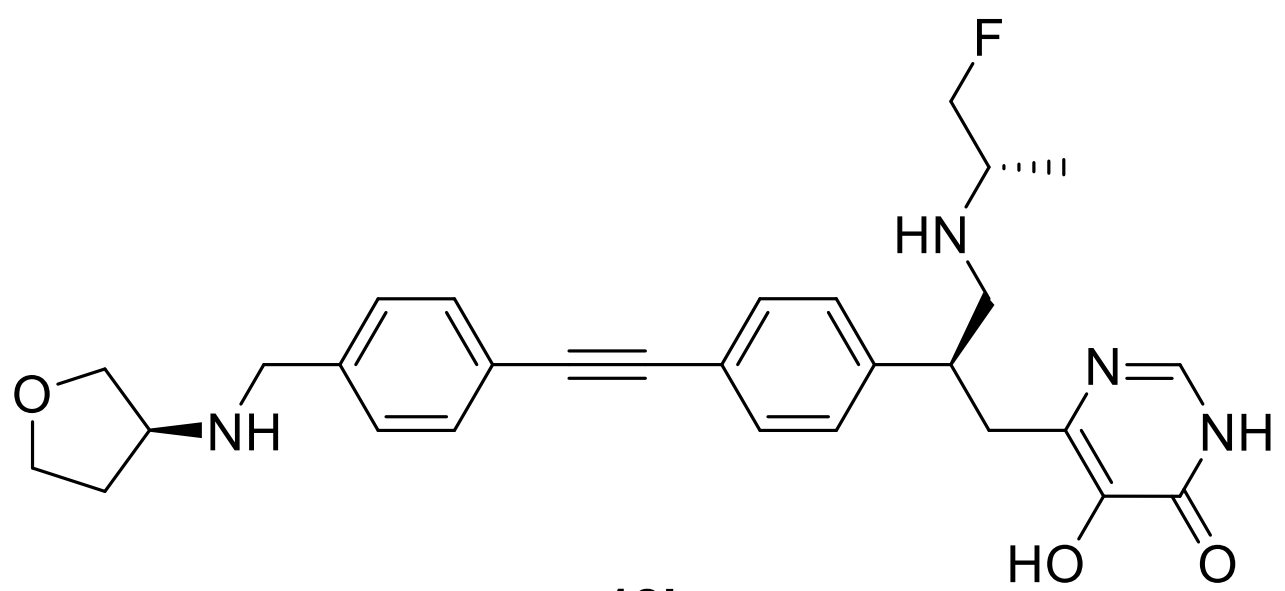

18k

```
Method info  : Column :Atlantis dC18 (50*4.6)5
               Mobile phase :A :0.1%Formic Acid in H2O B: ACN
               Flow Rate :1.5 ml/min
               Time(min) %B
               0.0 05
               2.5 95
               4.0 95
               4.5 05
               6.0 05
```

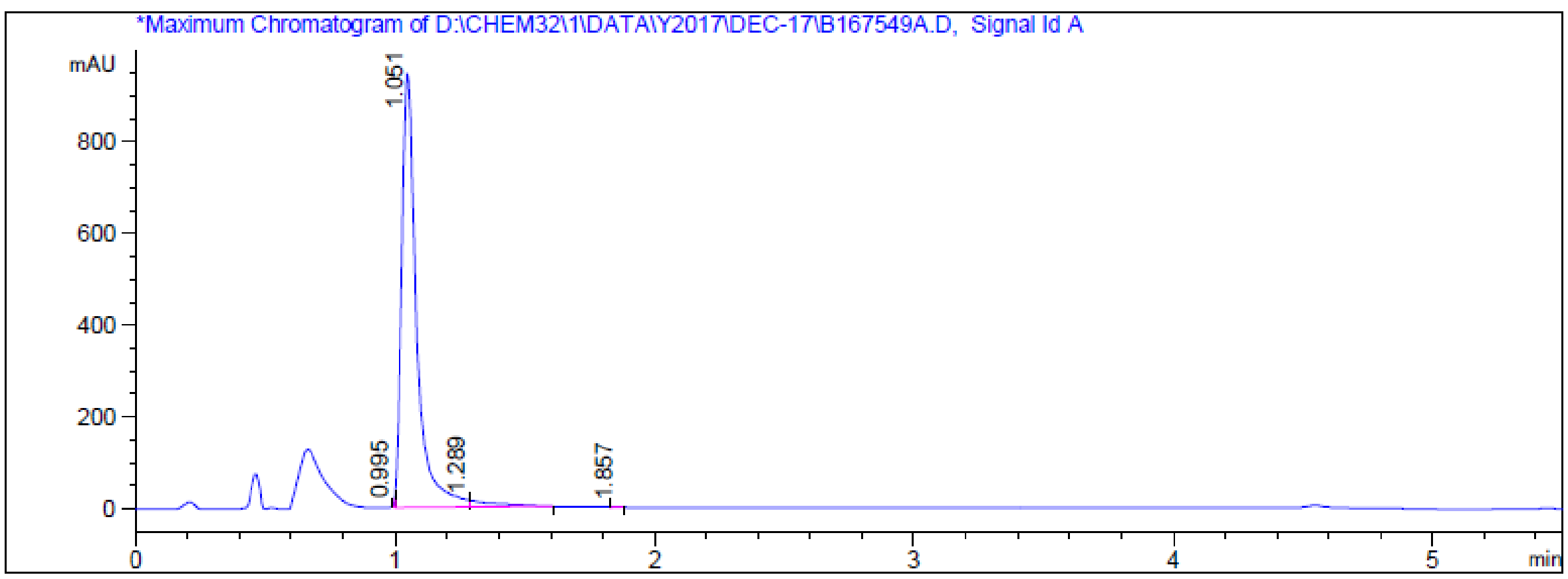

Peak No	RT min	Area	Area %
1	0.995	8.862e+000	0.230
2	1.051	3.747e+003	97.185
3	1.289	9.794e+001	2.540
4	1.857	1.728e+000	0.045

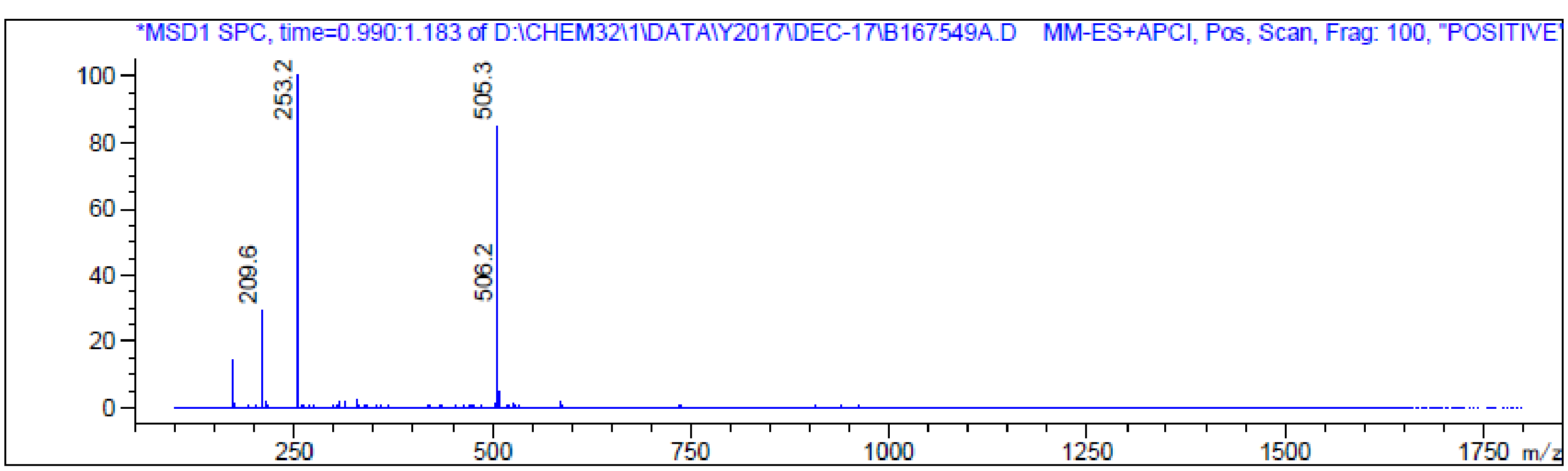

Table S1. Antimicrobial activity of selected compounds compared to analogs with (*R*) stereochemistry at the connection to the pendant.

Cmpd	MIC (µg/mL)				Cmpd	MIC (µg/mL)			
	Ec	*Kp*	*Pm*	*Pa*		*Ec*	*Kp*	*Pm*	*Pa*
**8b**	1	4	8	16	**8q**	0.13	0.5	0.5	8
**(*R*)-8b**	1	4	>32	>32	**(*R*)-8q**	0.13	0.5	4	>32
**8g**	1	4	8	16	**8r**	1	4	8	2
**(*R*)-8g**	8	>32	>32	>32	**(*R*)-8r**	4	>32	32	>32
**8i**	0.5	0.5	2	32	**8s**	0.5	1	8	8
**(*R*)-8i**	2	2	>32	>32	**(*R*)-8s**	1	8	32	>32
**8k**	0.13	0.5	4	8	**8w**	0.5	2	4	16
**(*R*)-8k**	0.5	4	>32	>32	**(*R*)-8w**	2	8	>32	>32
**8m**	0.5	4	8	4	**13k**	0.25	2	8	8
**(*R*)-8m**	2	>32	>32	>32	**(*R*)-13k**	1	4	>32	>32
**8n**	1	4	16	8	**18b**	0.25	2	16	8
**(*R*)-8n**	4	32	>32	>32	**(*R*)-18b**	2	32	>32	>32
**8p**	1	2	8	16					
**(*R*)-8p**	4	16	32	>32					

Ec = *E. coli* ATCC25922; *Kp* = *K. pneumoniae* ATCC13883; *Pm* = *P. mirabilis* ATCC21100; *Pa* = *P. aeruginosa* PAO1

Table S2. Pyridyl substitutions on the hydrophobic backbone result in a drastic loss in activity

Scaffold	Cmpd	X	Y	Z	MIC (µg/mL)			
					Ec	*Kp*	*Pm*	*Pa*
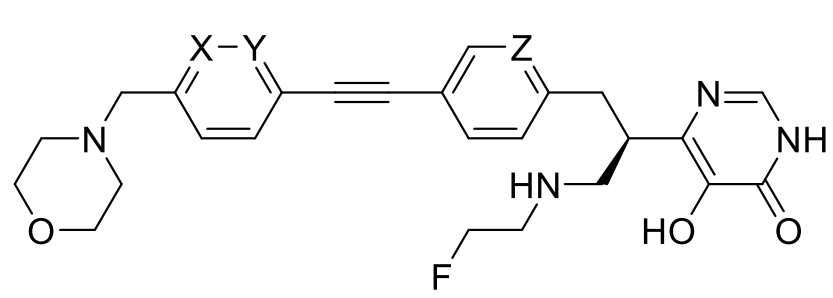	**6**	CH	CH	CH	0.13	0.5	4	8
	**6-Pyr1**	N	CH	CH	32	>32	>32	>32
	**6-Pyr2**	CH	N	CH	>32	>32	>32	>32
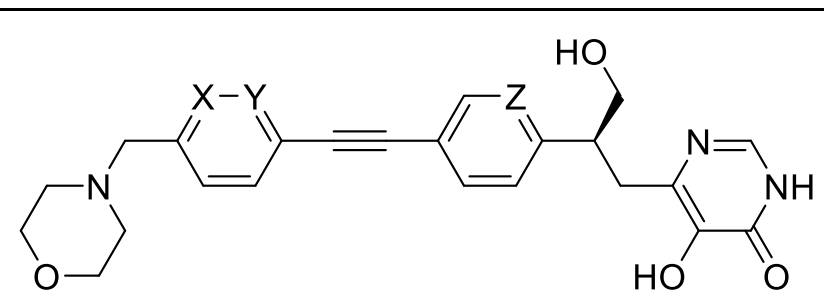	**8b**	CH	CH	CH	0.5	1	4	8
	**8b-Pyr3**	CH	CH	N	8	>32	>32	>32

Table S3. Antimicrobial activity and efficacy of selected compounds compared to analogs with opposite stereochemistry on the pendant.

Cmpd	MIC (µg/mL)				*P. mirabilis* Efficacy[a] (ΔCFU/g, $\log_{10}$)
	Ec	*Kp*	*Pm*	*Pa*	
**8k**	0.13	0.5	4	8	-0.84
**8k-(*R*)**	0.13	0.25	2	4	-0.29
**8m**	0.5	4	8	4	-0.02
**8m-(*S*)**	0.5	8	8	4	-0.04
**8n**	1	4	16	8	-0.10
**8n-(*S*)**	2	8	16	8	NT
**8o**	0.25	1	2	8	NT
**8o-(*S*)**	0.5	1	4	8	NT

[a]Change in bioburden relative to tigecycline (Tig) treatment. *Ec* = *E. coli* ATCC25922; *Kp* = *K. pneumoniae* ATCC13883; *Pm* = *P. mirabilis* ATCC21100; *Pa* = *P. aeruginosa* PAO1

Table S4. Antimicrobial activity of selected compounds against MDR strains.

Strain	Resistance	MIC (µg/mL)						
		**8g**	**8j**	**8k**	**8q**	**8r**	**8s**	**8t**
E. coli CDC346	AK, AMP, AZT, CEF, CIP, CN, COL, CRO, FEP, FOX, LEV, SXT, TET, TOB	0.5	1	0.25	0.5	1	0.5	1
E. coli CDC349	AMP, AZT, CAZ, CEF, CIP, CN, CRO, FEP, FOX, LEV, SXT, TET, TOB	0.5	0.5	0.06	0.13	1	0.5	0.5
K. pneumoniae CDC347	AMP, AZT, CEF, CTX, FOX, CAZ, CRO, CIP, ERT, IMI, LEV, MEM, SXT, TET, TOB, TZP	32	8	4	8	32	8	8
K. pneumoniae CDC361	AK, AMP, AZT, CEF, CTX, FOX, CAZ, CRO, CIP, ERT, IMI, LEV, MEM, SXT, TOB, TZP	4	8	2	2	4	2	4
P. mirabilis CDC377	AMP, CEF, CIP, CN, COL, CRO, CTX, ERT, FEP, IMI, LEV, MEM, NIT, SXT, TET, TIG, TOB	8	4	4	4	8	8	4

AK, amikacin; AMP, ampicillin; AZT, aztreonam; CAZ, ceftazidime; CEF, cefepime; CIP, ciprofloxacin; CN, gentamicin; COL, colistin; CRO, ceftriaxone; CTX, cefotaxime; ERT, ertapenem; FEP, cefepime; FOX, cefoxitin; IMI, imipenem; LEV, levofloxacin; MEM, meropenem; NIT, nitrofurantoin; SXT, trimethoprim/sulfamethoxazole; TET, tetracycline; TIG, tigecycline; TOB, tobramycin; TZP, piperacillin-tazobactam

Table S5. Refinement statistics for LpxC cocrystal structures. Values in parenthesis indicate those for the highest-resolution bin.

**Complex**	*Pa*LpxC:**6**	*Ec*LpxC:**7a**
**PDB ID**	29AH	29AI
**Unit Cell**	Monoclinic<br>a = 35.57 Å; b = 79.08 Å;<br>c = 50.78 Å; β = 92.38°	Orthorhombic<br>a = 91.66 Å; b = 146.74 Å;<br>c = 99.71 Å
**Space Group**	$P2_1$	$P2_12_12$
**Resolution (Å)**	42.69 – 2.20	99.71 - 3.00
**Completeness (%)**	75.5	99.98
$\mathbf{R_{work}}$	0.202	0.245
$\mathbf{R_{free}}$	0.264	0.270
**Complex**	*Pa*LpxC:**8k**	*Pa*LpxC:**8t**
**PDB ID**	29AK	10RY
**Unit Cell**	Monoclinic<br>a = 35.96 Å; b = 66.80 Å;<br>c = 64.15 Å; β = 90.55°	Hexagonal<br>a = b = 108.75 Å;<br>c = 58.26 Å
**Space Group**	$P2_1$	$P6_3$
**Resolution (Å)**	64.21 – 1.50	35.62 - 1.90
**Completeness (%)**	66.1	99.94
$\mathbf{R_{work}}$	0.185	0.198
$\mathbf{R_{free}}$	0.242	0.230

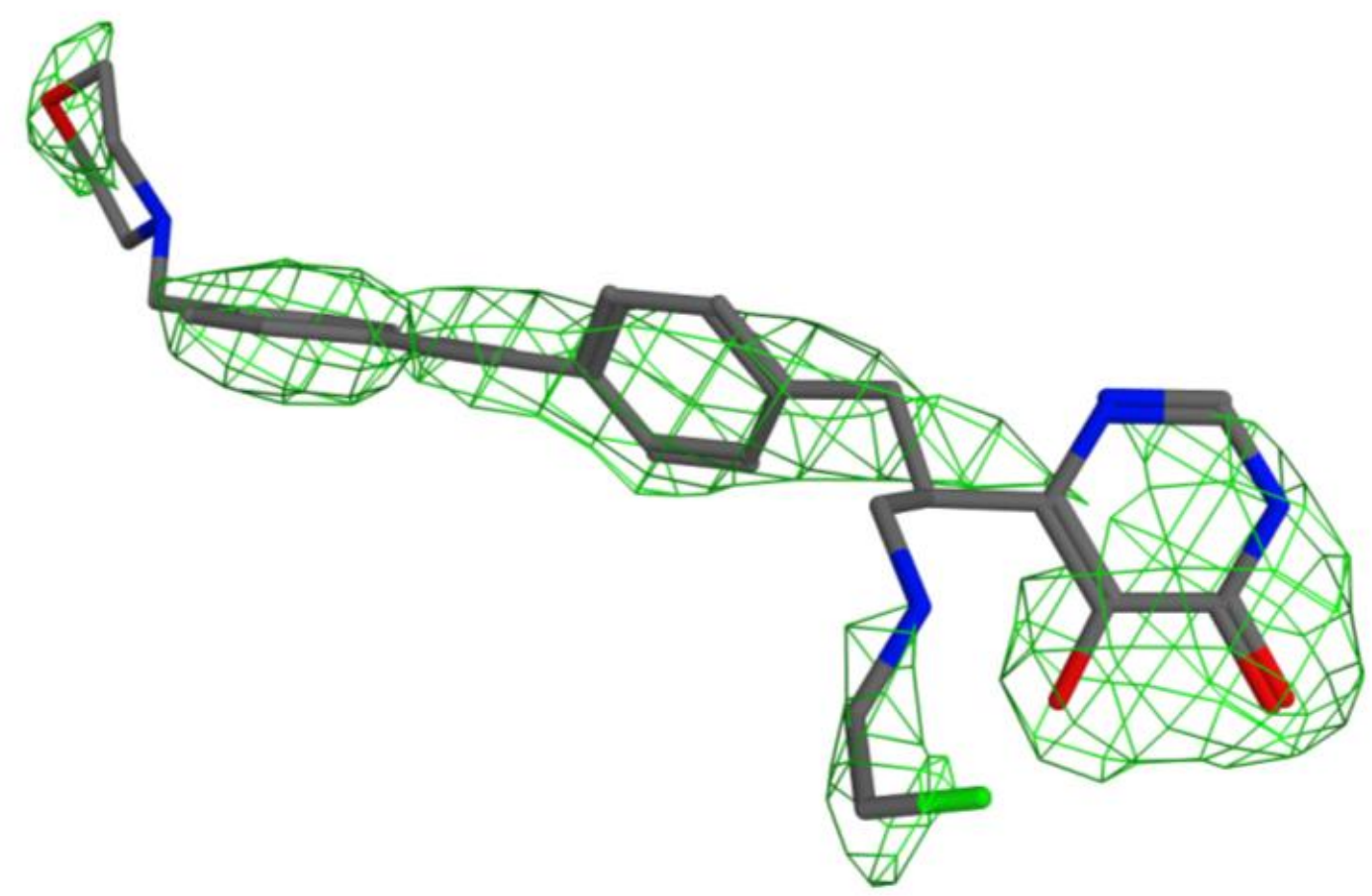

Figure S1. Inhibitor electron density (omit F_o-F_c map contoured at 3σ) for the *Pa*LpxC:**4** crystal structure.

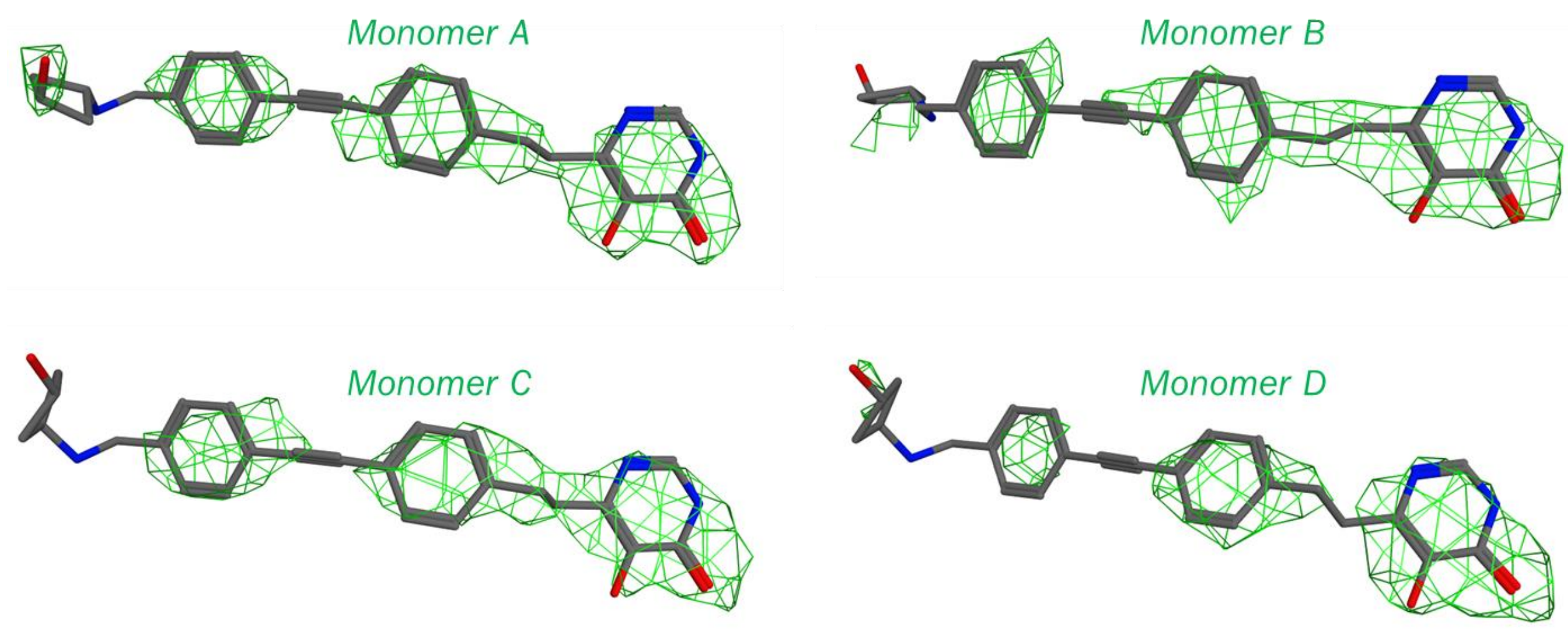

Figure S2. Inhibitor electron density (omit F_o-F_c maps contoured at 3σ) for the four monomers in the *Ec*LpxC:**5a** crystal structure.

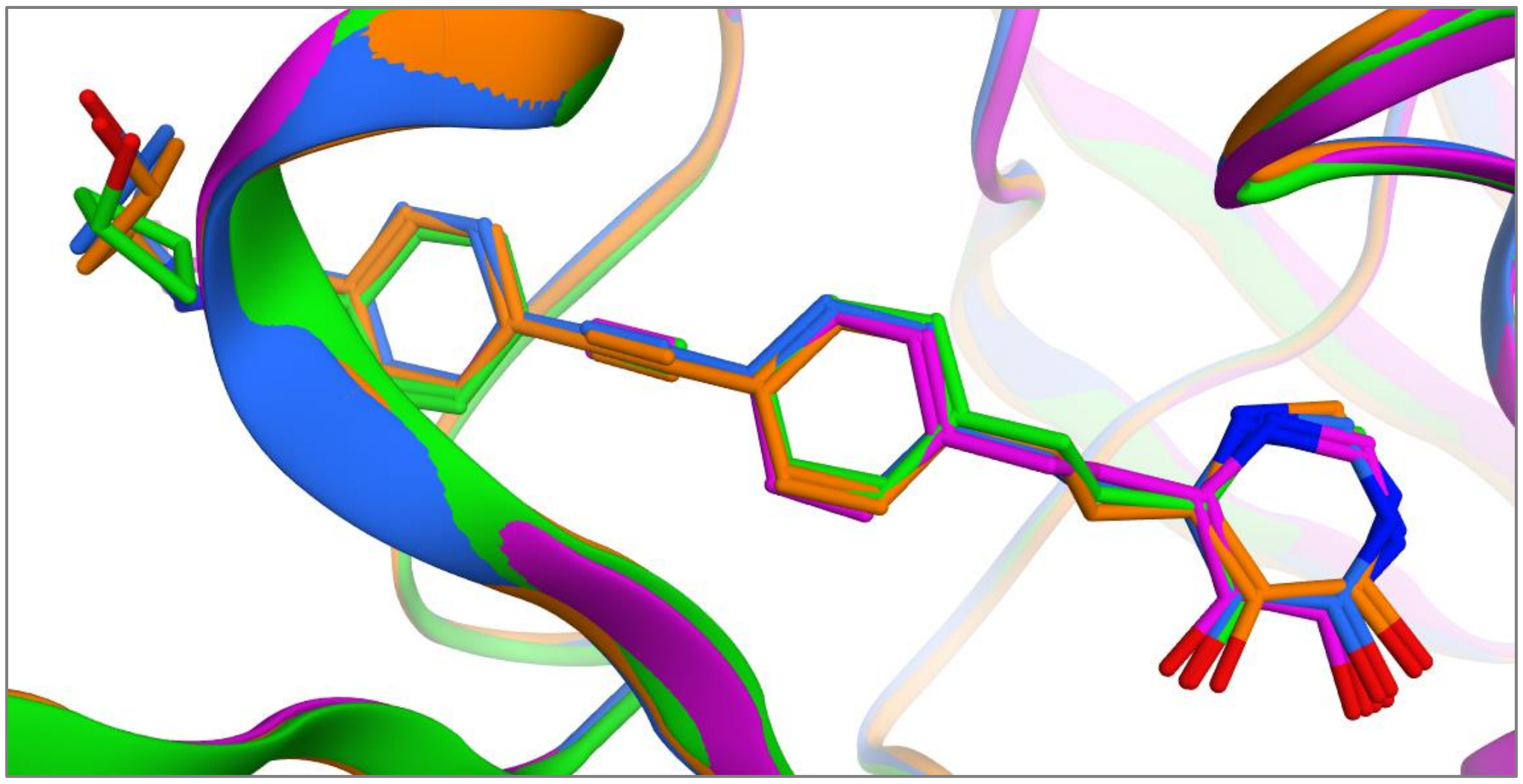

Figure S3. Overlay of the four active sites in the *Ec*LpxC:**5a** crystal structure showing a consistent pose for the MBP, linker, and diphenylacetylene backbone.

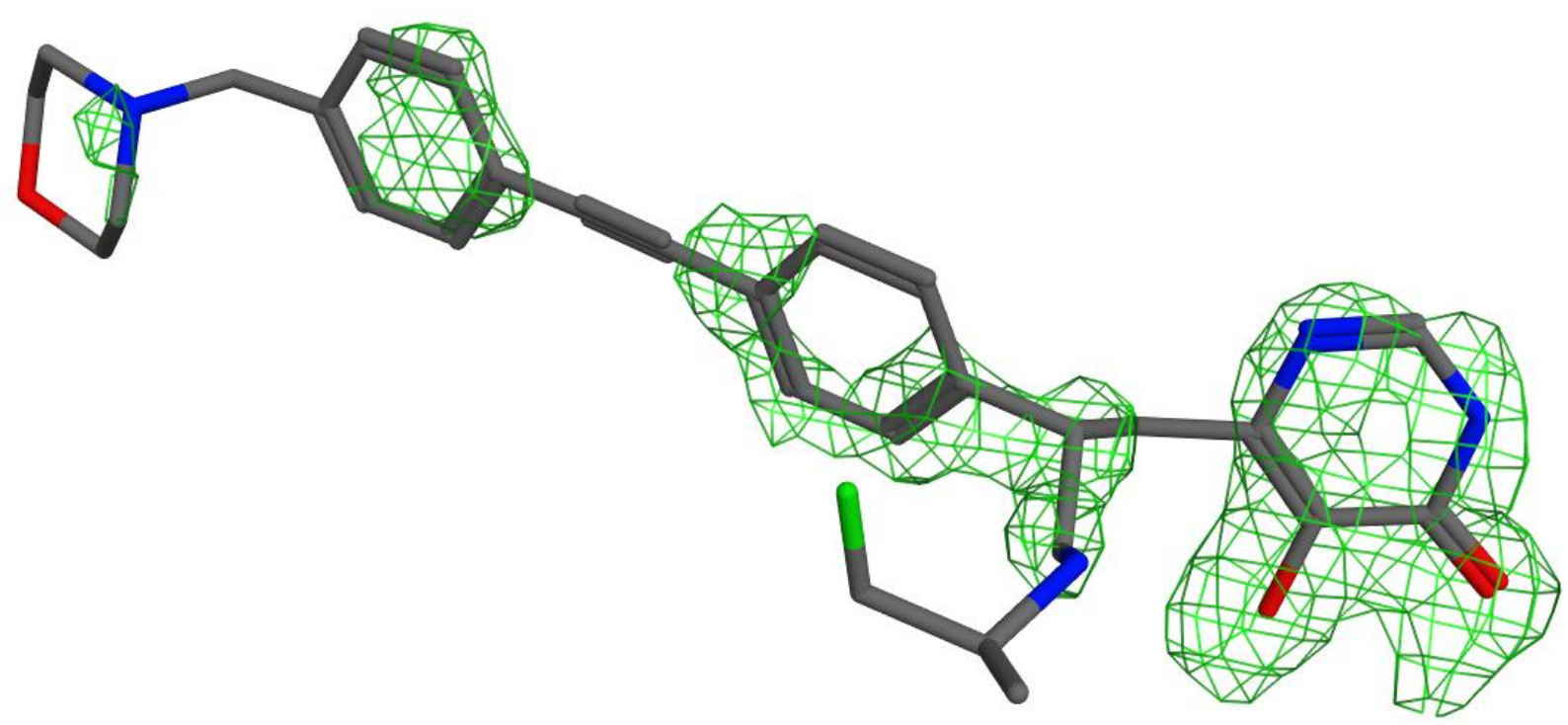

Figure S4. Inhibitor electron density (omit F_o-F_c map contoured at 3σ) for the *Pa*LpxC:**6k** crystal structure.

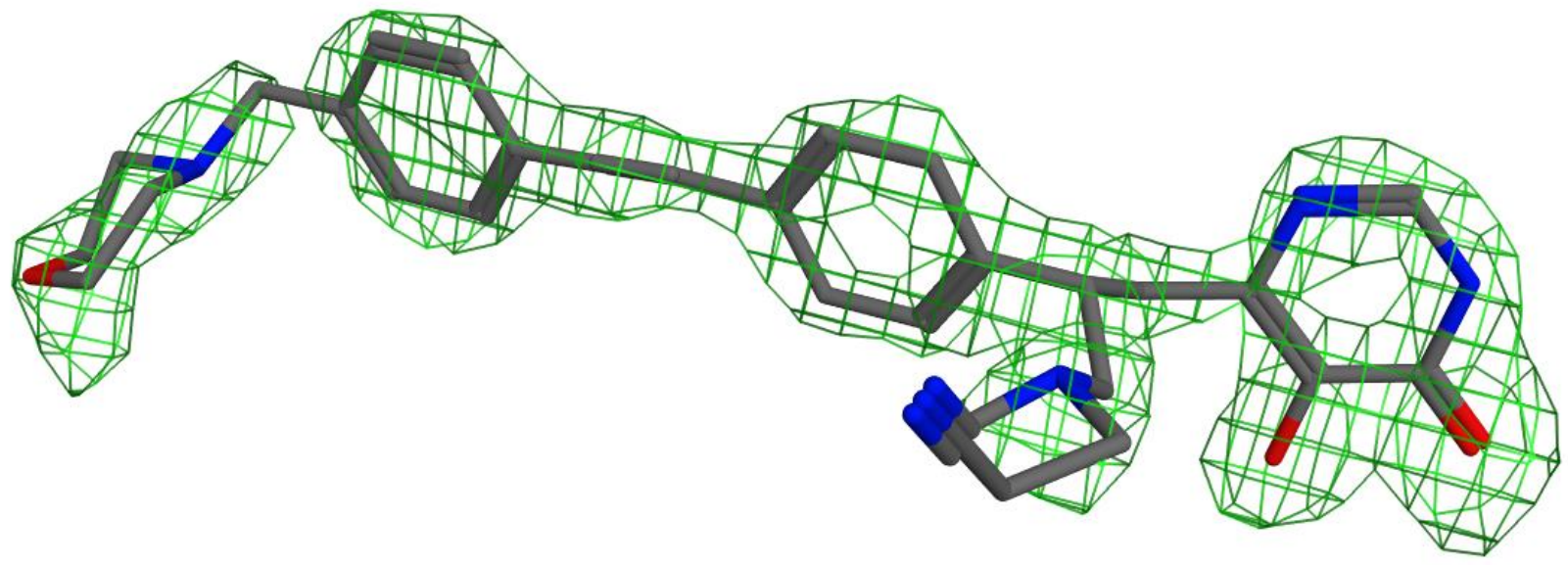

Figure S5. Inhibitor electron density (omit F_o-F_c map contoured at 4σ) for the *Pa*LpxC:**6t** crystal structure.

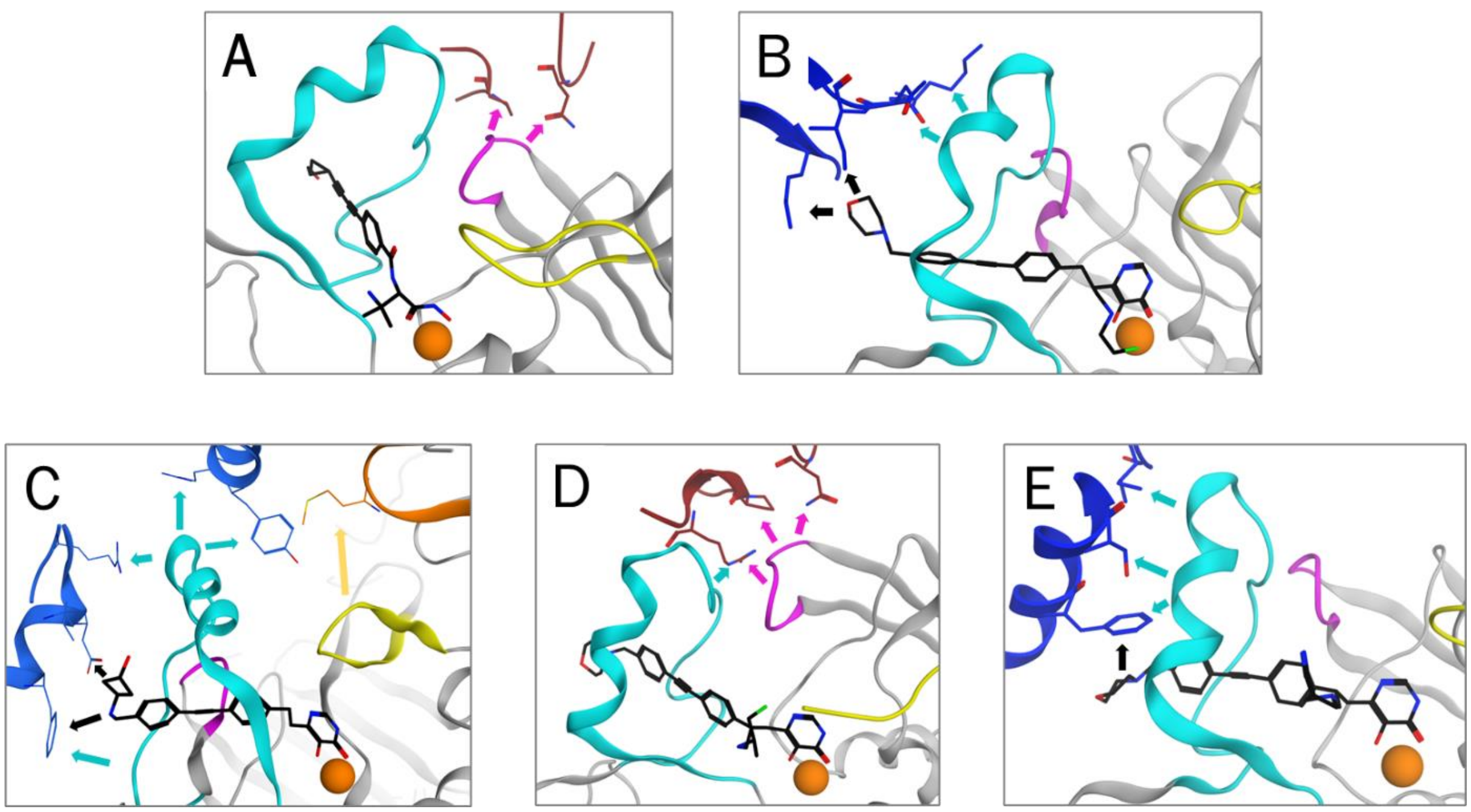

Figure S6. Differences in crystal packing interactions involving key active site regions in the *Pa*LpxC:**2** (A) *Pa*LpxC:**4** (B), *Ec*LpxC:**5a** (C), *Pa*LpxC:**6k** (D), and *Pa*LpxC:**6t** (E) complexes.

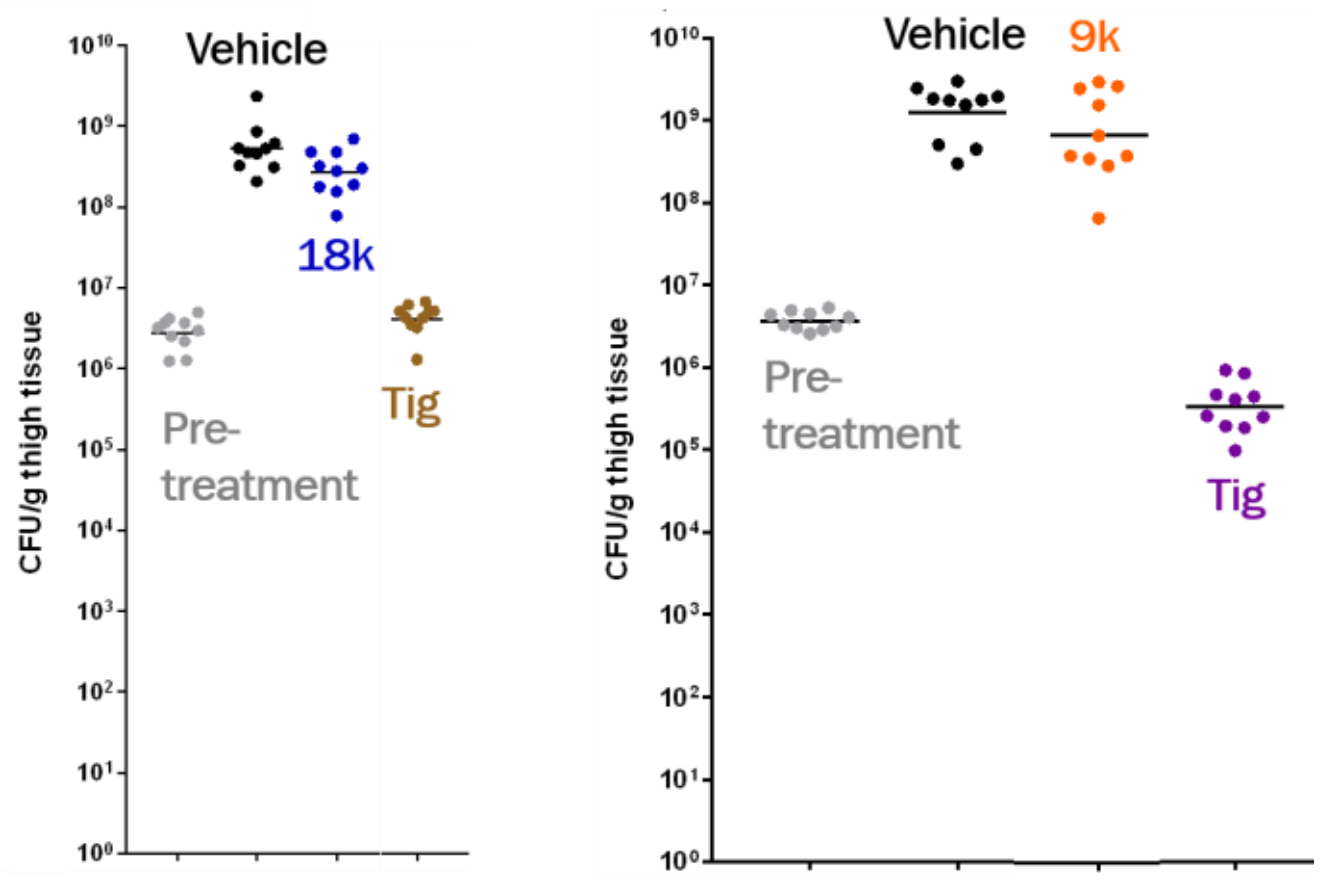

Figure S7. Compounds **9k** and **18k** did not show efficacy in a murine thigh infection model of *K. pneumoniae* infection (ATCC13883, MICs of 1 and 4 µg/mL, respectively)

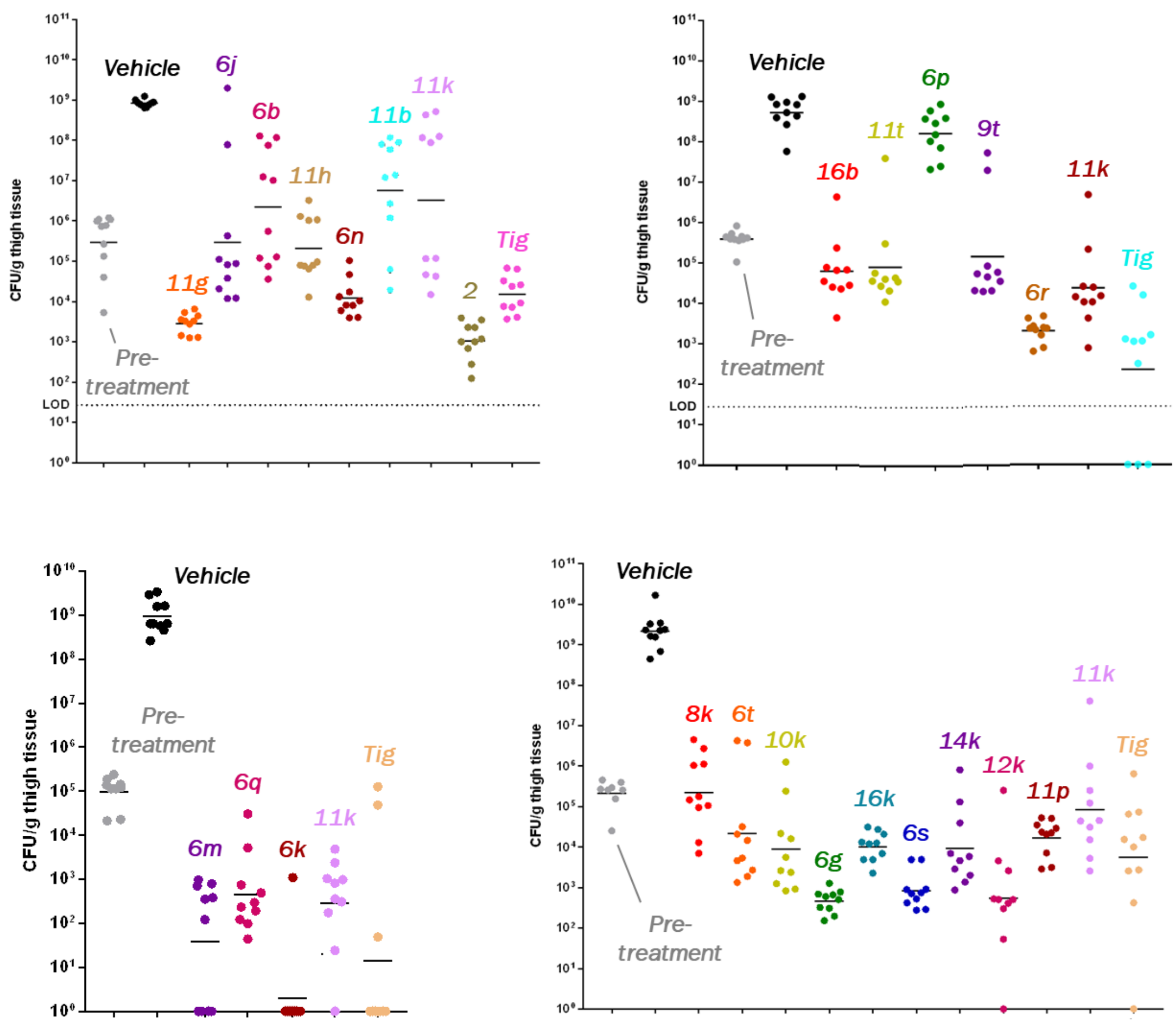

Figure S8. Scatter plots for individual *in vivo* efficacy studies.
